# Massive expansion and functional divergence of innate immune genes in a protostome

**DOI:** 10.1038/srep08693

**Published:** 2015-03-03

**Authors:** Linlin Zhang, Li Li, Ximing Guo, Gary W. Litman, Larry J. Dishaw, Guofan Zhang

**Affiliations:** 1 Institute of Oceanology, Chinese Academy of Sciences, Qingdao 266071, China; 2 Haskin Shellfish Research Laboratory, Institute of National and Local Marine and Coastal Sciences, Rutgers University, Port Norris, NJ 08349, USA; 3 Morsani College of Medicine, Department of Pediatrics, University of South Florida, St. Petersburg, FL 33701, USA; 4 All Children's Hospital Johns Hopkins Medicine, St. Petersburg, FL 33701, USA

## Abstract

The molecules that mediate innate immunity are encoded by relatively few genes and exhibit broad specificity. Detailed annotation of the Pacific oyster (*Crassostrea gigas*) genome, a protostome invertebrate, reveals large-scale duplication and divergence of multigene families encoding molecules that effect innate immunity. Transcriptome analyses indicate dynamic and orchestrated specific expression of numerous innate immune genes in response to experimental challenge with pathogens, including bacteria, and a pathogenic virus. Variable expression of individual members of the multigene families encoding these genes also occurs during different types of abiotic stress (environmentally-equivalent conditions of temperature, salinity and desiccation). Multiple families of immune genes are responsive in concert to certain biotic and abiotic challenges. Individual members of expanded families of immune genes are differentially expressed under both biotic challenge and abiotic stress conditions. Members of the same families of innate immune molecules also are transcribed in developmental stage- and tissue-specific manners. An integrated, highly complex innate immune system that exhibits remarkable discriminatory properties and responses to different pathogens as well as environmental stress has arisen through the adaptive recruitment of tandem duplicated genes. The co-adaptive evolution of stress and innate immune responses appears to have an ancient origin in phylogeny.

The distribution in phylogeny of the cells, molecules and interactive processes that effect immune protection is of broad biological interest. In vertebrates, innate immunity, as well as lymphocyte-mediated adaptive immunity, is mediated by various classes of molecules and function in different phases of the response to foreign challenges. Adaptive immunity is a shared character of all vertebrates and is mediated through the somatic rearrangement of genes that give rise to genetically unique receptors expressed on the surface of individual lymphocytes^1^,^2^. Innate immunity is a shared character of both vertebrates and invertebrates and relies on recognition of conserved pathogen-associated molecular patterns (PAMPs) present in microbes by germline encoded pathogen-associated pattern recognition receptors (PAMRs) including: toll-like receptors (TLR), retinoic acid-inducible gene I [RIG-I]-like receptors (RLRs) and NACHT-leucine-rich repeat receptor (NLR)^3^. Upon PAMP recognition, PAMRs activate intracellular signaling pathways, including adaptor molecules, kinases, and transcription factors and trigger proinflammatory and antimicrobial effectors^4^.

A number of different genetic mechanisms increase the diversity and specificity of the innate response of invertebrates, including: massive alternative splicing of Down syndrome cell adhesion molecule in fruit fly (*Drosophila* *melanogaster*)^5^, hypervariation and somatic variation in the fibrinogen-related proteins of the snail (*Biomphalaria glabrata*)^6^ and high allelic diversity in the immunoglobulin (Ig) domains of amphioxus (*Branchiostoma floridae*) variable region-containing chitin-binding proteins^7^ increase the diversity and specificity of the innate response of invertebrates. In both sea urchin (*Strongylocentrotus purpuratus*) and amphioxus^8^,^9^, the diversity and likely specificity of immunity is achieved through large-scale expansion and diversification of multigene families encoding innate immune genes. The mechanisms underlying the expansion and functional diversification of the molecules at the transcriptional level are not understood.

Stress conditions, including challenges to the immune system, also are rapidly changing and highly variable. Stress adaptation likely requires rapid adaptive innovation^10^. Certain immune genes have been shown to have significant roles during abiotic stress responses, including: social stress in primates^11^, nutritional stress in the fruit fly^12^ and temperature stress in the alfalfa leafcutting bee^13^. Functionally divergent immune genes that are expressed during abiotic stress and/or biotic defense may share common pathways and are of broad general interest.

The Pacific oyster (*Crassostrea gigas*) is a member of the lophotrochozoa, a group of protostomes representing a large taxonomic group encompassing several major invertebrate taxa such as the Mollusca. As a sessile, filter-feeder exposed to a wide range of biotic (bacterial and viral) and abiotic stresses (dynamic variation in temperature, salinity and prolonged desiccation), the oyster represents an attractive model for studying the relationship of immunity and stress adaptation^14^ and complements findings from an important lophotrocozoan system, *B. glabrata*, from which it was recently shown that a large proportion of transcripts are challenge-specific with some level of functional divergence noted from expanded gene families^15^. We present here a comprehensive genomic annotation and transcriptomic analyses of the large subset of genes that constitute the oyster immune system and determine that the lineage-specific expansion of genes is associated not only with differential responses to pathogens but also with differential expression under environmental stress conditions that emulate those of its natural habitat, as well as during the course of developmental maturation.

## Results

### Immune gene family expansion

**Figure 1:**
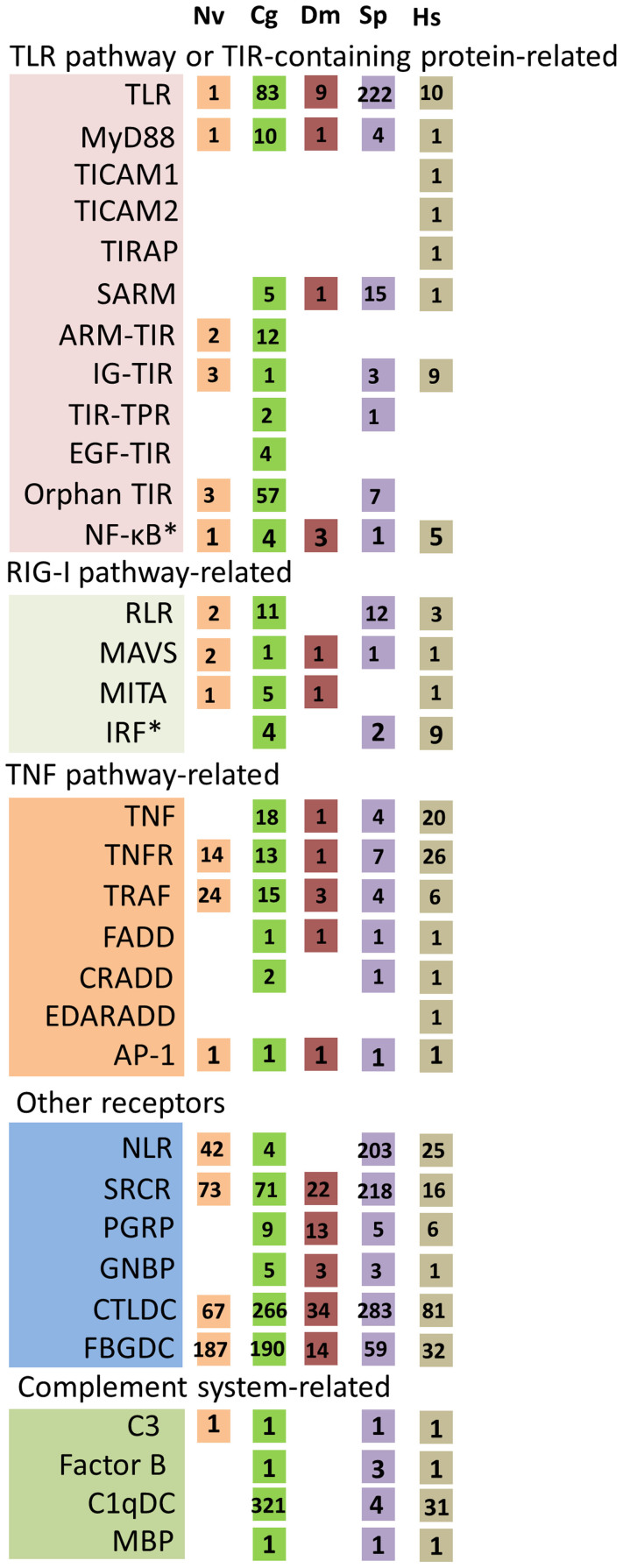
Expansion of innate immune genes in the oyster. The species analyzed are: sea anemone (*Nematostella vectensis* - NV.), Pacific oyster (*Crassostrea gigas* - CG), fruit fly (*Drosophila melanogaster* - DM), sea urchin (*Strongylocentrotus purpuratus* - SP) and human (*Homo sapiens* - HS). Numbers of gene homologs in oyster were identified and compared to those reported for sea anemone, *Drosophila* (http://cegg.unige.ch/Insecta/immunodb), sea urchin^1^ and human (http://genome.ucsc.edu/). *TLR*, Toll-like receptor; *MyD88*, myeloid differentiation primary response gene 88; *TICAM1/2*, Toll-interleukin 1 receptor domain (TIR)-containing adaptor molecule-1/2; *TIRAP*, Toll/interleukin-1 receptor domain-containing adapter protein; *SARM*, sterile-alpha and armadillo motif-containing protein; *ARM-TIR,* proteins with armadillo (ARM) and TIR domains; *IG-TIR*, proteins with immunoglobulin (IG) and TIR domains; *TIR-TPR*, proteins with TIR and tetratricopeptide repeat (TPR) domains; *EGF-TIR*, proteins with epidermal growth factor (EGF) and TIR domains; *NF-κB*, nuclear factor-KappaB; *RLR*, RIG-1-like receptor; *MAVS*, mitochondrial antiviral-signaling protein; *MITA*, transmembrane protein 173; *IRF*, interferon regulatory factor; *TNF*, tumor necrosis factor; *TNFR*, tumor necrosis factor receptor; *TRAF*, TNF receptor associated factors; *FADD*, Fas-associated protein with Death domain; *CRADD*, Death domain-containing protein CRADD; *EDARADD*, cctodysplasin-A receptor-associated adapter protein; *AP-1*, activator protein 1; *NLR*, NOD-like receptor; *SRCR*, scavenger receptor cysteine-rich repeat protein; *PGRP*, peptidoglycan recognition proteins; *GNBP*, Gram-negative binding protein; *CTLDC*, C-lectin domain containing protein; *FBGDC*, fibrinogen-domain-containing proteins; *C3*, complement C3; *C1qDC*, globular head C1q domain containing protein; *MBP*, mannose binding protein. * genes involved in both TLR and RIG-I pathway.

In order to assess the complexity of immune genes in the oyster genome, detailed annotation was carried out using homology-based searches as well as manual annotation based on immune gene sets that have been identified from various species. A total of 1,405 genes, belonging to 61 families, were identified (see Supplementary Table S1 online). Multiple examples of gene families that exhibit significant expansion as compared to *Drosophila melanogaster*, *Homo sapiens* (human) and other model genomes were identified (Fig. 1 and see Supplementary Table S1 online). The findings relating to the complexity and function of the gene sets will be described below.

### Transcriptome response to diverse pathogens

A comprehensive profile of the oyster transcriptome following pathogen exposure was generated; the challenge experiments resulted in 17 RNA-seq libraries (see Supplementary Table S2 online). Five libraries were used to evaluate the temporal dynamics of expression of oyster immune genes during biotic challenges, which included a mixture of four strains of Gram-negative bacteria (*Vibrio spp: anguillarum, tubiashii, aestuarianus*, *alginolyticus-1*) at time points: 0, 6, 12, 24 and 48 hours. The responses to challenge with five strains of Gram-negative bacteria (*V. anguillarum, V. tubiashii, V. aestuarianus*, *V. alginolyticus-1* and *V. alginolyticus-2*), one strain of Gram-positive bacteria (*Micrococcus luteus*), LPS and PBS (control) were characterized using a total of nine libraries; three libraries were used to characterize the response to the OsHV-1 virus infection.

**Figure 2:**
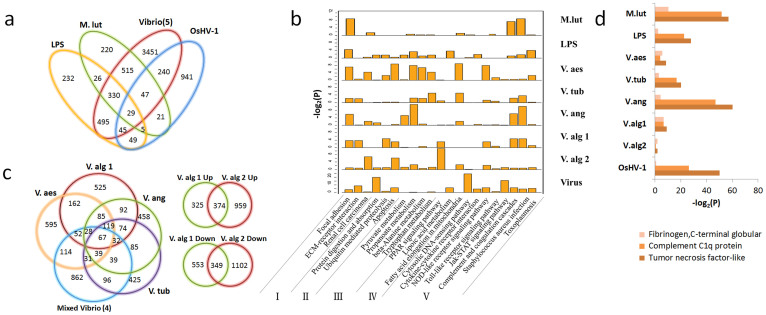
Infection response genes in the Pacific oyster, *Crassostrea gigas*. (a), Venn diagrams of differentially expressed genes under the challenge of LPS, Gram positive bacteria M. lut (*Micrococcus luteus*), Five types of *Vibrio* (*V. anguillarum, V. tubiashii, V. aestuarianus, V. alginolyticus-1, V. alginolyticus-2*), and OsHV-1 (ostreid herpesvirus). (b), Major pathways enriched for the up-regulated genes under seven types of pathogen and LPS challenges. I. Cell adhesion related; II. Cellular protein homeostasis; III. Amino acid metabolism; IV. Lipoic acid metabolism; V. Host immune defense. (c), (Left) Unique and differentially expressed genes infected with four types of *Vibrio* administered separately and simultaneously; (Right) Unique and differentially expressed genes infected with two strains of *V. alginolyticus*. (d), The three most enriched domain types of up-regulated transcripts generated by pairwise comparison of seven types of pathogens and LPS challenges vs PBS challenge. Three different colors represent three different enriched domains. The x axis is the – log 2 transformation of the *p*-value calculated in the enrichment test.

Overall, exposure to biotic challenges altered the expression of 8,866 genes. Over the course of the challenges with LPS, *M. luteus*, *Vibrio spp.* and a virus (OsHV-1), a total of 1,211, 1,193, 5,152 and 1,377 genes, respectively, were found to be differentially expressed, *P* < 0.001 (Fig. 2a). At a minimum, 232, 220, 3,451 and 941 genes are differentially expressed and specific to the LPS, *M. luteus*, *V. spp.* and viral challenges, respectively (Fig. 2a). For example, genes related to cytosolic DNA sensing or recognition and DNA replication only were up-regulated in the virus-infected transcriptome, whereas additional adhesion genes were up-regulated during bacterial challenge (Fig. 2b and see Supplementary Table S3). One explanation for the largest number of differentially expressed genes being associated with the *Vibrio*-induced group is that five different strains and multiple time-course samples were used. The large geneset may also include some genes involved in complex host-microbe interactions, i.e., symbiosis. In the *Vibrio* time-course infection, the total number of genes that are differentially expressed includes genes that are specific to certain, but not all, time points. The transcriptional responses to closely related bacteria can vary greatly (Fig. 2c). Notably, relatively few of the differentially expressed genes are shared among the four mixed and separate *Vibrio* infection transcriptomes (Fig. 2c).

Despite the considerable variation in the immune responses, the most prevalent or highly enriched protein domains of the up-regulated genes under each biotic stressor challenge include: the immune response-associated complement C1q protein, tumor necrosis factor-like and fibrinogen C-terminal globular domains (Fig. 2d). Activated pathways include those related to: cell adhesion, cellular protein homeostasis, amino acid metabolism, lipoic acid metabolism and host immune defense, including TLR and NLR pathways (Fig. 2b and see below).

Of 1,405 immune genes (see Supplementary Table S1), 1,362 (96.9%) are transcribed at RPKM (reads per kilobase per million) >1 and are presumed to represent functional genes (see Supplementary Fig. S1 online). Differential expression of 503 of the 1,362 immune genes was observed under at least one type of bacteria challenge. Up-regulation by only one type of bacteria or by LPS occurred in 145 immune genes. This observation may reflect a high level of functional divergence and (presumably) specificity, which in the case of *Vibrio* appears to be strain-specific.

### Expansion of TLRs is associated with functional diversity

**Figure 3:**
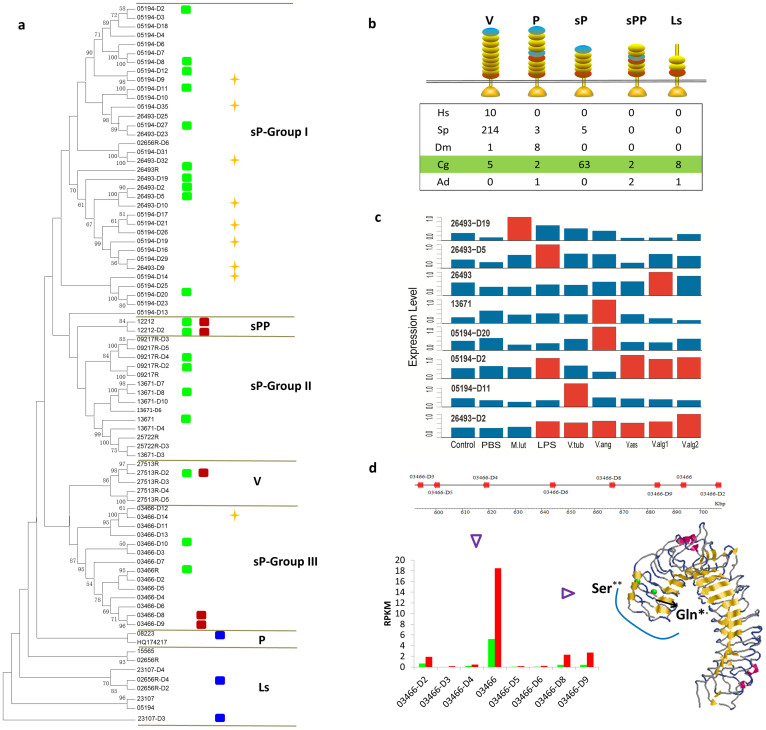
The duplication and functional divergence of Toll-like receptors in the oyster. (a), Phylogeny of the duplicated TLR family members in oyster and their expression divergence under biotic, abiotic challenge and during development. Unrooted neighbor-joining tree (left) were constructed based on TIR domains of TLRs in the oyster genome using MEGA5 (http://www.megasoftware.net/). The maximum likelihood method using PHYML^54^ also was used for confirmation of topology structure. TLRs can be classified into five divergent classes: sP (short protostome-like type), sPP (short protostome-like with protostome-like internal leucine-rich repeat C-terminal [LRRCT] leucine-rich repeat N-terminal [LRRNT] type), V (vertebrate-type), P (protostome-like type) and Ls (TLRs with only LRRCT domain). The oyster expanded sP-type can be subdivided further into three clades: group I, group II and group III. Rectangles indicate genes exhibiting upregulation under biotic (green) challenge, abiotic (red) challenge and specific expression at certain development stages (blue). Gold star denotes a pseudogene. (b), Comparison of Toll-like receptors (TLRs) with different domain architectures in: human (*Homo sapiens* - HS), sea urchin (*Strongylocentrotus purpuratus* - SP), fruit fly (*Drosophila melanogaster* - DM), Pacific oyster (*Crassostrea gigas* - CG) and staghorn coral (*Acropora* *digitifera* - AD). LRRCT in red, LRRNT in blue and LRR in yellow. Toll/interleukin-1 receptor (TIR) domains are shown as gold triangles. (c), Diverse expression of TLRs during challenge with five different *Vibrio spp.*, LPS (lipopolysaccharide), *Micrococcus luteus*, PBS (phosphate buffered saline) and a control (no treatment). Expression levels were normalized by considering the maximum expression levels for each gene. Red bar indicates that the expression levels are significantly different from the control and PBS; blue bar indicates no significant difference. (d), Divergent expression patterns and adaptive evolution of tandemly linked *TLRs* in scaffold 599 (top). (Left) Expression patterns of tandemly duplicated TLR genes in scaffold 599 during oyster herpes virus (OsHV-1) infection. Boxes indicate the expression levels (RPKM) of TLRs before (green [control]) and during (red) OsHV-1 infection. Structural modeling of ectodomain of TLR depicting sites (green dots) under positive selection. Only *TLR 03466R*, which encodes Ser^446^ and Gln^459^, was responsive to viral infection.

Pattern recognition molecules, which include TLRs, are an integral component of innate immune defense in all metazoans^4^. The oyster genome encodes an expanded set of 83 *TLR* genes, including nine with frameshift mutations (Fig. 3a and see Supplementary Table S1 online). Eighty TLR genes are predicted to contain the toll/interleukin-1 receptor (TIR) domain and on the basis of patterns of sequence relatedness can be divided into five groups: V (vertebrate-type), P (protostome-like with LRRCT [leucine-rich repeat C-terminal]-LRRNT [leucine-rich repeat N-terminal] ectodomains), sP (short protostome-like without LRRCT-LRRNT ectodomain), sPP (short protostome-like with LRRCT-LRRNT ectodomains) and Ls (LRRCT-specific ectodomains; Figs. 3a–b and see Supplementary Fig. S2 online). Five V- and two P-type TLRs were identified in oyster. The sP-TLRs, which have diverged into three subgroups, are the most extensively expanded (Fig. 3a). Of the nine TLRs of the sPP- and Ls-types, which do not exist in human, sea urchin or *Drosophila*, eight encode signal peptides and possess complete TIR domains, consistent with functional integrity. The greatly expanded short P-type TLRs in oyster along with equally extensive expanded P-type TLRs in *Drosophila* and V-type TLRs in the sea urchin suggest that species-specific TLR gene expansion is a frequent, independent occurrence in metazoan phylogeny. The clustering of a large number of oyster Ls- and P-type TLRs suggests that these genes are derived from oyster-specific expansions (see below) and may play a major role in host defense. The hypervariation in TLR genes seen in oyster as well as the presence of nine pseudogenes underscore the dynamic nature of this large gene family (Fig. 3a).

Of the 83 *TLR*s, 78 are expressed (RPKM > 1) (see Supplementary Table S1 online); 19 of the 78 are expressed differentially in the course of *Vibrio*, other bacterial and viral challenges (Fig. 3c). Both *Vibrio* and OsHV-1 are known pathogens of oyster. Expression of *TLR* Cg26493-D2 is elevated under challenge with both LPS and all *Vibrio* strains (Fig. 3c); however, the expression of some *TLRs* is induced only by a single or relatively few types of bacteria, e.g. *TLR*s Cg05194-D20 and Cg13671 are induced only by exposure to *V. anguillarum*; expression of *TLR* Cg26493-D19 is up-regulated only by challenge with *M. luteus*. Only *TLR 03466R* in scaffold 599 was up-regulated in response to an infection by OsHV-1 (Fig. 3d). Collectively, these results underscore the high degree of divergence of immune function within the tandemly duplicated, tightly linked *TLR* genes.

Examination of synonymous (*d_S_*) and nonsynonymous (*d_N_*) nucleotide substitutions among genes encoded in scaffold 599 indicates positive selection only in TLR 03466R at Ser/Lys^456^ (*P* < 0.05) and Gln/Gly^459^ (*P* < 0.1), which map to the surface of the third LRR and likely are critical to functional recognition (Fig. 3d). Three putative interferon regulatory factor-1 binding sites, which are important in antiviral response^16^, were identified in the promoter region of *TLR 03466R*, which is the highest number among the eight TLR tandem clusters in scaffold 599. Taken together, these results suggest that expansion of the TLR gene family coupled with selection in both the coding and promoter regions could result in both the diversification and specificity in pathogen recognition. Querying additional TLRs to determine responsiveness to other biotic challenges likely will shed light not only on the mechanisms of functional divergence but also on the relatedness of various polymorphisms that have been observed in individual members.

### Parallel expansion of immune adaptors

The immune adaptors that are coupled to TLR signaling via the TIR domain^4^ also have undergone considerable expansion in concert with the TLRs. Ten myeloid differentiation primary response 88 (MyD88)-like genes^17^ (Fig. 1) were identified; six possess a typical Death-TIR domain combination and four possess only a TIR domain (see Supplementary Fig. S3 online). This latter group is potentially significant as it could function as MyD88 competitors in interactions with TIR domains of TLRs. Homologs of the TIR domain-containing adapter molecules 1 and 2 are absent^18^; their functions likely have been assumed by other TIR-containing proteins. However, a greatly expanded set of additional genes containing the TIR domain (Fig. 1), which otherwise lacks homology to known adaptors, suggests a more complex and divergent system of signal transduction. The ancient Bilateria may have expanded the numbers of receptors as well as adaptors as an alternative means to expand and diversify immune function. Large numbers of adaptors likely were lost during the evolution of the immune system.

### Diverse innate immune mediators have undergone expansion and are differentially regulated in response to bacterial and viral challenge

RLRs are a family of cytoplasmic, antiviral sensors^19^. The oyster genome encodes 12 *RLRs*, equivalent to those seen in sea urchin (Fig. 1). However, at least seven RLRs in oyster possess vertebrate-type N-terminal caspase recruitment (CARD)-CARD structures, whereas none are present in sea urchin (see Supplementary Fig. S4 online). The presence of additional signal transduction molecules, including: mitochondrial antiviral-signaling protein, transmembrane protein 173 and interferon regulatory factor (Fig. 1), suggests that the RIG-I pathway functions in nonspecific antiviral responses.

Members of the tumor necrosis factor (*TNF*) and receptor (*TNFR*) superfamilies function in apoptosis, inflammation and tissue development (i.e., tissue remodeling)^20^. The number of genes encoding *TNF* and *TNFR* in the oyster genome is significantly higher than has been noted in other protostome invertebrates and is comparable to that seen in human and amphioxus (Fig. 1, see Supplementary Figs. S3 and S5 online). TNF signals typically are transduced through intermediate molecular TNF receptor-associated factors (TRAFs), which are coupled to the TLR, RIG-I and TNF pathways as well as to the apoptosis network^21^.

Four *TNF*, six *TNFR* and three *TRAF* members are expressed differentially during exposure to bacteria (*P* < 0.001), consistent with roles in immune and/or inflammatory processes that are downstream of TNF (see Supplementary Fig. S3 online). Three of the TNFs exhibit an early response pattern under time-course *Vibrio* infection, four exhibit late responses, one exhibits both early and late responses and three are down-regulated, underscoring a dynamic functional time course (see Supplementary Fig. S3 online).

**Figure 4:**
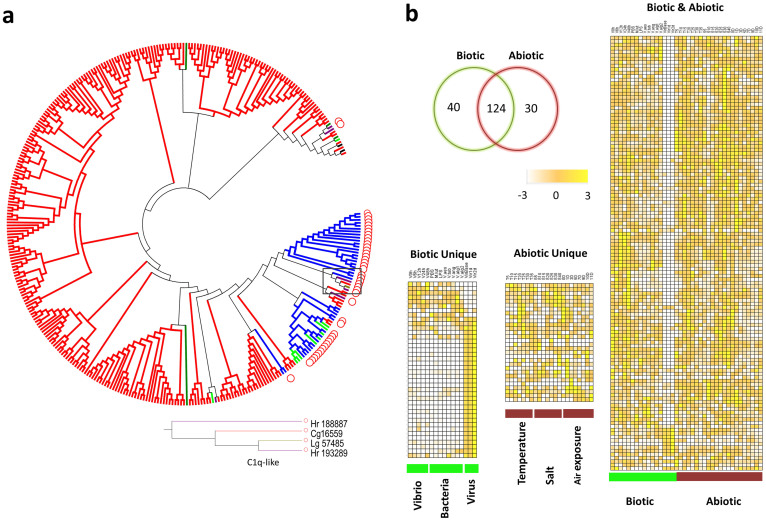
Expansion of C1qDC and divergent expression patterns under challenges and among different organs. (a), Phylogenetic tree (constructed with the maximum likelihood method) demonstrating lineage-specific expansion of C1qDC in Pacific oyster (*Crassostrea gigas* – red - Cg) and relationships to: owl limpet (*Lottia gigantea* – brown - Lg), leech (*Helobdella* *robusta* – purple - Hr), sea urchin (*Strongylocentrotus purpuratus* - green), amphioxus (*Branchiostoma floridae* - blue) and hydra (*Hydra magnipapillata* - black) C1qDC sequences. C1qDCs encoding an additional cysteine-rich domain (CRD) domain are noted with red circles. A single C1q-like gene with a collagen domain has been identified in oyster and its phylogenetic tree is shown (above). Scaled expression values are color-coded according to the legend on the top. (b), The number of differentially expressed *C1qDCs* associated with exposure to biotic and abiotic stressors. Biotic challenges include several species of *Vibrio* and other bacteria (see Supplementary Table S2 online); abiotic stressors are temperature, salinity and air exposure (see Supplementary Table S2 online). A number of immune genes function in abiotic stress. Heat maps from left to right are expression patterns of *C1qDC*: only under biotic challenges, only under abiotic challenges and those *C1qDCs* up-regulated by both biotic and abiotic challenges.

The complement system is defined by classical, lectin and alternative pathways and factors in the recognition, opsonization or lysis of various antigenic sources^22^. Members of the C1q family are integral elements of the classical complement pathway in vertebrates and effect antimicrobial as well as other functions^23^,^24^. The oyster genome encodes 321 C1q domain-containing (*C1qDC*) proteins, an order or two of magnitude greater number of molecules than is present in *Lottia gigantean* (5), *Capitella teleta* (23), *Helobdella robusta* (9) and *H. sapiens* (31) (Figs. 1 and 4a). However, only one oyster *C1q-like* gene possesses a typical vertebrate-type collagen domain. By contrast, no clear C1q-like homologs are seen in *Drosophila* or in the sea anemone, a group that diverged prior to the protostomes (Fig. 1). Genes encoding the typical domain structure of C3, as well as factor B-like molecules, CD109, four alpha-2 macroglobulins and three additional thioester-containing proteins also are present in oyster. Neither C4, C5 nor C6 was identified, consistent with their emergence from a C3-like ancestor in chordates. Gene structures related to those molecules encoding members of the alternative complement pathway also are present, although their physiological functions are not yet understood. Collectively, these findings suggest that a rudimentary complement system in the oyster may function through a set of expanded and diversified genes.

One hundred sixty-four of the *C1qDC* genes (51.1%) are differentially expressed following bacterial challenge (Fig. 4b), consistent with a direct role in pathogen recognition or as part of a complement system. A variety of expression patterns at different time points after challenge with *Vibrio* underscores the functional divergence of C1qDC (Fig. 4b).

Large numbers of both fibrinogen domain-containing proteins (see Supplementary Fig. S4 online), representing lectins that serve essential roles in vertebrate coagulation pathways^25^ and C-lectin domain containing proteins, which have been shown to function in invertebrate defense^26^, have been identified (Fig. 1 and see Supplementary Table S1 online). Additional genes involved in the recognition or clearance of bacteria, include: *NLR*s, peptidoglycan recognition proteins^27^, β-1,3-glucan recognition proteins, superoxide dismutases^28^, catalases^29^, *defensins*, *big defensins*, bactericidal permeability-increasing proteins^30^ and membrane attack complex and perforin domain-containing proteins^31^.

### Tandem duplication and lineage-specific expansion

The expansions in the multigene families encoding innate immune-type molecules can be attributed to multiple local tandem duplication events. Of the 83 *TLR*s, 57 are linked in tandem arrays; scaffold 599, encodes eight TLRs in relatively close linkage (Fig. 3d and see Supplementary Fig. S2 online). TNF (see Supplementary Fig. S5 online) and MyD88-like genes are either arranged in clusters of three or in pairs (see Supplementary Fig. S3 online). Six of the 11 putative *RIG-1* genes and four of the 15 *TRAF*s are in pairs. Bactericidal permeability-increasing proteins and many *C1qDC*s are in clusters.

In order to examine the divergence of immune gene families as well as their expansion, phylogenetic analyses based on 116 TLR-specific TIR domains were carried out. Comparisons of the TIR domains with those associated with TLRs in two other molluscan species, *Lottia gigantea* (owl limpet) and *Mytilus galloprovincialis* (Mediterranean mussel; see Supplementary Fig. S2 online) suggest that V-type TLRs are the most conserved. Compared to the TLRs in owl limpet (Gastropoda), the sP-I, II and III TLRs are expanded in mussel and oyster (Bivalvia). Eight of the 14 sP-group I type TLRs in oyster are in tandem linkage indicating that the sP-group I type TLRs may have undergone tandem duplication after the divergence of mussel and oyster. Only MgTLR-i belonging to the sPP-type group is inducible in mussel following bacterial and fungal challenges^32^; both sPP-type TLRs are up-regulated under biotic and abiotic stress conditions in oyster (see below). Such high intra-taxon homology that is evident in phylogenetic analyses of several other gene families, including: MyD88 (see Supplementary Fig. S3 online), TNFs (see Supplementary Fig. S3 online), TRAFs (see Supplementary Fig. S3 online), C1qDCs (Fig. 4a) and RIG-Is (see Supplementary Fig. S4 online), is consistent with lineage-specific expansion of these genes.

Scaffold 599, which encodes eight TLR genes, is informative. Distinctive distributions of putative promoter motifs are shown (see Supplementary Fig. S2). Differences in functional responses also are evident (see below). The particularly sophisticated innate immune system present in the extant oyster and likely other invertebrates most likely evolved through repeated duplications and selection driven divergence^33^,^34^,^35^.

### Expression of oyster immune genes is linked to abiotic stress and development

**Figure 5:**
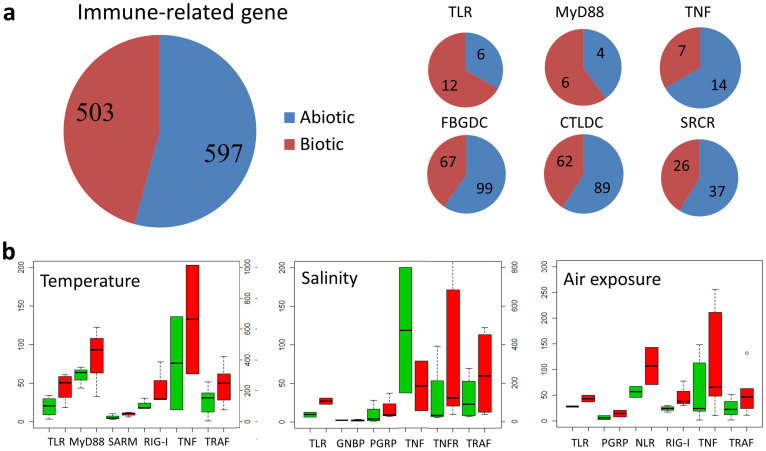
Expression differences of representative immune-related gene families under biotic and abiotic stress. (a), Comparison of the numbers of differentially expressed genes under abiotic and biotic challenges indicating that a set of immune genes function in abiotic stresses (see Supplementary Table S3 online). (b), Expression patterns of immune-related genes during different abiotic challenges. The boxes indicate the expression levels (RPKM) of representative immune genes under normal (green) and stress (red) conditions.

**Figure 6:**
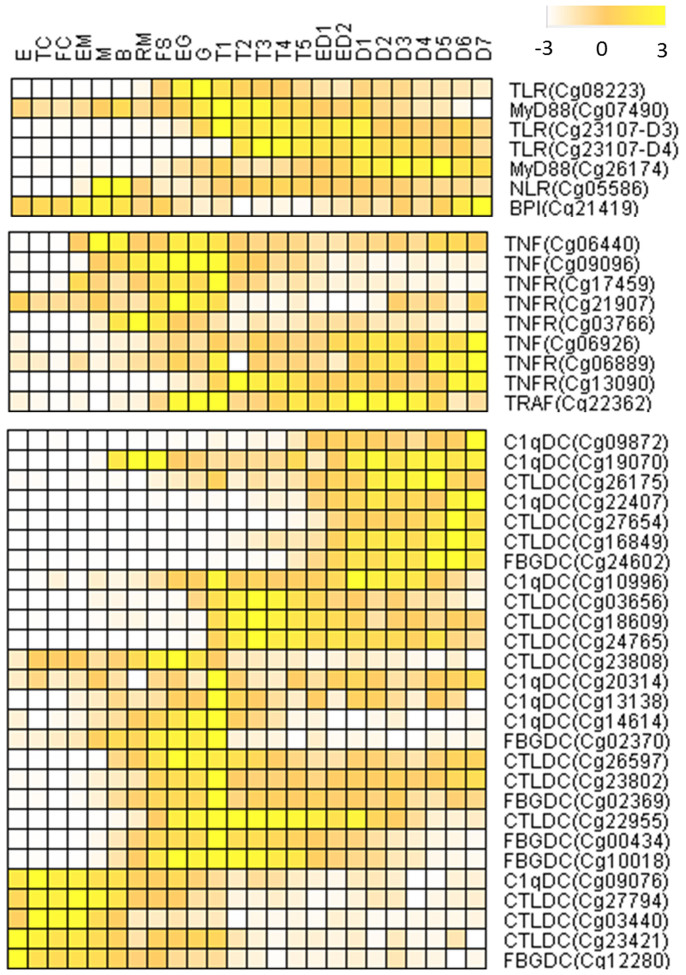
Expression of immune genes during development. Heat map depicting the specific expression of immune-related genes at different developmental stages: E, egg; TC, two cells; FC, four cells; EM, early morula; M, morula; B, blastula; RM, rotary movement; FS, free swimming; EG, early gastrula stage; G, gastrula; T1, trochophore 1; T2, trochophore 2; T3, trochophore 3; T4, trochophore 4; T5, trochophore 5; ED1, early D-larva 1; ED2, early D-larva 2; D1, D-larva 1; D2, D-larva 2; D3, D-larva 3; D4, D-larva 4; D5, D-larva 5; D6, D-larva 6; D7, D-larva 7.

Additional transcriptome data were queried to characterize the expression of immune genes at different developmental stages, in different organs, as well as under different conditions of abiotic stress (see Supplementary Table S2 online)^14^. Gene ontology terms related to ‘defense response’, ‘innate immune response’, ‘response to biotic stimulus’, ‘receptor binding’ and ‘tumor necrosis factor receptor binding’ are enriched in the abiotic response gene sets^14^. Of the immune genes affected, 54.2% were differentially expressed under abiotic stress involving changes in temperature, salinity and air exposure compared with 45.7% under biotic stress (Figs. 5a–b), suggesting a majority of the “immune genes” examined were responsive to abiotic stress. Among the immune genes that were up-regulated under abiotic stress, the most prevalent or highly enriched protein domains include those encoding: complement C1q, tumor necrosis factor-like and c-type lectin proteins^36^,^37^, suggesting significant functional divergence as well as cross-talk between the immune and stress responses. Mechanistically, not only do abiotic and biotic “response” genes belong to the same multigene families but they likely share pathways of intracellular signaling. About 25.6% of immune-related genes exhibit organ-specific expression (see Supplementary Fig. S1 online), which may reflect spatial diversification and functional compartmentalization. More than 5.6% of immune-related genes were shown to be expressed at discrete stages in early development (Fig. 6).

Five TLRs were up-regulated two- to six-fold under abiotic stress (Fig. 5b); two of these genes were not differentially expressed under biotic challenges, suggesting specific role(s) in abiotic responses. Expression of three TLRs also was up-regulated during gastrula and early trochophore stages of development (Fig. 6), indicating a a developmental regulatory role in the oyster. TLRs also demonstrate tissue-specific expression patterns including: six in hemocytes, six in labial palp and one in adductor muscle (see Supplementary Fig. S1). The V-type TLR (Cg27513R-D2), which is related to vertebrate-type TLRs, is responsive to both biotic and abiotic challenges, respectively, as are the sPP types 12212 and 12212-D2 (Fig. 3a). Of particular note, the three TLRs (3.6%) that are expressed in development are P- or Ls-types (Fig. 3a), which are closest in sequence to the putative ancestral form of TLRs (Fig. 3b)^38^.

Irrespective of the specific function, co-option of duplicated and diverged genes for more specialized roles appear to have occurred on an unanticipated scale, extending to other gene families. Patterns of functional diversification resembling those seen with TLRs also are seen with the 10 *MyD88-like* genes, of which four are expressed constitutively, four are expressed differentially in response to biotic and abiotic challenges and two respond only to biotic challenges (see Supplementary Fig. S3 online). Of the two *MyD88* genes that are present in tandem in the same scaffold, Cg26099 is highly responsive to both biotic and abiotic stresses whereas Cg26092, which clusters with orthologs from other species is not, again underscoring possible functional specialization even in recently duplicated paralogs (see Supplementary Fig. S3 online). Of the four *MyD88* genes that are expressed constitutively during challenges, Cg07490 is expressed during early trochophore stages and Cg26174 during early D-shape larvae stages (Fig. 6), consistent with important role(s) in larval development.

The TNF gene family also varies in response to abiotic stress, e.g. high temperature and air exposure with induced up-regulation of their transcription (Fig. 5b). More than half of TNFs were responsive to biotic challenge; whereas, 39% of TNFs were highly responsive to air exposure (Fig. 5b). The seven TNFs that responded to air exposure did so in different manners: four were up-regulated initially followed by rapid decreases and three others were down-regulated throughout the experimental course. One of the 13 TNFRs that possesses and five TNFRs that lack a death domain are expressed differentially during *Vibrio* exposure. Five TRAFs are up-regulated under temperature, four under salinity and six under air exposure stress (Fig. 5b), suggesting a general role in abiotic stress defense. Three of the four TRAF6-like genes are up-regulated during abiotic challenge; one is up-regulated by both biotic and abiotic stress. These findings, along with lineage-specific expansion of TRAF6, indicate that duplication of TRAF3 might be linked to protection during abiotic stress. Two TNFs and three TRAFs are expressed during early development, whereas one TNF, two TNFRs and one TRAF are expressed specifically at D-shape larval stages, suggesting function in development (Fig. 6).

C1qDC is the largest and most extensively diversified of the gene families encoding immune-type molecules; 79.4% of C1qDCs are expressed differentially under abiotic challenge (Fig. 4b). Of the genes that are responsive to biotic stress, 75% also are differentially expressed directly under abiotic stress, the highest percentage of any of the gene families reported. Compared to C1qDCs responses to both biotic and abiotic stress, the C1qDCs that are responsive only to biotic stress exhibit distinct or specific expression patterns under bacteria or virus challenges. Those C1qDCs that are responsive to biotic stress are highly expressed and likely function specifically in the digestive gland, which is an important first-line defense organ against pathogens (Supplementary Fig. S6 online). Some C1qDC genes are expressed during discrete stages of development (Supplementary Fig. S6 online), underscoring the wide range of functional variation among this particularly large, diversified family of genes. Other gene families exhibit significant up-regulation during development only, e.g. expression of CgNLR-2 increases up to 16.7-fold during the transition between early morula and gastrula stages (Fig. 6), but is not influenced by abiotic and biotic challenge. The expression of RIG-1 genes is up-regulated 2-fold during temperature and air exposure challenges (Fig. 5b); however, no specific expression pattern is evident for these genes during gastrula and early trochophore stages (Fig. 6).

Although the patterns of transcription differ markedly among the various genes surveyed, the differential response to abiotic vs. biotic challenge and the variation in transcription during development versus that observed in different tissues are a general character of the diversified families of innate immune genes. The actual numbers of genes that exhibit functional variation differ markedly by multigene families.

### Evolutionary perspectives

**Figure 7:**
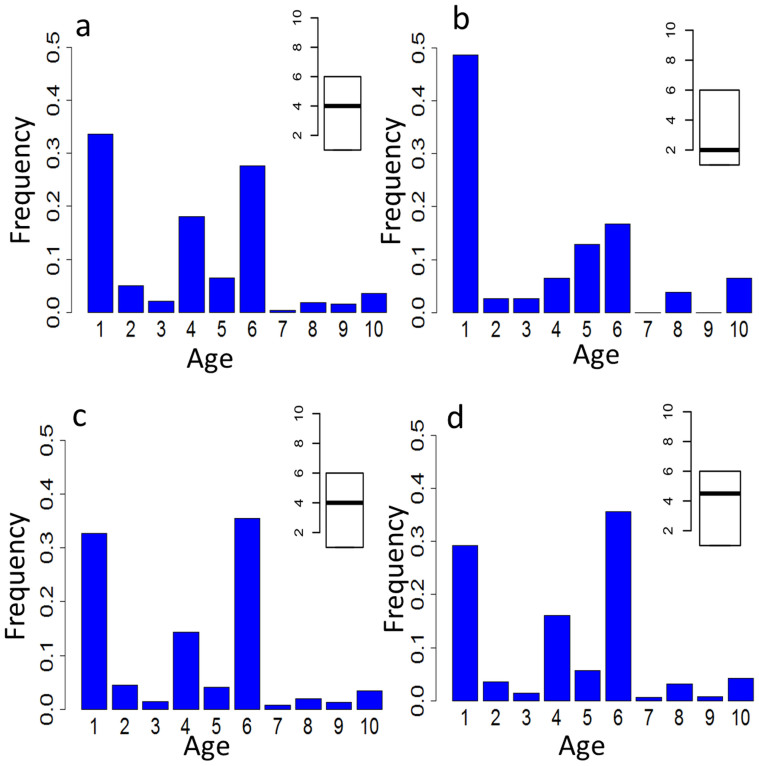
Phylostratigraphic ages of different functional groups of genes. Phylostratigraphic analyses classify genes into 10 levels, ranging from cellular organisms (age 1) to Bivalvia (age 10)^39^. The highest phylostratigraphic values corresponding to the youngest genes specific to Bivalvia. Other ages represented as: Eukaryota (2), Opisthokonts (3), Metazoa (4), Eumetazoa (5), Bilateria (6), Protostomia (7), Lophotrochozoa (8) and Mollusca (9). Frequencies of genes with different phylostratigraphic ages, including: (a), all immune-related genes; (b), specifically expressed during early development; (c), responsive to abiotic stress (temperature, salinity and air exposure) and (d), responsive to biotic (bacteria and LPS) challenges. The insert boxplots depict the mean and range of gene ages for the corresponding gene sets.

Phylostratic analyses were conducted in order to further understand the divergence of these highly complex gene families. This approach is based on estimates of sequence similarity from BLAST analyses of different genomes and traces evolutionary innovations and times of divergence of genes in terms of their relative ages^39^. Immune-type genes that are expressed specifically during early development exhibit lower phylostrata values (more ancient in origin) than do those that function in conventional immunity or abiotic stress responses (Figs. 7a–d). It follows that developmental genes or genes with dual functions may have emerged early in evolution, and further duplication and diversification within some of these gene families might give rise to genes that were recruited to function in abiotic stress and responses to immune challenge. If the tightly linked TLR genes that have been identified in scaffold 599 are any indication, functional divergence and adaptive recruitment of members of the expanded families of innate immune genes may have as much to do with the type, numbering and positioning of promoter elements flanking the genes as with the variation in coding sequence and association of genes with particular signaling pathways. The relative times of divergence of the biotic and abiotic stress response of genes are less clear and potentially could have been simultaneous. Furthermore, the basis for this general effect may lie not only in the primary function of a specific molecule, e.g. receptor specificity, but also could relate to the capacity of that molecule to form and/or mobilize a preexisting signaling network. Whereas the molluscs have a very long evolutionary history, the divergence of functions relating to development and abiotic/biotic responses within the members of the multigene families at issue likely arose early in evolution (probably during the emergence of metazoans), independently and well in advance of the extensive lineage-specific expansion of immune genes described here.

## Discussion

This study represents a comprehensive genomic and transcriptomic survey of innate immune molecules in the Pacific oyster, which have undergone adaptive evolution through tandem gene duplication and lineage-specific diversification that are an order of magnitude larger and more complex than seen in vertebrates. Seventeen transcriptome data sets from oysters challenged with different types of microorganisms have been subjected to in depth analysis and differential regulation of individual immune genes has been observed when animals are challenged with pathogenic bacteria and virus as well as in the course of environmentally relevant abiotic stress. Several observations indicate that immune genes in the oyster are enriched relative to other genes: Gene Ontology enrichment studies suggest that “immune-related” terms are enriched in abiotic stress^14^, immune related domains are enriched relative to other genes^14^, overall gene expression patterns exhibit differential expression of immune genes over non-immune genes in biotic challenge and immune genes that exhibit responses in abiotic stress not only belong to the same multigene families but share intracellular signaling pathways. The results herein provide evidence for the functional integration and specific roles of individual members of multiple families of innate immune genes in response to: bacteria, a bacterial product, a pathogenic virus and environmental stress as well as during normal development.

Multiple microbial encounters exert a powerful selective pressure on organisms and drive immune system co-option and integration of available cellular mechanisms to perform diverse immune functions. The defense machinery has experienced many rounds of genetic novelty and adaptive evolution. Highly specialized, systemic cells and processes are usually considered an exclusive property of vertebrate adaptive immunity. It is likely that alternative mechanisms of immune function and specificity have evolved in many invertebrate species. Expansion of multiple gene families encoding innate immune molecules also have been described in sea urchin^8^ and amphioxus^9^. Both of these species are invertebrate deuterostomes, one of the two branches of evolution of the bilaterian animals that include all vertebrates and some invertebrates. The oyster, a protostome representing the other branch of bilaterian evolution, possesses vastly expanded innate immune repertoires, the evolution of which likely is driven by both biotic and abiotic stress.

Gene duplication and expansion are important sources of evolutionary novelty as selection maintains duplicated genes only through functional divergence^40^,^41^. Recent work suggests that some members of multigene families are prone to expansion via various mechanisms such as tandem duplication^42^. MHC and Ig genes in vertebrates^43^, TLRs in sea urchin^8^, TNFs in amphioxus^9^ and FREPs in fresh-water snail^44^ have been shown to exist in tandem clusters. Local tandem duplication could be a major mechanism of immune gene expansion in oyster, in which a large proportion of the expanded innate repertoire exists as tandem gene clusters. The numbers of duplicated genes found in tandem linkage in oyster likely represent an underestimate owing to the relatively short scaffold N50 length of the oyster assembly (401 Kbp). There also is a tendency for minimally diverged paralogs to be lost in genome assembly^45^.

The studies presented here provide preliminary evidence for highly specific functional responses to biotic challenge by specific members of large multigene families encoding innate immune-type molecules. The extensive genetic and functional diversity of large families of innate immune genes is in marked contrast to our general notions of innate immunity in both higher vertebrates and other invertebrates, including another deuterostome, *Ciona* *intestinalis*^46^,^47^. Gene duplication and subsequent diversification of expanded members of gene families driven by selection is a recognized mechanism of genome innovation and adaptation^48^. The expansion and functional divergence of immune gene families in the oyster, as indicated by their expression profiles, suggest that strong selective pressures are being maintained by adaptive forces that are shaped both by its pathogen-rich and dynamically changing intertidal and estuarine environment, which create complex biotic and abiotic stress that also affect bacteria and virus directly. This is consistent with the finding in the fresh-water snail, *Biomphalaria glabrata*, that large expansions of FREPs might be due to lineage-specific selective pressure from (trematode) pathogens^44^ with individual members of large gene families participating in specific responses^15^. Selection for stress adaptation or immune protection, which is mediated by closely related members of the same diversified multigene families, may act on the same duplication events, exert powerful selective pressure on organisms and likely have driven immune system co-option and the integration of available cellular mechanisms to perform diverse defense functions.

Our overall understanding of immune phylogeny has been expanded by the comprehensive characterization of the patterns of expression of immune genes in different tissues and at different development stages as well as functional responses to experimentally imposed abiotic and biotic conditions. For example, in the expanded TLR gene family, divergence appears to have proceeded through adaptive evolution along three major functional lines: bacteria/virus-responsive innate immunity, responsiveness to multiple forms of abiotic challenge (and immunity) and developmental ontogeny. A similar general pattern of functional divergence is seen in *Drosophila* Toll^38^; however, where the analogy to *Drosophila* (a protostome) departs is the exceptionally large numbers of innate immune genes that have been identified in oyster, sea urchin and amphioxus. Although the developmental stage-specific expression of several of the innate immune genes is evident, discerning their function is challenging. These genes may be serving stage-specific functions relating to immunity and/or stress in the externally developing larva or lack a direct role in immunity.

The vastly expanded and multivariant nature of immune genes, the complex infection response of this species and the inherent rapid rate of expression divergence within multiple extensively diversified multigene families underscore the merits of the oyster as a model for further investigation of the evolution of the immune system. It will be of major interest to further define the signaling and regulatory networks that mediate key physiological responses in this species in order to gain a detailed understanding of how specialized (diverged) molecules interface with integral elements of the development and host-defense pathways and processes. It is highly likely that a far more complete understanding of the evolution and functional integration of multigene families in a broad range of host responses and defenses will emerge from further investigations of the innate immune recognition systems found in invertebrate species.

## Methods

### Gene annotation

Computational scanning to identify putative immune-related genes within the oyster genome involved building the first set from InterProScan annotation. Conserved domains from the immune genes were collected and scanned in the predicted protein database of the Pacific oyster. The second set was derived from homology searches of immune genes from other model species including: fruit fly, sea urchin and humans with BLASTP. The third set was derived from BLAST annotations between all of the predicted proteins and scanning the NCBI non-redundancy protein database to complement the BLASTP homology searches. The fourth set of immune genes was chosen from TBLASTN searches against the Pacific oyster genome sequences. Genewise (http://www.ebi.ac.uk/Tools/Wise2/index.html) analysis was applied for genes that were not predicted as models in the whole genome. To correct against the inadvertent inclusion of both haplotypes in genome assembly, which would artificially inflate estimations of copy number variants of gene families, 66.6 Gbp whole genome shotgun data with 105-fold coverage of the genome (SRA040229) were aligned onto the oyster genome assembly using Burrows-Wheeler Aligner (BWA)^49^ and the predicted genes with average depth of less than 80-fold were deleted^14^. Extensive manual correction of the putative oyster immune-related genes was conducted to eliminate possible errors.

For brevity, only the last five digits of the *C. gigas* gene names are used. GenBank accession numbers for all genes can be obtained by adding the prefix CGI_100 to the five-digit gene codes. For example, the accession number for 26099 or Cg26099 is CGI_10026099. Any suffix to the gene code represents a manually annotated gene.

### Phylogenetic analysis

Oyster gene sets were compared with those of sea anemone, sea urchin, fruit fly and human, which were downloaded from JGI, SpBase, Flybase and ENSEMBL, respectively. Chi-square testing was used to determine whether or not the Pacific oyster had significantly more genes in each selected family than were found in the other species.

Multiple sequence alignments were performed using MUSCLE^50^ with default parameters and the resulting alignments were refined with trimAl^51^. Phylogenetic trees were constructed by neighbor-joining (NJ) or maximum likelihood (ML) analytical approaches. PROTEST was used to select the substitution model^52^. MEGA^53^ and PHYML^54^ were used to find the NJ and ML trees, respectively. The robustness of the inferred trees was assessed using bootstrapping, 1000 in the NJ tree and 500 in the ML tree.

PAML version 4^55^ was used for comparing the rate per site of *d_N_* to the rate per site of *d_S_*. The eight TLRs located to scaffold 599 were used in this analysis. Two alternative models were implemented: M8a is the null hypothesis that allows all sites to evolve neutrally and M8 is the alternative hypothesis that lets some sites evolve under positive selection. M8 and M7 were compared using a likelihood-ratio test (LRT). In order to ensure convergence, the M1a and M2a models also were used. SWISS-MODEL (http://swissmodel.expasy.org/) was used to locate and visualize the positively selected sites.

### Bacterial strains, oyster and immune challenge

*V. anguillarum, V. tubiashii, V. aestuarianus*, *V. alginolyticus-1* and *V. alginolyticus-2* were cultured in trypticase soy broth (TSB marine broth) at 28°C. *M. luteus* was cultured in Luria–Bertani (LB) medium at 37°C. Pacific oysters averaging 10 cm in shell height were purchased in Qingdao, China and acclimatized in tanks with 25 ± 0.5°C, pH 8.0 ± 0.3 seawater and an 8:12 light:dark photo period. A sand-saw was used to cut a small hole on the edge of the oyster to accommodate injection^56^. Oysters were acclimated for two days prior to the onset of experimentation.

Oysters were challenged by injecting 100 μL of an equal mixture of four pathogenic *Vibrio* species (*V. anguillarum, V. tubiashii, V. aestuarianus, V. alginolyticus*) into the adductor muscle. Gill tissue from each oyster was collected at: 0, 6, 12, 24 and 48 h after injection and frozen immediately at −80°C. Each treatment included three replicates. In the other experiments, oysters were challenged with 100 μL of five *Vibrio* strains(*V. anguillarum*, *V. tubiashii*, *V. aestuarianus*, *V. alginolyticus-1*, *V. alginolyticus-2*, *M. luteus*), LPS (Sigma-Aldrich, USA) or sterile PBS (control sample) (see Supplementary Table S2 online). All the *Vibrio* strains were grown separately, overnight at 28°C in TSB marine broth; *M. luteus* was grown overnight at 37°C in LB broth. Oysters with only small holes on the edge also were used as control samples. The gill of each oyster was collected at 12 h after injection and frozen immediately at −80°C. Each treatment had three replicates. RNA was extracted from each sample using the guanidinium thiocyanate-phenol-chloroform extraction protocol (Trizol, Invitrogen) and pooled in equal-molar amounts from the three replicates. Although pooling of triplicate samples is not ideal, several points merit consideration: 1) independent qPCR experiments of multiple immune genes (including TLRs and two MyD88 genes in the response to virus) with replicate samples has shown that differences observed in the pooled samples are reproduced (data not shown), 2) the same effects are observed when experiments are repeated with different populations of animals, in some cases pools of as many as 46 animals have been used, and 3) effects on the same or related differentially expressed genes are observed in dosage effects or time series challenges.

For the virus infection experiment, sexually mature female and male oysters were collected, dissected and fertilized in Qingdao, China. Larvae were collected at umbo stage^14^ (see Supplementary Table S2 online). OsHV-1 was detected using a nested PCR primer designed based on OsHV-1 genome^57^,^58^. RNA-seq reads were mapped to the OsHV-1 genome to confirm virus replication in the infected oysters (see Supplementary Fig. S4 online).

### Library preparation and RNA sequencing

Poly-A RNA was isolated with oligo-dT-coupled beads from 20 μg total RNA of each sample and used for first strand cDNA synthesis with random hexamers and Superscript II reverse transcriptase (Invitrogen). Second strand cDNA was synthesized by DNA polymerase I (Invitrogen) and the double-stranded cDNA was end-repaired. A 3′ dA overhang was added and the double-strand product was ligated with Illumina adapters. The adapter-ligated sample was size-selected to ~200 bp fragments by electrophoresis. After 15 PCR cycles, libraries were sequenced using an Illumina GAIIx sequencer. Transcriptomic data that were generated in earlier studies (Gene Expression Omnibus accession number GSE31012^56^) also were analyzed. These data include Illumina RNA sequencing reads from: seven organs, 38 developmental stages and under various abiotic challenges, including extreme temperature, salinity and air exposure. Acquisition and analysis of RNA sequencing data are as described in other parts of this study and follow that described previously^14^.

### Expression calculation and differential expressed genes

Tophat was used to align sequence reads to the genome^59^. Gene expression levels for transcripts, exons and introns were measured by RPKM and normalized as suggested^60^. Two methods were used to identify the *Vibrio*, bacterial and viral induced gene set. Differentially expressed genes and gene orthology enrichments were determined using established methods^61^. Heatmaps were generated using cluster 3.0 (http://bonsai.hgc.jp/~mdehoon/software/cluster/software.htm).

### Immune genes in oyster-specific phylostrata

Phylostratigraphic ages for oyster genes were defined on the basis of the current understanding of phylogenetic relationships (see Supplementary Table S1 online). Several databases or websites were considered, including: NCBI Taxonomy database (http://www.ncbi.nlm.nih.gov/taxonomy), TimeTree (http://www.timetree.org/)^62^, Tree of Life (http://tolweb.org/tree/), Wikipedia (http://en.wikipedia.org/) and the latest genome papers^14^,^63^. Genome information was considered to be the most reliable for such comparisons. The organisms selected for every phylostrata are listed (see Supplementary Table S1 online). The Nr database was subjected to BLASTP analysis using all oyster-predicted protein sequences. For the organisms not included in Nr database, their genome protein sequences also were subjected to BLASTP analysis. The BLASTP E-value cut-off was set to 1E-3. The genes from level 10 are defined as bivalvia lineage-specific (see Supplementary Fig. S5 online).

## Author Contributions

L.Z. and G.Z. designed research, L.Z. performed research, L.L., G.Z., L.Z., X.G., G.W.L. and L.J.D. analyzed data; and L.Z., G.W.L., X.G., L.J.D., L.L. and G.Z. wrote the paper.

## Additional information

**Accession codes**: Raw sequencing data of the transcriptomes have been deposited into the Short Read Archive (SRA) (http://www.ncbi.nlm.nih.gov/sra) under the accession number SRP019967.

## Supplementary Material

Supplementary Information Supplementary Information
