# Supplementary material for: Massive expansion and functional divergence of innate immune genes in a protostome
Source: Sci Rep. 2015 Mar 3;5:8693. doi: 10.1038/srep08693 (PMC4346834; doi:10.1038/srep08693)
Supplement: Supplementary Information [file srep08693-s1.doc]

**Massive expansion and functional divergence of innate immune genes in a protostome**

Linlin Zhang^a^, Li Li^a^, Ximing Guo^b*^, Gary W. Litman^c,d,*^, Larry J. Dishaw^c^, Guofan Zhang^a*^

^a^Institute of Oceanology, Chinese Academy of Sciences, Qingdao 266071, China.

^b^Haskin Shellfish Research Laboratory, Institute of Marine and Coastal Sciences, Rutgers University, Port Norris, NJ 08349, USA.

^c^Morsani College of Medicine, Department of Pediatrics, University of South Florida, St. Petersburg, FL 33701, USA.

^d^All Children’s Hospital Johns Hopkins Medicine, St. Petersburg, FL 33701, USA.

The authors declare no conflict of interest.

*corresponding authors: G.W.L. (glitman1@jhmi.edu), X.G. (xguo@hsrl.rutgers.edu), G.Z. (gzhang@qdio.ac.cn).

**
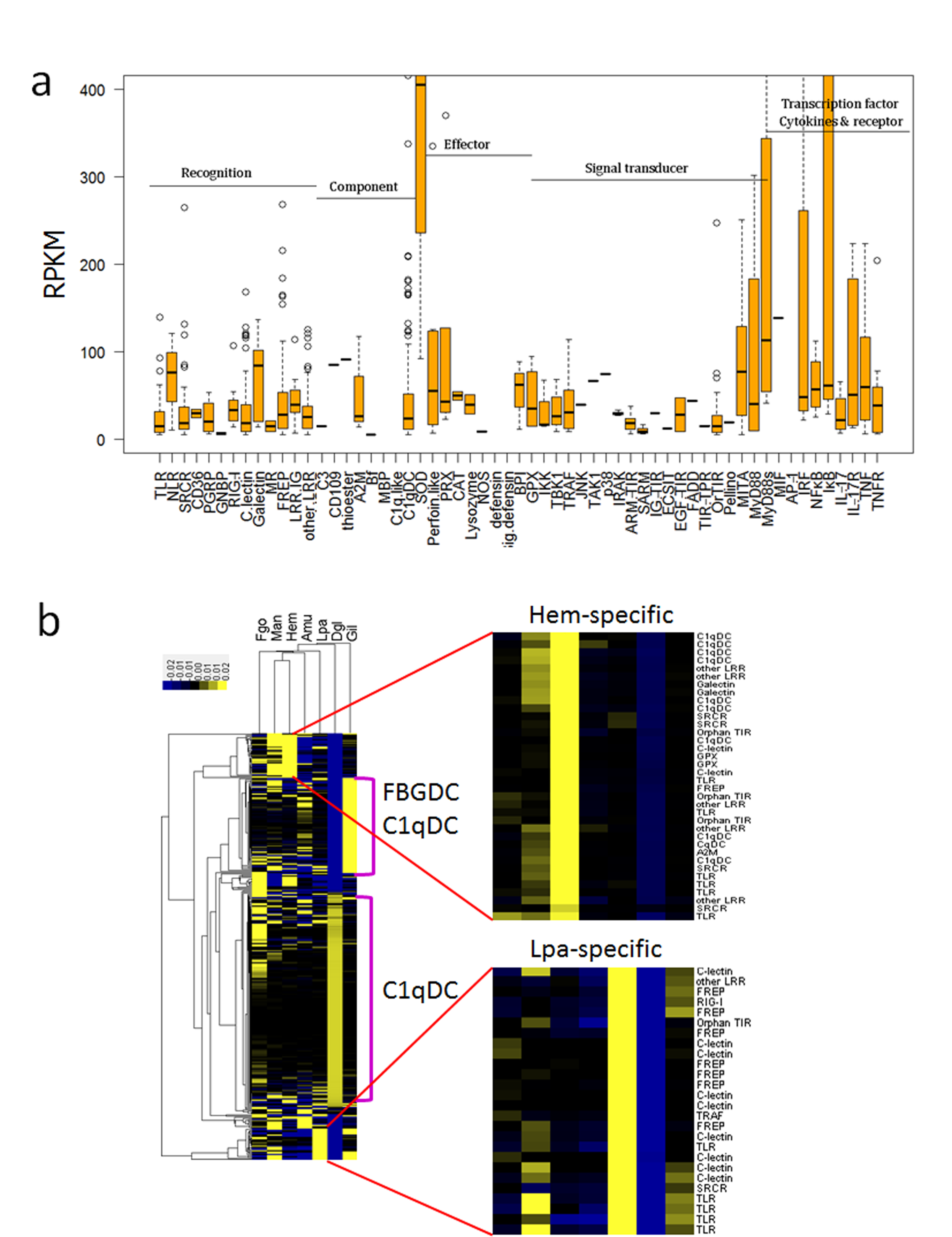
**

**Supplementary Figure 1 | a Maximum expression levels of immune gene families associated with all 14 biotic challenge transcriptomes (Supplementary Table 2)**.

Different immune genes are indicated on the X axis. Functional classes are indicated directly. Genes with RPKM <5 are not included. Gene family abbreviations are defined in Supplementary Table 1. Genes encoding receptors, adaptors and transcription factors: TLR, MyD88, TNF, TRAF, PGRP, βGRP and AMP, are highly up-regulated by biotic challenges. **b** **Heat map of 359 genes that are specifically expressed in different tissues of the Pacific oyster.** Tissues are: female gonad (Fgo), mantle (Man), hemocyte (Hem), adductor muscle (Amu), labial palp (Lpa), digestive gland (Dgl) and gill (Gil). Thirteen TLRs, including six in hemocytes and six in labial palps, exhibit differential expression. Genes encoding C1qDC and FBGDC are expressed specifically in gill or digestive gland.


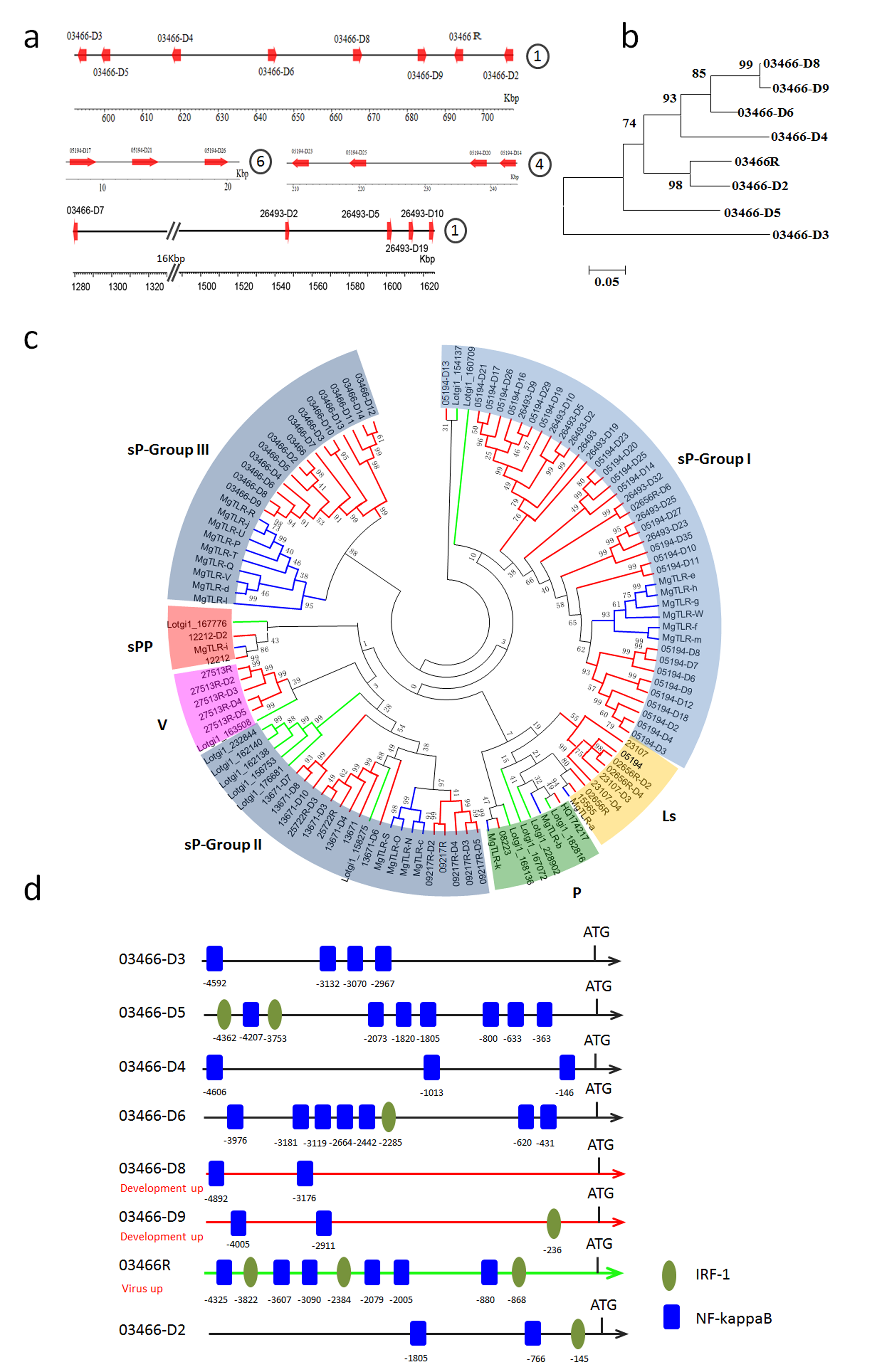


**Supplementary Figure 2| Phylogenetic analysis of *TLR* genes and their diverse promoters.**

**a** Clustering of representative *TLR* genes (red arrows) in the Pacific oyster (*Crassostrea gigas*). Numbers in circles represent the number of times that the specific gene set is duplicated. The top cluster encodes eight linked *TLRs* and is designated scaffold 599. Bottom scale bar represents relative positions. **b** Order of tandemly duplicated TLR genes in scaffold 599 is reflected in the phylogenetic tree. Trees were constructed with 1,000 bootstrap trials with MEGA program using the neighbor-joining method. The scale bar indicates a branch length of 0.05. **c** Phylogenetic analysis is based on 116 TIR domains of *TLRs*. The predicted amino acid sequences are from: Pacific oyster (red), owl limpet (*Lottia gigantea* - green), Mediterranean mussel (*Mytilus galloprovincialis* - blue). Tree is based on the maximum likelihood method. **d** The diverse putative promoters of eight linked *TLRs* that map to scaffold 599. Immune-related regulatory elements IRF-1 and NF-kappaB were predicted by AliBaba 2.1 (http://www.gene-regulation.com/pub/programs/ alibaba2/index.html). Among the eight *TLRs*, TLR-03466 with the greatest number of IRF-1 binding elements is the only virus inducible TLR in scaffold 599. *TLRs* 03466-D8 and 03466-D9, with comparatively reduced number of these regulatory elements are the only genes that are up-regulated in development; their regulation did not vary significant during immune challenge.


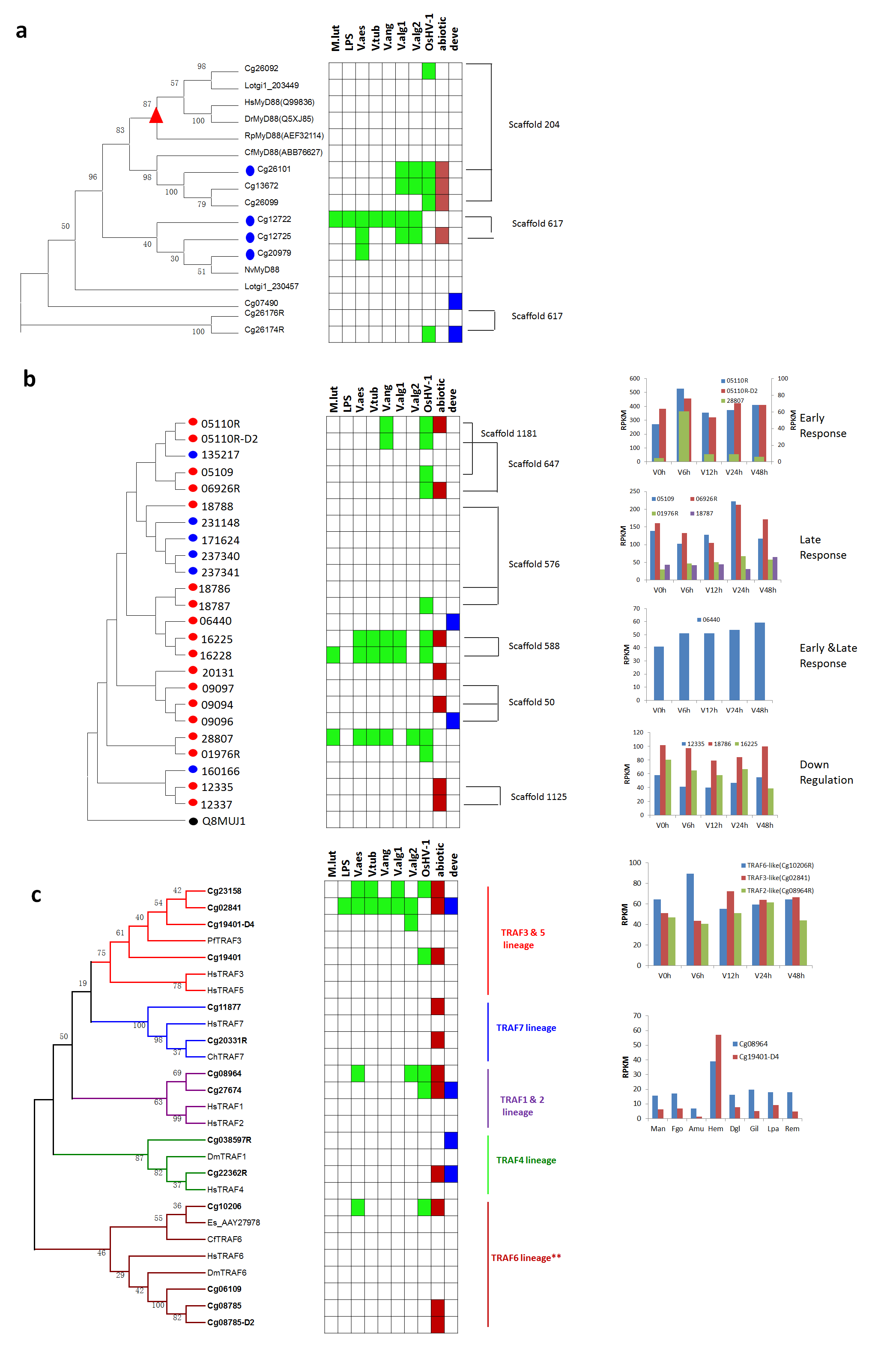


**Supplementary Figure 3 | Evolution and expression divergence of *MyD88-like*, *TNF* and *TRAF* gene families.**

**a** Neighbor-joining tree of MyD88 based on 17 MyD88 predicted proteins from: Pacific oyster (*Crassostrea gigas* - Cg), owl limpet (*Lottia gigantea* - Lotgi)*,* anemone (*Nematostella Vectensis* - Nv), human ([*Homo sapiens*](http://www.google.com.hk/search?hl=zh-CN&newwindow=1&safe=strict&biw=1191&bih=584&sa=X&ei=9ss9UMLzHJGeiAfLkYDwAQ&ved=0CB0QBSgA&q=Homo+spaniens&spell=1) - Hs), zebrafish (*Danio rerio* - Dr), clam (*Ruditapes philippinarum* - Rp) and scallop (*Chlamys farreri* – Cf). The short type MyD88s in oyster are marked with blue circles. Putative MyD88 orthologs in different species are denoted with a red triangle. The expression patterns of 10 oyster MyD88-like genes under biotic (green rectangle) and abiotic challenges (red rectangle) as well as during different developmental stages (blue rectangle) are indicated. The expression profiles from left to right are *Micrococcus luteus* (M. lut), LPS, *Vibrio aestuarianus* (V. aes), *V. tubiashii* (V. tub), *V. anguillarum* (V. ang), *V. alginolyticus-1* (V. alg1), *V. alginolyticus-2* (V. alg2), oyster herpes virus (OsHV-1), abiotic stressors and developmental stages. Cg26101, Cg20979, Cg12722 and Cg12725 lack the death domain. The gene IDs connected with black horizontal lines indicate that they are from the same scaffold. **b** (**Left**) Phylogenetic tree of oyster (red circle) and owl limpet (blue circle) TNFs; fruit fly (black circle) homolog serves as an outgroup. Green, red and blue boxes indicate differential expression of TNFs under biotic and abiotic challenges as well as during development. Both gene families exhibit lineage-specific expansion and functional diversification. Limpet (*Lottia gigantea*) and oyster *TNF* members cluster in a taxon-specific manner, suggesting that the expansion of oyster *TNFs* occurred after the divergence of the Bivalvia and Gastropoda. Tree was constructed by the neighbor-joining method, maximum likelihood method, which also was used for confirmation of topology structure. Robustness of the tree was assessed using bootstrapping (100 pseudoreplicates). (**Middle**) Expression pattern of CgTNFs. Color indicates genes that exhibit up-regulation under biotic (green) challenge, abiotic (red) challenge and specific expression at certain development stages (blue). The stressors from left to right are *Micrococcus luteus* (M. lut), LPS, *Vibrio aestuarianus* (V. aes), *V. tubiashii* (V. tub), *V. anguillarum* (V. ang), *V. alginolyticus-1* (V. alg1), *V. alginolyticus-2* (V. alg2), oyster herpes virus (OsHV-1) and abiotic stressors; developmental stages also are included. (**Right**) Expression of 11 TNFs underscores expression divergence in response to *Vibrio* time-course challenge. From top to bottom: early response up-regulation, later response up-regulation, up-regulation in both early and late time course and down-regulation. **c** (**Left**) Phylogenetic tree of TRAF. Tree was constructed using the maximum likelihood method and includes: Pacific oyster (*Crassostrea gigas* – Cg), human (*Homo sapiens* - Hs), fruit fly (*Drosophila melanogaster* - Dm), Hong Kong oyster (*Crassostrea hongkongensis* – Ch), pearl oyster (*Pinctada fucata* – Pf), zhikong scallop (*Chlamys farreri* – Cf) and bobtail squid (*Euprymna scolopes* - Es) TRAFs from the NCBI database. The oyster TRAFs are clustered into five subgroups: TRAF1 & 2 lineage, TRAF3 & 5 lineage, TRAF4 lineage, TRAF6 lineage and TRAF7 lineage, which are marked in different colors. Duplications are noted in lineage 6 (**). **(Middle)** Expression patterns of members of CgTRAF lineages. Colors indicate genes exhibiting up-regulation under biotic (green) challenge, abiotic (red) challenge and specific expression at certain development stages (blue). The stressors from left to right are *Micrococcus luteus* (M. lut), LPS, *Vibrio aestuarianus* (V. aes), *V. tubiashii* (V. tub), *V. anguillarum* (V. ang), *V. alginolyticus-1* (V. alg1), *V. alginolyticus-2* (V. alg2), oyster herpes virus (OsHV-1), abiotic stressors and different developmental stages. **(Left)** Expression of three TRAFs indicates differential expression in response to *Vibrio* in time-course challenges. Two TRAFs exhibit hemocyte- specific expression patterns. Organs and tissues used are: mantle (Man), female gonad (Fgo), adductor muscle (Amu), hemocytes (Hem), digestive gland (Dgl), gill (Gil), labial palp (Lpa) and male gonad (Mgo).

**
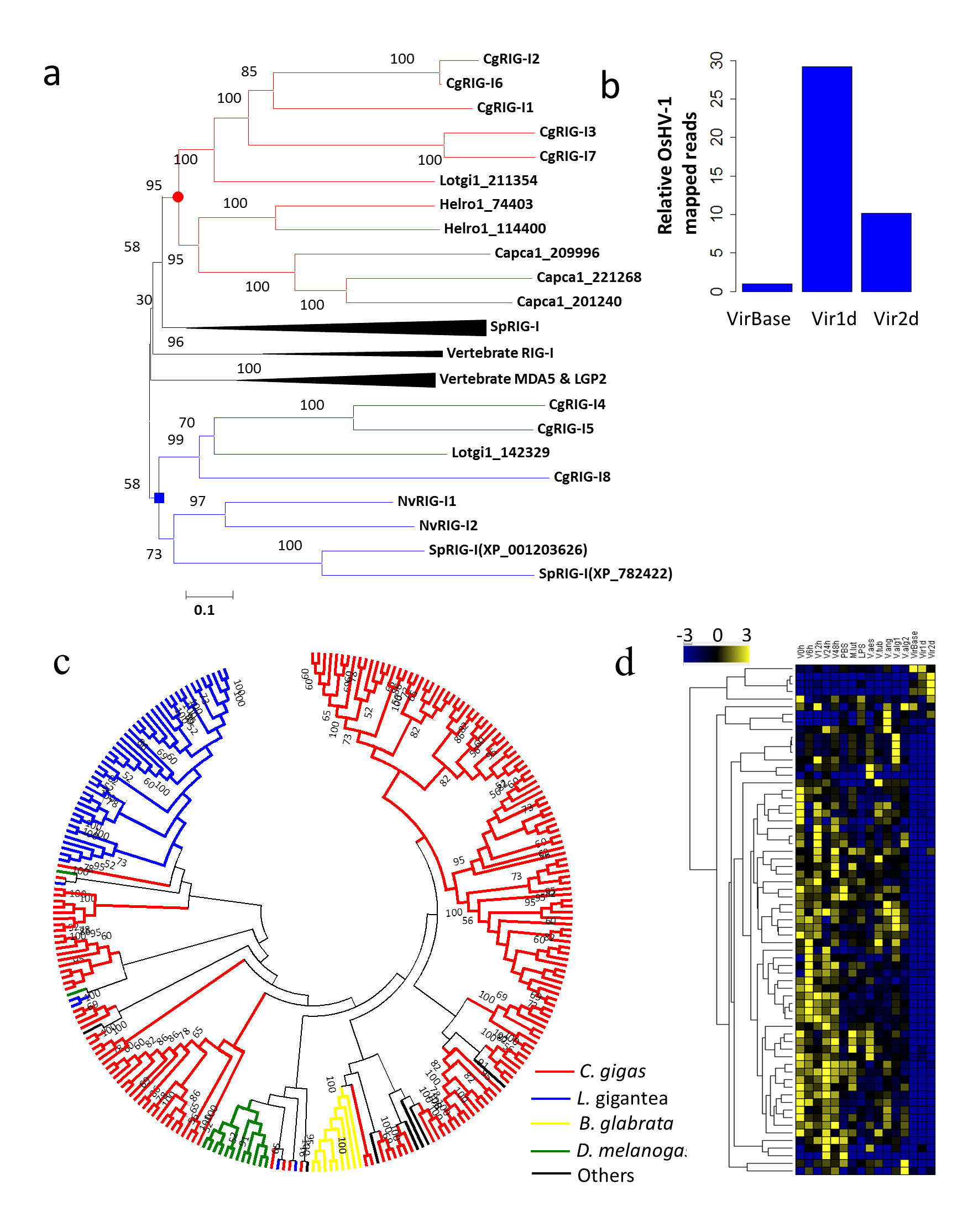
**

**Supplementary Figure 4 | The adaptive evolution of retinoic acid-inducible gene (RIG-I)-like receptor (*RLR*) and FBG domain containing protein (FBGDC).**

**a** Phylogenetic analysis of 34 RIG-like receptors from: Pacific oyster (*Crassostrea gigas -* Cg), owl limpet (*Lottia gigantea* - Lotgi), leech (*Helro robusta* - Helro), polychaete worm (*Capitella teleta* - Capca), sea urchin *(Strongylocentrotus purpuratus* - Sp), anemone (*Nematostella vectensis* - Nv) and two vertebrates. Tree was constructed by the neighbor-joining method; the maximum likelihood method also was used for confirmation of topology structure. Robustness of the tree was assessed using bootstrapping (100 pseudoreplicates). Oyster *RIG-1* genes cluster in two lineage-specific groups, of which one is a sister to *RIG-1* from lophotrochozoa and the other group, which may be of ancient origin, is a sister to *RIG-1* from sea anemone. **b** Change in sequence reads during infection with oyster herpes virus (OsHV-1). **c** Phylogenetic analysis is based on 248 *FBGDCs* from: Pacific oyster (*Crassostrea gigas* - red), owl limpet (*Lottia gigantea* - blue), snail (*Biomphalaria glabrata* - yellow), fruit fly (*Drosophila melanogaster* - green) and other invertebrate (black) sequences. Tree is based on the maximum likelihood method. In snail, FREP genes comprised of both Ig and a FBG domain undergo somatic change and point mutation in response to challenge (yellow line). Genes possessing Ig and FBG domains have not been identified in *C. gigas*. ^13^. **d** Expression dynamics of 67 differentially expressed *FBGDC* genes under biotic challenge including: over a time course with a pathogenic viral challenge and following challenge with four pathogenic *Vibrio* species: *V. anguillarum, V. tubiashii, V. aestuarianus* and *V. alginolyticus*, six types of bacteria (define) and LPS. Median-normalized expression levels have been sorted by expression RPKM at different time points (horizontal axis). Yellow indicates high RPKM and blue indicates low RPKM.


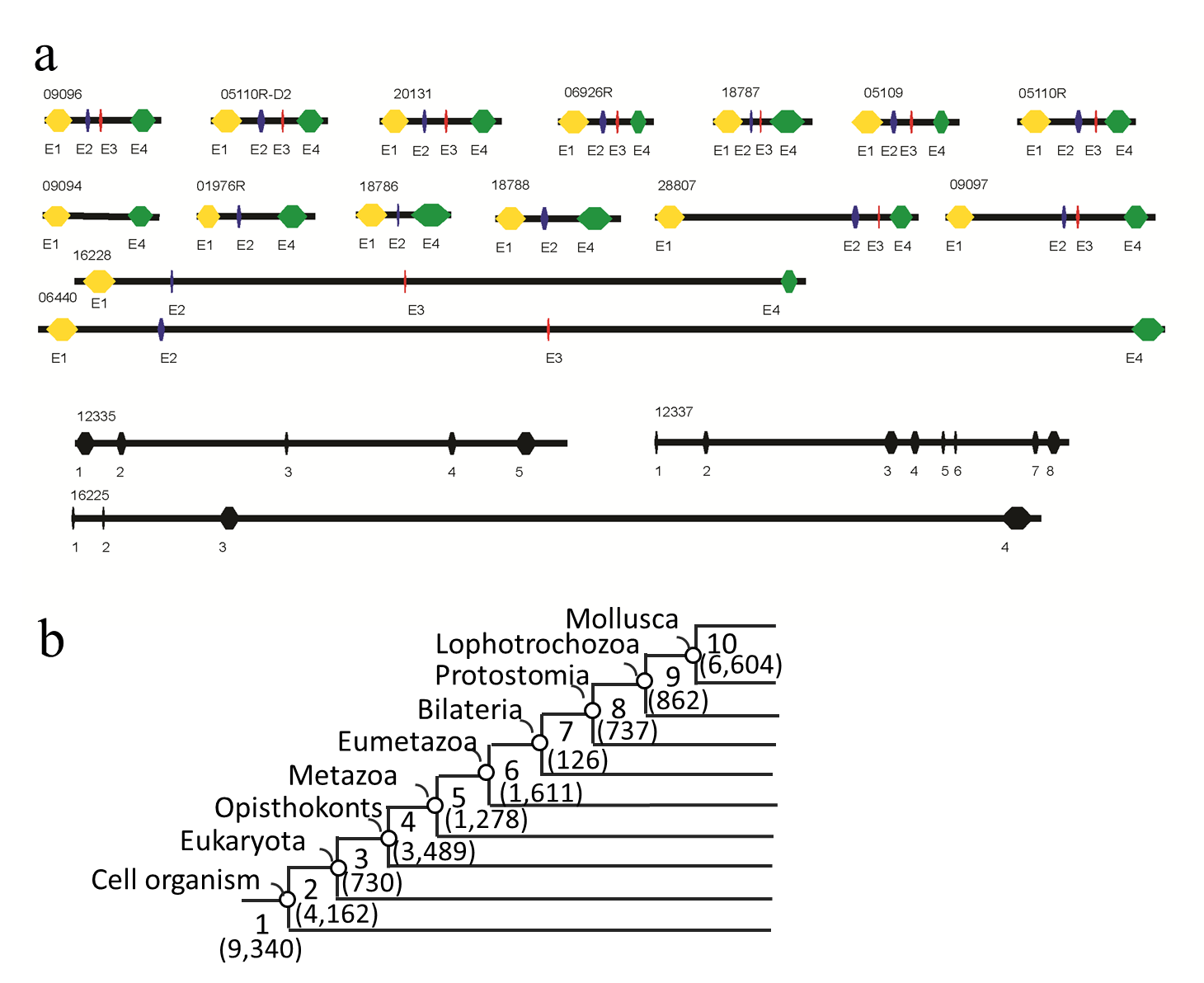


**Supplementary Figure 5 | Genomic architecture of the *TNFs* in the Pacific oyster and distribution of oyster genes for each phylostratum.** **a** The relative order of exons in 15 *TNFs* is conserved. All genes possess two longer exons flanking two shorter exons or exhibit related patterns of organization. Intron lengths vary appreciably. The yellow, blue, red and green colors indicate the four phylogenetically conserved exons: E1, E2, E3 and E4, respectively, from 5′ to 3′. (The bottom three) Exons of the three *TNF* genes that exhibit different TNF genome architectures are numbered (1-8); no homology is implied in the numbering sequences. **b** Numbers in parentheses show the number of genes in each phylostrata assigned and numbers outside of parentheses represent the assigned ages.


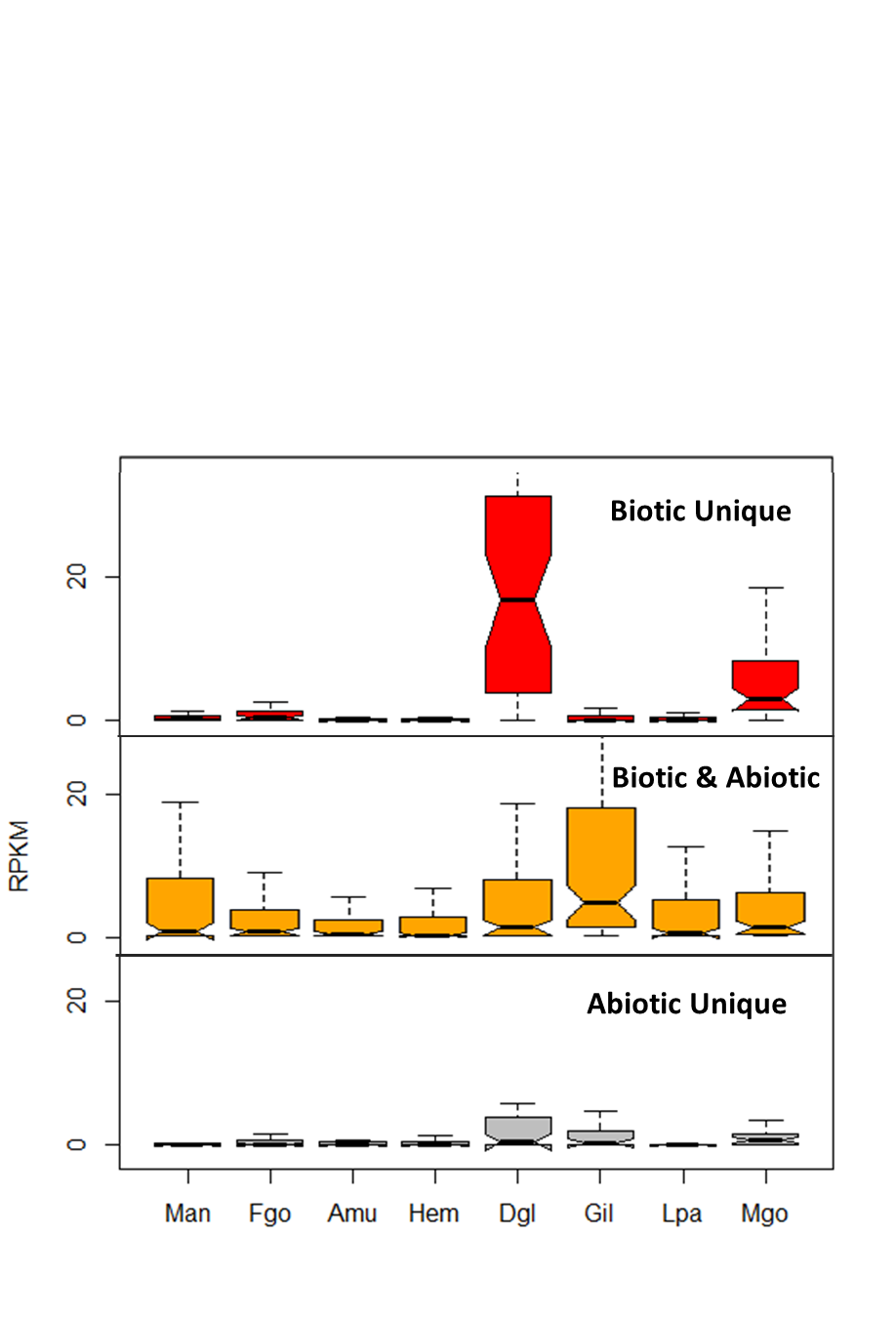


**Supplementary Figure 6 | Organ and tissue expression profiles suggest biotic response *C1qDCs* are highly expressed in digestive gland. Mantle (Man), female gonad (Fgo), adductor muscle (Amu), hemocytes (Hem), digestive gland (Dgl), gill (Gil), labial palp (Lpa) and male gonad (Mgo).**

**Supplementary Table 1 Immune-related genes found in Pacific oyster, cross-species comparison of the immune-related protein domains and summary of the phylostatigraphic ages using the method of phylostratigraphy**

1. **Immune-related genes found in Pacific oyster**

| **Gene Catergory** | **Number** | **Gene mode ID** | **Expression(RPKM>1)** | | |  | **Note** |
| --- | --- | --- | --- | --- | --- | --- | --- |
| I. IMMUNITY |  |  | Development | Tissue | Biotic | Abiotic |  |
| I.A1 Immune receptor-Pattern Recognition Receptors (PRRs) | | |  |  |  |  |  |
| Toll-like Receptors(TLRs) | 83 |  |  |  |  |  | sheet 2 |
| NACHT and leucine-rich Repeat receptors (NLRs) | 4 | Cgig_10052864 | Y | Y | Y | Y | CgNLR-1 |
|  |  | CGI_10005586 | Y | Y | Y | Y | CgNLR-2 |
|  |  | CGI_10012965 | Y | Y | Y | Y | CgNLR-3 |
|  |  | CGI_10028811 | Y | Y | Y | Y | CgNLR-4 |
| Scavenger Receptor Cysteine-Rich repeat protein (SRCR) | 71 | CGI_10000563 | Y | Y | N | N |  |
|  |  | CGI_10001510 | N | N | N | N |  |
|  |  | CGI_10001511 | Y | Y | Y | Y |  |
|  |  | CGI_10004025 | Y | Y | Y | Y |  |
|  |  | CGI_10005018 | Y | Y | N | N |  |
|  |  | CGI_10006371 | N | Y | N | N |  |
|  |  | CGI_10006693 | Y | Y | Y | Y |  |
|  |  | CGI_10007628 | Y | Y | Y | Y |  |
|  |  | CGI_10007783 | Y | Y | N | N |  |
|  |  | CGI_10007812 | Y | Y | Y | Y |  |
|  |  | CGI_10007951 | Y | N | Y | Y |  |
|  |  | CGI_10007953 | Y | Y | Y | Y |  |
|  |  | CGI_10008197 | Y | Y | N | N |  |
|  |  | CGI_10008422 | N | Y | Y | Y |  |
|  |  | CGI_10009063 | Y | Y | Y | T |  |
|  |  | CGI_10011074 | Y | Y | N | N |  |
|  |  | CGI_10011481 | Y | Y | Y | Y |  |
|  |  | CGI_10011852 | Y | Y | Y | Y |  |
|  |  | CGI_10012379 | Y | Y | N | Y |  |
|  |  | CGI_10012447 | Y | Y | Y | Y |  |
|  |  | CGI_10012562 | Y | Y | Y | Y |  |
|  |  | CGI_10012936 | Y | Y | N | N |  |
|  |  | CGI_10013540 | Y | N | Y | Y |  |
|  |  | CGI_10013769 | Y | Y | Y | Y |  |
|  |  | CGI_10013874 | Y | Y | Y | Y |  |
|  |  | CGI_10014442 | N | Y | Y | Y |  |
|  |  | CGI_10015479 | Y | Y | Y | Y |  |
|  |  | CGI_10015645 | Y | Y | Y | Y |  |
|  |  | CGI_10016401 | Y | Y | Y | Y |  |
|  |  | CGI_10016426 | Y | Y | Y | Y |  |
|  |  | CGI_10016467 | N | Y | Y | Y |  |
|  |  | CGI_10016651 | N | Y | Y | Y |  |
|  |  | CGI_10017479 | Y | Y | Y | Y |  |
|  |  | CGI_10017884 | Y | Y | Y | Y |  |
|  |  | CGI_10018394 | Y | Y | Y | Y |  |
|  |  | CGI_10018395 | Y | Y | Y | Y |  |
|  |  | CGI_10018684 | Y | Y | Y | Y |  |
|  |  | CGI_10018753 | Y | Y | Y | Y |  |
|  |  | CGI_10018754 | Y | Y | Y | Y |  |
|  |  | CGI_10018755 | N | Y | Y | Y |  |
|  |  | CGI_10018871 | Y | Y | Y | Y |  |
|  |  | CGI_10018872 | Y | Y | N | Y |  |
|  |  | CGI_10019298 | Y | Y | Y | Y |  |
|  |  | CGI_10020472 | Y | Y | Y | Y |  |
|  |  | CGI_10020473 | Y | Y | Y | Y |  |
|  |  | CGI_10020474 | Y | Y | Y | Y |  |
|  |  | CGI_10020579 | Y | Y | Y | Y |  |
|  |  | CGI_10020700 | Y | Y | Y | Y |  |
|  |  | CGI_10020771 | Y | N | N | N |  |
|  |  | CGI_10021829 | Y | Y | Y | Y |  |
|  |  | CGI_10022218 | Y | Y | Y | Y |  |
|  |  | CGI_10022225 | Y | Y | Y | Y |  |
|  |  | CGI_10022550 | Y | Y | Y | Y |  |
|  |  | CGI_10022827 | N | N | N | N |  |
|  |  | CGI_10022828 | N | N | N | N |  |
|  |  | CGI_10023034 | N | N | Y | Y |  |
|  |  | CGI_10023196 | Y | Y | Y | Y |  |
|  |  | CGI_10023417 | N | Y | N | N |  |
|  |  | CGI_10023866 | Y | Y | Y | Y |  |
|  |  | CGI_10024222 | N | N | Y | Y |  |
|  |  | CGI_10024452 | Y | Y | Y | Y |  |
|  |  | CGI_10025335 | N | N | Y | Y |  |
|  |  | CGI_10025337 | N | Y | Y | Y |  |
|  |  | CGI_10025339 | N | Y | N | Y |  |
|  |  | CGI_10025439 | N | Y | Y | Y |  |
|  |  | CGI_10025773 | Y | Y | Y | Y |  |
|  |  | CGI_10026985 | Y | Y | Y | Y |  |
|  |  | CGI_10027018 | N | Y | Y | Y |  |
|  |  | CGI_10027681 | Y | Y | Y | Y |  |
|  |  | CGI_10028387 | Y | Y | Y | Y |  |
|  |  | CGI_10028833 | N | Y | Y | Y |  |
| CD36-like | 2 | CGI_10007366 | Y | Y | Y | Y |  |
|  |  | CGI_10027519 | Y | Y | Y | Y |  |
| Peptidoglycan Recognition Proteins (PGRP) | 9 | CGI_10001975 | Y | Y | Y | Y |  |
|  |  | CGI_10027188 | N | Y | Y | Y |  |
|  |  | CGI_10027186 | Y | Y | N | Y |  |
|  |  | CGI_10009905 | Y | Y | Y | Y |  |
|  |  | CGI_10001545 | Y | Y | Y | Y |  |
|  |  | CGI_10025288 | Y | Y | Y | Y |  |
|  |  | CGI_10027187 | Y | Y | N | Y |  |
|  |  | CGI_10028092 | Y | Y | Y | Y |  |
|  |  | CGI_10028827 | N | Y | N | N |  |
| β-1,3-Glucan recognition protein (βGRP) | 5 | CGI_10013834 | Y | Y | Y | Y |  |
|  |  | CGI_10008341 | Y | Y | Y | Y |  |
|  |  | CGI_10027688 | Y | Y | N | N |  |
|  |  | CGI_10027689 | Y | Y | N | Y |  |
|  |  | CGI_10000576 | Y | Y | Y | Y |  |
| RIG-I like receptor (RLR) | 11 | CGI_10024392R | Y | Y | Y | Y | CgRIG-I1 |
|  |  | CGI_10022973 | Y | Y | Y | Y | CgRIG-I2 |
|  |  | CGI_10028631 | Y | Y | Y | Y | CgRIG-I3 |
|  |  | CGI_10014034 | Y | Y | Y | Y | CgRIG-I4 |
|  |  | CGI_10010459 | Y | Y | Y | Y | CgRIG-I5 |
|  |  | CGI_10010459R-D2 | Y | Y | Y | Y | CgRIG-I6 |
|  |  | CGI_10025133 | Y | Y | Y | Y | CgRIG-I7 |
|  |  | CGI_10016990R | Y | Y | Y | Y | CgRIG-I8 |
|  |  | CGI_10000225 | Y | Y | Y | Y | CgRIG-I9 |
|  |  | CGI_10014035 | Y | Y | Y | Y | CgRIG-I10 |
|  |  | CGI_10024393 | Y | Y | Y | Y | CgRIG-I11 |
| Mitochondrial antiviral-signaling protein (MAVS) | 1 | CgMAVS | Y | Y | Y | Y |  |
| Transmembrane protein 173 (MITA) | 5 | CGI_10003079 | Y | Y | Y | Y |  |
|  |  | CGI_10012051 | Y | Y | Y | Y |  |
|  |  | CGI_10022970 | Y | Y | Y | Y |  |
|  |  | CGI_10006439 | Y | Y | Y | Y |  |
|  |  | CGI_10004069 | Y | Y | Y | Y |  |
| 1.A2 Immune receptors-lectins | |  |  |  |  |  |  |
| C-type lectin domain proteins (CTLDC) | 266 | CGI_10000016 | Y | Y | Y | Y |  |
|  |  | CGI_10000359 | Y | Y | Y | Y |  |
|  |  | CGI_10000503 | Y | N | N | Y |  |
|  |  | CGI_10017889 | Y | Y | Y | N |  |
|  |  | CGI_10001189 | Y | Y | Y | Y |  |
|  |  | CGI_10001400 | Y | Y | Y | Y |  |
|  |  | CGI_10001434 | N | Y | N | N |  |
|  |  | CGI_10001708 | N | N | N | N |  |
|  |  | CGI_10001743 | Y | Y | N | N |  |
|  |  | CGI_10002246 | Y | N | N | N |  |
|  |  | CGI_10002325 | Y | N | N | Y |  |
|  |  | CGI_10002529 | Y | Y | Y | Y |  |
|  |  | CGI_10002753 | N | Y | Y | Y |  |
|  |  | CGI_10002756 | N | Y | Y | Y |  |
|  |  | CGI_10002838 | Y | Y | Y | Y |  |
|  |  | CGI_10002839 | Y | Y | Y | Y |  |
|  |  | CGI_10002963 | Y | Y | N | Y |  |
|  |  | CGI_10003002 | Y | N | N | N |  |
|  |  | CGI_10003003 | Y | Y | N | Y |  |
|  |  | CGI_10003004 | N | Y | Y | Y |  |
|  |  | CGI_10003119 | N | Y | Y | Y |  |
|  |  | CGI_10003144 | N | N | Y | Y |  |
|  |  | CGI_10003221 | N | Y | N | N |  |
|  |  | CGI_10003256 | N | Y | N | N |  |
|  |  | CGI_10003266 | Y | Y | Y | Y |  |
|  |  | CGI_10003440 | Y | Y | Y | Y |  |
|  |  | CGI_10003643 | Y | Y | Y | Y |  |
|  |  | CGI_10003656 | Y | Y | Y | Y |  |
|  |  | CGI_10003747 | Y | Y | Y | Y |  |
|  |  | CGI_10003823 | Y | Y | Y | Y |  |
|  |  | CGI_10003895 | Y | Y | Y | Y |  |
|  |  | CGI_10004277 | Y | Y | N | N |  |
|  |  | CGI_10004791 | Y | Y | Y | Y |  |
|  |  | CGI_10004828 | Y | Y | Y | Y |  |
|  |  | CGI_10004933 | Y | Y | Y | Y |  |
|  |  | CGI_10005100 | Y | N | N | Y |  |
|  |  | CGI_10005141 | Y | Y | N | N |  |
|  |  | CGI_10005177 | N | Y | N | N |  |
|  |  | CGI_10005274 | Y | Y | N | Y |  |
|  |  | CGI_10005326 | N | Y | N | Y |  |
|  |  | CGI_10005327 | N | Y | Y | Y |  |
|  |  | CGI_10005510 | N | Y | Y | Y |  |
|  |  | CGI_10005511 | N | N | Y | Y |  |
|  |  | CGI_10005529 | Y | Y | Y | Y |  |
|  |  | CGI_10005567 | Y | Y | Y | Y |  |
|  |  | CGI_10005781 | Y | Y | Y | Y |  |
|  |  | CGI_10005862 | Y | Y | Y | Y |  |
|  |  | CGI_10006366 | N | N | Y | Y |  |
|  |  | CGI_10006367 | Y | Y | Y | Y |  |
|  |  | CGI_10006368 | Y | Y | N | N |  |
|  |  | CGI_10006493 | N | N | N | N |  |
|  |  | CGI_10006495 | Y | Y | Y | Y |  |
|  |  | CGI_10006917 | Y | N | N | Y |  |
|  |  | CGI_10006918 | Y | N | N | N |  |
|  |  | CGI_10006919 | Y | N | N | N |  |
|  |  | CGI_10006920 | Y | Y | N | Y |  |
|  |  | CGI_10006921 | Y | N | N | N |  |
|  |  | CGI_10006922 | Y | Y | N | Y |  |
|  |  | CGI_10007353 | Y | Y | Y | N |  |
|  |  | CGI_10007386 | N | Y | Y | Y |  |
|  |  | CGI_10007445 | Y | Y | Y | Y |  |
|  |  | CGI_10007840 | Y | Y | Y | Y |  |
|  |  | CGI_10008038 | Y | N | N | N |  |
|  |  | CGI_10008131 | Y | Y | Y | Y |  |
|  |  | CGI_10008310 | N | Y | Y | Y |  |
|  |  | CGI_10008321 | Y | Y | N | Y |  |
|  |  | CGI_10008626 | Y | Y | N | N |  |
|  |  | CGI_10008643 | N | N | Y | Y |  |
|  |  | CGI_10008644 | N | N | N | N |  |
|  |  | CGI_10008940 | N | Y | Y | Y |  |
|  |  | CGI_10009161 | Y | N | Y | N |  |
|  |  | CGI_10009290 | Y | Y | Y | Y |  |
|  |  | CGI_10009560 | Y | Y | Y | Y |  |
|  |  | CGI_10009691 | Y | Y | N | N |  |
|  |  | CGI_10009692 | Y | N | Y | N |  |
|  |  | CGI_10009934 | N | N | Y | Y |  |
|  |  | CGI_10010004 | Y | N | N | N |  |
|  |  | CGI_10010033 | Y | Y | N | N |  |
|  |  | CGI_10010035 | Y | Y | N | N |  |
|  |  | CGI_10010245 | Y | Y | Y | Y |  |
|  |  | CGI_10010246 | Y | Y | N | Y |  |
|  |  | CGI_10010247 | Y | Y | N | Y |  |
|  |  | CGI_10010248 | Y | Y | Y | Y |  |
|  |  | CGI_10010346 | Y | Y | N | Y |  |
|  |  | CGI_10010505 | Y | Y | N | Y |  |
|  |  | CGI_10010614 | Y | N | N | Y |  |
|  |  | CGI_10010615 | Y | N | N | N |  |
|  |  | CGI_10010691 | N | N | Y | N |  |
|  |  | CGI_10010693 | N | N | Y | Y |  |
|  |  | CGI_10011027 | N | Y | Y | Y |  |
|  |  | CGI_10011074 | Y | Y | N | N |  |
|  |  | CGI_10011253 | Y | Y | Y | Y |  |
|  |  | CGI_10011368 | N | Y | Y | Y |  |
|  |  | CGI_10011669 | Y | Y | Y | Y |  |
|  |  | CGI_10011670 | Y | Y | Y | Y |  |
|  |  | CGI_10011671 | Y | Y | Y | Y |  |
|  |  | CGI_10011672 | N | N | N | N |  |
|  |  | CGI_10011673 | Y | Y | Y | Y |  |
|  |  | CGI_10011856 | Y | N | N | Y |  |
|  |  | CGI_10012132 | Y | Y | Y | Y |  |
|  |  | CGI_10012135 | Y | Y | Y | Y |  |
|  |  | CGI_10012174 | Y | Y | Y | Y |  |
|  |  | CGI_10012211 | Y | Y | Y | Y |  |
|  |  | CGI_10012284 | Y | Y | Y | Y |  |
|  |  | CGI_10012426 | Y | Y | Y | Y |  |
|  |  | CGI_10012471 | Y | Y | N | Y |  |
|  |  | CGI_10012515 | N | Y | Y | Y |  |
|  |  | CGI_10012516 | N | Y | Y | Y |  |
|  |  | CGI_10012756 | Y | Y | Y | Y |  |
|  |  | CGI_10012778 | Y | Y | N | N |  |
|  |  | CGI_10013049 | N | Y | Y | Y |  |
|  |  | CGI_10013133 | Y | Y | Y | Y |  |
|  |  | CGI_10013598 | Y | Y | Y | Y |  |
|  |  | CGI_10014286 | Y | N | Y | N |  |
|  |  | CGI_10014497 | Y | Y | Y | Y |  |
|  |  | CGI_10014510 | N | N | N | Y |  |
|  |  | CGI_10014511 | Y | Y | N | Y |  |
|  |  | CGI_10014755 | Y | Y | Y | Y |  |
|  |  | CGI_10014830 | Y | Y | Y | N |  |
|  |  | CGI_10014903 | Y | Y | Y | Y |  |
|  |  | CGI_10014905 | Y | Y | Y | Y |  |
|  |  | CGI_10015011 | Y | Y | Y | N |  |
|  |  | CGI_10015470 | Y | N | Y | Y |  |
|  |  | CGI_10015485 | N | N | N | N |  |
|  |  | CGI_10015534 | Y | Y | Y | Y |  |
|  |  | CGI_10015627 | N | Y | Y | Y |  |
|  |  | CGI_10015654 | N | Y | N | N |  |
|  |  | CGI_10015655 | Y | N | Y | Y |  |
|  |  | CGI_10015759 | Y | Y | N | N |  |
|  |  | CGI_10015830 | Y | Y | Y | Y |  |
|  |  | CGI_10015880 | N | Y | N | Y |  |
|  |  | CGI_10016582 | Y | Y | N | N |  |
|  |  | CGI_10016583 | Y | Y | N | N |  |
|  |  | CGI_10016584 | Y | Y | N | N |  |
|  |  | CGI_10016703 | Y | N | Y | N |  |
|  |  | CGI_10016714 | Y | Y | N | N |  |
|  |  | CGI_10016849 | Y | Y | Y | N |  |
|  |  | CGI_10016903 | N | Y | N | N |  |
|  |  | CGI_10017462 | N | Y | Y | Y |  |
|  |  | CGI_10017463 | Y | Y | Y | Y |  |
|  |  | CGI_10017464 | Y | Y | Y | Y |  |
|  |  | CGI_10017690 | Y | Y | N | Y |  |
|  |  | CGI_10017879 | Y | Y | N | Y |  |
|  |  | CGI_10017889 | Y | Y | Y | Y |  |
|  |  | CGI_10017998 | Y | Y | Y | Y |  |
|  |  | CGI_10018105 | Y | N | N | Y |  |
|  |  | CGI_10018363 | N | Y | Y | Y |  |
|  |  | CGI_10018364 | N | Y | Y | Y |  |
|  |  | CGI_10018426 | N | N | Y | Y |  |
|  |  | CGI_10018514 | Y | Y | Y | Y |  |
|  |  | CGI_10018515 | Y | Y | N | Y |  |
|  |  | CGI_10018516 | Y | Y | Y | Y |  |
|  |  | CGI_10018541 | Y | Y | Y | Y |  |
|  |  | CGI_10018554 | Y | Y | Y | Y |  |
|  |  | CGI_10018556 | Y | Y | Y | Y |  |
|  |  | CGI_10018609 | Y | Y | Y | Y |  |
|  |  | CGI_10018813 | N | Y | Y | N |  |
|  |  | CGI_10018822 | Y | Y | Y | Y |  |
|  |  | CGI_10019184 | Y | Y | Y | Y |  |
|  |  | CGI_10019212 | Y | Y | Y | Y |  |
|  |  | CGI_10019215 | Y | Y | N | N |  |
|  |  | CGI_10019300 | N | Y | N | N |  |
|  |  | CGI_10019317 | N | Y | N | N |  |
|  |  | CGI_10019375 | Y | Y | N | N |  |
|  |  | CGI_10019379 | Y | Y | N | N |  |
|  |  | CGI_10019381 | Y | N | N | Y |  |
|  |  | CGI_10019407 | Y | N | N | N |  |
|  |  | CGI_10019843 | Y | Y | Y | Y |  |
|  |  | CGI_10020264 | Y | Y | Y | Y |  |
|  |  | CGI_10020290 | Y | Y | N | N |  |
|  |  | CGI_10020292 | Y | N | N | N |  |
|  |  | CGI_10020508 | Y | Y | Y | Y |  |
|  |  | CGI_10020693 | Y | Y | N | Y |  |
|  |  | CGI_10020694 | Y | Y | Y | Y |  |
|  |  | CGI_10020742 | Y | Y | Y | Y |  |
|  |  | CGI_10021084 | Y | Y | N | N |  |
|  |  | CGI_10021085 | Y | Y | Y | N |  |
|  |  | CGI_10021137 | N | Y | Y | Y |  |
|  |  | CGI_10021150 | Y | Y | N | N |  |
|  |  | CGI_10021262 | Y | Y | Y | Y |  |
|  |  | CGI_10021328 | Y | Y | N | N |  |
|  |  | CGI_10021329 | Y | Y | N | N |  |
|  |  | CGI_10021456 | N | Y | Y | Y |  |
|  |  | CGI_10021615 | Y | Y | Y | Y |  |
|  |  | CGI_10021658 | N | Y | Y | Y |  |
|  |  | CGI_10021829 | Y | Y | Y | Y |  |
|  |  | CGI_10022082 | Y | Y | Y | Y |  |
|  |  | CGI_10022251 | Y | Y | Y | Y |  |
|  |  | CGI_10022406 | N | N | N | Y |  |
|  |  | CGI_10022626 | Y | Y | Y | Y |  |
|  |  | CGI_10022627 | Y | Y | N | N |  |
|  |  | CGI_10022708 | N | Y | Y | Y |  |
|  |  | CGI_10022709 | N | Y | Y | Y |  |
|  |  | CGI_10022955 | Y | Y | N | Y |  |
|  |  | CGI_10023035 | Y | Y | N | N |  |
|  |  | CGI_10023126 | Y | Y | Y | Y |  |
|  |  | CGI_10023319 | Y | Y | N | N |  |
|  |  | CGI_10023421 | Y | Y | Y | Y |  |
|  |  | CGI_10023546 | N | Y | N | N |  |
|  |  | CGI_10023643 | Y | Y | Y | Y |  |
|  |  | CGI_10023695 | Y | Y | N | N |  |
|  |  | CGI_10023802 | Y | Y | Y | Y |  |
|  |  | CGI_10023808 | Y | Y | Y | Y |  |
|  |  | CGI_10023840 | N | Y | Y | Y |  |
|  |  | CGI_10023970 | Y | Y | Y | Y |  |
|  |  | CGI_10023978 | Y | Y | Y | Y |  |
|  |  | CGI_10024093 | N | Y | Y | Y |  |
|  |  | CGI_10024122 | Y | Y | Y | Y |  |
|  |  | CGI_10024123 | Y | Y | Y | Y |  |
|  |  | CGI_10024211 | Y | Y | Y | Y |  |
|  |  | CGI_10024327 | N | N | Y | Y |  |
|  |  | CGI_10024415 | Y | Y | Y | Y |  |
|  |  | CGI_10024458 | N | N | N | N |  |
|  |  | CGI_10024634 | N | N | Y | Y |  |
|  |  | CGI_10024723 | Y | Y | Y | Y |  |
|  |  | CGI_10024765 | Y | Y | Y | Y |  |
|  |  | CGI_10024769 | Y | N | Y | N |  |
|  |  | CGI_10024876 | Y | Y | N | Y |  |
|  |  | CGI_10024882 | Y | Y | Y | Y |  |
|  |  | CGI_10024932 | Y | Y | Y | Y |  |
|  |  | CGI_10024933 | Y | Y | Y | Y |  |
|  |  | CGI_10025155 | Y | Y | N | Y |  |
|  |  | CGI_10025207 | Y | Y | Y | Y |  |
|  |  | CGI_10025240 | Y | Y | Y | Y |  |
|  |  | CGI_10025357 | Y | Y | Y | Y |  |
|  |  | CGI_10025691 | Y | Y | Y | Y |  |
|  |  | CGI_10025847 | Y | Y | Y | Y |  |
|  |  | CGI_10025848 | Y | Y | Y | Y |  |
|  |  | CGI_10026070 | Y | Y | N | N |  |
|  |  | CGI_10026175 | Y | Y | Y | Y |  |
|  |  | CGI_10026204 | Y | N | N | N |  |
|  |  | CGI_10026206 | N | N | N | N |  |
|  |  | CGI_10026207 | Y | Y | Y | N |  |
|  |  | CGI_10026235 | N | N | N | N |  |
|  |  | CGI_10026489 | Y | Y | N | Y |  |
|  |  | CGI_10026560 | N | Y | Y | Y |  |
|  |  | CGI_10026561 | Y | Y | Y | Y |  |
|  |  | CGI_10026597 | Y | Y | Y | Y |  |
|  |  | CGI_10026912 | Y | Y | N | N |  |
|  |  | CGI_10026922 | Y | Y | N | Y |  |
|  |  | CGI_10026977 | Y | Y | N | Y |  |
|  |  | CGI_10027448 | Y | Y | Y | Y |  |
|  |  | CGI_10027555 | Y | Y | Y | Y |  |
|  |  | CGI_10027557 | Y | Y | N | N |  |
|  |  | CGI_10027561 | N | Y | Y | Y |  |
|  |  | CGI_10027625 | Y | Y | N | N |  |
|  |  | CGI_10027638 | Y | Y | N | N |  |
|  |  | CGI_10027653 | Y | N | Y | Y |  |
|  |  | CGI_10027654 | Y | N | Y | Y |  |
|  |  | CGI_10027739 | Y | Y | Y | Y |  |
|  |  | CGI_10027778 | Y | Y | Y | Y |  |
|  |  | CGI_10027794 | Y | Y | Y | Y |  |
|  |  | CGI_10027808 | Y | Y | Y | N |  |
|  |  | CGI_10027809 | N | Y | N | N |  |
|  |  | CGI_10027920 | Y | Y | N | N |  |
|  |  | CGI_10027921 | Y | Y | N | N |  |
|  |  | CGI_10027922 | Y | Y | N | N |  |
|  |  | CGI_10027935 | Y | N | N | Y |  |
|  |  | CGI_10028066 | N | Y | Y | Y |  |
|  |  | CGI_10028113 | Y | N | N | N |  |
|  |  | CGI_10028219 | Y | Y | N | Y |  |
|  |  | CGI_10028583 | Y | Y | N | Y |  |
|  |  | CGI_10028777 | Y | N | Y | Y |  |
|  |  | CGI_10028836 | Y | Y | Y | Y |  |
|  |  | CGI_10028895 | N | N | N | N |  |
|  |  | CGI_10017889 | Y | Y | N | N |  |
| Galectin | 11 | CGI_10005550 | Y | Y | Y | Y |  |
|  |  | CGI_10009751 | N | Y | Y | Y |  |
|  |  | CGI_10009817 | Y | Y | Y | Y |  |
|  |  | CGI_10014752 | Y | Y | Y | Y |  |
|  |  | CGI_10015449 | Y | Y | Y | Y |  |
|  |  | CGI_10015451 | N | Y | Y | Y |  |
|  |  | CGI_10022939 | Y | Y | Y | Y |  |
|  |  | CGI_10022940 | Y | Y | Y | Y |  |
|  |  | CGI_10022941 | Y | Y | Y | Y |  |
|  |  | CGI_10023949 | N | Y | Y | Y |  |
|  |  | CGI_10023950 | Y | Y | Y | Y |  |
| Mannose-6-phosphate receptor (M6P) | 3 | CGI_10013150 | Y | Y | Y | Y |  |
|  |  | CGI_10012347 | Y | Y | Y | Y |  |
|  |  | CGI_10019056 | Y | Y | Y | Y |  |
| 1.A3 Immune receptors-FBG-domain containing protein | | |  |  |  |  |  |
| FBG-domain containing proteins (FBGDC) | 190 | CGI_10000003 | Y | Y | Y | Y |  |
|  |  | CGI_10000088 | Y | Y | Y | Y |  |
|  |  | CGI_10000212 | N | Y | Y | Y |  |
|  |  | CGI_10000434 | Y | Y | Y | Y |  |
|  |  | CGI_10000643 | N | N | N | N |  |
|  |  | CGI_10000672 | N | Y | Y | Y |  |
|  |  | CGI_10000736 | N | Y | N | N |  |
|  |  | CGI_10000959 | N | Y | N | N |  |
|  |  | CGI_10000986 | Y | Y | Y | Y |  |
|  |  | CGI_10001352 | N | Y | Y | Y |  |
|  |  | CGI_10002044 | N | Y | Y | Y |  |
|  |  | CGI_10002045 | N | N | Y | Y |  |
|  |  | CGI_10002099 | N | Y | Y | Y |  |
|  |  | CGI_10002148 | N | N | N | N |  |
|  |  | CGI_10002149 | N | N | N | N |  |
|  |  | CGI_10002258 | Y | Y | N | Y |  |
|  |  | CGI_10002369 | Y | N | Y | Y |  |
|  |  | CGI_10002370 | Y | Y | Y | Y |  |
|  |  | CGI_10002427 | Y | Y | Y | Y |  |
|  |  | CGI_10002660 | Y | Y | Y | Y |  |
|  |  | CGI_10002665 | Y | Y | Y | Y |  |
|  |  | CGI_10002908 | Y | Y | Y | Y |  |
|  |  | CGI_10003063 | Y | Y | Y | Y |  |
|  |  | CGI_10003122 | Y | Y | Y | Y |  |
|  |  | CGI_10003123 | N | Y | Y | Y |  |
|  |  | CGI_10003158 | Y | Y | Y | Y |  |
|  |  | CGI_10003187 | N | N | N | N |  |
|  |  | CGI_10003302 | N | N | Y | Y |  |
|  |  | CGI_10003303 | Y | Y | Y | Y |  |
|  |  | CGI_10003304 | N | Y | Y | Y |  |
|  |  | CGI_10003902 | N | N | N | Y |  |
|  |  | CGI_10004043 | N | Y | Y | Y |  |
|  |  | CGI_10004278 | Y | Y | Y | Y |  |
|  |  | CGI_10004280 | Y | Y | Y | Y |  |
|  |  | CGI_10004282 | N | Y | N | N |  |
|  |  | CGI_10004283 | Y | N | N | N |  |
|  |  | CGI_10004381 | Y | Y | Y | Y |  |
|  |  | CGI_10004382 | Y | N | Y | Y |  |
|  |  | CGI_10004409 | Y | Y | Y | Y |  |
|  |  | CGI_10004425 | N | Y | Y | Y |  |
|  |  | CGI_10005170 | N | Y | Y | Y |  |
|  |  | CGI_10005275 | Y | N | Y | Y |  |
|  |  | CGI_10005574 | N | Y | Y | Y |  |
|  |  | CGI_10005575 | Y | Y | Y | Y |  |
|  |  | CGI_10005577 | Y | Y | N | N |  |
|  |  | CGI_10005757 | Y | Y | Y | Y |  |
|  |  | CGI_10005774 | Y | Y | Y | Y |  |
|  |  | CGI_10006226 | N | Y | Y | Y |  |
|  |  | CGI_10006228 | N | Y | Y | Y |  |
|  |  | CGI_10006229 | N | Y | Y | Y |  |
|  |  | CGI_10006490 | Y | Y | Y | Y |  |
|  |  | CGI_10006673 | Y | Y | Y | Y |  |
|  |  | CGI_10006674 | Y | Y | N | Y |  |
|  |  | CGI_10006675 | Y | N | N | N |  |
|  |  | CGI_10007042 | N | Y | Y | Y |  |
|  |  | CGI_10007043 | N | Y | Y | Y |  |
|  |  | CGI_10007600 | N | N | N | N |  |
|  |  | CGI_10007601 | N | Y | N | N |  |
|  |  | CGI_10007602 | N | N | N | N |  |
|  |  | CGI_10007603 | N | Y | N | Y |  |
|  |  | CGI_10007604 | Y | N | N | N |  |
|  |  | CGI_10007645 | Y | Y | Y | Y |  |
|  |  | CGI_10007991 | Y | Y | Y | Y |  |
|  |  | CGI_10007993R1 | Y | Y | Y | Y |  |
|  |  | CGI_10007993R2 | Y | Y | Y | Y |  |
|  |  | CGI_10008137 | N | N | N | N |  |
|  |  | CGI_10008354 | Y | N | N | N |  |
|  |  | CGI_10008454 | Y | Y | Y | Y |  |
|  |  | CGI_10008543 | Y | Y | Y | Y |  |
|  |  | CGI_10008641 | Y | Y | Y | Y |  |
|  |  | CGI_10008742 | N | N | Y | Y |  |
|  |  | CGI_10008744 | N | Y | Y | Y |  |
|  |  | CGI_10008747 | Y | Y | Y | Y |  |
|  |  | CGI_10008773 | N | N | N | Y |  |
|  |  | CGI_10008845 | Y | Y | Y | Y |  |
|  |  | CGI_10009080 | N | Y | N | N |  |
|  |  | CGI_10009082 | N | Y | Y | Y |  |
|  |  | CGI_10009083 | N | Y | N | N |  |
|  |  | CGI_10009232 | Y | Y | Y | N |  |
|  |  | CGI_10009242 | Y | Y | Y | Y |  |
|  |  | CGI_10009405 | Y | Y | N | N |  |
|  |  | CGI_10009439 | N | Y | Y | Y |  |
|  |  | CGI_10009440 | N | Y | Y | Y |  |
|  |  | CGI_10009597 | N | N | N | N |  |
|  |  | CGI_10009600 | N | N | N | N |  |
|  |  | CGI_10010018 | Y | Y | Y | Y |  |
|  |  | CGI_10010443 | Y | Y | Y | Y |  |
|  |  | CGI_10010517 | Y | Y | Y | Y |  |
|  |  | CGI_10010804 | N | Y | N | N |  |
|  |  | CGI_10010806 | N | N | Y | N |  |
|  |  | CGI_10011310 | Y | Y | Y | Y |  |
|  |  | CGI_10011455 | N | N | N | N |  |
|  |  | CGI_10011462 | N | Y | Y | Y |  |
|  |  | CGI_10011664 | Y | N | N | Y |  |
|  |  | CGI_10011665 | Y | N | N | N |  |
|  |  | CGI_10012280 | Y | Y | Y | Y |  |
|  |  | CGI_10012415 | Y | Y | Y | Y |  |
|  |  | CGI_10013502 | Y | N | N | N |  |
|  |  | CGI_10013675 | Y | Y | Y | Y |  |
|  |  | CGI_10013829 | Y | Y | Y | Y |  |
|  |  | CGI_10014022 | N | Y | Y | Y |  |
|  |  | CGI_10014025 | N | N | N | N |  |
|  |  | CGI_10014178 | Y | Y | Y | Y |  |
|  |  | CGI_10014269 | Y | Y | N | N |  |
|  |  | CGI_10014317 | N | Y | Y | Y |  |
|  |  | CGI_10014318 | N | Y | Y | Y |  |
|  |  | CGI_10014355 | N | N | N | Y |  |
|  |  | CGI_10014356 | N | N | Y | Y |  |
|  |  | CGI_10014357 | Y | Y | N | Y |  |
|  |  | CGI_10014359 | Y | Y | N | Y |  |
|  |  | CGI_10014360 | N | N | N | Y |  |
|  |  | CGI_10014386 | N | Y | N | Y |  |
|  |  | CGI_10014490 | Y | Y | Y | Y |  |
|  |  | CGI_10014499 | N | Y | Y | Y |  |
|  |  | CGI_10014500 | N | Y | Y | Y |  |
|  |  | CGI_10014735 | Y | N | N | Y |  |
|  |  | CGI_10015541 | N | N | N | N |  |
|  |  | CGI_10015542 | N | N | N | N |  |
|  |  | CGI_10015723 | Y | Y | N | N |  |
|  |  | CGI_10016242 | Y | Y | N | N |  |
|  |  | CGI_10016296 | N | Y | Y | Y |  |
|  |  | CGI_10016586 | N | Y | Y | Y |  |
|  |  | CGI_10016587 | N | Y | N | Y |  |
|  |  | CGI_10016599 | N | Y | Y | Y |  |
|  |  | CGI_10016796 | N | Y | N | Y |  |
|  |  | CGI_10016797 | Y | Y | Y | Y |  |
|  |  | CGI_10017787 | N | Y | Y | Y |  |
|  |  | CGI_10017800 | Y | Y | Y | Y |  |
|  |  | CGI_10018026 | N | Y | Y | Y |  |
|  |  | CGI_10018027 | Y | Y | Y | Y |  |
|  |  | CGI_10018207 | Y | Y | Y | Y |  |
|  |  | CGI_10018443 | N | Y | Y | Y |  |
|  |  | CGI_10018449 | Y | Y | Y | Y |  |
|  |  | CGI_10018687 | N | Y | Y | Y |  |
|  |  | CGI_10018708 | Y | Y | Y | Y |  |
|  |  | CGI_10018709 | Y | Y | Y | Y |  |
|  |  | CGI_10019058 | Y | N | N | Y |  |
|  |  | CGI_10019059 | Y | Y | Y | Y |  |
|  |  | CGI_10019304 | N | N | N | N |  |
|  |  | CGI_10019702 | Y | Y | Y | Y |  |
|  |  | CGI_10020017 | N | Y | Y | Y |  |
|  |  | CGI_10020018 | Y | Y | Y | Y |  |
|  |  | CGI_10020135 | Y | Y | Y | Y |  |
|  |  | CGI_10020148 | Y | Y | N | N |  |
|  |  | CGI_10020726 | Y | N | N | N |  |
|  |  | CGI_10020880 | N | Y | N | N |  |
|  |  | CGI_10020881 | N | Y | Y | Y |  |
|  |  | CGI_10021064 | Y | Y | N | Y |  |
|  |  | CGI_10021065 | N | N | N | N |  |
|  |  | CGI_10021796 | N | N | N | Y |  |
|  |  | CGI_10022261 | Y | Y | Y | Y |  |
|  |  | CGI_10022335 | Y | Y | Y | Y |  |
|  |  | CGI_10022336 | N | Y | Y | Y |  |
|  |  | CGI_10022338 | Y | Y | Y | Y |  |
|  |  | CGI_10022340 | Y | Y | Y | Y |  |
|  |  | CGI_10022835 | Y | Y | Y | Y |  |
|  |  | CGI_10022836 | N | Y | Y | Y |  |
|  |  | CGI_10022838 | N | Y | Y | Y |  |
|  |  | CGI_10023246 | Y | Y | Y | Y |  |
|  |  | CGI_10023308 | Y | N | N | Y |  |
|  |  | CGI_10024207 | N | N | N | N |  |
|  |  | CGI_10024348 | Y | Y | N | N |  |
|  |  | CGI_10024436 | Y | Y | Y | N |  |
|  |  | CGI_10024602 | Y | Y | Y | Y |  |
|  |  | CGI_10024663 | N | N | N | N |  |
|  |  | CGI_10025473 | N | Y | Y | Y |  |
|  |  | CGI_10026200 | N | Y | Y | Y |  |
|  |  | CGI_10026201 | N | N | Y | Y |  |
|  |  | CGI_10026202 | N | N | N | N |  |
|  |  | CGI_10026334 | N | N | N | N |  |
|  |  | CGI_10026877 | N | N | N | N |  |
|  |  | CGI_10026878 | N | Y | Y | Y |  |
|  |  | CGI_10026879 | N | Y | Y | Y |  |
|  |  | CGI_10026880 | N | N | Y | N |  |
|  |  | CGI_10026881 | N | Y | Y | Y |  |
|  |  | CGI_10026882 | N | Y | Y | N |  |
|  |  | CGI_10026883 | N | Y | Y | Y |  |
|  |  | CGI_10026884 | N | Y | Y | Y |  |
|  |  | CGI_10026885 | N | Y | Y | Y |  |
|  |  | CGI_10026886 | N | Y | Y | Y |  |
|  |  | CGI_10027271 | Y | N | N | N |  |
|  |  | CGI_10027545 | N | Y | Y | Y |  |
|  |  | CGI_10027636 | N | N | N | N |  |
|  |  | CGI_10027637 | N | N | N | N |  |
|  |  | CGI_10027833 | Y | Y | Y | Y |  |
|  |  | CGI_10028317 | N | Y | Y | Y |  |
|  |  | CGI_10028579 | Y | N | N | Y |  |
|  |  | CGI_10028906 | Y | Y | Y | Y |  |
|  |  | CGI_10028907 | Y | Y | Y | Y |  |
|  |  | CGI_10028910 | N | Y | Y | Y |  |
| 1.A4 Immune receptors-LRR-domain containing protein | | |  |  |  |  |  |
| Proteins with leucine-rich repeat and immunoglobulin domains (LRR-IG) | 11 | CGI_10008003 | Y | Y | N | N |  |
|  |  | CGI_10008004 | Y | Y | N | N |  |
|  |  | CGI_10008005 | N | Y | Y | N |  |
|  |  | CGI_10008006 | Y | Y | N | N |  |
|  |  | CGI_10015472 | Y | Y | Y | Y |  |
|  |  | CGI_10016215 | Y | Y | Y | Y |  |
|  |  | CGI_10018263 | Y | Y | Y | Y |  |
|  |  | CGI_10018712 | Y | Y | Y | Y |  |
|  |  | CGI_10022637 | Y | Y | Y | Y |  |
|  |  | CGI_10027763 | Y | Y | Y | Y |  |
|  |  | CGI_10028288 | Y | Y | Y | Y |  |
| Other LRR-containing protein (LRR other) | 168 | CGI_10000248 | Y | Y | N | Y |  |
|  |  | CGI_10001725 | N | N | N | N |  |
|  |  | CGI_10002293 | Y | Y | Y | Y |  |
|  |  | CGI_10002465 | Y | Y | Y | Y |  |
|  |  | CGI_10002587 | Y | Y | Y | Y |  |
|  |  | CGI_10002664 | Y | Y | Y | Y |  |
|  |  | CGI_10002699 | Y | Y | Y | Y |  |
|  |  | CGI_10002835 | Y | Y | Y | Y |  |
|  |  | CGI_10003222 | Y | Y | Y | Y |  |
|  |  | CGI_10003223 | Y | Y | Y | Y |  |
|  |  | CGI_10003468 | Y | Y | Y | Y |  |
|  |  | CGI_10003556 | Y | Y | Y | Y |  |
|  |  | CGI_10003864 | Y | Y | Y | Y |  |
|  |  | CGI_10004294 | Y | Y | Y | Y |  |
|  |  | CGI_10004829 | Y | Y | Y | Y |  |
|  |  | CGI_10005036 | Y | Y | Y | Y |  |
|  |  | CGI_10005324 | Y | Y | Y | Y |  |
|  |  | CGI_10005379 | Y | Y | Y | Y |  |
|  |  | CGI_10005485 | Y | Y | Y | Y |  |
|  |  | CGI_10005586 | Y | Y | Y | Y |  |
|  |  | CGI_10005776 | Y | Y | Y | Y |  |
|  |  | CGI_10005777 | Y | Y | Y | Y |  |
|  |  | CGI_10005997 | Y | Y | Y | Y |  |
|  |  | CGI_10006059 | Y | Y | Y | Y |  |
|  |  | CGI_10006069 | Y | Y | Y | Y |  |
|  |  | CGI_10006201 | Y | Y | Y | Y |  |
|  |  | CGI_10006214 | Y | Y | Y | Y |  |
|  |  | CGI_10006591 | Y | Y | Y | Y |  |
|  |  | CGI_10006653 | Y | Y | Y | Y |  |
|  |  | CGI_10006745 | Y | Y | Y | Y |  |
|  |  | CGI_10006799 | Y | Y | N | Y |  |
|  |  | CGI_10007235 | N | N | N | Y |  |
|  |  | CGI_10007253 | Y | Y | Y | Y |  |
|  |  | CGI_10007718 | Y | Y | Y | Y |  |
|  |  | CGI_10008384 | Y | Y | Y | Y |  |
|  |  | CGI_10008583 | Y | Y | Y | Y |  |
|  |  | CGI_10008876 | Y | Y | Y | Y |  |
|  |  | CGI_10009002 | Y | Y | Y | Y |  |
|  |  | CGI_10009035 | Y | Y | Y | Y |  |
|  |  | CGI_10009044 | N | Y | Y | Y |  |
|  |  | CGI_10009166 | Y | N | Y | N |  |
|  |  | CGI_10009636 | Y | Y | Y | Y |  |
|  |  | CGI_10009849 | Y | Y | Y | Y |  |
|  |  | CGI_10010010 | Y | Y | Y | Y |  |
|  |  | CGI_10010162 | Y | Y | Y | Y |  |
|  |  | CGI_10010172 | Y | N | Y | Y |  |
|  |  | CGI_10010218 | Y | Y | Y | Y |  |
|  |  | CGI_10010243 | Y | Y | Y | Y |  |
|  |  | CGI_10010342 | Y | Y | Y | Y |  |
|  |  | CGI_10010514 | Y | Y | Y | Y |  |
|  |  | CGI_10010624 | Y | Y | Y | Y |  |
|  |  | CGI_10010830 | Y | Y | Y | Y |  |
|  |  | CGI_10011071 | Y | N | Y | Y |  |
|  |  | CGI_10011146 | Y | Y | Y | Y |  |
|  |  | CGI_10011155 | Y | Y | Y | Y |  |
|  |  | CGI_10011156 | Y | Y | Y | Y |  |
|  |  | CGI_10011239 | Y | Y | Y | Y |  |
|  |  | CGI_10011574 | Y | Y | Y | Y |  |
|  |  | CGI_10011648 | Y | Y | Y | Y |  |
|  |  | CGI_10011696 | Y | Y | Y | Y |  |
|  |  | CGI_10011703 | Y | Y | Y | Y |  |
|  |  | CGI_10011831 | Y | Y | Y | Y |  |
|  |  | CGI_10012028 | Y | Y | Y | Y |  |
|  |  | CGI_10012378 | Y | Y | Y | Y |  |
|  |  | CGI_10012516 | N | Y | Y | Y |  |
|  |  | CGI_10012520 | Y | Y | Y | Y |  |
|  |  | CGI_10012818 | Y | Y | Y | Y |  |
|  |  | CGI_10012965 | Y | Y | Y | Y |  |
|  |  | CGI_10013180 | Y | N | N | N |  |
|  |  | CGI_10013238 | Y | Y | Y | Y |  |
|  |  | CGI_10013664 | Y | Y | Y | Y |  |
|  |  | CGI_10013991 | Y | Y | Y | Y |  |
|  |  | CGI_10014567 | Y | N | Y | N |  |
|  |  | CGI_10014674 | Y | Y | Y | Y |  |
|  |  | CGI_10014757 | Y | Y | Y | Y |  |
|  |  | CGI_10014871 | Y | Y | Y | Y |  |
|  |  | CGI_10015110 | Y | N | Y | N |  |
|  |  | CGI_10015401 | Y | Y | Y | Y |  |
|  |  | CGI_10015597 | Y | Y | Y | Y |  |
|  |  | CGI_10015629 | Y | Y | Y | Y |  |
|  |  | CGI_10015912 | Y | Y | Y | Y |  |
|  |  | CGI_10015924 | Y | Y | Y | Y |  |
|  |  | CGI_10015959 | Y | Y | Y | Y |  |
|  |  | CGI_10016035 | Y | Y | Y | Y |  |
|  |  | CGI_10016178 | Y | Y | Y | Y |  |
|  |  | CGI_10016213 | Y | Y | Y | Y |  |
|  |  | CGI_10016293 | Y | Y | N | N |  |
|  |  | CGI_10016294 | Y | Y | Y | Y |  |
|  |  | CGI_10016295 | Y | Y | N | N |  |
|  |  | CGI_10016625 | Y | Y | N | N |  |
|  |  | CGI_10016632 | Y | Y | Y | Y |  |
|  |  | CGI_10016633 | Y | Y | Y | Y |  |
|  |  | CGI_10016659 | Y | Y | Y | Y |  |
|  |  | CGI_10016855 | Y | Y | Y | Y |  |
|  |  | CGI_10016865 | Y | Y | Y | Y |  |
|  |  | CGI_10017082 | N | Y | N | N |  |
|  |  | CGI_10017147 | Y | Y | Y | Y |  |
|  |  | CGI_10017217 | Y | Y | Y | Y |  |
|  |  | CGI_10017225 | Y | Y | Y | Y |  |
|  |  | CGI_10017764 | Y | Y | Y | Y |  |
|  |  | CGI_10018512 | Y | Y | Y | Y |  |
|  |  | CGI_10018655 | Y | Y | Y | Y |  |
|  |  | CGI_10018795 | Y | Y | N | Y |  |
|  |  | CGI_10019148 | Y | Y | N | Y |  |
|  |  | CGI_10019547 | Y | Y | Y | Y |  |
|  |  | CGI_10019926 | Y | Y | Y | Y |  |
|  |  | CGI_10020063 | Y | Y | Y | Y |  |
|  |  | CGI_10020123 | Y | Y | Y | Y |  |
|  |  | CGI_10020175 | Y | Y | Y | N |  |
|  |  | CGI_10020177 | Y | Y | N | N |  |
|  |  | CGI_10020495 | Y | Y | N | Y |  |
|  |  | CGI_10020496 | Y | Y | Y | Y |  |
|  |  | CGI_10020498 | Y | Y | Y | Y |  |
|  |  | CGI_10020952 | Y | Y | Y | Y |  |
|  |  | CGI_10021564 | Y | Y | Y | Y |  |
|  |  | CGI_10022067 | Y | Y | Y | Y |  |
|  |  | CGI_10022069 | Y | Y | Y | Y |  |
|  |  | CGI_10022145 | Y | Y | Y | Y |  |
|  |  | CGI_10022156 | Y | Y | Y | Y |  |
|  |  | CGI_10022206 | Y | Y | Y | Y |  |
|  |  | CGI_10022208 | Y | Y | Y | Y |  |
|  |  | CGI_10022804 | Y | Y | Y | Y |  |
|  |  | CGI_10022862 | Y | N | N | N |  |
|  |  | CGI_10022863 | Y | N | N | N |  |
|  |  | CGI_10022864 | Y | N | N | N |  |
|  |  | CGI_10022866 | Y | N | N | N |  |
|  |  | CGI_10022867 | Y | N | N | N |  |
|  |  | CGI_10022885 | Y | Y | Y | Y |  |
|  |  | CGI_10022982 | Y | Y | Y | Y |  |
|  |  | CGI_10023242 | Y | Y | Y | Y |  |
|  |  | CGI_10023351 | Y | Y | Y | Y |  |
|  |  | CGI_10023628 | Y | Y | N | N |  |
|  |  | CGI_10023776 | Y | Y | Y | Y |  |
|  |  | CGI_10023782 | Y | Y | Y | Y |  |
|  |  | CGI_10023826 | Y | N | N | N |  |
|  |  | CGI_10023829 | Y | Y | N | Y |  |
|  |  | CGI_10023874 | Y | Y | Y | Y |  |
|  |  | CGI_10023981 | Y | Y | Y | Y |  |
|  |  | CGI_10024078 | Y | Y | Y | Y |  |
|  |  | CGI_10024120 | Y | Y | Y | Y |  |
|  |  | CGI_10024376 | Y | Y | Y | Y |  |
|  |  | CGI_10024490 | Y | Y | Y | Y |  |
|  |  | CGI_10024685 | Y | N | N | N |  |
|  |  | CGI_10024852 | Y | Y | Y | Y |  |
|  |  | CGI_10025025 | Y | Y | Y | Y |  |
|  |  | CGI_10025394 | Y | Y | Y | Y |  |
|  |  | CGI_10025426 | Y | Y | Y | Y |  |
|  |  | CGI_10025655 | Y | Y | Y | Y |  |
|  |  | CGI_10025673 | Y | Y | Y | Y |  |
|  |  | CGI_10025690 | Y | Y | Y | Y |  |
|  |  | CGI_10025736 | Y | Y | Y | Y |  |
|  |  | CGI_10025832 | Y | Y | Y | Y |  |
|  |  | CGI_10026203 | Y | Y | Y | Y |  |
|  |  | CGI_10026208 | Y | Y | Y | Y |  |
|  |  | CGI_10026406 | N | Y | N | N |  |
|  |  | CGI_10026610 | Y | Y | Y | Y |  |
|  |  | CGI_10026757 | N | Y | Y | Y |  |
|  |  | CGI_10026788 | Y | Y | Y | Y |  |
|  |  | CGI_10027278 | Y | Y | Y | Y |  |
|  |  | CGI_10027412 | Y | Y | Y | Y |  |
|  |  | CGI_10027663 | Y | Y | Y | Y |  |
|  |  | CGI_10028054 | Y | Y | N | Y |  |
|  |  | CGI_10028069 | Y | Y | Y | Y |  |
|  |  | CGI_10028131 | Y | Y | Y | Y |  |
|  |  | CGI_10028416 | Y | Y | Y | Y |  |
|  |  | CGI_10028572 | Y | Y | Y | Y |  |
|  |  | CGI_10028811 | Y | Y | Y | Y |  |
|  |  | CGI_10028924 | Y | Y | Y | Y |  |
| 1.A5 Gene associated with VDJ recombination | | | | | | | |
| V(D)J recombination-activating protein 1-like ( RAG1-like)  2 | | RAG1-like1 | N | N | N | N |  |
|  |  | RAG1-like2 | N | N | N | N |  |
| Genes for which no models with evident homology or similar domain structure could be found using the search criteria detailed in Materials and Methods: RAG1/2. | | | | | | | |
| 1.B1 Effector Genes-Complement | | | | | | | |
| complement component 3/4/5 (C3/4/5) | 1 | CGI_10014037 | Y | Y | Y | Y |  |
| CD109 antigene (CD109) | 1 | CGI_10023706 | Y | Y | Y | Y |  |
| thioester containing protein (thioester) | 3 | CGI_10003729 | Y | Y | Y | Y |  |
|  |  | CGI_10004826 | Y | Y | Y | Y |  |
|  |  | CGI_10015501 | Y | Y | Y | Y |  |
| alpha-2-macroglobulin (α-2M) | 4 | CGI_10006874 | Y | Y | Y | Y |  |
|  |  | CGI_10013417 | Y | Y | Y | Y |  |
|  |  | CGI_10023765 | Y | Y | Y | Y |  |
|  |  | CGI_10026294 | Y | Y | Y | Y |  |
| Factor B-like (Bf) | 1 | CGI_10004149 | N | Y | Y | Y |  |
| Mannose Binding Protein (MBP) | 1 | CGI_10027962 | N | Y | N | N |  |
| C1q-like | 1 | CGI_10016559 | Y | Y | Y | N |  |
| globular head C1q domain containing protein (C1qDC) | 320 | CGI_10000648 | N | N | N | N |  |
|  |  | CGI_10000824 | Y | N | N | N |  |
|  |  | CGI_10000983R1 | Y | Y | Y | Y |  |
|  |  | CGI_10000983R2 | Y | Y | Y | Y |  |
|  |  | CGI_10001216 | N | Y | Y | Y |  |
|  |  | CGI_10001514 | Y | N | Y | Y |  |
|  |  | CGI_10001781 | N | Y | Y | Y |  |
|  |  | CGI_10002966 | N | N | N | N |  |
|  |  | CGI_10004133 | N | Y | Y | Y |  |
|  |  | CGI_10004134 | N | Y | Y | Y |  |
|  |  | CGI_10006380 | Y | Y | N | Y |  |
|  |  | CGI_10007098 | Y | Y | Y | Y |  |
|  |  | CGI_10007374 | Y | Y | N | N |  |
|  |  | CGI_10008379 | Y | Y | Y | Y |  |
|  |  | CGI_10008438 | Y | Y | Y | Y |  |
|  |  | CGI_10009269 | Y | Y | Y | Y |  |
|  |  | CGI_10011385 | N | Y | Y | Y |  |
|  |  | CGI_10011986 | Y | Y | Y | Y |  |
|  |  | CGI_10015042 | N | Y | Y | Y |  |
|  |  | CGI_10015043 | N | Y | Y | Y |  |
|  |  | CGI_10015259 | Y | Y | Y | Y |  |
|  |  | CGI_10015440 | Y | Y | Y | Y |  |
|  |  | CGI_10015754 | Y | Y | Y | Y |  |
|  |  | CGI_10016021 | N | Y | Y | Y |  |
|  |  | CGI_10017058 | Y | Y | N | N |  |
|  |  | CGI_10018405 | N | Y | Y | Y |  |
|  |  | CGI_10018409 | N | N | Y | Y |  |
|  |  | CGI_10018638 | Y | Y | Y | Y |  |
|  |  | CGI_10018846 | N | Y | Y | Y |  |
|  |  | CGI_10019601 | Y | Y | Y | Y |  |
|  |  | CGI_10020667 | Y | Y | Y | Y |  |
|  |  | CGI_10020809 | Y | Y | Y | Y |  |
|  |  | CGI_10021714 | Y | Y | Y | Y |  |
|  |  | CGI_10022590 | Y | Y | Y | Y |  |
|  |  | CGI_10023473 | Y | Y | Y | Y |  |
|  |  | CGI_10023483 | Y | Y | Y | Y |  |
|  |  | CGI_10024878 | Y | Y | Y | Y |  |
|  |  | CGI_10026374 | Y | Y | Y | Y |  |
|  |  | CGI_10028064 | N | Y | N | Y |  |
|  |  | CGI_10028151 | Y | Y | N | N |  |
|  |  | CGI_10000910 | Y | Y | Y | Y |  |
|  |  | CGI_10002247 | Y | Y | Y | Y |  |
|  |  | CGI_10002309 | Y | Y | N | N |  |
|  |  | CGI_10002906 | Y | Y | Y | Y |  |
|  |  | CGI_10003000 | N | Y | Y | Y |  |
|  |  | CGI_10003367 | Y | Y | Y | Y |  |
|  |  | CGI_10004134 | N | Y | Y | Y |  |
|  |  | CGI_10004651 | Y | Y | Y | Y |  |
|  |  | CGI_10006377 | Y | N | N | N |  |
|  |  | CGI_10006829 | N | Y | Y | Y |  |
|  |  | CGI_10007199 | N | Y | Y | Y |  |
|  |  | CGI_10007376 | Y | Y | Y | Y |  |
|  |  | CGI_10007585 | Y | Y | Y | Y |  |
|  |  | CGI_10008112 | Y | Y | N | N |  |
|  |  | CGI_10008113 | N | Y | N | N |  |
|  |  | CGI_10008114 | Y | Y | N | N |  |
|  |  | CGI_10008121 | N | Y | Y | Y |  |
|  |  | CGI_10009076 | Y | Y | Y | Y |  |
|  |  | CGI_10009242 | Y | Y | Y | Y |  |
|  |  | CGI_10010262 | Y | Y | Y | Y |  |
|  |  | CGI_10010437 | Y | Y | Y | Y |  |
|  |  | CGI_10011387 | Y | Y | Y | Y |  |
|  |  | CGI_10015763 | Y | Y | N | N |  |
|  |  | CGI_10015859 | Y | Y | Y | Y |  |
|  |  | CGI_10017851 | N | Y | Y | Y |  |
|  |  | CGI_10019357 | N | Y | Y | Y |  |
|  |  | CGI_10020314 | Y | Y | Y | Y |  |
|  |  | CGI_10022439 | Y | Y | Y | Y |  |
|  |  | CGI_10022592 | N | Y | Y | Y |  |
|  |  | CGI_10022633 | N | Y | Y | Y |  |
|  |  | CGI_10023014 | Y | Y | N | N |  |
|  |  | CGI_10026242 | Y | Y | N | Y |  |
|  |  | CGI_10000823 | N | N | N | Y |  |
|  |  | CGI_10000950 | N | N | N | N |  |
|  |  | CGI_10001215 | Y | Y | Y | Y |  |
|  |  | CGI_10001428 | N | Y | Y | Y |  |
|  |  | CGI_10001435 | N | Y | Y | Y |  |
|  |  | CGI_10001455 | N | Y | Y | Y |  |
|  |  | CGI_10001534 | N | N | N | N |  |
|  |  | CGI_10001557 | N | N | Y | Y |  |
|  |  | CGI_10001779 | N | Y | Y | Y |  |
|  |  | CGI_10001805 | N | Y | N | N |  |
|  |  | CGI_10001815 | Y | Y | N | N |  |
|  |  | CGI_10001861 | Y | Y | Y | Y |  |
|  |  | CGI_10002101 | Y | Y | Y | Y |  |
|  |  | CGI_10002102 | Y | Y | Y | Y |  |
|  |  | CGI_10002141 | N | Y | Y | Y |  |
|  |  | CGI_10002235 | Y | N | Y | Y |  |
|  |  | CGI_10002389 | N | Y | Y | Y |  |
|  |  | CGI_10002615 | N | Y | Y | N |  |
|  |  | CGI_10002674 | Y | Y | N | Y |  |
|  |  | CGI_10002721 | Y | N | Y | Y |  |
|  |  | CGI_10002821 | Y | Y | N | N |  |
|  |  | CGI_10002907 | Y | Y | Y | Y |  |
|  |  | CGI_10003166 | Y | N | N | N |  |
|  |  | CGI_10003934 | N | Y | Y | Y |  |
|  |  | CGI_10003957 | N | N | Y | Y |  |
|  |  | CGI_10004135 | Y | Y | Y | Y |  |
|  |  | CGI_10004177 | Y | Y | N | N |  |
|  |  | CGI_10004625 | Y | Y | N | N |  |
|  |  | CGI_10004653 | Y | Y | Y | Y |  |
|  |  | CGI_10004907 | Y | N | Y | N |  |
|  |  | CGI_10004908 | N | N | N | N |  |
|  |  | CGI_10005027 | Y | Y | N | N |  |
|  |  | CGI_10005029 | Y | Y | N | Y |  |
|  |  | CGI_10005030 | Y | Y | Y | Y |  |
|  |  | CGI_10005031 | Y | Y | N | Y |  |
|  |  | CGI_10005032 | Y | Y | N | Y |  |
|  |  | CGI_10005135 | Y | Y | N | Y |  |
|  |  | CGI_10005220 | N | Y | Y | Y |  |
|  |  | CGI_10005221 | N | Y | Y | Y |  |
|  |  | CGI_10005418 | Y | Y | N | Y |  |
|  |  | CGI_10005419 | N | Y | Y | Y |  |
|  |  | CGI_10005427 | Y | Y | N | N |  |
|  |  | CGI_10005428 | Y | Y | N | N |  |
|  |  | CGI_10005429 | Y | Y | N | N |  |
|  |  | CGI_10006058 | Y | Y | Y | Y |  |
|  |  | CGI_10006548 | Y | Y | Y | Y |  |
|  |  | CGI_10006725 | Y | Y | N | N |  |
|  |  | CGI_10006726 | Y | Y | N | Y |  |
|  |  | CGI_10007015 | N | N | Y | Y |  |
|  |  | CGI_10007278 | Y | Y | Y | Y |  |
|  |  | CGI_10007769 | Y | Y | N | N |  |
|  |  | CGI_10007808 | Y | Y | N | N |  |
|  |  | CGI_10007882 | N | Y | Y | Y |  |
|  |  | CGI_10007994 | N | Y | N | N |  |
|  |  | CGI_10008115 | N | Y | N | N |  |
|  |  | CGI_10008220 | Y | Y | Y | Y |  |
|  |  | CGI_10008239 | Y | Y | Y | Y |  |
|  |  | CGI_10008380 | Y | Y | Y | Y |  |
|  |  | CGI_10008604 | Y | Y | N | N |  |
|  |  | CGI_10008906 | Y | Y | N | Y |  |
|  |  | CGI_10008908 | Y | Y | N | N |  |
|  |  | CGI_10009046 | Y | Y | N | N |  |
|  |  | CGI_10009047 | N | Y | Y | Y |  |
|  |  | CGI_10009048 | N | Y | Y | Y |  |
|  |  | CGI_10009049 | N | Y | Y | Y |  |
|  |  | CGI_10009270 | N | Y | Y | Y |  |
|  |  | CGI_10009271 | N | Y | Y | Y |  |
|  |  | CGI_10009273 | N | Y | Y | Y |  |
|  |  | CGI_10009274 | N | Y | Y | Y |  |
|  |  | CGI_10009275 | N | Y | Y | Y |  |
|  |  | CGI_10009279 | Y | N | N | N |  |
|  |  | CGI_10009753 | Y | Y | Y | Y |  |
|  |  | CGI_10009789 | N | Y | Y | Y |  |
|  |  | CGI_10009872 | Y | Y | Y | Y |  |
|  |  | CGI_10009984 | Y | Y | Y | Y |  |
|  |  | CGI_10009985 | Y | Y | Y | N |  |
|  |  | CGI_10010226 | N | N | N | N |  |
|  |  | CGI_10010227 | Y | Y | N | N |  |
|  |  | CGI_10010229 | Y | Y | Y | N |  |
|  |  | CGI_10010506 | Y | Y | Y | Y |  |
|  |  | CGI_10010590 | N | N | Y | Y |  |
|  |  | CGI_10010996 | Y | Y | Y | Y |  |
|  |  | CGI_10010998 | Y | N | N | N |  |
|  |  | CGI_10010999 | Y | Y | Y | Y |  |
|  |  | CGI_10011137 | Y | Y | N | N |  |
|  |  | CGI_10011342 | N | Y | Y | Y |  |
|  |  | CGI_10011386 | Y | Y | Y | Y |  |
|  |  | CGI_10011450 | Y | Y | N | N |  |
|  |  | CGI_10011621 | Y | Y | N | N |  |
|  |  | CGI_10011639 | Y | Y | Y | Y |  |
|  |  | CGI_10011836 | Y | Y | Y | Y |  |
|  |  | CGI_10011837 | Y | Y | Y | Y |  |
|  |  | CGI_10011909 | Y | Y | Y | Y |  |
|  |  | CGI_10011923 | Y | Y | Y | Y |  |
|  |  | CGI_10012045 | N | Y | Y | Y |  |
|  |  | CGI_10012046 | N | Y | Y | Y |  |
|  |  | CGI_10012047 | Y | Y | Y | Y |  |
|  |  | CGI_10012232 | Y | Y | Y | Y |  |
|  |  | CGI_10012233 | Y | Y | Y | Y |  |
|  |  | CGI_10012320 | N | Y | Y | Y |  |
|  |  | CGI_10012321 | N | Y | Y | Y |  |
|  |  | CGI_10012699 | N | Y | Y | Y |  |
|  |  | CGI_10012721 | Y | Y | N | N |  |
|  |  | CGI_10012832 | Y | Y | Y | Y |  |
|  |  | CGI_10012844 | N | N | N | Y |  |
|  |  | CGI_10012888 | Y | N | N | N |  |
|  |  | CGI_10012889 | N | Y | N | N |  |
|  |  | CGI_10012890 | Y | Y | N | N |  |
|  |  | CGI_10013066 | N | Y | Y | Y |  |
|  |  | CGI_10013069 | Y | N | Y | Y |  |
|  |  | CGI_10013070 | Y | Y | Y | Y |  |
|  |  | CGI_10013071 | N | Y | Y | Y |  |
|  |  | CGI_10013072 | Y | N | Y | Y |  |
|  |  | CGI_10013073 | Y | Y | Y | Y |  |
|  |  | CGI_10013136 | Y | Y | N | N |  |
|  |  | CGI_10013138 | Y | Y | N | Y |  |
|  |  | CGI_10013361 | N | Y | Y | Y |  |
|  |  | CGI_10013362 | Y | Y | Y | Y |  |
|  |  | CGI_10013788 | N | N | Y | N |  |
|  |  | CGI_10014277 | Y | Y | Y | Y |  |
|  |  | CGI_10014614 | Y | Y | Y | Y |  |
|  |  | CGI_10014672 | N | N | N | N |  |
|  |  | CGI_10014673 | N | N | N | N |  |
|  |  | CGI_10014944 | N | Y | Y | Y |  |
|  |  | CGI_10014945 | N | Y | Y | Y |  |
|  |  | CGI_10015041 | N | Y | Y | Y |  |
|  |  | CGI_10015044 | N | Y | Y | Y |  |
|  |  | CGI_10015052 | N | Y | N | Y |  |
|  |  | CGI_10015346 | Y | Y | Y | Y |  |
|  |  | CGI_10015347 | N | Y | N | N |  |
|  |  | CGI_10015348 | Y | Y | N | N |  |
|  |  | CGI_10015544 | N | Y | Y | Y |  |
|  |  | CGI_10015610 | Y | Y | N | N |  |
|  |  | CGI_10015625 | N | Y | N | N |  |
|  |  | CGI_10015755 | N | Y | Y | Y |  |
|  |  | CGI_10015762 | Y | Y | N | N |  |
|  |  | CGI_10015852 | Y | Y | N | N |  |
|  |  | CGI_10015853 | Y | Y | Y | N |  |
|  |  | CGI_10016005 | N | N | N | Y |  |
|  |  | CGI_10016022 | N | Y | Y | Y |  |
|  |  | CGI_10016023 | N | Y | Y | Y |  |
|  |  | CGI_10016092 | N | Y | N | N |  |
|  |  | CGI_10016309 | N | Y | Y | Y |  |
|  |  | CGI_10016318 | Y | Y | N | N |  |
|  |  | CGI_10016348 | Y | Y | Y | Y |  |
|  |  | CGI_10016349 | Y | Y | Y | Y |  |
|  |  | CGI_10016350 | Y | Y | Y | Y |  |
|  |  | CGI_10016352 | Y | Y | Y | Y |  |
|  |  | CGI_10016353 | Y | Y | Y | Y |  |
|  |  | CGI_10016361 | N | Y | Y | Y |  |
|  |  | CGI_10016362 | Y | Y | Y | Y |  |
|  |  | CGI_10016424 | N | Y | Y | Y |  |
|  |  | CGI_10016713 | Y | N | N | N |  |
|  |  | CGI_10017131 | Y | Y | Y | Y |  |
|  |  | CGI_10017364 | N | Y | Y | Y |  |
|  |  | CGI_10017436 | N | N | N | Y |  |
|  |  | CGI_10017821 | Y | Y | N | Y |  |
|  |  | CGI_10018214 | Y | Y | N | N |  |
|  |  | CGI_10018355 | N | Y | Y | Y |  |
|  |  | CGI_10018356 | N | N | Y | Y |  |
|  |  | CGI_10018411 | N | N | Y | Y |  |
|  |  | CGI_10018639 | Y | Y | Y | Y |  |
|  |  | CGI_10018642 | Y | Y | Y | Y |  |
|  |  | CGI_10018692 | N | N | Y | Y |  |
|  |  | CGI_10018780 | Y | Y | N | Y |  |
|  |  | CGI_10018974 | N | N | Y | Y |  |
|  |  | CGI_10019023 | Y | Y | N | N |  |
|  |  | CGI_10019070 | Y | Y | Y | Y |  |
|  |  | CGI_10019278 | Y | N | N | N |  |
|  |  | CGI_10020461 | N | N | Y | Y |  |
|  |  | CGI_10020464 | N | Y | Y | Y |  |
|  |  | CGI_10020567 | Y | Y | Y | Y |  |
|  |  | CGI_10020666 | N | Y | Y | Y |  |
|  |  | CGI_10020715 | Y | Y | N | Y |  |
|  |  | CGI_10021047 | Y | Y | N | N |  |
|  |  | CGI_10021048 | Y | Y | N | Y |  |
|  |  | CGI_10021050 | Y | Y | Y | Y |  |
|  |  | CGI_10021051 | Y | Y | N | Y |  |
|  |  | CGI_10021405 | Y | N | Y | Y |  |
|  |  | CGI_10021449 | Y | Y | N | N |  |
|  |  | CGI_10021488 | N | Y | Y | Y |  |
|  |  | CGI_10021489 | Y | Y | N | Y |  |
|  |  | CGI_10021493 | Y | Y | Y | Y |  |
|  |  | CGI_10021742 | N | Y | Y | Y |  |
|  |  | CGI_10021869 | Y | Y | Y | N |  |
|  |  | CGI_10022054 | Y | Y | N | Y |  |
|  |  | CGI_10022091 | Y | Y | N | Y |  |
|  |  | CGI_10022092 | Y | Y | N | N |  |
|  |  | CGI_10022405 | N | Y | Y | Y |  |
|  |  | CGI_10022407 | Y | Y | Y | Y |  |
|  |  | CGI_10022591 | Y | Y | Y | Y |  |
|  |  | CGI_10022632 | N | Y | N | Y |  |
|  |  | CGI_10022634 | Y | Y | N | N |  |
|  |  | CGI_10022875 | N | N | Y | Y |  |
|  |  | CGI_10022877 | N | Y | Y | Y |  |
|  |  | CGI_10023012 | N | Y | N | N |  |
|  |  | CGI_10023013 | Y | N | N | N |  |
|  |  | CGI_10023015 | N | Y | N | N |  |
|  |  | CGI_10023202 | Y | Y | Y | N |  |
|  |  | CGI_10023214 | Y | Y | N | Y |  |
|  |  | CGI_10023215 | Y | Y | N | N |  |
|  |  | CGI_10023216 | N | Y | Y | N |  |
|  |  | CGI_10023217 | Y | Y | N | N |  |
|  |  | CGI_10023843 | Y | Y | N | N |  |
|  |  | CGI_10023844 | Y | Y | N | N |  |
|  |  | CGI_10023845 | Y | Y | N | N |  |
|  |  | CGI_10023888 | Y | Y | N | N |  |
|  |  | CGI_10023889 | Y | Y | Y | Y |  |
|  |  | CGI_10024034 | N | Y | Y | Y |  |
|  |  | CGI_10024230 | Y | Y | Y | Y |  |
|  |  | CGI_10024231 | Y | Y | Y | Y |  |
|  |  | CGI_10024232 | Y | Y | Y | Y |  |
|  |  | CGI_10024521 | Y | Y | N | N |  |
|  |  | CGI_10024522 | N | Y | N | Y |  |
|  |  | CGI_10024533 | Y | N | Y | Y |  |
|  |  | CGI_10024595 | N | Y | Y | N |  |
|  |  | CGI_10024596 | Y | N | Y | Y |  |
|  |  | CGI_10024597 | Y | Y | Y | Y |  |
|  |  | CGI_10024606 | N | Y | Y | Y |  |
|  |  | CGI_10024879 | N | Y | Y | Y |  |
|  |  | CGI_10025046 | N | Y | N | N |  |
|  |  | CGI_10025120 | N | N | N | Y |  |
|  |  | CGI_10025141 | Y | Y | N | N |  |
|  |  | CGI_10025495 | N | Y | Y | Y |  |
|  |  | CGI_10025581 | N | Y | Y | Y |  |
|  |  | CGI_10025591 | Y | Y | N | N |  |
|  |  | CGI_10025601 | Y | Y | N | Y |  |
|  |  | CGI_10026199 | Y | Y | Y | Y |  |
|  |  | CGI_10026375 | N | Y | Y | Y |  |
|  |  | CGI_10026376 | N | Y | Y | Y |  |
|  |  | CGI_10026377 | Y | Y | Y | Y |  |
|  |  | CGI_10026379 | N | Y | Y | Y |  |
|  |  | CGI_10026716 | Y | N | N | Y |  |
|  |  | CGI_10026769 | N | Y | Y | Y |  |
|  |  | CGI_10026873 | N | Y | Y | Y |  |
|  |  | CGI_10026904 | Y | N | Y | Y |  |
|  |  | CGI_10026905 | Y | Y | Y | Y |  |
|  |  | CGI_10026907 | Y | Y | Y | Y |  |
|  |  | CGI_10027019 | N | Y | Y | Y |  |
|  |  | CGI_10027984 | N | Y | Y | Y |  |
|  |  | CGI_10027985 | N | Y | Y | Y |  |
|  |  | CGI_10028067 | N | Y | Y | Y |  |
|  |  | CGI_10028107 | Y | N | N | Y |  |
|  |  | CGI_10028108 | Y | Y | N | N |  |
|  |  | CGI_10028109 | Y | Y | N | N |  |
|  |  | CGI_10028110 | Y | Y | N | Y |  |
|  |  | CGI_10028187 | Y | Y | N | Y |  |
|  |  | CGI_10028278 | N | Y | Y | Y |  |
| Genes for which no models with evident homology or similar domain structure could be found using the search criteria detailed in Materials and Methods: C1s,C1r,MASP1/2,C6-9(terminal pathway). | | | | | | | |
| 1.B2 Effector Genes-Cytolytic pathways | | |  |  |  |  |  |
| MACPF domain containing protein (Perfoin-like) | 15 | CGI_10002181 | Y | Y | Y | Y |  |
|  |  | CGI_10004798 | Y | Y | Y | Y |  |
|  |  | CGI_10008793 | N | Y | Y | Y |  |
|  |  | CGI_10008794 | N | Y | Y | Y |  |
|  |  | CGI_10011472 | N | Y | Y | Y |  |
|  |  | CGI_10012918 | Y | Y | N | Y |  |
|  |  | CGI_10020082 | N | Y | Y | Y |  |
|  |  | CGI_10020085 | Y | Y | Y | Y |  |
|  |  | CGI_10020086 | N | N | N | N |  |
|  |  | CGI_10023598 | Y | Y | Y | Y |  |
|  |  | CGI_10024486 | Y | Y | N | N |  |
|  |  | CGI_10024487 | Y | Y | N | Y |  |
|  |  | CGI_10027826 | Y | Y | Y | Y |  |
|  |  | CGI_10027827 | Y | Y | Y | Y |  |
|  |  | CGI_10027905 | Y | Y | Y | Y |  |
| 1.B3 Effector genes-Additional cytotoxic effectors and regulators | | | |  |  |  |  |
| Nitric oxide synthase (NOS) | 1 | CGI_10014826 | Y | Y | Y | Y |  |
| Peroxiredoxin (PRX) | 5 | CGI_10024227 | Y | Y | Y | Y |  |
|  |  | CGI_10014465 | Y | Y | Y | Y |  |
|  |  | CGI_10008877 | Y | Y | Y | Y |  |
|  |  | CGI_10027917 | Y | Y | Y | Y |  |
|  |  | CGI_10017617 | Y | Y | Y | Y |  |
| Catalase (CAT) | 2 | CGI_10003355 | Y | Y | Y | Y |  |
|  |  | CGI_10003354 | Y | Y | Y | Y |  |
| Superoxide dismutase (SOD) | 9 | CGI_10021362 | Y | Y | Y | Y |  |
|  |  | CGI_10021360 | Y | Y | N | Y |  |
|  |  | CGI_10017958 | Y | Y | Y | Y |  |
|  |  | CGI_10017307 | Y | Y | Y | Y |  |
|  |  | CGI_10026148 | Y | Y | Y | Y |  |
|  |  | CGI_10004092 | Y | Y | Y | Y |  |
|  |  | CGI_10018833 | Y | Y | Y | Y |  |
|  |  | CGI_10000265 | Y | Y | Y | Y |  |
|  |  | CGI_10018834 | Y | Y | Y | Y |  |
| Lysozyme | 3 | CGI_10024457 | Y | Y | Y | Y |  |
|  |  | CGI_10024455 | N | Y | Y | Y |  |
|  |  | CGI_10004145 | N | N | Y | Y |  |
| defensin | 2 | CGI_10009486 | Y | Y | N | N |  |
|  |  | CGI_10009488 | Y | Y | Y | Y |  |
| Big defensin | 1 | CGI_10007635 | N | Y | N | Y |  |
| Bactericidal permeability-increasing protein (BPI) | 3 | CGI_10021420 | Y | Y | Y | Y |  |
|  |  | CGI_10021419 | Y | Y | Y | Y |  |
|  |  | CGI_10021418 | Y | Y | Y | Y |  |
| Glutathione peroxidase (GPX) | 8 | CGI_10002435 | Y | Y | Y | Y |  |
|  |  | CGI_10005470 | Y | Y | Y | Y |  |
|  |  | CGI_10007023 | Y | Y | Y | Y |  |
|  |  | CGI_10007024 | Y | Y | Y | Y |  |
|  |  | CGI_10008378 | Y | Y | N | N |  |
|  |  | CGI_10017612 | Y | Y | Y | Y |  |
|  |  | CGI_10022127 | Y | N | N | N |  |
|  |  | CGI_10025106 | Y | Y | Y | Y |  |
| II. REGULATORY PATHWAYS | |  |  |  |  |  |  |
| II.A1 Intracellular signal transduction-NFκB/IRF pathway | | |  |  |  |  |  |
| Nuclear factor kappa-B (NFκB) | 4 | CGI_10018142 | Y | Y | Y | Y |  |
|  |  | CGI_10004837 | Y | Y | Y | Y |  |
|  |  | CGI_10028750 | Y | Y | Y | Y |  |
|  |  | CGI_10021567 | Y | Y | Y | Y |  |
| Inhibitor of nuclear factor kappa-B (IκB) | 4 | CGI_10020475 | Y | Y | Y | Y |  |
|  |  | CGI_10009725 | Y | Y | Y | Y |  |
|  |  | CGI_10021534 | Y | Y | Y | Y |  |
|  |  | CGI_10009849 | Y | Y | Y | Y |  |
| Serine/threonine-protein kinase TBK1 (TBK1) | 4 | CGI_10004654 | Y | Y | Y | Y |  |
|  |  | CGI_10004656 | Y | Y | Y | Y |  |
|  |  | CGI_10016954 | Y | Y | Y | Y |  |
|  |  | CGI_10012642 | Y | Y | Y | Y |  |
| TNF receptor-associated factor (TRAF) | 15 | CGI_10023158 | Y | Y | Y | Y |  |
|  |  | CGI_10010206 | Y | Y | Y | Y |  |
|  |  | CGI_10008964 | Y | Y | Y | Y |  |
|  |  | CGI_10002841 | Y | Y | Y | Y |  |
|  |  | CGI_10006109 | Y | Y | N | N |  |
|  |  | CGI_10008785 | N | N | Y | Y |  |
|  |  | CGI_10019401 | Y | Y | Y | Y |  |
|  |  | CGI_10022362 | Y | Y | Y | Y |  |
|  |  | CGI_10019401 | Y | Y | Y | Y |  |
|  |  | CGI_10027674 | Y | Y | Y | Y |  |
|  |  | CGI_10011877 | Y | Y | Y | Y |  |
|  |  | CGI_10020331 | Y | Y | Y | Y |  |
|  |  | CGI_10003859 | Y | Y | Y | Y |  |
|  |  | CGI_10027979 | Y | Y | Y | Y |  |
|  |  | CGI_10008785 | N | N | Y | Y |  |
| Stress-activated protein kinase JNK (JNK) | 1 | CGI_10020378 | Y | Y | Y | Y |  |
| Mitogen-activated protein kinase kinase kinase 7 (TAK1) | 1 | CGI_10006841 | Y | Y | Y | Y |  |
| Mitogen-activated protein kinase p38 (p38) | 1 | CGI_10004156 | Y | Y | Y | Y |  |
| Transcription factor AP-1 (AP-1) | 1 | CGI_10006579 | Y | Y | Y | Y |  |
| Interleukin-1 receptor-associated kinase (IRAK) | 3 | CGI_10018046 | Y | Y | Y | Y |  |
|  |  | CGI_10000165 | Y | Y | Y | Y |  |
|  |  | CGI_10000166 | Y | Y | Y | Y |  |
| Inhibitor of nuclear factor kappa-B kinase (IKK) | 4 | CGI_10006185 | Y | Y | Y | Y |  |
|  |  | CGI_10006186 | Y | Y | Y | Y |  |
|  |  | CGI_10001373 | Y | Y | Y | Y |  |
|  |  | CGI_10016288 | Y | Y | Y | Y |  |
| Interferon regulatory factor (IRF) | 4 | CGI_10003270 | Y | Y | Y | Y |  |
|  |  | CGI_10005133 | Y | Y | Y | Y |  |
|  |  | CGI_10021170 | Y | Y | Y | Y |  |
|  |  | CGI_10021171 | Y | Y | Y | Y |  |
| II.A2 Intracellular signal transduction-TLR adaptor moleculues | | |  |  |  |  |  |
| Myeloid differentiation primary response gene 88 (MyD88) | 6 | CGI_10007490 | Y | Y | Y | Y |  |
|  |  | CGI_10013672 | Y | Y | Y | Y |  |
|  |  | CGI_10026092 | Y | Y | Y | Y |  |
|  |  | CGI_10026099 | Y | Y | Y | Y |  |
|  |  | CGI_10026174 | Y | Y | Y | Y |  |
|  |  | CGI_10026176 | Y | Y | Y | Y |  |
| Myeloid differentiation primary response gene 88-short type (MyD88s) | 4 | CGI_10026101 | Y | Y | Y | Y |  |
|  |  | CGI_10020979 | N | Y | Y | Y |  |
|  |  | CGI_10012722 | Y | Y | Y | Y |  |
|  |  | CGI_10012725 | Y | Y | Y | Y |  |
| Proteins with armadillo (ARM) and TIR domains (ARM-TIR) | 12 | CGI_10006788 | Y | Y | Y | Y |  |
|  |  | CGI_10008306 | Y | Y | Y | Y |  |
|  |  | CGI_10008481 | Y | Y | Y | Y |  |
|  |  | CGI_10011054 | Y | Y | Y | Y |  |
|  |  | CGI_10011056 | Y | Y | Y | Y |  |
|  |  | CGI_10021687 | Y | Y | Y | Y |  |
|  |  | CGI_10021877 | Y | Y | Y | Y |  |
|  |  | CGI_10025543 | Y | Y | Y | Y |  |
|  |  | CGI_10028729 | Y | Y | Y | Y |  |
|  |  | CGI_10011056 | Y | Y | Y | Y |  |
|  |  | CGI_10021687 | Y | Y | Y | Y |  |
|  |  | CGI_10021687 | Y | Y | Y | Y |  |
| Sterile-alpha and armadillo motif-containing protein (SARM) | 5 | CGI_10016756 | Y | Y | Y | Y |  |
|  |  | CGI_10023667 | Y | Y | Y | Y |  |
|  |  | CGI_10026370 | Y | Y | Y | Y |  |
|  |  | CGI_10016757 | Y | Y | Y | Y |  |
|  |  | CGI_10016378 | Y | Y | Y | Y |  |
| Proteins with immunoglobulin (IG) and TIR domains (IG-TIR) | 1 | CGI_10003267 | Y | Y | Y | Y |  |
| Proteins with TIR and tetratricopeptide repeat (TPR) domains (TIR-TPR) | 2 | CGI_10025069 | Y | Y | Y | Y |  |
|  |  | CGI_10025070 | Y | Y | Y | Y |  |
| Proteins with epidermal growth factor (EGF) and TIR domains (EGF-TIR) | 4 | CGI_10003134 | Y | Y | Y | Y |  |
|  |  | CGI_10007705 | Y | Y | Y | Y |  |
|  |  | CGI_10023403 | Y | Y | N | Y |  |
|  |  | CGI_10026389 | Y | Y | Y | Y |  |
| Orphan TIR (OrTIR) | 57 | CGI_10019719 | Y | Y | Y | Y |  |
|  |  | CGI_10018641 | Y | Y | Y | Y |  |
|  |  | CGI_10005883 | Y | Y | Y | Y |  |
|  |  | CGI_10001792 | Y | Y | Y | Y |  |
|  |  | CGI_10000919 | Y | Y | Y | Y |  |
|  |  | CGI_10018640 | Y | Y | Y | Y |  |
|  |  | CGI_10017729 | Y | Y | Y | Y |  |
|  |  | CGI_10027512 | Y | Y | Y | Y |  |
|  |  | CGI_10024612 | Y | Y | Y | Y |  |
|  |  | CGI_10010101 | N | N | Y | Y |  |
|  |  | CGI_10016562 | N | Y | Y | Y |  |
|  |  | CGI_10016512 | N | Y | N | N |  |
|  |  | CGI_10028905 | Y | Y | Y | Y |  |
|  |  | CGI_10011483 | Y | Y | Y | Y |  |
|  |  | CGI_10019717 | N | Y | Y | Y |  |
|  |  | CGI_10028922 | Y | Y | Y | Y |  |
|  |  | CGI_10025723 | N | Y | Y | Y |  |
|  |  | CGI_10016560 | Y | Y | Y | Y |  |
|  |  | CGI_10012517 | N | Y | N | Y |  |
|  |  | CGI_10020169 | Y | Y | Y | Y |  |
|  |  | CGI_10023106 | Y | Y | N | Y |  |
|  |  | CGI_10003575 | N | Y | N | N |  |
|  |  | CGI_10021736 | N | Y | Y | Y |  |
|  |  | CGI_10003567 | N | Y | Y | Y |  |
|  |  | CGI_10028708 | N | Y | Y | N |  |
|  |  | CGI_10005641 | Y | Y | Y | Y |  |
|  |  | CGI_10027415 | N | Y | N | N |  |
|  |  | CGI_10025724 | Y | Y | Y | Y |  |
|  |  | CGI_10017728 | Y | Y | Y | Y |  |
|  |  | CGI_10008951 | N | Y | Y | Y |  |
|  |  | CGI_10017269 | Y | Y | Y | Y |  |
|  |  | CGI_10003576 | N | N | N | N |  |
|  |  | CGI_10006285 | Y | Y | Y | Y |  |
|  |  | CGI_10016561 | N | Y | Y | Y |  |
|  |  | CGI_10001346 | N | Y | Y | Y |  |
|  |  | CGI_10012514 | N | Y | Y | Y |  |
|  |  | CGI_10027414 | N | Y | Y | N |  |
|  |  | CGI_10002173 | Y | Y | Y | Y |  |
|  |  | CGI_10013586 | Y | Y | Y | Y |  |
|  |  | CGI_10028923 | Y | Y | Y | Y |  |
|  |  | CGI_10028870 | Y | N | N | Y |  |
|  |  | CGI_10014165 | N | N | Y | Y |  |
|  |  | CGI_10005193 | Y | Y | Y | Y |  |
|  |  | CGI_10017268 | Y | Y | Y | Y |  |
|  |  | CGI_10003577 | N | N | N | N |  |
|  |  | CGI_10021136 | Y | Y | Y | Y |  |
|  |  | CGI_10028039 | N | Y | Y | Y |  |
|  |  | CGI_10001854 | Y | Y | Y | Y |  |
|  |  | CGI_10021876 | Y | Y | Y | Y |  |
|  |  | CGI_10013178 | Y | Y | Y | Y |  |
|  |  | CGI_10001122 | Y | Y | Y | Y |  |
|  |  | CGI_10019716 | N | Y | N | N |  |
|  |  | CGI_10013179 | Y | Y | N | N |  |
|  |  | CGI_10021737 | Y | N | Y | Y |  |
|  |  | CGI_10006284 | Y | Y | Y | Y |  |
|  |  | CGI_10014842 | Y | Y | Y | Y |  |
|  |  | CGI_10003569 | Y | Y | Y | Y |  |
| 5 TIR-containing adaptors has been found in mammalian. They are Myd88, TIRAP, TICAM-1, TICAM-2 and SARM. Among them, TIRAP, TICAM-1, TICAM-2 has been reported to be orginated from amphioxus. | | | | | | | |
| Evolutionarily conserved signaling intermediate in Toll pathway (ECSIT) | 1 | CGI_10002845 | Y | Y | Y | Y |  |
| II.A3 Intracellular signal transduction-NLR signaling | | |  |  |  |  |  |
| II.B1 Intercellular signaling(Cytokines and Growth Factors)-Interleukins, Cytokines and Hematopoietins | | | | | | | |
| Macrophage migration inhibitory factor (MIF) | 1 | CGI_10009263 | Y | Y | Y | Y |  |
| Pellino | 1 | CGI_10024357 | Y | Y | Y | Y |  |
| Interleukin-17A (IL-17) | 8 | CGI_10004922 | Y | Y | Y | Y |  |
|  |  | CGI_10014828 | Y | Y | Y | Y |  |
|  |  | CGI_10015251 | Y | N | Y | N |  |
|  |  | CGI_10020734 | Y | N | Y | Y |  |
|  |  | CGI_10025754 | Y | Y | Y | Y |  |
|  |  | CGI_10026344 | N | N | N | N |  |
|  |  | CGI_10026592 | Y | Y | Y | Y |  |
|  |  | CGI_10027182 | N | N | Y | Y |  |
| Interleukin-17 receptor (IL17R) | 5 | CGI_10000871 | Y | Y | Y | Y |  |
|  |  | CGI_10002512 | Y | Y | Y | Y |  |
|  |  | CGI_10021486 | Y | Y | Y | Y |  |
|  |  | CGI_10027691 | Y | Y | Y | Y |  |
|  |  | CGI_10027692 | Y | Y | Y | Y |  |
| Interferons, most interleukins, chemokines | | |  |  |  |  |  |
| II.B2 Intercellular signaling (Cytokines and Growth Factors)-TNF pathway | | | |  |  |  |  |
| Tumor necrosis factor (TNF) | 19 | CGI_10001976 | Y | Y | Y | Y |  |
|  |  | CGI_10005109 | Y | Y | Y | Y |  |
|  |  | CGI_10005110 | Y | Y | Y | Y |  |
|  |  | CGI_10005949 | Y | Y | Y | Y |  |
|  |  | CGI_10006440 | Y | Y | Y | Y |  |
|  |  | CGI_10006926 | Y | Y | Y | Y |  |
|  |  | CGI_10009094 | Y | Y | Y | Y |  |
|  |  | CGI_10009096 | Y | Y | Y | Y |  |
|  |  | CGI_10009097 | Y | Y | Y | Y |  |
|  |  | CGI_10012335 | Y | Y | Y | Y |  |
|  |  | CGI_10012337 | Y | Y | Y | Y |  |
|  |  | CGI_10016225 | Y | Y | Y | Y |  |
|  |  | CGI_10016228 | Y | Y | Y | Y |  |
|  |  | CGI_10018786 | Y | Y | Y | Y |  |
|  |  | CGI_10018787 | Y | Y | Y | Y |  |
|  |  | CGI_10018788 | Y | Y | Y | Y |  |
|  |  | CGI_10020131 | Y | Y | Y | Y |  |
|  |  | CGI_10028807 | Y | Y | Y | Y |  |
|  |  | CGI_10005110R-D2 | Y | Y | Y | Y |  |
| Tumor necrosis factor receptor (TNFR) | 13 | CGI_10021621 | Y | Y | Y | Y |  |
|  |  | CGI_10003766 | Y | Y | Y | Y |  |
|  |  | CGI_10011686 | Y | Y | Y | Y |  |
|  |  | CGI_10017459 | Y | Y | Y | Y |  |
|  |  | CGI_10021907 | Y | Y | Y | Y |  |
|  |  | CGI_10013090 | Y | Y | Y | Y |  |
|  |  | CGI_10006889 | Y | Y | Y | Y |  |
|  |  | CGI_10006294 | Y | Y | Y | Y |  |
|  |  | CGI_10019984 | N | Y | Y | Y |  |
|  |  | CGI_10021624 | Y | Y | Y | Y |  |
|  |  | CGI_10002984 | Y | Y | Y | Y |  |
|  |  | CGI_10017460 | Y | Y | Y | Y |  |
|  |  | CGI_10017293 | Y | Y | Y | Y |  |
| FAS-associated death domain protein (FADD) | 1 | AEB54798 | Y | Y | Y | Y |  |
|  |  |  |  |  |  |  |  |

1. **Categorized list of *TLR* genes in the Pacific oyster**

| Cluster | Gene ID | Location | | | Domain structure by SMART | Shift | Express (RPKM>1) | | |
| --- | --- | --- | --- | --- | --- | --- | --- | --- | --- |
|  |  | Scaffold ID | Start | End |  |  | Tiss | Dev | Imm |
| V type (V) | CGI_10027513_R | scaffold501 | 289314 | 292004 | SP-NT-LRR(18)-CT-TM-TIR | 0 | Y | N | Y |
|  | CGI_10027513_R-D3* | scaffold408 | 479785 | 482409 | SP-NT-LRR(19)-CT-TM-TIR | 0 | Y | N | Y |
|  | CGI_10027513_R-D2* | scaffold501 | 279239 | 281878 | SP-NT-LRR(18)-CT-TM-TIR | 0 | Y | Y | Y |
|  | CGI_10027513_R-D4* | scaffold501 | 297721 | 300287 | SP-NT-LRR(18)-CT-TM-TIR | 0 | Y | N | Y |
|  | CGI_10027513_R-D5* | scaffold501 | 293401 | 296001 | SP-NT-LRR(18)-CT-TM-TIR | 0 | Y | N | Y |
| P type (P) | HQ174217 | scaffold750 | 61129 | 65081 | SP-LRR(15)-NT-LRR(4)-TM-TIR | 0 | Y | Y | Y |
|  | CGI_10008223 | scaffold42948 | 42228 | 46561 | SP-LRR(20)-NT-LRR(3)-CT-TM | 0 | Y | Y | Y |
| Short P type Group I  (sP-Group I) | CGI_10005194-D7* | scaffold780 | 170037 | 172310 | SP-LRR(4)-TM-TIR | 0 | Y | N | N |
|  | CGI_10005194-D19* | scaffold1641 | 288127 | 290257 | LRR(5)-TM-TIR | 2 | Y | N | N |
|  | CGI_10026493-D9* | scaffold1641 | 293368 | 295535 | SP-LRR(6)-CT-TM-TIR | 1 | Y | N | Y |
|  | CGI_10005194-D29* | scaffold1641 | 303725 | 307344 | SP-NT-LRR(7)-TM-TIR | 0 | Y | Y | Y |
|  | CGI_10026493-D23* | scaffold1031 | 557767 | 560421 | SP-LRR(7)-TM-TIR | 0 | N | N | N |
|  | CGI_10005194-D35* | scaffold1031 | 575262 | 576443 | LRR-TM-TIR | 3 | N | N | N |
|  | CGI_10005194-D27* | scaffold1031 | 569158 | 571806 | SP-LRR(7)-TM-TIR | 0 | Y | Y | Y |
|  | CGI_10026493-D25* | scaffold1031 | 601777 | 604341 | LRR(6)-TM-TIR | 0 | Y | N | Y |
|  | CGI_10005194-D6* | scaffold1101 | 310135 | 312423 | SP-LRR(4)-TM-TIR | 0 | Y | N | Y |
|  | CGI_10005194-D16* | scaffold150 | 1843115 | 1845298 | SP-LRR(7)-TM-TIR | 0 | Y | Y | N |
|  | CGI_10005194-D3* | scaffold1752 | 24991 | 27294 | SP-LRR(5)-TM-TIR | 0 | N | N | Y |
|  | CGI_10005194-D2* | scaffold1752 | 33437 | 35734 | SP-LRR(6)-TM-TIR | 0 | Y | N | Y |
|  | CGI_10005194-D9* | scaffold1254 | 9414 | 11980 | SP-LRR(3)-TM-TIR | 1 | Y | N | Y |
|  | CGI_10026493-D19* | scaffold22 | 1612084 | 1614696 | NT-LRR(6)-TM-TIR | 0 | Y | N | Y |
|  | CGI_10026493-D10* | scaffold22 | 1623074 | 1625662 | NT-LRR(7)-CT-TM-TIR | 2 | Y | N | Y |
|  | CGI_10026493-D5* | scaffold22 | 1600411 | 1603059 | SP-LRR(6)-TM-TIR | 0 | Y | N | Y |
|  | CGI_10026493-D2* | scaffold22 | 1545566 | 1547821 | LRR(4)-TM-TIR | 0 | Y | N | Y |
|  | CGI_10005194-D23* | scaffold304 | 209765 | 212365 | SP-NT-LRR(10)-TM-TIR | 0 | Y | Y | Y |
|  | CGI_10005194-D20* | scaffold304 | 236762 | 239365 | SP-LRR(7)-TM-TIR | 0 | Y | N | Y |
|  | CGI_10005194-D14* | scaffold304 | 241272 | 243773 | SP-LRR(4)-TIR | 1 | Y | Y | Y |
|  | CGI_10005194-D25* | scaffold304 | 218475 | 221069 | SP-NT-LRR(5)-TM-TIR | 0 | Y | Y | Y |
|  | CGI_10005194-D11* | scaffold33732 | 4625 | 6913 | SP-LRR(8)-TM-TIR | 0 | Y | N | Y |
|  | CGI_10026493 | scaffold156 | 307826 | 309985 | SP-LRR(6)-TM-TIR | 0 | Y | N | Y |
|  | CGI_10005194-D10* | scaffold767 | 160216 | 162480 | SP-LRR(10)-TM-TIR | 0 | Y | N | Y |
|  | CGI_10005194-D31* | scaffold1179 | 1205025 | 1206311 | TM-TIR | 0 | Y | Y | Y |
|  | CGI_10026493-D32* | scaffold1179 | 1211321 | 1212966 | LRR(4)-TM-TIR | 3 | Y | N | N |
|  | CGI_10002656_R-D6* | scaffold1179 | 1225099 | 1227216 | SP-LRR(5)-TM-TIR | 0 | Y | N | Y |
|  | CGI_10005194-D17* | C34708 | 7314 | 9485 | SP-LRR(6)-TM-TIR | 0 | Y | Y | Y |
|  | CGI_10005194-D21* | C34708 | 12346 | 14498 | SP-LRR(7)-TM-TIR | 1 | N | Y | Y |
|  | CGI_10005194-D12* | scaffold1254 | 2 | 2476 | SP-LRR(4)-TM-TIR | 0 | Y | N | Y |
|  | CGI_10005194-D18* | scaffold1564 | 49952 | 50747 | TM-TIR | 0 | Y | Y | Y |
|  | CGI_10005194-D26* | C34708 | 18193 | 20121 | SP-LRR(7)-TM-TIR | 0 | Y | Y | Y |
|  | CGI_10005194-D4* | scaffold1564 | 15527 | 17611 | LRR(5)-TM-TIR | 0 | Y | N | Y |
|  | CGI_10005194-D8* | scaffold780 | 190464 | 192737 | SP-LRR(6)-TM-TIR | 0 | Y | N | Y |
| Short P type GroupⅡ  (sP-Group Ⅱ) | CGI_10013671-D7* | scaffold41970 | 39139 | 41358 | SP-LRR(7)-TM-TIR | 0 | Y | N | Y |
|  | CGI_10013671-D8* | scaffold41970 | 48727 | 50892 | SP-LRR(6)-TM-TIR | 0 | Y | N | Y |
|  | CGI_10013671-D10* | scaffold41970 | 29971 | 32142 | SP-LRR(7)-TM-TIR | 0 | Y | N | Y |
|  | CGI_10013671 | scaffold43868 | 359935 | 362052 | SP-LRR(10)-CT-TM-TIR | 0 | Y | N | Y |
|  | CGI_10013671-D4* | scaffold354 | 532881 | 534974 | SP-NT-LRR(6)-CT-TM-TIR | 0 | Y | N | Y |
|  | CGI_10025722R | scaffold354 | 525449 | 527569 | SP-NT-LRR(5)-CT-TM-TIR | 0 | Y | N | Y |
|  | CGI_10013671-D3* | scaffold354 | 539734 | 541824 | SP-LRR(6)-CT-TM-TIR | 0 | Y | N | Y |
|  | CGI_10009217R | scaffold1245 | 4291 | 6423 | SP-LRR(8)-CT-TM-TIR | 0 | Y | N | Y |
|  | CGI_10013671-D6* | scaffold425 | 699900 | 702014 | SP-LRR(5)-CT-TM-TIR | 0 | Y | N | Y |
|  | CGI_10009217R-D2* | scaffold621 | 65448 | 67559 | SP-LRR(7)-CT-TM-TIR | 0 | Y | N | Y |
|  | CGI_10009217R-D3* | scaffold1258 | 352916 | 354652 | LRR(7)-CT-TM-TIR | 0 | Y | N | Y |
|  | CGI_10009217R-D4* | scaffold610 | 11610 | 14291 | SP-LRR(4)-CT-TM-TIR | 0 | Y | N | Y |
|  | CGI_10009217R-D5* | scaffold1258 | 376656 | 379182 | LRR(4)-CT-TM-TIR | 0 | Y | N | Y |
|  | CGI_10025722R-D3* | scaffold1671 | 121710 | 123788 | SP-NT-LRR(5)-CT-TM-TIR | 0 | N | N | N |
|  | CGI_10005194-D13* | scaffold1247 | 183563 | 186145 | SP-LRR(4)-TM-TIR | 0 | Y | Y | Y |
| Short P type Group Ⅲ  (sP-Group Ⅲ) | CGI_10003466-D7* | scaffold22 | 1279106 | 1281523 | LRR(5)-TM-TIR | 0 | Y | N | N |
|  | CGI_10003466-D11* | scaffold189 | 395481 | 397994 | LRR(4)-TM-TIR | 0 | N | N | N |
|  | CGI_10003466 | scaffold599 | 692333 | 694714 | LRR(7)-TM-TIR | 0 | Y | Y | Y |
|  | CGI_10003466-D8* | scaffold599 | 665609 | 668050 | LRR(8)-TM-TIR | 0 | Y | Y | Y |
|  | CGI_10003466-D9* | scaffold599 | 682707 | 685074 | LRR(6)-TM-TIR | 0 | Y | Y | Y |
|  | CGI_10003466-D2* | scaffold599 | 705479 | 707944 | LRR(6)-TM-TIR | 0 | Y | N | N |
|  | CGI_10003466-D5* | scaffold599 | 599084 | 601522 | LRR(8)-TM-TIR | 0 | N | N | Y |
|  | CGI_10003466-D4* | scaffold599 | 617735 | 620161 | LRR(6)-TM-TIR | 0 | N | N | Y |
|  | CGI_10003466-D14* | scaffold1852 | 36321 | 38745 | LRR(3)-TM-TIR | 1 | Y | N | Y |
|  | CGI_10003466-D12* | scaffold1852 | 48094 | 50580 | LRR(2)-TM-TIR | 0 | N | N | N |
|  | CGI_10003466-D6* | scaffold599 | 643134 | 645515 | LRR(6)-TM-TIR | 0 | Y | N | Y |
|  | CGI_10003466-D3* | scaffold599 | 592872 | 595232 | LRR(7)-TM-TIR | 0 | Y | N | Y |
|  | CGI_10003466-D13* | scaffold1403 | 363512 | 365833 | LRR(4)-TM-TIR | 0 | Y | N | Y |
|  | CGI_10003466-D10* | scaffold241 | 145533 | 147908 | LRR(5)-TM-TIR | 0 | Y | N | Y |
| Short PP type | CGI_10012212 | scaffold63 | 84543 | 88116 | SP-LRR(2)-CT-NT-(LRR)2-CT-TM-TIR | 0 | Y | Y | Y |
|  | CGI_10012212-D2* | scaffold1256 | 30322 | 32211 | SP-LRR(3)-CT-(LRR)2-CT-TM-TIR | 0 | Y | Y | Y |
| LRRCT specific group | CGI_10015565 | scaffold1901 | 397044 | 398870 | SP-LRR-TM-TIR | 0 | Y | N | Y |
|  | CGI_10002656_R | scaffold38166 | 41363 | 43372 | SP-LRR-CT-TM-TIR | 0 | N | N | N |
|  | CGI_10002656_R-D4* | scaffold432 | 530237 | 531112 | SP-LRR-CT-TM-TIR | 0 | Y | N | N |
|  | CGI_10023107 | scaffold432 | 540614 | 542545 | SP-LRR-CT-TM-TIR | 0 | N | N | N |
|  | CGI_10023107-D4* | scaffold432 | 475568 | 477583 | TM-TIR | 0 | Y | N | N |
|  | CGI_10002656_R-D2* | scaffold718 | 11282 | 13297 | SP-LRR-TM-TIR | 0 | Y | N | N |
|  | CGI_10005194 | scaffold1564 | 28102 | 30679 | SP-LRR(6)-TM-TIR | 0 | Y | N | Y |
|  | CGI_10023107-D3* | scaffold432 | 487774 | 489774 | CT-TM-TIR | 0 | Y | Y | Y |
| Not classified | CGI_10025722R-D11* | scaffold290 | 98647 | 99711 | CT-TM | 0 | Y | N | Y |
|  | CGI_10005194-D30* | scaffold782 | 251971 | 254187 | SP-LRR(5)-TM | 0 | Y | Y | Y |
|  | CGI_10005194-D5* | C24984 | 1694 | 3016 | SP-LRR(3) | 0 | Y | N | Y |

**c）Cross-species comparison of the immune-related protein domains**

| **Domains** | **IPR ID** | **Notes** | ***AQ*** | ***CI*** | | ***HM*** | | ***NV*** | | ***SM*** | | ***CG*** | | ***LG*** | | ***CT*** | | ***HR*** | | **CE** | | ***DM*** | | ***SP*** | | ***BF*** | | ***DR*** | | ***GG*** | | ***MM*** | | ***HS*** | |  |
| --- | --- | --- | --- | --- | --- | --- | --- | --- | --- | --- | --- | --- | --- | --- | --- | --- | --- | --- | --- | --- | --- | --- | --- | --- | --- | --- | --- | --- | --- | --- | --- | --- | --- | --- | --- | --- |
| **domains involved in pathogen recognition and clearance** | | | | | | | | | | | | | | | | | | | | | | | | | | | | | | | | | | | | |
| SRCR | IPR001190 | Scavenger receptor cysteine-rich domain | 301 | 11 | | 10 | | 73 | | 5 | | **71** | | 17 | | 67 | | 20 | | 2 | | 5 | | 426 | | 143 | | 26 | | 32 | | 28 | | 25 | |  |
| CTLD | IPR001304 | Lectin C-type domain | 1 | 73 | | 71 | | 98 | | 10 | | **273** | | 126 | | 221 | | 111 | | 263 | | 38 | | 283 | | 690 | | 126 | | 49 | | 114 | | 84 | |  |
|  | **IPR016187** | C-type lectin fold | 5 | 102 | | 78 | | 107 | | 14 | | **352** | | 146 | | 250 | | 167 | | 275 | | 0 | | 0 | | 733 | | 150 | | 71 | | 135 | | 109 | |  |
| LDLRa | IPR002172 | low density lipoprotein receptors class A | 6 | 77 | | 21 | | 44 | | 15 | | **94** | | 61 | | 122 | | 60 | | 32 | | 41 | | 186 | | 226 | | 45 | | 37 | | 47 | | 48 | |  |
| LDLRB | **IPR000033** | Low-density lipoprotein receptor, YWTD repeat | 20 | 23 | | 16 | | 22 | | 0 | | **43** | | 21 | | 32 | | 12 | | 7 | | 10 | | 97 | | 51 | | 16 | | 14 | | 14 | | 15 | |  |
| IG | IPR007110 | Immunoglobulin-like | 352 | 100 | | 107 | | 266 | | 77 | | **284** | | 199 | | 210 | | 224 | | 76 | | 137 | | 448 | | 411 | | 688 | | 416 | | 827 | | 697 | |  |
| [Ig_E-set](http://supfam.cs.bris.ac.uk/SUPERFAMILY/cgi-bin/scop.cgi?ipid=SSF81296) | **IPR014756** | Immunoglobulin E-set | 151 | 71 | | 44 | | 95 | | 22 | | **100** | | 57 | | 97 | | 65 | | 51 | | 0 | | 0 | | 131 | | 107 | | 67 | | 93 | | 93 | |  |
| [Ig_I-set](http://supfam.cs.bris.ac.uk/SUPERFAMILY/cgi-bin/scop.cgi?ipid=SSF81296) | **IPR013098** | Immunoglobulin I-set | 178 | 74 | | 82 | | 153 | | 31 | | **167** | | 98 | | 115 | | 115 | | 38 | | 58 | | 110 | | 296 | | 178 | | 154 | | 191 | | 187 | |  |
| LRR | IPR001611 | Leucine Rich Repeat | 101 | 113 | | 53 | | 115 | | 50 | | **131** | | 104 | | 343 | | 101 | | 47 | | 89 | | 418 | | 687 | | 217 | | 152 | | 183 | | 190 | |  |
| VWA | **IPR002035** | von Willebrand factor, type A | 57 | 114 | | 64 | | 173 | | 10 | | **166** | | 75 | | 108 | | 47 | | 53 | | 12 | | 65 | | 178 | | 71 | | 57 | | 80 | | 83 | |  |
| FBG | **IPR002181** | complement system, fibrinogen-C-terminal domain | 257 | 69 | | 24 | | 187 | | 0 | | **205** | | 51 | | 141 | | 116 | | 5 | | 15 | | 59 | | 214 | | 48 | | 30 | | 31 | | 32 | |  |
| Galectin | **IPR001079** | carbohydrate recognition domain | 5 | 3 | | 0 | | 0 | | 2 | | **11** | | 1 | | 5 | | 1 | | 25 | | 6 | | 1 | | 1 | | 11 | | 8 | | 12 | | 15 | |  |
| FA58C | **IPR000421** | Coagulation factor 5/8 type, C-terminal | 3 | 18 | | 55 | | 379 | | 8 | | **111** | | 40 | | 153 | | 31 | | 3 | | 7 | | 144 | | 280 | | 36 | | 18 | | 24 | | 24 | |  |
| RIG-I_C-RD | **IPR021673** | C-terminal domain of RIG-I | 2 | 1 | | 0 | | 0 | | 0 | | **8** | | 0 | | 0 | | 0 | | 0 | | 0 | | 0 | | 5 | | 0 | | 2 | | 3 | | 3 | |  |
| HELICc | **IPR001650** | DNA/RNA helicase, C-terminal | 196 | 161 | | 94 | | 104 | | 76 | | **123** | | 63 | | 95 | | 91 | | 79 | | 77 | | 131 | | 98 | | 84 | | 86 | | 107 | | 108 | |  |
| AA(NACHT) | IPR007111 | NACHT nucleoside triphosphatase | 196 | 6 | | 4 | | 44 | | 0 | | **0** | | 1 | | 90 | | 0 | | 1 | | 1 | | 213 | | 60 | | 208 | | 5 | | 32 | | 23 | |  |
| C1q | **IPR001073** | complement system, Complement C1q protein | 0 | 4 | | 2 | | 0 | | 2 | | **333** | | 5 | | 23 | | 9 | | 0 | | 0 | | 12 | | 41 | | 45 | | 27 | | 29 | | 30 | |  |
| CCP | **IPR000436** | Sushi/SCR/CCP | 47 | 119 | | 18 | | 51 | | 1 | | **108** | | 126 | | 82 | | 10 | | 13 | | 16 | | 248 | | 242 | | 55 | | 41 | | 50 | | 58 | |  |
| TSP1 | IPR000884 | complement system, Thrombospondin type 1 repeats | 18 | 157 | | 196 | | 138 | | 17 | | **127** | | 42 | | 100 | | 30 | | 34 | | 18 | | 75 | | 157 | | 77 | | 63 | | 65 | | 67 | |  |
| CUB | **IPR000859** | complement system, Domains in C1r, C1s | 6 | 86 | | 41 | | 87 | | 9 | | **126** | | 71 | | 120 | | 40 | | 50 | | 28 | | 265 | | 246 | | 54 | | 46 | | 53 | | 57 | |  |
| CD225 | **IPR007593** | Interferon-induced transmembrane protein | 25 | 1 | | 21 | | 4 | | 0 | | **33** | | 17 | | 1 | | 0 | | 0 | | 0 | | 13 | | 9 | | 7 | | 6 | | 18 | | 15 | |  |
| MACPF | **IPR020864** | membrane-attack complex/perforin | 4 | 13 | | 9 | | 18 | | 1 | | **15** | | 4 | | 1 | | 0 | | 0 | | 0 | | 23 | | 35 | | 19 | | 10 | | 12 | | 12 | |  |
| Peptidase_C14 | **IPR011600** | Caspase | 49 | 22 | | 29 | | 14 | | 4 | | **31** | | 14 | | 39 | | 14 | | 5 | | 7 | | 42 | | 38 | | 22 | | 12 | | 12 | | 15 | |  |
| Cu-oxidase | **IPR001117** | Multicopper oxidase, type 1 | 24 | 1 | | 5 | | 2 | | 0 | | **32** | | 3 | | 7 | | 0 | | 1 | | 4 | | 9 | | 14 | | 0 | | 2 | | 0 | | 2 | |  |
| **domains of cytokines and their receptors** | |  |  | |  | |  | |  | |  | |  | |  | |  | |  | |  | |  | |  | |  | |  | |  | |  | |  | |
| IL17 | **IPR010345** | IL17 | 0 | 5 | | 0 | | 0 | | 0 | | **8** | | 3 | | 0 | | 0 | | 1 | | 0 | | 14 | | 7 | | 3 | | 4 | | 6 | | 6 | |  |
| TNFR | IPR001368 | tumor necrosis factor receptor | 1 | 4 | | 1 | | 21 | | 3 | | **16** | | 15 | | 6 | | 2 | | 1 | | 2 | | 17 | | 32 | | 23 | | 18 | | 24 | | 26 | |  |
| TNF | **IPR006052** | tumor necrosis factor | 0 | 6 | | 2 | | 6 | | 0 | | **19** | | 6 | | 6 | | 2 | | 0 | | 1 | | 4 | | 17 | | 18 | | 9 | | 18 | | 18 | |  |
|  | **IPR008983** | Tumour necrosis factor-like | 0 | 9 | | 5 | | 7 | | 2 | | **348** | | 12 | | 30 | | 12 | | 1 | | 0 | | 0 | | 59 | | 65 | | 37 | | 49 | | 51 | |  |
| EGF | **IPR006209** | Epidermal growth factor domain | 36 | 109 | | 49 | | 308 | | 31 | | **160** | | 87 | | 189 | | 82 | | 50 | | 41 | | 548 | | 327 | | 135 | | 59 | | 64 | | 60 | |  |
| **domains mediating protein interactions** | |  |  | |  | |  | |  | |  | |  | |  | |  | |  | |  | |  | |  | |  | |  | |  | |  | |  | |
| TIR | **IPR000157** | Toll/IL-1 receptor domain | 7 | 7 | | 8 | | 11 | | 3 | | **112** | | 53 | | 85 | | 15 | | 2 | | 10 | | 245 | | 108 | | 29 | | 22 | | 25 | | 24 | |  |
| CARD | **IPR001315** | Caspase Recruitment | 35 | 7 | | 7 | | 20 | | 3 | | **42** | | 26 | | 181 | | 11 | | 4 | | 0 | | 12 | | 107 | | 18 | | 15 | | 26 | | 27 | |  |
| DEATH | **IPR000488** | Death | 328 | 18 | | 73 | | 40 | | 4 | | **48** | | 35 | | 29 | | 19 | | 6 | | 9 | | 170 | | 219 | | 27 | | 27 | | 33 | | 35 | |  |
| DED | **IPR001875** | Death effector | 9 | 6 | | 21 | | 13 | | 0 | | **10** | | 5 | | 3 | | 1 | | 0 | | 0 | | 6 | | 109 | | 6 | | 6 | | 7 | | 7 | |  |
| BIR | **IPR001370** | Proteinase inhibitor I32, inhibitor of apoptosis | 6 | 31 | | 3 | | 3 | | 2 | | **49** | | 17 | | 24 | | 4 | | 2 | | 4 | | 9 | | 12 | | 6 | | 6 | | 11 | | 8 | |  |
| TRAF | **IPR008974** | TRAF-like | 260 | 22 | | 86 | | 42 | | 9 | | **31** | | 14 | | 22 | | 15 | | 78 | | 0 | | 0 | | 37 | | 22 | | 21 | | 34 | | 22 | |  |
| ANK | **IPR020683** | Ankyrin repeat-containing domain | 1030 | 147 | | 479 | | 256 | | 89 | | **352** | | 164 | | 451 | | 167 | | 95 | | 87 | | 650 | | 239 | | 213 | | 175 | | 232 | | 260 | |  |
| TPR | IPR001440 | Tetratricopeptide repeats | 51 | 64 | | 88 | | 144 | | 40 | | **59** | | 64 | | 69 | | 59 | | 38 | | 49 | | 100 | | 248 | | 75 | | 57 | | 61 | | 74 | |  |
| WD40 | **IPR017986** | WD40-repeat-containing domain | 270 | 264 | | 194 | | 241 | | 137 | | **236** | | 176 | | 238 | | 171 | | 116 | | 150 | | 290 | | 230 | | 210 | | 196 | | 233 | | 244 | |  |
| SAM | **IPR010993** | Sterile alpha motif homology | 62 | 71 | | 52 | | 68 | | 20 | | **77** | | 39 | | 49 | | 42 | | 26 | | 0 | | 0 | | 75 | | 113 | | 98 | | 103 | | 114 | |  |
| SPRY | IPR003877 | domains in butyrophilin/marenostrin/pyrin | 12 | 19 | | 9 | | 19 | | 9 | | **21** | | 13 | | 17 | | 13 | | 12 | | 12 | | 19 | | 33 | | 120 | | 42 | | 79 | | 104 | |  |
| **domains involved in signal transduction** | |  |  | |  | |  | |  | |  | |  | |  | |  | |  | |  | |  | |  | |  | |  | |  | |  | |  | |
| SH2 | IPR000980 | Src homology 2 domains | 94 | 67 | | 29 | | 44 | | 33 | | **46** | | 24 | | 43 | | 60 | | 66 | | 32 | | 52 | | 32 | | 98 | | 82 | | 106 | | 109 | |  |
| SH3 | IPR001452 | Src homology 3 domains | 214 | 147 | | 80 | | 106 | | 51 | | **87** | | 64 | | 101 | | 114 | | 62 | | 73 | | 111 | | 123 | | 215 | | 173 | | 210 | | 215 | |  |
| PDZ | IPR001478 | PDZ/DHR/GLGF | 96 | 88 | | 68 | | 90 | | 70 | | **86** | | 70 | | 99 | | 104 | | 66 | | 67 | | 95 | | 89 | | 169 | | 123 | | 150 | | 154 | |  |
| PH | IPR001849 | pleckstrin, inositol phosphate binding | 153 | 137 | | 79 | | 118 | | 51 | | **110** | | 82 | | 112 | | 110 | | 72 | | 75 | | 117 | | 115 | | 243 | | 207 | | 254 | | 276 | |  |
| PI3Kc | IPR000403 | Phosphoinositide 3-kinase | 19 | 28 | | 12 | | 14 | | 10 | | **13** | | 14 | | 15 | | 13 | | 12 | | 11 | | 21 | | 14 | | 13 | | 18 | | 18 | | 19 | |  |
| S_TKc | IPR002290 | Serine/Threonine protein kinases | 550 | 431 | | 312 | | 266 | | 95 | | **335** | | 223 | | 331 | | 422 | | 163 | | 128 | | 425 | | 111 | | 563 | | 318 | | 401 | | 387 | |  |
| Kinase_like | **IPR011009** | Protein kinase-like domain | 746 | 518 | | 448 | | 433 | | 248 | | **410** | | 272 | | 431 | | 486 | | 477 | | 0 | | 0 | | 612 | | 751 | | 440 | | 543 | | 526 | |  |
| TyrKc | **IPR001245** | Tyrosine kinase | 302 | 119 | | 151 | | 116 | | 41 | | **119** | | 51 | | 118 | | 111 | | 113 | | 44 | | 126 | | 283 | | 110 | | 109 | | 122 | | 120 | |  |
| Y_phosphatase | IPR000242 | Protein tyrosine phosphatase, catalytic domain | 99 | 43 | | 45 | | 55 | | 18 | | **113** | | 42 | | 105 | | 45 | | 100 | | 18 | | 65 | | 30 | | 46 | | 38 | | 40 | | 40 | |  |
| PKC-C1 | IPR002219 | protein kinase C, C1 domain | 33 | 43 | | 26 | | 38 | | 21 | | **26** | | 17 | | 26 | | 39 | | 35 | | 31 | | 32 | | 25 | | 60 | | 46 | | 64 | | 66 | |  |
| PKC-C2 | IPR000008 | Protein kinase C conserved region 2 (CalB) | 41 | 88 | | 71 | | 105 | | 41 | | **72** | | 56 | | 76 | | 81 | | 47 | | 44 | | 87 | | 98 | | 133 | | 109 | | 129 | | 138 | |  |
| Rho | **IPR003578** | Ras small GTPase, Rho type | 119 | 78 | | 77 | | 61 | | 9 | | **118** | | 67 | | 92 | | 71 | | 13 | | 31 | | 97 | | 15 | | 65 | | 64 | | 91 | | 76 | |  |
| Ras | **IPR003577** | Ras small GTPase, Ras type | 129 | 76 | | 91 | | 64 | | 5 | | **130** | | 76 | | 102 | | 81 | | 16 | | 34 | | 112 | | 18 | | 59 | | 68 | | 99 | | 87 | |  |
| Rab | **IPR003579** | Ras small GTPase, Rab type | 159 | 84 | | 116 | | 85 | | 22 | | **146** | | 90 | | 116 | | 94 | | 35 | | 48 | | 138 | | 40 | | 98 | | 84 | | 123 | | 110 | |  |
| ARF | **IPR006689** | ARF/SAR superfamily | 26 | 27 | | 18 | | 26 | | 12 | | **38** | | 20 | | 23 | | 17 | | 14 | | 13 | | 24 | | 21 | | 41 | | 25 | | 30 | | 33 | |  |
| zf-B_box | **IPR000315** | Zinc finger, B-box | 270 | 11 | | 12 | | 87 | | 9 | | **631** | | 43 | | 29 | | 35 | | 21 | | 8 | | 382 | | 240 | | 138 | | 38 | | 65 | | 88 | |  |
| ZF_TRAF | **IPR001293** | Zinc finger, TRAF-type | 190 | 5 | | 20 | | 26 | | 2 | | **16** | | 8 | | 9 | | 4 | | 2 | | 2 | | 10 | | 15 | | 15 | | 9 | | 11 | | 12 | |  |
| IRF | IPR001346 | Interferon regulatory factor | 2 | 6 | | 4 | | 5 | | 1 | | **4** | | 1 | | 3 | | 5 | | 0 | | 0 | | 2 | | 10 | | 11 | | 8 | | 9 | | 9 | |  |
| ARM-type_fold | **IPR016024** | Armadillo-type fold | 246 | 323 | | 219 | | 230 | | 115 | | **251** | | 183 | | 213 | | 160 | | 143 | | 0 | | 0 | | 236 | | 212 | | 251 | | 291 | | 299 | |  |
| KAZAL | **IPR002350** | Proteinase inhibitor I1, Kazal | 5 | 18 | | 24 | | 47 | | 3 | | **38** | | 16 | | 9 | | 14 | | 4 | | 26 | | 26 | | 46 | | 27 | | 30 | | 42 | | 41 | |  |
| KUNITZ | **IPR002223** | Proteinase inhibitor I3, Kunitz legume | 13 | 7 | | 14 | | 20 | | 12 | | **27** | | 12 | | 39 | | 9 | | 38 | | 26 | | 8 | | 17 | | 15 | | 13 | | 20 | | 19 | |  |
| serpin | IPR000215 | Protease inhibitor I4, serpin | 0 | 8 | | 2 | | 4 | | 8 | | **9** | | 7 | | 5 | | 16 | | 14 | | 30 | | 13 | | 6 | | 23 | | 26 | | 64 | | 36 | |  |

AQ：***Amphimedon queenslandica;CI：Ciona intestinalis;HM: Hydra magnipapillata; NV: Nematostella vectensis; SM: Schistosoma mansoni; CG: Crassostrea gigas; LG: Lottia gigantean; CT: Capitella teleta; HR: Helobdella robusta; CE: Caenorhabditis elegans*; DM: *Drosophila melanogaster; SP: Strongylocentrotus purpuratus; BF: Branchiostoma floridae; DR: Danio rerio; GG: Gallus gallus; MM: Mus musculus; HS: Homo sapien***

1. **Summary of the phylostatigraphic ages** using the method of phylostratigraphy

| **Rank** | **Phylostrata** | **Orgnisms** |
| --- | --- | --- |
| 1 | Cellular orgnism | 'Nostoc azollae' 0708 |
| 1 | Cellular orgnism | 'Rehmannia glutinosa' phytoplasma |
| 1 | Cellular orgnism | Abiotrophia defectiva |
| 1 | Cellular orgnism | Abiotrophia defectiva ATCC 49176 |
| 1 | Cellular orgnism | Acaryochloris marina |
| 1 | Cellular orgnism | Acaryochloris marina MBIC11017 |
| 1 | Cellular orgnism | Acetivibrio cellulolyticus |
| 1 | Cellular orgnism | Acetivibrio cellulolyticus CD2 |
| 1 | Cellular orgnism | Acetobacter aceti |
| 1 | Cellular orgnism | Acetobacter pasteurianus |
| 1 | Cellular orgnism | Acetobacter pasteurianus IFO 3283-01 |
| 1 | Cellular orgnism | Acetohalobium arabaticum DSM 5501 |
| 1 | Cellular orgnism | Acholeplasma laidlawii |
| 1 | Cellular orgnism | Acholeplasma laidlawii PG-8A |
| 1 | Cellular orgnism | Achromobacter denitrificans |
| 1 | Cellular orgnism | Achromobacter piechaudii |
| 1 | Cellular orgnism | Achromobacter piechaudii ATCC 43553 |
| 1 | Cellular orgnism | Achromobacter xylosoxidans |
| 1 | Cellular orgnism | Achromobacter xylosoxidans A8 |
| 1 | Cellular orgnism | Acidaminococcus fermentans |
| 1 | Cellular orgnism | Acidaminococcus fermentans DSM 20731 |
| 1 | Cellular orgnism | Acidaminococcus sp. D21 |
| 1 | Cellular orgnism | Acidimicrobium ferrooxidans DSM 10331 |
| 1 | Cellular orgnism | Acidiphilium cryptum |
| 1 | Cellular orgnism | Acidiphilium cryptum JF-5 |
| 1 | Cellular orgnism | Acidiphilium multivorum |
| 1 | Cellular orgnism | Acidithiobacillus caldus |
| 1 | Cellular orgnism | Acidithiobacillus caldus ATCC 51756 |
| 1 | Cellular orgnism | Acidithiobacillus ferrooxidans |
| 1 | Cellular orgnism | Acidithiobacillus ferrooxidans ATCC 23270 |
| 1 | Cellular orgnism | Acidithiobacillus ferrooxidans ATCC 53993 |
| 1 | Cellular orgnism | Acidobacterium capsulatum |
| 1 | Cellular orgnism | Acidobacterium capsulatum ATCC 51196 |
| 1 | Cellular orgnism | Acidobacterium sp. MP5ACTX8 |
| 1 | Cellular orgnism | Acidobacterium sp. MP5ACTX9 |
| 1 | Cellular orgnism | Acidothermus cellulolyticus |
| 1 | Cellular orgnism | Acidothermus cellulolyticus 11B |
| 1 | Cellular orgnism | Acidovorax citrulli |
| 1 | Cellular orgnism | Acidovorax citrulli AAC00-1 |
| 1 | Cellular orgnism | Acidovorax delafieldii |
| 1 | Cellular orgnism | Acidovorax delafieldii 2AN |
| 1 | Cellular orgnism | Acidovorax ebreus TPSY |
| 1 | Cellular orgnism | Acidovorax sp. JS42 |
| 1 | Cellular orgnism | Acinetobacter baumannii |
| 1 | Cellular orgnism | Acinetobacter baumannii AB0057 |
| 1 | Cellular orgnism | Acinetobacter baumannii AB056 |
| 1 | Cellular orgnism | Acinetobacter baumannii AB058 |
| 1 | Cellular orgnism | Acinetobacter baumannii AB059 |
| 1 | Cellular orgnism | Acinetobacter baumannii AB307-0294 |
| 1 | Cellular orgnism | Acinetobacter baumannii AB900 |
| 1 | Cellular orgnism | Acinetobacter baumannii ACICU |
| 1 | Cellular orgnism | Acinetobacter baumannii ATCC 17978 |
| 1 | Cellular orgnism | Acinetobacter baumannii ATCC 19606 |
| 1 | Cellular orgnism | Acinetobacter baumannii AYE |
| 1 | Cellular orgnism | Acinetobacter baumannii SDF |
| 1 | Cellular orgnism | Acinetobacter calcoaceticus |
| 1 | Cellular orgnism | Acinetobacter calcoaceticus RUH2202 |
| 1 | Cellular orgnism | Acinetobacter genomosp. 13TU |
| 1 | Cellular orgnism | Acinetobacter genomosp. 3 |
| 1 | Cellular orgnism | Acinetobacter haemolyticus |
| 1 | Cellular orgnism | Acinetobacter haemolyticus ATCC 19194 |
| 1 | Cellular orgnism | Acinetobacter johnsonii |
| 1 | Cellular orgnism | Acinetobacter johnsonii SH046 |
| 1 | Cellular orgnism | Acinetobacter junii |
| 1 | Cellular orgnism | Acinetobacter junii SH205 |
| 1 | Cellular orgnism | Acinetobacter lwoffii |
| 1 | Cellular orgnism | Acinetobacter lwoffii SH145 |
| 1 | Cellular orgnism | Acinetobacter radioresistens |
| 1 | Cellular orgnism | Acinetobacter radioresistens SH164 |
| 1 | Cellular orgnism | Acinetobacter radioresistens SK82 |
| 1 | Cellular orgnism | Acinetobacter sp. ADP1 |
| 1 | Cellular orgnism | Acinetobacter sp. ATCC 27244 |
| 1 | Cellular orgnism | Acinetobacter sp. DR1 |
| 1 | Cellular orgnism | Acinetobacter sp. EB104 |
| 1 | Cellular orgnism | Acinetobacter sp. RUH2624 |
| 1 | Cellular orgnism | Acinetobacter sp. SH024 |
| 1 | Cellular orgnism | Acinetobacter sp. SUN |
| 1 | Cellular orgnism | Acinetobacter venetianus |
| 1 | Cellular orgnism | Actinobacillus minor |
| 1 | Cellular orgnism | Actinobacillus minor 202 |
| 1 | Cellular orgnism | Actinobacillus minor NM305 |
| 1 | Cellular orgnism | Actinobacillus pleuropneumoniae |
| 1 | Cellular orgnism | Actinobacillus pleuropneumoniae serovar 1 |
| 1 | Cellular orgnism | Actinobacillus pleuropneumoniae serovar 1 str. 4074 |
| 1 | Cellular orgnism | Actinobacillus pleuropneumoniae serovar 10 str. D13039 |
| 1 | Cellular orgnism | Actinobacillus pleuropneumoniae serovar 11 str. 56153 |
| 1 | Cellular orgnism | Actinobacillus pleuropneumoniae serovar 12 str. 1096 |
| 1 | Cellular orgnism | Actinobacillus pleuropneumoniae serovar 13 str. N273 |
| 1 | Cellular orgnism | Actinobacillus pleuropneumoniae serovar 2 |
| 1 | Cellular orgnism | Actinobacillus pleuropneumoniae serovar 2 str. 4226 |
| 1 | Cellular orgnism | Actinobacillus pleuropneumoniae serovar 2 str. S1536 |
| 1 | Cellular orgnism | Actinobacillus pleuropneumoniae serovar 3 str. JL03 |
| 1 | Cellular orgnism | Actinobacillus pleuropneumoniae serovar 4 str. M62 |
| 1 | Cellular orgnism | Actinobacillus pleuropneumoniae serovar 5 |
| 1 | Cellular orgnism | Actinobacillus pleuropneumoniae serovar 6 str. Femo |
| 1 | Cellular orgnism | Actinobacillus pleuropneumoniae serovar 7 str. AP76 |
| 1 | Cellular orgnism | Actinobacillus pleuropneumoniae serovar 9 str. CVJ13261 |
| 1 | Cellular orgnism | Actinobacillus porcitonsillarum |
| 1 | Cellular orgnism | Actinobacillus succinogenes |
| 1 | Cellular orgnism | Actinobacillus succinogenes 130Z |
| 1 | Cellular orgnism | Actinobacillus ureae |
| 1 | Cellular orgnism | Actinomyces coleocanis DSM 15436 |
| 1 | Cellular orgnism | Actinomyces odontolyticus |
| 1 | Cellular orgnism | Actinomyces odontolyticus ATCC 17982 |
| 1 | Cellular orgnism | Actinomyces odontolyticus F0309 |
| 1 | Cellular orgnism | Actinomyces sp. oral taxon 848 str. F0332 |
| 1 | Cellular orgnism | Actinomyces urogenitalis DSM 15434 |
| 1 | Cellular orgnism | Actinomyces viscosus |
| 1 | Cellular orgnism | Actinomyces viscosus C505 |
| 1 | Cellular orgnism | Actinosynnema mirum |
| 1 | Cellular orgnism | Actinosynnema mirum DSM 43827 |
| 1 | Cellular orgnism | Aerococcus viridans |
| 1 | Cellular orgnism | Aerococcus viridans ATCC 11563 |
| 1 | Cellular orgnism | Aeromicrobium marinum DSM 15272 |
| 1 | Cellular orgnism | Aeromonas bestiarum |
| 1 | Cellular orgnism | Aeromonas caviae |
| 1 | Cellular orgnism | Aeromonas hydrophila |
| 1 | Cellular orgnism | Aeromonas hydrophila subsp. hydrophila |
| 1 | Cellular orgnism | Aeromonas hydrophila subsp. hydrophila ATCC 7966 |
| 1 | Cellular orgnism | Aeromonas salmonicida |
| 1 | Cellular orgnism | Aeromonas salmonicida subsp. salmonicida |
| 1 | Cellular orgnism | Aeromonas salmonicida subsp. salmonicida A449 |
| 1 | Cellular orgnism | Afipia sp. 1NLS2 |
| 1 | Cellular orgnism | Aggregatibacter actinomycetemcomitans |
| 1 | Cellular orgnism | Aggregatibacter actinomycetemcomitans D11S-1 |
| 1 | Cellular orgnism | Aggregatibacter actinomycetemcomitans D7S-1 |
| 1 | Cellular orgnism | Aggregatibacter aphrophilus |
| 1 | Cellular orgnism | Aggregatibacter aphrophilus NJ8700 |
| 1 | Cellular orgnism | Aggregatibacter segnis |
| 1 | Cellular orgnism | Agrobacterium radiobacter K84 |
| 1 | Cellular orgnism | Agrobacterium rhizogenes |
| 1 | Cellular orgnism | Agrobacterium tumefaciens |
| 1 | Cellular orgnism | Agrobacterium tumefaciens str. C58 |
| 1 | Cellular orgnism | Agrobacterium vitis |
| 1 | Cellular orgnism | Agrobacterium vitis S4 |
| 1 | Cellular orgnism | Ahrensia sp. R2A130 |
| 1 | Cellular orgnism | Akkermansia muciniphila |
| 1 | Cellular orgnism | Akkermansia muciniphila ATCC BAA-835 |
| 1 | Cellular orgnism | Alcanivorax borkumensis |
| 1 | Cellular orgnism | Alcanivorax borkumensis SK2 |
| 1 | Cellular orgnism | Alcanivorax sp. DG881 |
| 1 | Cellular orgnism | Algoriphagus sp. PR1 |
| 1 | Cellular orgnism | Alicycliphilus denitrificans BC |
| 1 | Cellular orgnism | Alicyclobacillus acidocaldarius |
| 1 | Cellular orgnism | Alicyclobacillus acidocaldarius LAA1 |
| 1 | Cellular orgnism | Alicyclobacillus acidocaldarius subsp. acidocaldarius |
| 1 | Cellular orgnism | Alicyclobacillus acidocaldarius subsp. acidocaldarius DSM 446 |
| 1 | Cellular orgnism | Aliivibrio fischeri |
| 1 | Cellular orgnism | Aliivibrio salmonicida |
| 1 | Cellular orgnism | Aliivibrio salmonicida LFI1238 |
| 1 | Cellular orgnism | Alistipes putredinis |
| 1 | Cellular orgnism | Alistipes putredinis DSM 17216 |
| 1 | Cellular orgnism | Alkalilimnicola ehrlichii MLHE-1 |
| 1 | Cellular orgnism | Alkaliphilus metalliredigens QYMF |
| 1 | Cellular orgnism | Alkaliphilus oremlandii OhILAs |
| 1 | Cellular orgnism | Allochromatium vinosum |
| 1 | Cellular orgnism | Allochromatium vinosum DSM 180 |
| 1 | Cellular orgnism | Alteromonadales bacterium TW-7 |
| 1 | Cellular orgnism | Alteromonas |
| 1 | Cellular orgnism | Alteromonas macleodii |
| 1 | Cellular orgnism | Alteromonas macleodii 'Deep ecotype' |
| 1 | Cellular orgnism | Alteromonas macleodii ATCC 27126 |
| 1 | Cellular orgnism | Aminobacterium colombiense DSM 12261 |
| 1 | Cellular orgnism | Aminomonas paucivorans DSM 12260 |
| 1 | Cellular orgnism | Ammonifex degensii KC4 |
| 1 | Cellular orgnism | Amycolatopsis benzoatilytica |
| 1 | Cellular orgnism | Amycolatopsis mediterranei |
| 1 | Cellular orgnism | Amycolatopsis mediterranei U32 |
| 1 | Cellular orgnism | Anabaena |
| 1 | Cellular orgnism | Anabaena azollae |
| 1 | Cellular orgnism | Anabaena variabilis |
| 1 | Cellular orgnism | Anabaena variabilis ATCC 29413 |
| 1 | Cellular orgnism | Anaerobaculum hydrogeniformans ATCC BAA-1850 |
| 1 | Cellular orgnism | Anaerococcus hydrogenalis DSM 7454 |
| 1 | Cellular orgnism | Anaerococcus lactolyticus |
| 1 | Cellular orgnism | Anaerococcus lactolyticus ATCC 51172 |
| 1 | Cellular orgnism | Anaerococcus prevotii |
| 1 | Cellular orgnism | Anaerococcus prevotii DSM 20548 |
| 1 | Cellular orgnism | Anaerococcus tetradius ATCC 35098 |
| 1 | Cellular orgnism | Anaerococcus vaginalis ATCC 51170 |
| 1 | Cellular orgnism | Anaerofustis stercorihominis DSM 17244 |
| 1 | Cellular orgnism | Anaeromyxobacter dehalogenans |
| 1 | Cellular orgnism | Anaeromyxobacter dehalogenans 2CP-1 |
| 1 | Cellular orgnism | Anaeromyxobacter dehalogenans 2CP-C |
| 1 | Cellular orgnism | Anaeromyxobacter sp. Fw109-5 |
| 1 | Cellular orgnism | Anaeromyxobacter sp. K |
| 1 | Cellular orgnism | Anaerostipes caccae |
| 1 | Cellular orgnism | Anaerostipes caccae DSM 14662 |
| 1 | Cellular orgnism | Anaerotruncus colihominis DSM 17241 |
| 1 | Cellular orgnism | Anaplasma centrale |
| 1 | Cellular orgnism | Anaplasma centrale str. Israel |
| 1 | Cellular orgnism | Anaplasma marginale |
| 1 | Cellular orgnism | Anaplasma marginale str. Florida |
| 1 | Cellular orgnism | Anaplasma marginale str. Mississippi |
| 1 | Cellular orgnism | Anaplasma marginale str. Puerto Rico |
| 1 | Cellular orgnism | Anaplasma marginale str. St. Maries |
| 1 | Cellular orgnism | Anaplasma marginale str. Virginia |
| 1 | Cellular orgnism | Anaplasma phagocytophilum HZ |
| 1 | Cellular orgnism | Anoxybacillus flavithermus |
| 1 | Cellular orgnism | Anoxybacillus flavithermus WK1 |
| 1 | Cellular orgnism | Aquifex aeolicus |
| 1 | Cellular orgnism | Aquifex aeolicus VF5 |
| 1 | Cellular orgnism | Arcanobacterium haemolyticum |
| 1 | Cellular orgnism | Arcanobacterium haemolyticum DSM 20595 |
| 1 | Cellular orgnism | Arcanobacterium pyogenes |
| 1 | Cellular orgnism | Arcobacter butzleri |
| 1 | Cellular orgnism | Arcobacter butzleri RM4018 |
| 1 | Cellular orgnism | Arcobacter nitrofigilis |
| 1 | Cellular orgnism | Arcobacter nitrofigilis DSM 7299 |
| 1 | Cellular orgnism | Aromatoleum aromaticum EbN1 |
| 1 | Cellular orgnism | Arthrobacter arilaitensis |
| 1 | Cellular orgnism | Arthrobacter arilaitensis Re117 |
| 1 | Cellular orgnism | Arthrobacter aurescens |
| 1 | Cellular orgnism | Arthrobacter aurescens TC1 |
| 1 | Cellular orgnism | Arthrobacter chlorophenolicus |
| 1 | Cellular orgnism | Arthrobacter chlorophenolicus A6 |
| 1 | Cellular orgnism | Arthrobacter nitroguajacolicus |
| 1 | Cellular orgnism | Arthrobacter sp. AK-1 |
| 1 | Cellular orgnism | Arthrobacter sp. Chr15 |
| 1 | Cellular orgnism | Arthrobacter sp. FB24 |
| 1 | Cellular orgnism | Arthrospira maxima |
| 1 | Cellular orgnism | Arthrospira maxima CS-328 |
| 1 | Cellular orgnism | Arthrospira platensis |
| 1 | Cellular orgnism | Arthrospira platensis str. Paraca |
| 1 | Cellular orgnism | Aster yellows phytoplasma |
| 1 | Cellular orgnism | Aster yellows witches'-broom phytoplasma |
| 1 | Cellular orgnism | Aster yellows witches'-broom phytoplasma AYWB |
| 1 | Cellular orgnism | Asticcacaulis excentricus CB 48 |
| 1 | Cellular orgnism | Atopobium parvulum DSM 20469 |
| 1 | Cellular orgnism | Atopobium rimae ATCC 49626 |
| 1 | Cellular orgnism | Atopobium vaginae |
| 1 | Cellular orgnism | Atopobium vaginae DSM 15829 |
| 1 | Cellular orgnism | Atopobium vaginae PB189-T1-4 |
| 1 | Cellular orgnism | Aurantimonas manganoxydans SI85-9A1 |
| 1 | Cellular orgnism | Avibacterium paragallinarum |
| 1 | Cellular orgnism | Azoarcus sp. BH72 |
| 1 | Cellular orgnism | Azorhizobium caulinodans |
| 1 | Cellular orgnism | Azorhizobium caulinodans ORS 571 |
| 1 | Cellular orgnism | Azospirillum sp. B510 |
| 1 | Cellular orgnism | Azotobacter vinelandii |
| 1 | Cellular orgnism | Azotobacter vinelandii DJ |
| 1 | Cellular orgnism | Bacillus amyloliquefaciens |
| 1 | Cellular orgnism | Bacillus amyloliquefaciens FZB42 |
| 1 | Cellular orgnism | Bacillus anthracis Tsiankovskii-I |
| 1 | Cellular orgnism | Bacillus anthracis str. 'Ames Ancestor' |
| 1 | Cellular orgnism | Bacillus anthracis str. A0174 |
| 1 | Cellular orgnism | Bacillus anthracis str. A0193 |
| 1 | Cellular orgnism | Bacillus anthracis str. A0248 |
| 1 | Cellular orgnism | Bacillus anthracis str. A0389 |
| 1 | Cellular orgnism | Bacillus anthracis str. A0442 |
| 1 | Cellular orgnism | Bacillus anthracis str. A0465 |
| 1 | Cellular orgnism | Bacillus anthracis str. A0488 |
| 1 | Cellular orgnism | Bacillus anthracis str. A1055 |
| 1 | Cellular orgnism | Bacillus anthracis str. A2012 |
| 1 | Cellular orgnism | Bacillus anthracis str. Ames |
| 1 | Cellular orgnism | Bacillus anthracis str. Australia 94 |
| 1 | Cellular orgnism | Bacillus anthracis str. CDC 684 |
| 1 | Cellular orgnism | Bacillus anthracis str. CNEVA-9066 |
| 1 | Cellular orgnism | Bacillus anthracis str. Kruger B |
| 1 | Cellular orgnism | Bacillus anthracis str. Sterne |
| 1 | Cellular orgnism | Bacillus anthracis str. Vollum |
| 1 | Cellular orgnism | Bacillus anthracis str. Western North America USA6153 |
| 1 | Cellular orgnism | Bacillus atrophaeus |
| 1 | Cellular orgnism | Bacillus cellulosilyticus DSM 2522 |
| 1 | Cellular orgnism | Bacillus cereus |
| 1 | Cellular orgnism | Bacillus cereus 03BB102 |
| 1 | Cellular orgnism | Bacillus cereus 03BB108 |
| 1 | Cellular orgnism | Bacillus cereus 172560W |
| 1 | Cellular orgnism | Bacillus cereus 95/8201 |
| 1 | Cellular orgnism | Bacillus cereus AH1134 |
| 1 | Cellular orgnism | Bacillus cereus AH1271 |
| 1 | Cellular orgnism | Bacillus cereus AH1272 |
| 1 | Cellular orgnism | Bacillus cereus AH1273 |
| 1 | Cellular orgnism | Bacillus cereus AH187 |
| 1 | Cellular orgnism | Bacillus cereus AH603 |
| 1 | Cellular orgnism | Bacillus cereus AH621 |
| 1 | Cellular orgnism | Bacillus cereus AH676 |
| 1 | Cellular orgnism | Bacillus cereus AH820 |
| 1 | Cellular orgnism | Bacillus cereus ATCC 10876 |
| 1 | Cellular orgnism | Bacillus cereus ATCC 10987 |
| 1 | Cellular orgnism | Bacillus cereus ATCC 14579 |
| 1 | Cellular orgnism | Bacillus cereus ATCC 4342 |
| 1 | Cellular orgnism | Bacillus cereus B4264 |
| 1 | Cellular orgnism | Bacillus cereus BDRD-Cer4 |
| 1 | Cellular orgnism | Bacillus cereus BDRD-ST196 |
| 1 | Cellular orgnism | Bacillus cereus BDRD-ST24 |
| 1 | Cellular orgnism | Bacillus cereus BDRD-ST26 |
| 1 | Cellular orgnism | Bacillus cereus BGSC 6E1 |
| 1 | Cellular orgnism | Bacillus cereus E33L |
| 1 | Cellular orgnism | Bacillus cereus F65185 |
| 1 | Cellular orgnism | Bacillus cereus G9241 |
| 1 | Cellular orgnism | Bacillus cereus G9842 |
| 1 | Cellular orgnism | Bacillus cereus H3081.97 |
| 1 | Cellular orgnism | Bacillus cereus MM3 |
| 1 | Cellular orgnism | Bacillus cereus NVH0597-99 |
| 1 | Cellular orgnism | Bacillus cereus Q1 |
| 1 | Cellular orgnism | Bacillus cereus R309803 |
| 1 | Cellular orgnism | Bacillus cereus Rock1-15 |
| 1 | Cellular orgnism | Bacillus cereus Rock1-3 |
| 1 | Cellular orgnism | Bacillus cereus Rock3-28 |
| 1 | Cellular orgnism | Bacillus cereus Rock3-29 |
| 1 | Cellular orgnism | Bacillus cereus Rock3-42 |
| 1 | Cellular orgnism | Bacillus cereus Rock3-44 |
| 1 | Cellular orgnism | Bacillus cereus Rock4-18 |
| 1 | Cellular orgnism | Bacillus cereus Rock4-2 |
| 1 | Cellular orgnism | Bacillus cereus SJ1 |
| 1 | Cellular orgnism | Bacillus cereus W |
| 1 | Cellular orgnism | Bacillus cereus biovar anthracis str. CI |
| 1 | Cellular orgnism | Bacillus cereus m1293 |
| 1 | Cellular orgnism | Bacillus cereus m1550 |
| 1 | Cellular orgnism | Bacillus clausii |
| 1 | Cellular orgnism | Bacillus clausii KSM-K16 |
| 1 | Cellular orgnism | Bacillus coagulans |
| 1 | Cellular orgnism | Bacillus coagulans 36D1 |
| 1 | Cellular orgnism | Bacillus coahuilensis |
| 1 | Cellular orgnism | Bacillus coahuilensis m4-4 |
| 1 | Cellular orgnism | Bacillus cytotoxicus |
| 1 | Cellular orgnism | Bacillus cytotoxicus NVH 391-98 |
| 1 | Cellular orgnism | Bacillus halodurans |
| 1 | Cellular orgnism | Bacillus halodurans C-125 |
| 1 | Cellular orgnism | Bacillus licheniformis |
| 1 | Cellular orgnism | Bacillus licheniformis ATCC 14580 |
| 1 | Cellular orgnism | Bacillus megaterium |
| 1 | Cellular orgnism | Bacillus megaterium DSM 319 |
| 1 | Cellular orgnism | Bacillus megaterium QM B1551 |
| 1 | Cellular orgnism | Bacillus methanolicus |
| 1 | Cellular orgnism | Bacillus methanolicus MGA3 |
| 1 | Cellular orgnism | Bacillus mycoides |
| 1 | Cellular orgnism | Bacillus mycoides DSM 2048 |
| 1 | Cellular orgnism | Bacillus mycoides Rock1-4 |
| 1 | Cellular orgnism | Bacillus mycoides Rock3-17 |
| 1 | Cellular orgnism | Bacillus pseudofirmus |
| 1 | Cellular orgnism | Bacillus pseudofirmus OF4 |
| 1 | Cellular orgnism | Bacillus pseudomycoides |
| 1 | Cellular orgnism | Bacillus pseudomycoides DSM 12442 |
| 1 | Cellular orgnism | Bacillus pumilus |
| 1 | Cellular orgnism | Bacillus pumilus ATCC 7061 |
| 1 | Cellular orgnism | Bacillus pumilus SAFR-032 |
| 1 | Cellular orgnism | Bacillus selenitireducens MLS10 |
| 1 | Cellular orgnism | Bacillus sp. B-3 |
| 1 | Cellular orgnism | Bacillus sp. B14905 |
| 1 | Cellular orgnism | Bacillus sp. BS-01 |
| 1 | Cellular orgnism | Bacillus sp. BS-02 |
| 1 | Cellular orgnism | Bacillus sp. JAMB750 |
| 1 | Cellular orgnism | Bacillus sp. NRRL B-14911 |
| 1 | Cellular orgnism | Bacillus sp. SG-1 |
| 1 | Cellular orgnism | Bacillus subtilis |
| 1 | Cellular orgnism | Bacillus subtilis subsp. natto |
| 1 | Cellular orgnism | Bacillus subtilis subsp. spizizenii |
| 1 | Cellular orgnism | Bacillus subtilis subsp. spizizenii ATCC 6633 |
| 1 | Cellular orgnism | Bacillus subtilis subsp. spizizenii str. W23 |
| 1 | Cellular orgnism | Bacillus subtilis subsp. subtilis |
| 1 | Cellular orgnism | Bacillus subtilis subsp. subtilis str. 168 |
| 1 | Cellular orgnism | Bacillus subtilis subsp. subtilis str. JH642 |
| 1 | Cellular orgnism | Bacillus subtilis subsp. subtilis str. NCIB 3610 |
| 1 | Cellular orgnism | Bacillus subtilis subsp. subtilis str. SMY |
| 1 | Cellular orgnism | Bacillus thuringiensis |
| 1 | Cellular orgnism | Bacillus thuringiensis BMB171 |
| 1 | Cellular orgnism | Bacillus thuringiensis Bt407 |
| 1 | Cellular orgnism | Bacillus thuringiensis IBL 200 |
| 1 | Cellular orgnism | Bacillus thuringiensis IBL 4222 |
| 1 | Cellular orgnism | Bacillus thuringiensis serovar andalousiensis |
| 1 | Cellular orgnism | Bacillus thuringiensis serovar andalousiensis BGSC 4AW1 |
| 1 | Cellular orgnism | Bacillus thuringiensis serovar berliner |
| 1 | Cellular orgnism | Bacillus thuringiensis serovar berliner ATCC 10792 |
| 1 | Cellular orgnism | Bacillus thuringiensis serovar darmstadiensis |
| 1 | Cellular orgnism | Bacillus thuringiensis serovar entomocidus |
| 1 | Cellular orgnism | Bacillus thuringiensis serovar huazhongensis |
| 1 | Cellular orgnism | Bacillus thuringiensis serovar huazhongensis BGSC 4BD1 |
| 1 | Cellular orgnism | Bacillus thuringiensis serovar israelensis |
| 1 | Cellular orgnism | Bacillus thuringiensis serovar israelensis ATCC 35646 |
| 1 | Cellular orgnism | Bacillus thuringiensis serovar konkukian |
| 1 | Cellular orgnism | Bacillus thuringiensis serovar konkukian str. 97-27 |
| 1 | Cellular orgnism | Bacillus thuringiensis serovar kurstaki |
| 1 | Cellular orgnism | Bacillus thuringiensis serovar kurstaki str. T03a001 |
| 1 | Cellular orgnism | Bacillus thuringiensis serovar kurstaki str. YBT-1520 |
| 1 | Cellular orgnism | Bacillus thuringiensis serovar monterrey |
| 1 | Cellular orgnism | Bacillus thuringiensis serovar monterrey BGSC 4AJ1 |
| 1 | Cellular orgnism | Bacillus thuringiensis serovar pakistani |
| 1 | Cellular orgnism | Bacillus thuringiensis serovar pakistani str. T13001 |
| 1 | Cellular orgnism | Bacillus thuringiensis serovar pondicheriensis |
| 1 | Cellular orgnism | Bacillus thuringiensis serovar pondicheriensis BGSC 4BA1 |
| 1 | Cellular orgnism | Bacillus thuringiensis serovar pulsiensis |
| 1 | Cellular orgnism | Bacillus thuringiensis serovar pulsiensis BGSC 4CC1 |
| 1 | Cellular orgnism | Bacillus thuringiensis serovar sotto |
| 1 | Cellular orgnism | Bacillus thuringiensis serovar sotto str. T04001 |
| 1 | Cellular orgnism | Bacillus thuringiensis serovar tenebrionis |
| 1 | Cellular orgnism | Bacillus thuringiensis serovar thuringiensis |
| 1 | Cellular orgnism | Bacillus thuringiensis serovar thuringiensis str. T01001 |
| 1 | Cellular orgnism | Bacillus thuringiensis serovar tochigiensis |
| 1 | Cellular orgnism | Bacillus thuringiensis serovar tochigiensis BGSC 4Y1 |
| 1 | Cellular orgnism | Bacillus thuringiensis str. Al Hakam |
| 1 | Cellular orgnism | Bacillus tusciae |
| 1 | Cellular orgnism | Bacillus tusciae DSM 2912 |
| 1 | Cellular orgnism | Bacillus weihenstephanensis |
| 1 | Cellular orgnism | Bacillus weihenstephanensis KBAB4 |
| 1 | Cellular orgnism | Bacteroides caccae |
| 1 | Cellular orgnism | Bacteroides caccae ATCC 43185 |
| 1 | Cellular orgnism | Bacteroides capillosus ATCC 29799 |
| 1 | Cellular orgnism | Bacteroides cellulosilyticus |
| 1 | Cellular orgnism | Bacteroides cellulosilyticus DSM 14838 |
| 1 | Cellular orgnism | Bacteroides coprocola |
| 1 | Cellular orgnism | Bacteroides coprocola DSM 17136 |
| 1 | Cellular orgnism | Bacteroides coprophilus |
| 1 | Cellular orgnism | Bacteroides coprophilus DSM 18228 |
| 1 | Cellular orgnism | Bacteroides dorei 5_1_36/D4 |
| 1 | Cellular orgnism | Bacteroides dorei DSM 17855 |
| 1 | Cellular orgnism | Bacteroides eggerthii |
| 1 | Cellular orgnism | Bacteroides eggerthii DSM 20697 |
| 1 | Cellular orgnism | Bacteroides finegoldii DSM 17565 |
| 1 | Cellular orgnism | Bacteroides fragilis |
| 1 | Cellular orgnism | Bacteroides fragilis 3_1_12 |
| 1 | Cellular orgnism | Bacteroides fragilis NCTC 9343 |
| 1 | Cellular orgnism | Bacteroides fragilis YCH46 |
| 1 | Cellular orgnism | Bacteroides helcogenes |
| 1 | Cellular orgnism | Bacteroides helcogenes P 36-108 |
| 1 | Cellular orgnism | Bacteroides intestinalis DSM 17393 |
| 1 | Cellular orgnism | Bacteroides ovatus |
| 1 | Cellular orgnism | Bacteroides ovatus ATCC 8483 |
| 1 | Cellular orgnism | Bacteroides ovatus SD CC 2a |
| 1 | Cellular orgnism | Bacteroides ovatus SD CMC 3f |
| 1 | Cellular orgnism | Bacteroides pectinophilus ATCC 43243 |
| 1 | Cellular orgnism | Bacteroides plebeius |
| 1 | Cellular orgnism | Bacteroides plebeius DSM 17135 |
| 1 | Cellular orgnism | Bacteroides sp. 1_1_14 |
| 1 | Cellular orgnism | Bacteroides sp. 1_1_6 |
| 1 | Cellular orgnism | Bacteroides sp. 20_3 |
| 1 | Cellular orgnism | Bacteroides sp. 2_1_16 |
| 1 | Cellular orgnism | Bacteroides sp. 2_1_22 |
| 1 | Cellular orgnism | Bacteroides sp. 2_1_33B |
| 1 | Cellular orgnism | Bacteroides sp. 2_1_7 |
| 1 | Cellular orgnism | Bacteroides sp. 2_2_4 |
| 1 | Cellular orgnism | Bacteroides sp. 3_1_19 |
| 1 | Cellular orgnism | Bacteroides sp. 3_1_23 |
| 1 | Cellular orgnism | Bacteroides sp. 3_1_33FAA |
| 1 | Cellular orgnism | Bacteroides sp. 3_2_5 |
| 1 | Cellular orgnism | Bacteroides sp. 4_3_47FAA |
| 1 | Cellular orgnism | Bacteroides sp. 9_1_42FAA |
| 1 | Cellular orgnism | Bacteroides sp. D1 |
| 1 | Cellular orgnism | Bacteroides sp. D2 |
| 1 | Cellular orgnism | Bacteroides sp. D20 |
| 1 | Cellular orgnism | Bacteroides sp. D22 |
| 1 | Cellular orgnism | Bacteroides stercoris |
| 1 | Cellular orgnism | Bacteroides stercoris ATCC 43183 |
| 1 | Cellular orgnism | Bacteroides thetaiotaomicron |
| 1 | Cellular orgnism | Bacteroides thetaiotaomicron VPI-5482 |
| 1 | Cellular orgnism | Bacteroides uniformis |
| 1 | Cellular orgnism | Bacteroides uniformis ATCC 8492 |
| 1 | Cellular orgnism | Bacteroides vulgatus |
| 1 | Cellular orgnism | Bacteroides vulgatus ATCC 8482 |
| 1 | Cellular orgnism | Bacteroides vulgatus PC510 |
| 1 | Cellular orgnism | Bacteroides xylanisolvens |
| 1 | Cellular orgnism | Bacteroides xylanisolvens SD CC 1b |
| 1 | Cellular orgnism | Bacteroidetes oral taxon 274 str. F0058 |
| 1 | Cellular orgnism | Bartonella bacilliformis |
| 1 | Cellular orgnism | Bartonella bacilliformis KC583 |
| 1 | Cellular orgnism | Bartonella clarridgeiae |
| 1 | Cellular orgnism | Bartonella grahamii |
| 1 | Cellular orgnism | Bartonella grahamii as4aup |
| 1 | Cellular orgnism | Bartonella henselae |
| 1 | Cellular orgnism | Bartonella henselae str. Houston-1 |
| 1 | Cellular orgnism | Bartonella quintana |
| 1 | Cellular orgnism | Bartonella quintana str. Toulouse |
| 1 | Cellular orgnism | Bartonella tribocorum |
| 1 | Cellular orgnism | Bartonella tribocorum CIP 105476 |
| 1 | Cellular orgnism | Baumannia cicadellinicola str. Hc (Homalodisca coagulata) |
| 1 | Cellular orgnism | Bdellovibrio bacteriovorus |
| 1 | Cellular orgnism | Bdellovibrio bacteriovorus HD100 |
| 1 | Cellular orgnism | Beet leafhopper transmitted virescence phytoplasma |
| 1 | Cellular orgnism | Beggiatoa sp. PS |
| 1 | Cellular orgnism | Beggiatoa sp. SS |
| 1 | Cellular orgnism | Beijerinckia indica subsp. indica |
| 1 | Cellular orgnism | Beijerinckia indica subsp. indica ATCC 9039 |
| 1 | Cellular orgnism | Beutenbergia cavernae DSM 12333 |
| 1 | Cellular orgnism | Bibersteinia trehalosi |
| 1 | Cellular orgnism | Bifidobacterium adolescentis |
| 1 | Cellular orgnism | Bifidobacterium adolescentis ATCC 15703 |
| 1 | Cellular orgnism | Bifidobacterium adolescentis L2-32 |
| 1 | Cellular orgnism | Bifidobacterium angulatum |
| 1 | Cellular orgnism | Bifidobacterium angulatum DSM 20098 |
| 1 | Cellular orgnism | Bifidobacterium animalis |
| 1 | Cellular orgnism | Bifidobacterium animalis subsp. lactis |
| 1 | Cellular orgnism | Bifidobacterium animalis subsp. lactis AD011 |
| 1 | Cellular orgnism | Bifidobacterium animalis subsp. lactis Bl-04 |
| 1 | Cellular orgnism | Bifidobacterium animalis subsp. lactis DSM 10140 |
| 1 | Cellular orgnism | Bifidobacterium animalis subsp. lactis HN019 |
| 1 | Cellular orgnism | Bifidobacterium asteroides |
| 1 | Cellular orgnism | Bifidobacterium bifidum |
| 1 | Cellular orgnism | Bifidobacterium bifidum NCIMB 41171 |
| 1 | Cellular orgnism | Bifidobacterium bifidum S17 |
| 1 | Cellular orgnism | Bifidobacterium breve |
| 1 | Cellular orgnism | Bifidobacterium breve DSM 20213 |
| 1 | Cellular orgnism | Bifidobacterium catenulatum |
| 1 | Cellular orgnism | Bifidobacterium catenulatum DSM 16992 |
| 1 | Cellular orgnism | Bifidobacterium dentium |
| 1 | Cellular orgnism | Bifidobacterium dentium ATCC 27678 |
| 1 | Cellular orgnism | Bifidobacterium dentium ATCC 27679 |
| 1 | Cellular orgnism | Bifidobacterium dentium Bd1 |
| 1 | Cellular orgnism | Bifidobacterium dentium JCVIHMP022 |
| 1 | Cellular orgnism | Bifidobacterium gallicum |
| 1 | Cellular orgnism | Bifidobacterium gallicum DSM 20093 |
| 1 | Cellular orgnism | Bifidobacterium longum |
| 1 | Cellular orgnism | Bifidobacterium longum DJO10A |
| 1 | Cellular orgnism | Bifidobacterium longum NCC2705 |
| 1 | Cellular orgnism | Bifidobacterium longum subsp. infantis |
| 1 | Cellular orgnism | Bifidobacterium longum subsp. infantis ATCC 15697 |
| 1 | Cellular orgnism | Bifidobacterium longum subsp. infantis ATCC 55813 |
| 1 | Cellular orgnism | Bifidobacterium longum subsp. infantis CCUG 52486 |
| 1 | Cellular orgnism | Bifidobacterium longum subsp. longum |
| 1 | Cellular orgnism | Bifidobacterium longum subsp. longum JCM 1217 |
| 1 | Cellular orgnism | Bifidobacterium longum subsp. longum JDM301 |
| 1 | Cellular orgnism | Bifidobacterium pseudocatenulatum |
| 1 | Cellular orgnism | Bifidobacterium pseudocatenulatum DSM 20438 |
| 1 | Cellular orgnism | Bifidobacterium pseudolongum |
| 1 | Cellular orgnism | Bifidobacterium pseudolongum subsp. globosum |
| 1 | Cellular orgnism | Bifidobacterium sp. A24 |
| 1 | Cellular orgnism | Bilophila wadsworthia |
| 1 | Cellular orgnism | Blastopirellula marina DSM 3645 |
| 1 | Cellular orgnism | Blattabacterium sp. (Blattella germanica) str. Bge |
| 1 | Cellular orgnism | Blattabacterium sp. (Periplaneta americana) str. BPLAN |
| 1 | Cellular orgnism | Blautia hansenii |
| 1 | Cellular orgnism | Blautia hansenii DSM 20583 |
| 1 | Cellular orgnism | Blautia hydrogenotrophica DSM 10507 |
| 1 | Cellular orgnism | Bordetella avium |
| 1 | Cellular orgnism | Bordetella avium 197N |
| 1 | Cellular orgnism | Bordetella bronchiseptica |
| 1 | Cellular orgnism | Bordetella bronchiseptica RB50 |
| 1 | Cellular orgnism | Bordetella parapertussis |
| 1 | Cellular orgnism | Bordetella parapertussis 12822 |
| 1 | Cellular orgnism | Bordetella pertussis |
| 1 | Cellular orgnism | Bordetella pertussis Tohama I |
| 1 | Cellular orgnism | Bordetella petrii |
| 1 | Cellular orgnism | Bordetella petrii DSM 12804 |
| 1 | Cellular orgnism | Borrelia afzelii |
| 1 | Cellular orgnism | Borrelia afzelii ACA-1 |
| 1 | Cellular orgnism | Borrelia afzelii PKo |
| 1 | Cellular orgnism | Borrelia bavariensis |
| 1 | Cellular orgnism | Borrelia burgdorferi 118a |
| 1 | Cellular orgnism | Borrelia burgdorferi 156a |
| 1 | Cellular orgnism | Borrelia burgdorferi 29805 |
| 1 | Cellular orgnism | Borrelia burgdorferi 64b |
| 1 | Cellular orgnism | Borrelia burgdorferi 72a |
| 1 | Cellular orgnism | Borrelia burgdorferi 80a |
| 1 | Cellular orgnism | Borrelia burgdorferi 94a |
| 1 | Cellular orgnism | Borrelia burgdorferi B31 |
| 1 | Cellular orgnism | Borrelia burgdorferi Bol26 |
| 1 | Cellular orgnism | Borrelia burgdorferi WI91-23 |
| 1 | Cellular orgnism | Borrelia burgdorferi ZS7 |
| 1 | Cellular orgnism | Borrelia duttonii |
| 1 | Cellular orgnism | Borrelia duttonii Ly |
| 1 | Cellular orgnism | Borrelia garinii |
| 1 | Cellular orgnism | Borrelia garinii Far04 |
| 1 | Cellular orgnism | Borrelia garinii PBi |
| 1 | Cellular orgnism | Borrelia garinii PBr |
| 1 | Cellular orgnism | Borrelia hermsii |
| 1 | Cellular orgnism | Borrelia hermsii DAH |
| 1 | Cellular orgnism | Borrelia recurrentis |
| 1 | Cellular orgnism | Borrelia recurrentis A1 |
| 1 | Cellular orgnism | Borrelia sp. SV1 |
| 1 | Cellular orgnism | Borrelia spielmanii |
| 1 | Cellular orgnism | Borrelia spielmanii A14S |
| 1 | Cellular orgnism | Borrelia turicatae |
| 1 | Cellular orgnism | Borrelia turicatae 91E135 |
| 1 | Cellular orgnism | Borrelia valaisiana |
| 1 | Cellular orgnism | Borrelia valaisiana VS116 |
| 1 | Cellular orgnism | Brachybacterium faecium DSM 4810 |
| 1 | Cellular orgnism | Brachyspira hyodysenteriae |
| 1 | Cellular orgnism | Brachyspira hyodysenteriae WA1 |
| 1 | Cellular orgnism | Brachyspira murdochii |
| 1 | Cellular orgnism | Brachyspira murdochii DSM 12563 |
| 1 | Cellular orgnism | Brachyspira pilosicoli |
| 1 | Cellular orgnism | Brachyspira pilosicoli 95/1000 |
| 1 | Cellular orgnism | Bradyrhizobium japonicum |
| 1 | Cellular orgnism | Bradyrhizobium japonicum USDA 110 |
| 1 | Cellular orgnism | Bradyrhizobium sp. BTAi1 |
| 1 | Cellular orgnism | Bradyrhizobium sp. ORS278 |
| 1 | Cellular orgnism | Brevibacillus borstelensis |
| 1 | Cellular orgnism | Brevibacillus brevis |
| 1 | Cellular orgnism | Brevibacillus brevis NBRC 100599 |
| 1 | Cellular orgnism | Brevibacterium |
| 1 | Cellular orgnism | Brevibacterium linens |
| 1 | Cellular orgnism | Brevibacterium linens BL2 |
| 1 | Cellular orgnism | Brevibacterium mcbrellneri ATCC 49030 |
| 1 | Cellular orgnism | Brevundimonas sp. BAL3 |
| 1 | Cellular orgnism | Brevundimonas subvibrioides |
| 1 | Cellular orgnism | Brevundimonas subvibrioides ATCC 15264 |
| 1 | Cellular orgnism | Brucella abortus |
| 1 | Cellular orgnism | Brucella abortus NCTC 8038 |
| 1 | Cellular orgnism | Brucella abortus S19 |
| 1 | Cellular orgnism | Brucella abortus bv. 1 |
| 1 | Cellular orgnism | Brucella abortus bv. 1 str. 9-941 |
| 1 | Cellular orgnism | Brucella abortus bv. 2 str. 86/8/59 |
| 1 | Cellular orgnism | Brucella abortus bv. 3 |
| 1 | Cellular orgnism | Brucella abortus bv. 3 str. Tulya |
| 1 | Cellular orgnism | Brucella abortus bv. 4 str. 292 |
| 1 | Cellular orgnism | Brucella abortus bv. 5 str. B3196 |
| 1 | Cellular orgnism | Brucella abortus bv. 6 str. 870 |
| 1 | Cellular orgnism | Brucella abortus bv. 9 str. C68 |
| 1 | Cellular orgnism | Brucella abortus str. 2308 A |
| 1 | Cellular orgnism | Brucella canis |
| 1 | Cellular orgnism | Brucella canis ATCC 23365 |
| 1 | Cellular orgnism | Brucella ceti |
| 1 | Cellular orgnism | Brucella ceti B1/94 |
| 1 | Cellular orgnism | Brucella ceti M13/05/1 |
| 1 | Cellular orgnism | Brucella ceti M490/95/1 |
| 1 | Cellular orgnism | Brucella ceti M644/93/1 |
| 1 | Cellular orgnism | Brucella ceti str. Cudo |
| 1 | Cellular orgnism | Brucella melitensis |
| 1 | Cellular orgnism | Brucella melitensis ATCC 23457 |
| 1 | Cellular orgnism | Brucella melitensis biovar Abortus 2308 |
| 1 | Cellular orgnism | Brucella melitensis bv. 1 str. 16M |
| 1 | Cellular orgnism | Brucella melitensis bv. 1 str. Rev.1 |
| 1 | Cellular orgnism | Brucella melitensis bv. 2 str. 63/9 |
| 1 | Cellular orgnism | Brucella melitensis bv. 3 str. Ether |
| 1 | Cellular orgnism | Brucella microti |
| 1 | Cellular orgnism | Brucella microti CCM 4915 |
| 1 | Cellular orgnism | Brucella neotomae |
| 1 | Cellular orgnism | Brucella neotomae 5K33 |
| 1 | Cellular orgnism | Brucella ovis |
| 1 | Cellular orgnism | Brucella ovis ATCC 25840 |
| 1 | Cellular orgnism | Brucella pinnipedialis |
| 1 | Cellular orgnism | Brucella pinnipedialis B2/94 |
| 1 | Cellular orgnism | Brucella pinnipedialis M163/99/10 |
| 1 | Cellular orgnism | Brucella pinnipedialis M292/94/1 |
| 1 | Cellular orgnism | Brucella sp. 83/13 |
| 1 | Cellular orgnism | Brucella sp. BO1 |
| 1 | Cellular orgnism | Brucella sp. BO2 |
| 1 | Cellular orgnism | Brucella sp. F5/99 |
| 1 | Cellular orgnism | Brucella sp. NF 2653 |
| 1 | Cellular orgnism | Brucella sp. NVSL 07-0026 |
| 1 | Cellular orgnism | Brucella suis |
| 1 | Cellular orgnism | Brucella suis 1330 |
| 1 | Cellular orgnism | Brucella suis ATCC 23445 |
| 1 | Cellular orgnism | Brucella suis bv. 3 str. 686 |
| 1 | Cellular orgnism | Brucella suis bv. 4 str. 40 |
| 1 | Cellular orgnism | Brucella suis bv. 5 str. 513 |
| 1 | Cellular orgnism | Buchnera aphidicola |
| 1 | Cellular orgnism | Buchnera aphidicola (Acyrthosiphon pisum) |
| 1 | Cellular orgnism | Buchnera aphidicola (Cinara cedri) |
| 1 | Cellular orgnism | Buchnera aphidicola (Schizaphis graminum) |
| 1 | Cellular orgnism | Buchnera aphidicola str. 5A (Acyrthosiphon pisum) |
| 1 | Cellular orgnism | Buchnera aphidicola str. APS (Acyrthosiphon pisum) |
| 1 | Cellular orgnism | Buchnera aphidicola str. Bp (Baizongia pistaciae) |
| 1 | Cellular orgnism | Buchnera aphidicola str. Cc (Cinara cedri) |
| 1 | Cellular orgnism | Buchnera aphidicola str. LSR1 (Acyrthosiphon pisum) |
| 1 | Cellular orgnism | Buchnera aphidicola str. Sg (Schizaphis graminum) |
| 1 | Cellular orgnism | Buchnera aphidicola str. Tuc7 (Acyrthosiphon pisum) |
| 1 | Cellular orgnism | Bulleidia extructa W1219 |
| 1 | Cellular orgnism | Burkholderia ambifaria |
| 1 | Cellular orgnism | Burkholderia ambifaria AMMD |
| 1 | Cellular orgnism | Burkholderia ambifaria IOP40-10 |
| 1 | Cellular orgnism | Burkholderia ambifaria MC40-6 |
| 1 | Cellular orgnism | Burkholderia ambifaria MEX-5 |
| 1 | Cellular orgnism | Burkholderia cenocepacia |
| 1 | Cellular orgnism | Burkholderia cenocepacia AU 1054 |
| 1 | Cellular orgnism | Burkholderia cenocepacia HI2424 |
| 1 | Cellular orgnism | Burkholderia cenocepacia J2315 |
| 1 | Cellular orgnism | Burkholderia cenocepacia MC0-3 |
| 1 | Cellular orgnism | Burkholderia cenocepacia PC184 |
| 1 | Cellular orgnism | Burkholderia cepacia |
| 1 | Cellular orgnism | Burkholderia dolosa |
| 1 | Cellular orgnism | Burkholderia dolosa AUO158 |
| 1 | Cellular orgnism | Burkholderia glumae |
| 1 | Cellular orgnism | Burkholderia glumae BGR1 |
| 1 | Cellular orgnism | Burkholderia graminis |
| 1 | Cellular orgnism | Burkholderia graminis C4D1M |
| 1 | Cellular orgnism | Burkholderia mallei |
| 1 | Cellular orgnism | Burkholderia mallei 2002721280 |
| 1 | Cellular orgnism | Burkholderia mallei ATCC 10399 |
| 1 | Cellular orgnism | Burkholderia mallei ATCC 23344 |
| 1 | Cellular orgnism | Burkholderia mallei FMH |
| 1 | Cellular orgnism | Burkholderia mallei GB8 horse 4 |
| 1 | Cellular orgnism | Burkholderia mallei JHU |
| 1 | Cellular orgnism | Burkholderia mallei NCTC 10229 |
| 1 | Cellular orgnism | Burkholderia mallei NCTC 10247 |
| 1 | Cellular orgnism | Burkholderia mallei PRL-20 |
| 1 | Cellular orgnism | Burkholderia mallei SAVP1 |
| 1 | Cellular orgnism | Burkholderia multivorans |
| 1 | Cellular orgnism | Burkholderia multivorans ATCC 17616 |
| 1 | Cellular orgnism | Burkholderia multivorans CGD1 |
| 1 | Cellular orgnism | Burkholderia multivorans CGD2 |
| 1 | Cellular orgnism | Burkholderia multivorans CGD2M |
| 1 | Cellular orgnism | Burkholderia oklahomensis C6786 |
| 1 | Cellular orgnism | Burkholderia oklahomensis EO147 |
| 1 | Cellular orgnism | Burkholderia phymatum |
| 1 | Cellular orgnism | Burkholderia phymatum STM815 |
| 1 | Cellular orgnism | Burkholderia phytofirmans |
| 1 | Cellular orgnism | Burkholderia phytofirmans PsJN |
| 1 | Cellular orgnism | Burkholderia pseudomallei |
| 1 | Cellular orgnism | Burkholderia pseudomallei 1106a |
| 1 | Cellular orgnism | Burkholderia pseudomallei 1106b |
| 1 | Cellular orgnism | Burkholderia pseudomallei 112 |
| 1 | Cellular orgnism | Burkholderia pseudomallei 14 |
| 1 | Cellular orgnism | Burkholderia pseudomallei 1655 |
| 1 | Cellular orgnism | Burkholderia pseudomallei 1710a |
| 1 | Cellular orgnism | Burkholderia pseudomallei 1710b |
| 1 | Cellular orgnism | Burkholderia pseudomallei 305 |
| 1 | Cellular orgnism | Burkholderia pseudomallei 406e |
| 1 | Cellular orgnism | Burkholderia pseudomallei 576 |
| 1 | Cellular orgnism | Burkholderia pseudomallei 668 |
| 1 | Cellular orgnism | Burkholderia pseudomallei 7894 |
| 1 | Cellular orgnism | Burkholderia pseudomallei 9 |
| 1 | Cellular orgnism | Burkholderia pseudomallei 91 |
| 1 | Cellular orgnism | Burkholderia pseudomallei B7210 |
| 1 | Cellular orgnism | Burkholderia pseudomallei BCC215 |
| 1 | Cellular orgnism | Burkholderia pseudomallei DM98 |
| 1 | Cellular orgnism | Burkholderia pseudomallei K96243 |
| 1 | Cellular orgnism | Burkholderia pseudomallei MSHR346 |
| 1 | Cellular orgnism | Burkholderia pseudomallei NCTC 13177 |
| 1 | Cellular orgnism | Burkholderia pseudomallei Pakistan 9 |
| 1 | Cellular orgnism | Burkholderia pseudomallei Pasteur 52237 |
| 1 | Cellular orgnism | Burkholderia pseudomallei S13 |
| 1 | Cellular orgnism | Burkholderia rhizoxinica |
| 1 | Cellular orgnism | Burkholderia rhizoxinica HKI 454 |
| 1 | Cellular orgnism | Burkholderia sp. 383 |
| 1 | Cellular orgnism | Burkholderia sp. CCGE1001 |
| 1 | Cellular orgnism | Burkholderia sp. CCGE1002 |
| 1 | Cellular orgnism | Burkholderia sp. CCGE1003 |
| 1 | Cellular orgnism | Burkholderia sp. Ch1-1 |
| 1 | Cellular orgnism | Burkholderia sp. H160 |
| 1 | Cellular orgnism | Burkholderia thailandensis |
| 1 | Cellular orgnism | Burkholderia thailandensis Bt4 |
| 1 | Cellular orgnism | Burkholderia thailandensis E264 |
| 1 | Cellular orgnism | Burkholderia thailandensis MSMB43 |
| 1 | Cellular orgnism | Burkholderia thailandensis TXDOH |
| 1 | Cellular orgnism | Burkholderia ubonensis |
| 1 | Cellular orgnism | Burkholderia ubonensis Bu |
| 1 | Cellular orgnism | Burkholderia vietnamiensis |
| 1 | Cellular orgnism | Burkholderia vietnamiensis G4 |
| 1 | Cellular orgnism | Burkholderia xenovorans |
| 1 | Cellular orgnism | Burkholderia xenovorans LB400 |
| 1 | Cellular orgnism | Burkholderiales bacterium 1_1_47 |
| 1 | Cellular orgnism | Butyrivibrio crossotus DSM 2876 |
| 1 | Cellular orgnism | Butyrivibrio fibrisolvens |
| 1 | Cellular orgnism | Butyrivibrio fibrisolvens OB157 |
| 1 | Cellular orgnism | Butyrivibrio proteoclasticus B316 |
| 1 | Cellular orgnism | Caedibacter taeniospiralis |
| 1 | Cellular orgnism | Caldicellulosiruptor bescii DSM 6725 |
| 1 | Cellular orgnism | Caldicellulosiruptor lactoaceticus |
| 1 | Cellular orgnism | Caldicellulosiruptor obsidiansis OB47 |
| 1 | Cellular orgnism | Caldicellulosiruptor saccharolyticus |
| 1 | Cellular orgnism | Caldicellulosiruptor saccharolyticus DSM 8903 |
| 1 | Cellular orgnism | Caminibacter mediatlanticus |
| 1 | Cellular orgnism | Caminibacter mediatlanticus TB-2 |
| 1 | Cellular orgnism | Campylobacter coli |
| 1 | Cellular orgnism | Campylobacter coli JV20 |
| 1 | Cellular orgnism | Campylobacter coli RM2228 |
| 1 | Cellular orgnism | Campylobacter concisus |
| 1 | Cellular orgnism | Campylobacter concisus 13826 |
| 1 | Cellular orgnism | Campylobacter curvus |
| 1 | Cellular orgnism | Campylobacter curvus 525.92 |
| 1 | Cellular orgnism | Campylobacter fetus |
| 1 | Cellular orgnism | Campylobacter fetus subsp. fetus |
| 1 | Cellular orgnism | Campylobacter fetus subsp. fetus 82-40 |
| 1 | Cellular orgnism | Campylobacter fetus subsp. venerealis |
| 1 | Cellular orgnism | Campylobacter fetus subsp. venerealis str. Azul-94 |
| 1 | Cellular orgnism | Campylobacter gracilis |
| 1 | Cellular orgnism | Campylobacter gracilis RM3268 |
| 1 | Cellular orgnism | Campylobacter hominis |
| 1 | Cellular orgnism | Campylobacter hominis ATCC BAA-381 |
| 1 | Cellular orgnism | Campylobacter jejuni |
| 1 | Cellular orgnism | Campylobacter jejuni RM1221 |
| 1 | Cellular orgnism | Campylobacter jejuni subsp. doylei |
| 1 | Cellular orgnism | Campylobacter jejuni subsp. doylei 269.97 |
| 1 | Cellular orgnism | Campylobacter jejuni subsp. jejuni |
| 1 | Cellular orgnism | Campylobacter jejuni subsp. jejuni 1336 |
| 1 | Cellular orgnism | Campylobacter jejuni subsp. jejuni 260.94 |
| 1 | Cellular orgnism | Campylobacter jejuni subsp. jejuni 414 |
| 1 | Cellular orgnism | Campylobacter jejuni subsp. jejuni 81-176 |
| 1 | Cellular orgnism | Campylobacter jejuni subsp. jejuni 81116 |
| 1 | Cellular orgnism | Campylobacter jejuni subsp. jejuni 84-25 |
| 1 | Cellular orgnism | Campylobacter jejuni subsp. jejuni BH-01-0142 |
| 1 | Cellular orgnism | Campylobacter jejuni subsp. jejuni CF93-6 |
| 1 | Cellular orgnism | Campylobacter jejuni subsp. jejuni CG8421 |
| 1 | Cellular orgnism | Campylobacter jejuni subsp. jejuni CG8486 |
| 1 | Cellular orgnism | Campylobacter jejuni subsp. jejuni HB93-13 |
| 1 | Cellular orgnism | Campylobacter jejuni subsp. jejuni NCTC 11168 |
| 1 | Cellular orgnism | Campylobacter lari |
| 1 | Cellular orgnism | Campylobacter lari RM2100 |
| 1 | Cellular orgnism | Campylobacter rectus |
| 1 | Cellular orgnism | Campylobacter rectus RM3267 |
| 1 | Cellular orgnism | Campylobacter showae |
| 1 | Cellular orgnism | Campylobacter showae RM3277 |
| 1 | Cellular orgnism | Campylobacter upsaliensis |
| 1 | Cellular orgnism | Campylobacter upsaliensis RM3195 |
| 1 | Cellular orgnism | Campylobacterales bacterium GD 1 |
| 1 | Cellular orgnism | Candidatus Accumulibacter phosphatis clade IIA str. UW-1 |
| 1 | Cellular orgnism | Candidatus Amoebophilus asiaticus |
| 1 | Cellular orgnism | Candidatus Amoebophilus asiaticus 5a2 |
| 1 | Cellular orgnism | Candidatus Azobacteroides pseudotrichonymphae genomovar. CFP2 |
| 1 | Cellular orgnism | Candidatus Baumannia cicadellinicola |
| 1 | Cellular orgnism | Candidatus Blochmannia floridanus |
| 1 | Cellular orgnism | Candidatus Blochmannia pennsylvanicus |
| 1 | Cellular orgnism | Candidatus Blochmannia pennsylvanicus str. BPEN |
| 1 | Cellular orgnism | Candidatus Blochmannia vafer |
| 1 | Cellular orgnism | Candidatus Carsonella ruddii |
| 1 | Cellular orgnism | Candidatus Carsonella ruddii PV |
| 1 | Cellular orgnism | Candidatus Cloacamonas acidaminovorans |
| 1 | Cellular orgnism | Candidatus Desulforudis audaxviator MP104C |
| 1 | Cellular orgnism | Candidatus Hamiltonella defensa |
| 1 | Cellular orgnism | Candidatus Hamiltonella defensa 5AT (Acyrthosiphon pisum) |
| 1 | Cellular orgnism | Candidatus Hodgkinia cicadicola Dsem |
| 1 | Cellular orgnism | Candidatus Koribacter versatilis Ellin345 |
| 1 | Cellular orgnism | Candidatus Liberibacter asiaticus |
| 1 | Cellular orgnism | Candidatus Liberibacter asiaticus str. psy62 |
| 1 | Cellular orgnism | Candidatus Liberibacter solanacearum |
| 1 | Cellular orgnism | Candidatus Nitrospira defluvii |
| 1 | Cellular orgnism | Candidatus Pelagibacter sp. HTCC7211 |
| 1 | Cellular orgnism | Candidatus Pelagibacter ubique |
| 1 | Cellular orgnism | Candidatus Pelagibacter ubique HTCC1002 |
| 1 | Cellular orgnism | Candidatus Pelagibacter ubique HTCC1062 |
| 1 | Cellular orgnism | Candidatus Phytoplasma australiense |
| 1 | Cellular orgnism | Candidatus Phytoplasma mali |
| 1 | Cellular orgnism | Candidatus Poribacteria sp. WGA-A3 |
| 1 | Cellular orgnism | Candidatus Protochlamydia amoebophila UWE25 |
| 1 | Cellular orgnism | Candidatus Puniceispirillum marinum IMCC1322 |
| 1 | Cellular orgnism | Candidatus Regiella insecticola |
| 1 | Cellular orgnism | Candidatus Regiella insecticola LSR1 |
| 1 | Cellular orgnism | Candidatus Rickettsia amblyommii |
| 1 | Cellular orgnism | Candidatus Riesia pediculicola |
| 1 | Cellular orgnism | Candidatus Riesia pediculicola USDA |
| 1 | Cellular orgnism | Candidatus Ruthia magnifica str. Cm (Calyptogena magnifica) |
| 1 | Cellular orgnism | Candidatus Solibacter usitatus Ellin6076 |
| 1 | Cellular orgnism | Candidatus Sulcia muelleri CARI |
| 1 | Cellular orgnism | Candidatus Sulcia muelleri DMIN |
| 1 | Cellular orgnism | Candidatus Sulcia muelleri GWSS |
| 1 | Cellular orgnism | Candidatus Sulcia muelleri SMDSEM |
| 1 | Cellular orgnism | Candidatus Sulcia muelleri str. Hc (Homalodisca coagulata) |
| 1 | Cellular orgnism | Candidatus Vesicomyosocius okutanii HA |
| 1 | Cellular orgnism | Candidatus Zinderia insecticola CARI |
| 1 | Cellular orgnism | Capnocytophaga canimorsus |
| 1 | Cellular orgnism | Capnocytophaga gingivalis |
| 1 | Cellular orgnism | Capnocytophaga gingivalis ATCC 33624 |
| 1 | Cellular orgnism | Capnocytophaga ochracea |
| 1 | Cellular orgnism | Capnocytophaga ochracea DSM 7271 |
| 1 | Cellular orgnism | Capnocytophaga sputigena |
| 1 | Cellular orgnism | Carboxydibrachium pacificum DSM 12653 |
| 1 | Cellular orgnism | Carboxydothermus hydrogenoformans |
| 1 | Cellular orgnism | Carboxydothermus hydrogenoformans Z-2901 |
| 1 | Cellular orgnism | Cardiobacterium hominis ATCC 15826 |
| 1 | Cellular orgnism | Carnobacterium sp. AT7 |
| 1 | Cellular orgnism | Catenibacterium mitsuokai DSM 15897 |
| 1 | Cellular orgnism | Catenulispora acidiphila DSM 44928 |
| 1 | Cellular orgnism | Catonella morbi ATCC 51271 |
| 1 | Cellular orgnism | Caulobacter crescentus CB15 |
| 1 | Cellular orgnism | Caulobacter crescentus NA1000 |
| 1 | Cellular orgnism | Caulobacter segnis |
| 1 | Cellular orgnism | Caulobacter segnis ATCC 21756 |
| 1 | Cellular orgnism | Caulobacter sp. K31 |
| 1 | Cellular orgnism | Caulobacter vibrioides |
| 1 | Cellular orgnism | Cellulomonas flavigena |
| 1 | Cellular orgnism | Cellulomonas flavigena DSM 20109 |
| 1 | Cellular orgnism | Cellvibrio japonicus |
| 1 | Cellular orgnism | Cellvibrio japonicus Ueda107 |
| 1 | Cellular orgnism | Chelativorans sp. BNC1 |
| 1 | Cellular orgnism | Chitinophaga pinensis |
| 1 | Cellular orgnism | Chitinophaga pinensis DSM 2588 |
| 1 | Cellular orgnism | Chlamydia muridarum MopnTet14 |
| 1 | Cellular orgnism | Chlamydia muridarum Nigg |
| 1 | Cellular orgnism | Chlamydia muridarum Weiss |
| 1 | Cellular orgnism | Chlamydia trachomatis |
| 1 | Cellular orgnism | Chlamydia trachomatis 434/Bu |
| 1 | Cellular orgnism | Chlamydia trachomatis 6276 |
| 1 | Cellular orgnism | Chlamydia trachomatis 6276s |
| 1 | Cellular orgnism | Chlamydia trachomatis 70 |
| 1 | Cellular orgnism | Chlamydia trachomatis 70s |
| 1 | Cellular orgnism | Chlamydia trachomatis A/HAR-13 |
| 1 | Cellular orgnism | Chlamydia trachomatis B/Jali20/OT |
| 1 | Cellular orgnism | Chlamydia trachomatis B/TZ1A828/OT |
| 1 | Cellular orgnism | Chlamydia trachomatis D(s)2923 |
| 1 | Cellular orgnism | Chlamydia trachomatis D/UW-3/CX |
| 1 | Cellular orgnism | Chlamydia trachomatis L2b/UCH-1/proctitis |
| 1 | Cellular orgnism | Chlamydia trachomatis L2tet1 |
| 1 | Cellular orgnism | Chlamydophila abortus |
| 1 | Cellular orgnism | Chlamydophila abortus S26/3 |
| 1 | Cellular orgnism | Chlamydophila caviae |
| 1 | Cellular orgnism | Chlamydophila caviae GPIC |
| 1 | Cellular orgnism | Chlamydophila felis |
| 1 | Cellular orgnism | Chlamydophila felis Fe/C-56 |
| 1 | Cellular orgnism | Chlamydophila pneumoniae |
| 1 | Cellular orgnism | Chlamydophila pneumoniae AR39 |
| 1 | Cellular orgnism | Chlamydophila pneumoniae CWL029 |
| 1 | Cellular orgnism | Chlamydophila pneumoniae J138 |
| 1 | Cellular orgnism | Chlamydophila pneumoniae TW-183 |
| 1 | Cellular orgnism | Chlamydophila psittaci |
| 1 | Cellular orgnism | Chlorobaculum parvum |
| 1 | Cellular orgnism | Chlorobaculum parvum NCIB 8327 |
| 1 | Cellular orgnism | Chlorobaculum tepidum |
| 1 | Cellular orgnism | Chlorobium chlorochromatii CaD3 |
| 1 | Cellular orgnism | Chlorobium ferrooxidans DSM 13031 |
| 1 | Cellular orgnism | Chlorobium limicola |
| 1 | Cellular orgnism | Chlorobium limicola DSM 245 |
| 1 | Cellular orgnism | Chlorobium luteolum DSM 273 |
| 1 | Cellular orgnism | Chlorobium phaeobacteroides |
| 1 | Cellular orgnism | Chlorobium phaeobacteroides BS1 |
| 1 | Cellular orgnism | Chlorobium phaeobacteroides DSM 266 |
| 1 | Cellular orgnism | Chlorobium phaeovibrioides |
| 1 | Cellular orgnism | Chlorobium phaeovibrioides DSM 265 |
| 1 | Cellular orgnism | Chlorobium tepidum TLS |
| 1 | Cellular orgnism | Chloroflexus aggregans DSM 9485 |
| 1 | Cellular orgnism | Chloroflexus aurantiacus |
| 1 | Cellular orgnism | Chloroflexus aurantiacus J-10-fl |
| 1 | Cellular orgnism | Chloroflexus sp. Y-400-fl |
| 1 | Cellular orgnism | Chloroherpeton thalassium |
| 1 | Cellular orgnism | Chloroherpeton thalassium ATCC 35110 |
| 1 | Cellular orgnism | Chromobacterium violaceum |
| 1 | Cellular orgnism | Chromobacterium violaceum ATCC 12472 |
| 1 | Cellular orgnism | Chromohalobacter salexigens |
| 1 | Cellular orgnism | Chromohalobacter salexigens DSM 3043 |
| 1 | Cellular orgnism | Chryseobacterium gleum |
| 1 | Cellular orgnism | Chryseobacterium gleum ATCC 35910 |
| 1 | Cellular orgnism | Chthoniobacter flavus Ellin428 |
| 1 | Cellular orgnism | Citreicella sp. SE45 |
| 1 | Cellular orgnism | Citrobacter freundii |
| 1 | Cellular orgnism | Citrobacter koseri |
| 1 | Cellular orgnism | Citrobacter koseri ATCC BAA-895 |
| 1 | Cellular orgnism | Citrobacter rodentium |
| 1 | Cellular orgnism | Citrobacter rodentium ICC168 |
| 1 | Cellular orgnism | Citrobacter sp. 30_2 |
| 1 | Cellular orgnism | Citrobacter youngae |
| 1 | Cellular orgnism | Citrobacter youngae ATCC 29220 |
| 1 | Cellular orgnism | Citromicrobium bathyomarinum JL354 |
| 1 | Cellular orgnism | Clavibacter michiganensis |
| 1 | Cellular orgnism | Clavibacter michiganensis subsp. michiganensis |
| 1 | Cellular orgnism | Clavibacter michiganensis subsp. michiganensis NCPPB 382 |
| 1 | Cellular orgnism | Clavibacter michiganensis subsp. sepedonicus |
| 1 | Cellular orgnism | Clostridiales genomosp. BVAB3 str. UPII9-5 |
| 1 | Cellular orgnism | Clostridium acetobutylicum |
| 1 | Cellular orgnism | Clostridium acetobutylicum ATCC 824 |
| 1 | Cellular orgnism | Clostridium asparagiforme DSM 15981 |
| 1 | Cellular orgnism | Clostridium bartlettii DSM 16795 |
| 1 | Cellular orgnism | Clostridium beijerinckii |
| 1 | Cellular orgnism | Clostridium beijerinckii NCIMB 8052 |
| 1 | Cellular orgnism | Clostridium bolteae |
| 1 | Cellular orgnism | Clostridium bolteae ATCC BAA-613 |
| 1 | Cellular orgnism | Clostridium botulinum |
| 1 | Cellular orgnism | Clostridium botulinum A |
| 1 | Cellular orgnism | Clostridium botulinum A str. ATCC 19397 |
| 1 | Cellular orgnism | Clostridium botulinum A str. ATCC 3502 |
| 1 | Cellular orgnism | Clostridium botulinum A str. Hall |
| 1 | Cellular orgnism | Clostridium botulinum A2 str. Kyoto |
| 1 | Cellular orgnism | Clostridium botulinum A3 str. Loch Maree |
| 1 | Cellular orgnism | Clostridium botulinum B |
| 1 | Cellular orgnism | Clostridium botulinum B str. Eklund 17B |
| 1 | Cellular orgnism | Clostridium botulinum B1 str. Okra |
| 1 | Cellular orgnism | Clostridium botulinum Ba4 str. 657 |
| 1 | Cellular orgnism | Clostridium botulinum Bf |
| 1 | Cellular orgnism | Clostridium botulinum C |
| 1 | Cellular orgnism | Clostridium botulinum C str. Eklund |
| 1 | Cellular orgnism | Clostridium botulinum D |
| 1 | Cellular orgnism | Clostridium botulinum D str. 1873 |
| 1 | Cellular orgnism | Clostridium botulinum E |
| 1 | Cellular orgnism | Clostridium botulinum E1 str. 'BoNT E Beluga' |
| 1 | Cellular orgnism | Clostridium botulinum E3 str. Alaska E43 |
| 1 | Cellular orgnism | Clostridium botulinum F str. Langeland |
| 1 | Cellular orgnism | Clostridium botulinum NCTC 2916 |
| 1 | Cellular orgnism | Clostridium butyricum |
| 1 | Cellular orgnism | Clostridium butyricum 5521 |
| 1 | Cellular orgnism | Clostridium butyricum E4 str. BoNT E BL5262 |
| 1 | Cellular orgnism | Clostridium carboxidivorans P7 |
| 1 | Cellular orgnism | Clostridium cellulolyticum |
| 1 | Cellular orgnism | Clostridium cellulolyticum H10 |
| 1 | Cellular orgnism | Clostridium cellulovorans |
| 1 | Cellular orgnism | Clostridium cellulovorans 743B |
| 1 | Cellular orgnism | Clostridium difficile |
| 1 | Cellular orgnism | Clostridium difficile 630 |
| 1 | Cellular orgnism | Clostridium difficile ATCC 43255 |
| 1 | Cellular orgnism | Clostridium difficile CD196 |
| 1 | Cellular orgnism | Clostridium difficile CIP 107932 |
| 1 | Cellular orgnism | Clostridium difficile NAP07 |
| 1 | Cellular orgnism | Clostridium difficile NAP08 |
| 1 | Cellular orgnism | Clostridium difficile QCD-23m63 |
| 1 | Cellular orgnism | Clostridium difficile QCD-32g58 |
| 1 | Cellular orgnism | Clostridium difficile QCD-37x79 |
| 1 | Cellular orgnism | Clostridium difficile QCD-63q42 |
| 1 | Cellular orgnism | Clostridium difficile QCD-66c26 |
| 1 | Cellular orgnism | Clostridium difficile QCD-76w55 |
| 1 | Cellular orgnism | Clostridium difficile QCD-97b34 |
| 1 | Cellular orgnism | Clostridium difficile R20291 |
| 1 | Cellular orgnism | Clostridium hathewayi |
| 1 | Cellular orgnism | Clostridium hathewayi DSM 13479 |
| 1 | Cellular orgnism | Clostridium hiranonis DSM 13275 |
| 1 | Cellular orgnism | Clostridium hylemonae DSM 15053 |
| 1 | Cellular orgnism | Clostridium kluyveri |
| 1 | Cellular orgnism | Clostridium kluyveri DSM 555 |
| 1 | Cellular orgnism | Clostridium kluyveri NBRC 12016 |
| 1 | Cellular orgnism | Clostridium lentocellum DSM 5427 |
| 1 | Cellular orgnism | Clostridium leptum DSM 753 |
| 1 | Cellular orgnism | Clostridium ljungdahlii DSM 13528 |
| 1 | Cellular orgnism | Clostridium methylpentosum DSM 5476 |
| 1 | Cellular orgnism | Clostridium nexile DSM 1787 |
| 1 | Cellular orgnism | Clostridium novyi |
| 1 | Cellular orgnism | Clostridium novyi NT |
| 1 | Cellular orgnism | Clostridium papyrosolvens DSM 2782 |
| 1 | Cellular orgnism | Clostridium perfringens |
| 1 | Cellular orgnism | Clostridium perfringens ATCC 13124 |
| 1 | Cellular orgnism | Clostridium perfringens B |
| 1 | Cellular orgnism | Clostridium perfringens B str. ATCC 3626 |
| 1 | Cellular orgnism | Clostridium perfringens C |
| 1 | Cellular orgnism | Clostridium perfringens C str. JGS1495 |
| 1 | Cellular orgnism | Clostridium perfringens CPE str. F4969 |
| 1 | Cellular orgnism | Clostridium perfringens D |
| 1 | Cellular orgnism | Clostridium perfringens D str. JGS1721 |
| 1 | Cellular orgnism | Clostridium perfringens E str. JGS1987 |
| 1 | Cellular orgnism | Clostridium perfringens NCTC 8239 |
| 1 | Cellular orgnism | Clostridium perfringens SM101 |
| 1 | Cellular orgnism | Clostridium perfringens str. 13 |
| 1 | Cellular orgnism | Clostridium phytofermentans ISDg |
| 1 | Cellular orgnism | Clostridium ramosum |
| 1 | Cellular orgnism | Clostridium ramosum DSM 1402 |
| 1 | Cellular orgnism | Clostridium saccharolyticum WM1 |
| 1 | Cellular orgnism | Clostridium scindens |
| 1 | Cellular orgnism | Clostridium scindens ATCC 35704 |
| 1 | Cellular orgnism | Clostridium sp. 7_2_43FAA |
| 1 | Cellular orgnism | Clostridium sp. L2-50 |
| 1 | Cellular orgnism | Clostridium sp. M62/1 |
| 1 | Cellular orgnism | Clostridium sp. MCF-1 |
| 1 | Cellular orgnism | Clostridium sp. SS2/1 |
| 1 | Cellular orgnism | Clostridium spiroforme |
| 1 | Cellular orgnism | Clostridium spiroforme DSM 1552 |
| 1 | Cellular orgnism | Clostridium sporogenes |
| 1 | Cellular orgnism | Clostridium sporogenes ATCC 15579 |
| 1 | Cellular orgnism | Clostridium sticklandii |
| 1 | Cellular orgnism | Clostridium sticklandii DSM 519 |
| 1 | Cellular orgnism | Clostridium symbiosum |
| 1 | Cellular orgnism | Clostridium tetani |
| 1 | Cellular orgnism | Clostridium tetani E88 |
| 1 | Cellular orgnism | Clostridium thermocellum |
| 1 | Cellular orgnism | Clostridium thermocellum ATCC 27405 |
| 1 | Cellular orgnism | Clostridium thermocellum DSM 2360 |
| 1 | Cellular orgnism | Clostridium thermocellum JW20 |
| 1 | Cellular orgnism | Collimonas fungivorans |
| 1 | Cellular orgnism | Collinsella aerofaciens |
| 1 | Cellular orgnism | Collinsella aerofaciens ATCC 25986 |
| 1 | Cellular orgnism | Collinsella intestinalis DSM 13280 |
| 1 | Cellular orgnism | Collinsella stercoris DSM 13279 |
| 1 | Cellular orgnism | Colwellia psychrerythraea |
| 1 | Cellular orgnism | Colwellia psychrerythraea 34H |
| 1 | Cellular orgnism | Comamonas sp. CNB-1 |
| 1 | Cellular orgnism | Comamonas testosteroni |
| 1 | Cellular orgnism | Comamonas testosteroni CNB-2 |
| 1 | Cellular orgnism | Comamonas testosteroni KF-1 |
| 1 | Cellular orgnism | Comamonas testosteroni S44 |
| 1 | Cellular orgnism | Conexibacter woesei DSM 14684 |
| 1 | Cellular orgnism | Congregibacter litoralis KT71 |
| 1 | Cellular orgnism | Coprobacillus sp. D7 |
| 1 | Cellular orgnism | Coprococcus comes ATCC 27758 |
| 1 | Cellular orgnism | Coprococcus eutactus ATCC 27759 |
| 1 | Cellular orgnism | Coprothermobacter proteolyticus DSM 5265 |
| 1 | Cellular orgnism | Coraliomargarita akajimensis DSM 45221 |
| 1 | Cellular orgnism | Corynebacterium accolens |
| 1 | Cellular orgnism | Corynebacterium accolens ATCC 49725 |
| 1 | Cellular orgnism | Corynebacterium accolens ATCC 49726 |
| 1 | Cellular orgnism | Corynebacterium ammoniagenes |
| 1 | Cellular orgnism | Corynebacterium ammoniagenes DSM 20306 |
| 1 | Cellular orgnism | Corynebacterium amycolatum |
| 1 | Cellular orgnism | Corynebacterium amycolatum SK46 |
| 1 | Cellular orgnism | Corynebacterium aurimucosum |
| 1 | Cellular orgnism | Corynebacterium aurimucosum ATCC 700975 |
| 1 | Cellular orgnism | Corynebacterium callunae |
| 1 | Cellular orgnism | Corynebacterium casei |
| 1 | Cellular orgnism | Corynebacterium diphtheriae |
| 1 | Cellular orgnism | Corynebacterium diphtheriae NCTC 13129 |
| 1 | Cellular orgnism | Corynebacterium efficiens |
| 1 | Cellular orgnism | Corynebacterium efficiens YS-314 |
| 1 | Cellular orgnism | Corynebacterium genitalium ATCC 33030 |
| 1 | Cellular orgnism | Corynebacterium glucuronolyticum |
| 1 | Cellular orgnism | Corynebacterium glucuronolyticum ATCC 51866 |
| 1 | Cellular orgnism | Corynebacterium glucuronolyticum ATCC 51867 |
| 1 | Cellular orgnism | Corynebacterium glutamicum |
| 1 | Cellular orgnism | Corynebacterium glutamicum ATCC 13032 |
| 1 | Cellular orgnism | Corynebacterium glutamicum R |
| 1 | Cellular orgnism | Corynebacterium jeikeium |
| 1 | Cellular orgnism | Corynebacterium jeikeium ATCC 43734 |
| 1 | Cellular orgnism | Corynebacterium jeikeium K411 |
| 1 | Cellular orgnism | Corynebacterium kroppenstedtii DSM 44385 |
| 1 | Cellular orgnism | Corynebacterium lipophiloflavum |
| 1 | Cellular orgnism | Corynebacterium lipophiloflavum DSM 44291 |
| 1 | Cellular orgnism | Corynebacterium matruchotii |
| 1 | Cellular orgnism | Corynebacterium matruchotii ATCC 14266 |
| 1 | Cellular orgnism | Corynebacterium matruchotii ATCC 33806 |
| 1 | Cellular orgnism | Corynebacterium pseudogenitalium |
| 1 | Cellular orgnism | Corynebacterium pseudogenitalium ATCC 33035 |
| 1 | Cellular orgnism | Corynebacterium pseudotuberculosis |
| 1 | Cellular orgnism | Corynebacterium pseudotuberculosis FRC41 |
| 1 | Cellular orgnism | Corynebacterium renale |
| 1 | Cellular orgnism | Corynebacterium resistens DSM 45100 |
| 1 | Cellular orgnism | Corynebacterium sp. L2-79-05 |
| 1 | Cellular orgnism | Corynebacterium striatum |
| 1 | Cellular orgnism | Corynebacterium striatum ATCC 6940 |
| 1 | Cellular orgnism | Corynebacterium tuberculostearicum |
| 1 | Cellular orgnism | Corynebacterium tuberculostearicum SK141 |
| 1 | Cellular orgnism | Corynebacterium urealyticum |
| 1 | Cellular orgnism | Corynebacterium urealyticum DSM 7109 |
| 1 | Cellular orgnism | Corynebacterium variabile |
| 1 | Cellular orgnism | Coxiella burnetii |
| 1 | Cellular orgnism | Coxiella burnetii 'MSU Goat Q177' |
| 1 | Cellular orgnism | Coxiella burnetii CbuG_Q212 |
| 1 | Cellular orgnism | Coxiella burnetii CbuK_Q154 |
| 1 | Cellular orgnism | Coxiella burnetii Dugway 5J108-111 |
| 1 | Cellular orgnism | Coxiella burnetii RSA 331 |
| 1 | Cellular orgnism | Coxiella burnetii RSA 334 |
| 1 | Cellular orgnism | Coxiella burnetii RSA 493 |
| 1 | Cellular orgnism | Croceibacter atlanticus HTCC2559 |
| 1 | Cellular orgnism | Crocosphaera watsonii |
| 1 | Cellular orgnism | Crocosphaera watsonii WH 8501 |
| 1 | Cellular orgnism | Cronobacter sakazakii |
| 1 | Cellular orgnism | Cronobacter sakazakii ATCC BAA-894 |
| 1 | Cellular orgnism | Cronobacter turicensis |
| 1 | Cellular orgnism | Cronobacter turicensis z3032 |
| 1 | Cellular orgnism | Cryptobacterium curtum DSM 15641 |
| 1 | Cellular orgnism | Cupriavidus metallidurans |
| 1 | Cellular orgnism | Cupriavidus metallidurans CH34 |
| 1 | Cellular orgnism | Cupriavidus necator |
| 1 | Cellular orgnism | Cupriavidus taiwanensis |
| 1 | Cellular orgnism | Cyanobium sp. PCC 7001 |
| 1 | Cellular orgnism | Cyanothece sp. ATCC 51142 |
| 1 | Cellular orgnism | Cyanothece sp. CCY0110 |
| 1 | Cellular orgnism | Cyanothece sp. PCC 7424 |
| 1 | Cellular orgnism | Cyanothece sp. PCC 7425 |
| 1 | Cellular orgnism | Cyanothece sp. PCC 7822 |
| 1 | Cellular orgnism | Cyanothece sp. PCC 8801 |
| 1 | Cellular orgnism | Cyanothece sp. PCC 8802 |
| 1 | Cellular orgnism | Cylindrospermopsis raciborskii |
| 1 | Cellular orgnism | Cylindrospermopsis raciborskii CS-505 |
| 1 | Cellular orgnism | Cylindrospermum sp. A1345 |
| 1 | Cellular orgnism | Cytophaga hutchinsonii |
| 1 | Cellular orgnism | Cytophaga hutchinsonii ATCC 33406 |
| 1 | Cellular orgnism | Dechloromonas aromatica RCB |
| 1 | Cellular orgnism | Deferribacter desulfuricans SSM1 |
| 1 | Cellular orgnism | Dehalococcoides ethenogenes |
| 1 | Cellular orgnism | Dehalococcoides ethenogenes 195 |
| 1 | Cellular orgnism | Dehalococcoides sp. BAV1 |
| 1 | Cellular orgnism | Dehalococcoides sp. CBDB1 |
| 1 | Cellular orgnism | Dehalococcoides sp. GT |
| 1 | Cellular orgnism | Dehalococcoides sp. VS |
| 1 | Cellular orgnism | Dehalogenimonas lykanthroporepellens BL-DC-9 |
| 1 | Cellular orgnism | Deinococcus deserti |
| 1 | Cellular orgnism | Deinococcus deserti VCD115 |
| 1 | Cellular orgnism | Deinococcus geothermalis |
| 1 | Cellular orgnism | Deinococcus geothermalis DSM 11300 |
| 1 | Cellular orgnism | Deinococcus radiodurans |
| 1 | Cellular orgnism | Deinococcus radiodurans R1 |
| 1 | Cellular orgnism | Deinococcus radiopugnans ATCC 19172 |
| 1 | Cellular orgnism | Delftia acidovorans |
| 1 | Cellular orgnism | Delftia acidovorans SPH-1 |
| 1 | Cellular orgnism | Denitrovibrio acetiphilus DSM 12809 |
| 1 | Cellular orgnism | Dermacoccus sp. Ellin185 |
| 1 | Cellular orgnism | Desulfarculus baarsii DSM 2075 |
| 1 | Cellular orgnism | Desulfatibacillum alkenivorans |
| 1 | Cellular orgnism | Desulfatibacillum alkenivorans AK-01 |
| 1 | Cellular orgnism | Desulfitobacterium hafniense |
| 1 | Cellular orgnism | Desulfitobacterium hafniense DCB-2 |
| 1 | Cellular orgnism | Desulfitobacterium hafniense Y51 |
| 1 | Cellular orgnism | Desulfobacterium autotrophicum |
| 1 | Cellular orgnism | Desulfobacterium autotrophicum HRM2 |
| 1 | Cellular orgnism | Desulfobulbus propionicus |
| 1 | Cellular orgnism | Desulfobulbus propionicus DSM 2032 |
| 1 | Cellular orgnism | Desulfococcus oleovorans Hxd3 |
| 1 | Cellular orgnism | Desulfohalobium retbaense DSM 5692 |
| 1 | Cellular orgnism | Desulfomicrobium baculatum |
| 1 | Cellular orgnism | Desulfomicrobium baculatum DSM 4028 |
| 1 | Cellular orgnism | Desulfonatronospira thiodismutans ASO3-1 |
| 1 | Cellular orgnism | Desulfotalea psychrophila LSv54 |
| 1 | Cellular orgnism | Desulfotomaculum acetoxidans |
| 1 | Cellular orgnism | Desulfotomaculum acetoxidans DSM 771 |
| 1 | Cellular orgnism | Desulfotomaculum nigrificans |
| 1 | Cellular orgnism | Desulfotomaculum nigrificans DSM 574 |
| 1 | Cellular orgnism | Desulfotomaculum reducens MI-1 |
| 1 | Cellular orgnism | Desulfovibrio aespoeensis |
| 1 | Cellular orgnism | Desulfovibrio aespoeensis Aspo-2 |
| 1 | Cellular orgnism | Desulfovibrio desulfuricans |
| 1 | Cellular orgnism | Desulfovibrio desulfuricans subsp. desulfuricans |
| 1 | Cellular orgnism | Desulfovibrio desulfuricans subsp. desulfuricans str. ATCC 27774 |
| 1 | Cellular orgnism | Desulfovibrio desulfuricans subsp. desulfuricans str. G20 |
| 1 | Cellular orgnism | Desulfovibrio fructosovorans |
| 1 | Cellular orgnism | Desulfovibrio fructosovorans JJ |
| 1 | Cellular orgnism | Desulfovibrio magneticus RS-1 |
| 1 | Cellular orgnism | Desulfovibrio piger |
| 1 | Cellular orgnism | Desulfovibrio piger ATCC 29098 |
| 1 | Cellular orgnism | Desulfovibrio salexigens |
| 1 | Cellular orgnism | Desulfovibrio salexigens DSM 2638 |
| 1 | Cellular orgnism | Desulfovibrio sp. 3_1_syn3 |
| 1 | Cellular orgnism | Desulfovibrio sp. FW1012B |
| 1 | Cellular orgnism | Desulfovibrio vulgaris |
| 1 | Cellular orgnism | Desulfovibrio vulgaris DP4 |
| 1 | Cellular orgnism | Desulfovibrio vulgaris str. 'Miyazaki F' |
| 1 | Cellular orgnism | Desulfovibrio vulgaris str. Hildenborough |
| 1 | Cellular orgnism | Desulfurivibrio alkaliphilus AHT2 |
| 1 | Cellular orgnism | Desulfuromonas acetoxidans |
| 1 | Cellular orgnism | Desulfuromonas acetoxidans DSM 684 |
| 1 | Cellular orgnism | Dethiobacter alkaliphilus AHT 1 |
| 1 | Cellular orgnism | Dethiosulfovibrio peptidovorans DSM 11002 |
| 1 | Cellular orgnism | Dialister invisus DSM 15470 |
| 1 | Cellular orgnism | Dialister micraerophilus |
| 1 | Cellular orgnism | Dichelobacter nodosus |
| 1 | Cellular orgnism | Dichelobacter nodosus VCS1703A |
| 1 | Cellular orgnism | Dickeya dadantii |
| 1 | Cellular orgnism | Dickeya dadantii 3937 |
| 1 | Cellular orgnism | Dickeya dadantii Ech586 |
| 1 | Cellular orgnism | Dickeya dadantii Ech703 |
| 1 | Cellular orgnism | Dickeya zeae |
| 1 | Cellular orgnism | Dickeya zeae Ech1591 |
| 1 | Cellular orgnism | Dictyoglomus thermophilum |
| 1 | Cellular orgnism | Dictyoglomus thermophilum H-6-12 |
| 1 | Cellular orgnism | Dictyoglomus turgidum DSM 6724 |
| 1 | Cellular orgnism | Dietzia cinnamea |
| 1 | Cellular orgnism | Dinoroseobacter shibae DFL 12 |
| 1 | Cellular orgnism | Dokdonia donghaensis |
| 1 | Cellular orgnism | Dokdonia donghaensis MED134 |
| 1 | Cellular orgnism | Dorea formicigenerans ATCC 27755 |
| 1 | Cellular orgnism | Dorea longicatena DSM 13814 |
| 1 | Cellular orgnism | Dyadobacter fermentans DSM 18053 |
| 1 | Cellular orgnism | Edwardsiella ictaluri |
| 1 | Cellular orgnism | Edwardsiella ictaluri 93-146 |
| 1 | Cellular orgnism | Edwardsiella tarda |
| 1 | Cellular orgnism | Edwardsiella tarda ATCC 23685 |
| 1 | Cellular orgnism | Edwardsiella tarda EIB202 |
| 1 | Cellular orgnism | Eggerthella lenta |
| 1 | Cellular orgnism | Eggerthella lenta DSM 2243 |
| 1 | Cellular orgnism | Ehrlichia canis |
| 1 | Cellular orgnism | Ehrlichia canis str. Jake |
| 1 | Cellular orgnism | Ehrlichia chaffeensis |
| 1 | Cellular orgnism | Ehrlichia chaffeensis str. Arkansas |
| 1 | Cellular orgnism | Ehrlichia chaffeensis str. Sapulpa |
| 1 | Cellular orgnism | Ehrlichia ruminantium str. Gardel |
| 1 | Cellular orgnism | Ehrlichia ruminantium str. Welgevonden |
| 1 | Cellular orgnism | Eikenella corrodens |
| 1 | Cellular orgnism | Eikenella corrodens ATCC 23834 |
| 1 | Cellular orgnism | Elusimicrobium minutum Pei191 |
| 1 | Cellular orgnism | Endoriftia persephone 'Hot96_1+Hot96_2' |
| 1 | Cellular orgnism | Enhydrobacter aerosaccus SK60 |
| 1 | Cellular orgnism | Enterobacter aerogenes |
| 1 | Cellular orgnism | Enterobacter cancerogenus |
| 1 | Cellular orgnism | Enterobacter cancerogenus ATCC 35316 |
| 1 | Cellular orgnism | Enterobacter cloacae |
| 1 | Cellular orgnism | Enterobacter cloacae SCF1 |
| 1 | Cellular orgnism | Enterobacter cloacae subsp. cloacae |
| 1 | Cellular orgnism | Enterobacter cloacae subsp. cloacae ATCC 13047 |
| 1 | Cellular orgnism | Enterobacter sp. 638 |
| 1 | Cellular orgnism | Enterobacter sp. RFL1396 |
| 1 | Cellular orgnism | Enterococcus casseliflavus |
| 1 | Cellular orgnism | Enterococcus casseliflavus EC10 |
| 1 | Cellular orgnism | Enterococcus casseliflavus EC20 |
| 1 | Cellular orgnism | Enterococcus casseliflavus EC30 |
| 1 | Cellular orgnism | Enterococcus faecalis |
| 1 | Cellular orgnism | Enterococcus faecalis ATCC 29200 |
| 1 | Cellular orgnism | Enterococcus faecalis ATCC 4200 |
| 1 | Cellular orgnism | Enterococcus faecalis CH188 |
| 1 | Cellular orgnism | Enterococcus faecalis D6 |
| 1 | Cellular orgnism | Enterococcus faecalis DAPTO 512 |
| 1 | Cellular orgnism | Enterococcus faecalis DS5 |
| 1 | Cellular orgnism | Enterococcus faecalis E1Sol |
| 1 | Cellular orgnism | Enterococcus faecalis Fly1 |
| 1 | Cellular orgnism | Enterococcus faecalis HH22 |
| 1 | Cellular orgnism | Enterococcus faecalis HIP11704 |
| 1 | Cellular orgnism | Enterococcus faecalis JH1 |
| 1 | Cellular orgnism | Enterococcus faecalis Merz96 |
| 1 | Cellular orgnism | Enterococcus faecalis OG1RF |
| 1 | Cellular orgnism | Enterococcus faecalis PC1.1 |
| 1 | Cellular orgnism | Enterococcus faecalis R712 |
| 1 | Cellular orgnism | Enterococcus faecalis S613 |
| 1 | Cellular orgnism | Enterococcus faecalis T1 |
| 1 | Cellular orgnism | Enterococcus faecalis T11 |
| 1 | Cellular orgnism | Enterococcus faecalis T2 |
| 1 | Cellular orgnism | Enterococcus faecalis T3 |
| 1 | Cellular orgnism | Enterococcus faecalis T8 |
| 1 | Cellular orgnism | Enterococcus faecalis TUSoD Ef11 |
| 1 | Cellular orgnism | Enterococcus faecalis TX0102 |
| 1 | Cellular orgnism | Enterococcus faecalis TX0104 |
| 1 | Cellular orgnism | Enterococcus faecalis TX0109 |
| 1 | Cellular orgnism | Enterococcus faecalis TX0411 |
| 1 | Cellular orgnism | Enterococcus faecalis TX0635 |
| 1 | Cellular orgnism | Enterococcus faecalis TX0855 |
| 1 | Cellular orgnism | Enterococcus faecalis TX0860 |
| 1 | Cellular orgnism | Enterococcus faecalis TX1322 |
| 1 | Cellular orgnism | Enterococcus faecalis TX2134 |
| 1 | Cellular orgnism | Enterococcus faecalis TX4248 |
| 1 | Cellular orgnism | Enterococcus faecalis V583 |
| 1 | Cellular orgnism | Enterococcus faecalis X98 |
| 1 | Cellular orgnism | Enterococcus faecium |
| 1 | Cellular orgnism | Enterococcus faecium 1,141,733 |
| 1 | Cellular orgnism | Enterococcus faecium 1,230,933 |
| 1 | Cellular orgnism | Enterococcus faecium 1,231,408 |
| 1 | Cellular orgnism | Enterococcus faecium 1,231,410 |
| 1 | Cellular orgnism | Enterococcus faecium 1,231,501 |
| 1 | Cellular orgnism | Enterococcus faecium 1,231,502 |
| 1 | Cellular orgnism | Enterococcus faecium C68 |
| 1 | Cellular orgnism | Enterococcus faecium Com12 |
| 1 | Cellular orgnism | Enterococcus faecium Com15 |
| 1 | Cellular orgnism | Enterococcus faecium D344SRF |
| 1 | Cellular orgnism | Enterococcus faecium DO |
| 1 | Cellular orgnism | Enterococcus faecium E1039 |
| 1 | Cellular orgnism | Enterococcus faecium E1071 |
| 1 | Cellular orgnism | Enterococcus faecium E1162 |
| 1 | Cellular orgnism | Enterococcus faecium E1636 |
| 1 | Cellular orgnism | Enterococcus faecium E1679 |
| 1 | Cellular orgnism | Enterococcus faecium E980 |
| 1 | Cellular orgnism | Enterococcus faecium PC4.1 |
| 1 | Cellular orgnism | Enterococcus faecium TC 6 |
| 1 | Cellular orgnism | Enterococcus faecium TX1330 |
| 1 | Cellular orgnism | Enterococcus faecium U0317 |
| 1 | Cellular orgnism | Enterococcus gallinarum |
| 1 | Cellular orgnism | Enterococcus gallinarum EG2 |
| 1 | Cellular orgnism | Enterococcus italicus |
| 1 | Cellular orgnism | Epulopiscium sp. 'N.t. morphotype B' |
| 1 | Cellular orgnism | Erwinia amylovora |
| 1 | Cellular orgnism | Erwinia amylovora ATCC 49946 |
| 1 | Cellular orgnism | Erwinia amylovora CFBP1430 |
| 1 | Cellular orgnism | Erwinia billingiae |
| 1 | Cellular orgnism | Erwinia billingiae Eb661 |
| 1 | Cellular orgnism | Erwinia pyrifoliae |
| 1 | Cellular orgnism | Erwinia pyrifoliae Ep1/96 |
| 1 | Cellular orgnism | Erwinia sp. Ejp 556 |
| 1 | Cellular orgnism | Erwinia tasmaniensis |
| 1 | Cellular orgnism | Erwinia tasmaniensis Et1/99 |
| 1 | Cellular orgnism | Erysipelothrix rhusiopathiae |
| 1 | Cellular orgnism | Erysipelothrix rhusiopathiae ATCC 19414 |
| 1 | Cellular orgnism | Erysipelotrichaceae bacterium 3_1_53 |
| 1 | Cellular orgnism | Erysipelotrichaceae bacterium 5_2_54FAA |
| 1 | Cellular orgnism | Erythrobacter litoralis |
| 1 | Cellular orgnism | Erythrobacter litoralis HTCC2594 |
| 1 | Cellular orgnism | Erythrobacter sp. NAP1 |
| 1 | Cellular orgnism | Erythrobacter sp. SD-21 |
| 1 | Cellular orgnism | Escherichia albertii |
| 1 | Cellular orgnism | Escherichia albertii TW07627 |
| 1 | Cellular orgnism | Escherichia coli |
| 1 | Cellular orgnism | Escherichia coli 'BL21-Gold(DE3)pLysS AG' |
| 1 | Cellular orgnism | Escherichia coli 101-1 |
| 1 | Cellular orgnism | Escherichia coli 1520 |
| 1 | Cellular orgnism | Escherichia coli 1827-70 |
| 1 | Cellular orgnism | Escherichia coli 536 |
| 1 | Cellular orgnism | Escherichia coli 53638 |
| 1 | Cellular orgnism | Escherichia coli 55989 |
| 1 | Cellular orgnism | Escherichia coli 83972 |
| 1 | Cellular orgnism | Escherichia coli APEC O1 |
| 1 | Cellular orgnism | Escherichia coli ATCC 8739 |
| 1 | Cellular orgnism | Escherichia coli B |
| 1 | Cellular orgnism | Escherichia coli B str. REL606 |
| 1 | Cellular orgnism | Escherichia coli B088 |
| 1 | Cellular orgnism | Escherichia coli B171 |
| 1 | Cellular orgnism | Escherichia coli B185 |
| 1 | Cellular orgnism | Escherichia coli B354 |
| 1 | Cellular orgnism | Escherichia coli B7A |
| 1 | Cellular orgnism | Escherichia coli BW2952 |
| 1 | Cellular orgnism | Escherichia coli CFT073 |
| 1 | Cellular orgnism | Escherichia coli E110019 |
| 1 | Cellular orgnism | Escherichia coli E22 |
| 1 | Cellular orgnism | Escherichia coli E24377A |
| 1 | Cellular orgnism | Escherichia coli ED1a |
| 1 | Cellular orgnism | Escherichia coli ETEC 1392/75 |
| 1 | Cellular orgnism | Escherichia coli ETEC H10407 |
| 1 | Cellular orgnism | Escherichia coli F11 |
| 1 | Cellular orgnism | Escherichia coli FVEC1302 |
| 1 | Cellular orgnism | Escherichia coli FVEC1412 |
| 1 | Cellular orgnism | Escherichia coli H299 |
| 1 | Cellular orgnism | Escherichia coli H591 |
| 1 | Cellular orgnism | Escherichia coli H736 |
| 1 | Cellular orgnism | Escherichia coli HS |
| 1 | Cellular orgnism | Escherichia coli IAI1 |
| 1 | Cellular orgnism | Escherichia coli IAI39 |
| 1 | Cellular orgnism | Escherichia coli K-12 |
| 1 | Cellular orgnism | Escherichia coli M605 |
| 1 | Cellular orgnism | Escherichia coli M718 |
| 1 | Cellular orgnism | Escherichia coli MS 107-1 |
| 1 | Cellular orgnism | Escherichia coli MS 115-1 |
| 1 | Cellular orgnism | Escherichia coli MS 116-1 |
| 1 | Cellular orgnism | Escherichia coli MS 119-7 |
| 1 | Cellular orgnism | Escherichia coli MS 124-1 |
| 1 | Cellular orgnism | Escherichia coli MS 145-7 |
| 1 | Cellular orgnism | Escherichia coli MS 146-1 |
| 1 | Cellular orgnism | Escherichia coli MS 175-1 |
| 1 | Cellular orgnism | Escherichia coli MS 182-1 |
| 1 | Cellular orgnism | Escherichia coli MS 185-1 |
| 1 | Cellular orgnism | Escherichia coli MS 187-1 |
| 1 | Cellular orgnism | Escherichia coli MS 196-1 |
| 1 | Cellular orgnism | Escherichia coli MS 198-1 |
| 1 | Cellular orgnism | Escherichia coli MS 200-1 |
| 1 | Cellular orgnism | Escherichia coli MS 21-1 |
| 1 | Cellular orgnism | Escherichia coli MS 45-1 |
| 1 | Cellular orgnism | Escherichia coli MS 69-1 |
| 1 | Cellular orgnism | Escherichia coli MS 78-1 |
| 1 | Cellular orgnism | Escherichia coli MS 84-1 |
| 1 | Cellular orgnism | Escherichia coli NC101 |
| 1 | Cellular orgnism | Escherichia coli O103:H2 |
| 1 | Cellular orgnism | Escherichia coli O103:H2 str. 12009 |
| 1 | Cellular orgnism | Escherichia coli O111:H- |
| 1 | Cellular orgnism | Escherichia coli O111:H- str. 11128 |
| 1 | Cellular orgnism | Escherichia coli O127:H6 |
| 1 | Cellular orgnism | Escherichia coli O127:H6 str. E2348/69 |
| 1 | Cellular orgnism | Escherichia coli O157:H7 |
| 1 | Cellular orgnism | Escherichia coli O157:H7 str. EC4024 |
| 1 | Cellular orgnism | Escherichia coli O157:H7 str. EC4042 |
| 1 | Cellular orgnism | Escherichia coli O157:H7 str. EC4045 |
| 1 | Cellular orgnism | Escherichia coli O157:H7 str. EC4076 |
| 1 | Cellular orgnism | Escherichia coli O157:H7 str. EC4113 |
| 1 | Cellular orgnism | Escherichia coli O157:H7 str. EC4115 |
| 1 | Cellular orgnism | Escherichia coli O157:H7 str. EC4196 |
| 1 | Cellular orgnism | Escherichia coli O157:H7 str. EC4206 |
| 1 | Cellular orgnism | Escherichia coli O157:H7 str. EC4401 |
| 1 | Cellular orgnism | Escherichia coli O157:H7 str. EC4486 |
| 1 | Cellular orgnism | Escherichia coli O157:H7 str. EC4501 |
| 1 | Cellular orgnism | Escherichia coli O157:H7 str. EC508 |
| 1 | Cellular orgnism | Escherichia coli O157:H7 str. EC869 |
| 1 | Cellular orgnism | Escherichia coli O157:H7 str. FRIK2000 |
| 1 | Cellular orgnism | Escherichia coli O157:H7 str. FRIK966 |
| 1 | Cellular orgnism | Escherichia coli O157:H7 str. Sakai |
| 1 | Cellular orgnism | Escherichia coli O157:H7 str. TW14359 |
| 1 | Cellular orgnism | Escherichia coli O157:H7 str. TW14588 |
| 1 | Cellular orgnism | Escherichia coli O26:H- |
| 1 | Cellular orgnism | Escherichia coli O26:H11 |
| 1 | Cellular orgnism | Escherichia coli O26:H11 str. 11368 |
| 1 | Cellular orgnism | Escherichia coli O55:H7 |
| 1 | Cellular orgnism | Escherichia coli O55:H7 str. CB9615 |
| 1 | Cellular orgnism | Escherichia coli OP50 |
| 1 | Cellular orgnism | Escherichia coli S88 |
| 1 | Cellular orgnism | Escherichia coli SE11 |
| 1 | Cellular orgnism | Escherichia coli SMS-3-5 |
| 1 | Cellular orgnism | Escherichia coli TA143 |
| 1 | Cellular orgnism | Escherichia coli TA206 |
| 1 | Cellular orgnism | Escherichia coli TA271 |
| 1 | Cellular orgnism | Escherichia coli TA280 |
| 1 | Cellular orgnism | Escherichia coli UMN026 |
| 1 | Cellular orgnism | Escherichia coli UTI89 |
| 1 | Cellular orgnism | Escherichia coli Vir68 |
| 1 | Cellular orgnism | Escherichia coli W |
| 1 | Cellular orgnism | Escherichia coli str. K-12 substr. DH10B |
| 1 | Cellular orgnism | Escherichia coli str. K-12 substr. MG1655 |
| 1 | Cellular orgnism | Escherichia coli str. K-12 substr. W3110 |
| 1 | Cellular orgnism | Escherichia fergusonii |
| 1 | Cellular orgnism | Escherichia fergusonii ATCC 35469 |
| 1 | Cellular orgnism | Escherichia sp. 1_1_43 |
| 1 | Cellular orgnism | Escherichia sp. 3_2_53FAA |
| 1 | Cellular orgnism | Escherichia sp. 4_1_40B |
| 1 | Cellular orgnism | Escherichia sp. Sflu5 |
| 1 | Cellular orgnism | Ethanoligenens harbinense |
| 1 | Cellular orgnism | Ethanoligenens harbinense YUAN-3 |
| 1 | Cellular orgnism | Eubacterium biforme DSM 3989 |
| 1 | Cellular orgnism | Eubacterium cellulosolvens |
| 1 | Cellular orgnism | Eubacterium dolichum DSM 3991 |
| 1 | Cellular orgnism | Eubacterium eligens ATCC 27750 |
| 1 | Cellular orgnism | Eubacterium hallii |
| 1 | Cellular orgnism | Eubacterium hallii DSM 3353 |
| 1 | Cellular orgnism | Eubacterium limosum |
| 1 | Cellular orgnism | Eubacterium limosum KIST612 |
| 1 | Cellular orgnism | Eubacterium rectale |
| 1 | Cellular orgnism | Eubacterium rectale ATCC 33656 |
| 1 | Cellular orgnism | Eubacterium saburreum |
| 1 | Cellular orgnism | Eubacterium saphenum ATCC 49989 |
| 1 | Cellular orgnism | Eubacterium siraeum DSM 15702 |
| 1 | Cellular orgnism | Eubacterium ventriosum |
| 1 | Cellular orgnism | Eubacterium ventriosum ATCC 27560 |
| 1 | Cellular orgnism | Eubacterium yurii subsp. margaretiae ATCC 43715 |
| 1 | Cellular orgnism | Exiguobacterium arabatum |
| 1 | Cellular orgnism | Exiguobacterium sibiricum |
| 1 | Cellular orgnism | Exiguobacterium sibiricum 255-15 |
| 1 | Cellular orgnism | Exiguobacterium sp. AT1b |
| 1 | Cellular orgnism | Faecalibacterium cf. prausnitzii KLE1255 |
| 1 | Cellular orgnism | Faecalibacterium prausnitzii |
| 1 | Cellular orgnism | Faecalibacterium prausnitzii A2-165 |
| 1 | Cellular orgnism | Faecalibacterium prausnitzii M21/2 |
| 1 | Cellular orgnism | Ferrimonas balearica |
| 1 | Cellular orgnism | Ferrimonas balearica DSM 9799 |
| 1 | Cellular orgnism | Fervidobacterium nodosum Rt17-B1 |
| 1 | Cellular orgnism | Fibrobacter succinogenes |
| 1 | Cellular orgnism | Fibrobacter succinogenes subsp. succinogenes S85 |
| 1 | Cellular orgnism | Finegoldia magna |
| 1 | Cellular orgnism | Finegoldia magna ACS-171-V-Col3 |
| 1 | Cellular orgnism | Finegoldia magna ATCC 29328 |
| 1 | Cellular orgnism | Finegoldia magna ATCC 53516 |
| 1 | Cellular orgnism | Finegoldia magna BVS033A4 |
| 1 | Cellular orgnism | Flavobacteria bacterium BAL38 |
| 1 | Cellular orgnism | Flavobacteria bacterium BBFL7 |
| 1 | Cellular orgnism | Flavobacteria bacterium MS024-2A |
| 1 | Cellular orgnism | Flavobacteria bacterium MS024-3C |
| 1 | Cellular orgnism | Flavobacteriaceae bacterium 3519-10 |
| 1 | Cellular orgnism | Flavobacteriales bacterium ALC-1 |
| 1 | Cellular orgnism | Flavobacterium johnsoniae |
| 1 | Cellular orgnism | Flavobacterium johnsoniae UW101 |
| 1 | Cellular orgnism | Flavobacterium psychrophilum |
| 1 | Cellular orgnism | Flavobacterium psychrophilum JIP02/86 |
| 1 | Cellular orgnism | Flavobacterium sp. |
| 1 | Cellular orgnism | Flavobacterium sp. KI723T1 |
| 1 | Cellular orgnism | Fluoribacter dumoffii |
| 1 | Cellular orgnism | Francisella novicida |
| 1 | Cellular orgnism | Francisella novicida FTG |
| 1 | Cellular orgnism | Francisella novicida GA99-3548 |
| 1 | Cellular orgnism | Francisella novicida GA99-3549 |
| 1 | Cellular orgnism | Francisella novicida U112 |
| 1 | Cellular orgnism | Francisella philomiragia |
| 1 | Cellular orgnism | Francisella philomiragia subsp. philomiragia ATCC 25015 |
| 1 | Cellular orgnism | Francisella philomiragia subsp. philomiragia ATCC 25017 |
| 1 | Cellular orgnism | Francisella tularensis |
| 1 | Cellular orgnism | Francisella tularensis subsp. holarctica |
| 1 | Cellular orgnism | Francisella tularensis subsp. holarctica 257 |
| 1 | Cellular orgnism | Francisella tularensis subsp. holarctica FSC022 |
| 1 | Cellular orgnism | Francisella tularensis subsp. holarctica FSC200 |
| 1 | Cellular orgnism | Francisella tularensis subsp. holarctica FTNF002-00 |
| 1 | Cellular orgnism | Francisella tularensis subsp. holarctica LVS |
| 1 | Cellular orgnism | Francisella tularensis subsp. holarctica OSU18 |
| 1 | Cellular orgnism | Francisella tularensis subsp. holarctica URFT1 |
| 1 | Cellular orgnism | Francisella tularensis subsp. mediasiatica |
| 1 | Cellular orgnism | Francisella tularensis subsp. mediasiatica FSC147 |
| 1 | Cellular orgnism | Francisella tularensis subsp. tularensis |
| 1 | Cellular orgnism | Francisella tularensis subsp. tularensis FSC033 |
| 1 | Cellular orgnism | Francisella tularensis subsp. tularensis FSC198 |
| 1 | Cellular orgnism | Francisella tularensis subsp. tularensis MA00-2987 |
| 1 | Cellular orgnism | Francisella tularensis subsp. tularensis SCHU S4 |
| 1 | Cellular orgnism | Francisella tularensis subsp. tularensis WY96-3418 |
| 1 | Cellular orgnism | Frankia alni |
| 1 | Cellular orgnism | Frankia alni ACN14a |
| 1 | Cellular orgnism | Frankia sp. CcI3 |
| 1 | Cellular orgnism | Frankia sp. CpI1 |
| 1 | Cellular orgnism | Frankia sp. EAN1pec |
| 1 | Cellular orgnism | Frankia sp. EUN1f |
| 1 | Cellular orgnism | Frankia sp. EuI1c |
| 1 | Cellular orgnism | Frankia symbiont of Datisca glomerata |
| 1 | Cellular orgnism | Fulvimarina pelagi HTCC2506 |
| 1 | Cellular orgnism | Fusobacterium gonidiaformans ATCC 25563 |
| 1 | Cellular orgnism | Fusobacterium mortiferum |
| 1 | Cellular orgnism | Fusobacterium mortiferum ATCC 9817 |
| 1 | Cellular orgnism | Fusobacterium nucleatum |
| 1 | Cellular orgnism | Fusobacterium nucleatum subsp. nucleatum |
| 1 | Cellular orgnism | Fusobacterium nucleatum subsp. nucleatum ATCC 23726 |
| 1 | Cellular orgnism | Fusobacterium nucleatum subsp. nucleatum ATCC 25586 |
| 1 | Cellular orgnism | Fusobacterium nucleatum subsp. polymorphum ATCC 10953 |
| 1 | Cellular orgnism | Fusobacterium nucleatum subsp. vincentii ATCC 49256 |
| 1 | Cellular orgnism | Fusobacterium periodonticum |
| 1 | Cellular orgnism | Fusobacterium periodonticum ATCC 33693 |
| 1 | Cellular orgnism | Fusobacterium sp. 1_1_41FAA |
| 1 | Cellular orgnism | Fusobacterium sp. 2_1_31 |
| 1 | Cellular orgnism | Fusobacterium sp. 3_1_27 |
| 1 | Cellular orgnism | Fusobacterium sp. 3_1_33 |
| 1 | Cellular orgnism | Fusobacterium sp. 3_1_36A2 |
| 1 | Cellular orgnism | Fusobacterium sp. 3_1_5R |
| 1 | Cellular orgnism | Fusobacterium sp. 4_1_13 |
| 1 | Cellular orgnism | Fusobacterium sp. 7_1 |
| 1 | Cellular orgnism | Fusobacterium sp. D11 |
| 1 | Cellular orgnism | Fusobacterium sp. D12 |
| 1 | Cellular orgnism | Fusobacterium ulcerans ATCC 49185 |
| 1 | Cellular orgnism | Fusobacterium varium |
| 1 | Cellular orgnism | Fusobacterium varium ATCC 27725 |
| 1 | Cellular orgnism | Gallionella capsiferriformans ES-2 |
| 1 | Cellular orgnism | Gardnerella vaginalis |
| 1 | Cellular orgnism | Gardnerella vaginalis 409-05 |
| 1 | Cellular orgnism | Gardnerella vaginalis 5-1 |
| 1 | Cellular orgnism | Gardnerella vaginalis AMD |
| 1 | Cellular orgnism | Gardnerella vaginalis ATCC 14018 |
| 1 | Cellular orgnism | Gardnerella vaginalis ATCC 14019 |
| 1 | Cellular orgnism | Gemella haemolysans |
| 1 | Cellular orgnism | Gemella haemolysans ATCC 10379 |
| 1 | Cellular orgnism | Gemella morbillorum |
| 1 | Cellular orgnism | Gemmata obscuriglobus UQM 2246 |
| 1 | Cellular orgnism | Gemmatimonas aurantiaca T-27 |
| 1 | Cellular orgnism | Geobacillus kaustophilus |
| 1 | Cellular orgnism | Geobacillus kaustophilus HTA426 |
| 1 | Cellular orgnism | Geobacillus sp. C56-T3 |
| 1 | Cellular orgnism | Geobacillus sp. G11MC16 |
| 1 | Cellular orgnism | Geobacillus sp. WCH70 |
| 1 | Cellular orgnism | Geobacillus sp. Y4.1MC1 |
| 1 | Cellular orgnism | Geobacillus sp. Y412MC52 |
| 1 | Cellular orgnism | Geobacillus sp. Y412MC61 |
| 1 | Cellular orgnism | Geobacillus stearothermophilus |
| 1 | Cellular orgnism | Geobacillus thermodenitrificans |
| 1 | Cellular orgnism | Geobacillus thermodenitrificans NG80-2 |
| 1 | Cellular orgnism | Geobacillus thermoglucosidasius |
| 1 | Cellular orgnism | Geobacillus thermoglucosidasius C56-YS93 |
| 1 | Cellular orgnism | Geobacter bemidjiensis |
| 1 | Cellular orgnism | Geobacter bemidjiensis Bem |
| 1 | Cellular orgnism | Geobacter lovleyi SZ |
| 1 | Cellular orgnism | Geobacter metallireducens |
| 1 | Cellular orgnism | Geobacter metallireducens GS-15 |
| 1 | Cellular orgnism | Geobacter sp. FRC-32 |
| 1 | Cellular orgnism | Geobacter sp. M18 |
| 1 | Cellular orgnism | Geobacter sp. M21 |
| 1 | Cellular orgnism | Geobacter sulfurreducens |
| 1 | Cellular orgnism | Geobacter sulfurreducens PCA |
| 1 | Cellular orgnism | Geobacter uraniireducens Rf4 |
| 1 | Cellular orgnism | Geodermatophilus obscurus |
| 1 | Cellular orgnism | Geodermatophilus obscurus DSM 43160 |
| 1 | Cellular orgnism | Glaciecola sp. HTCC2999 |
| 1 | Cellular orgnism | Gloeobacter violaceus |
| 1 | Cellular orgnism | Gloeobacter violaceus PCC 7421 |
| 1 | Cellular orgnism | Gluconacetobacter diazotrophicus |
| 1 | Cellular orgnism | Gluconacetobacter diazotrophicus PAl 5 |
| 1 | Cellular orgnism | Gluconacetobacter hansenii |
| 1 | Cellular orgnism | Gluconacetobacter hansenii ATCC 23769 |
| 1 | Cellular orgnism | Gluconacetobacter xylinus |
| 1 | Cellular orgnism | Gluconacetobacter xylinus NBRC 3288 |
| 1 | Cellular orgnism | Gluconobacter oxydans |
| 1 | Cellular orgnism | Gluconobacter oxydans 621H |
| 1 | Cellular orgnism | Gordonia bronchialis |
| 1 | Cellular orgnism | Gordonia bronchialis DSM 43247 |
| 1 | Cellular orgnism | Gordonia westfalica |
| 1 | Cellular orgnism | Gramella forsetii KT0803 |
| 1 | Cellular orgnism | Granulibacter bethesdensis |
| 1 | Cellular orgnism | Granulibacter bethesdensis CGDNIH1 |
| 1 | Cellular orgnism | Granulicatella adiacens |
| 1 | Cellular orgnism | Granulicatella adiacens ATCC 49175 |
| 1 | Cellular orgnism | Granulicatella elegans |
| 1 | Cellular orgnism | Granulicatella elegans ATCC 700633 |
| 1 | Cellular orgnism | Grimontia hollisae |
| 1 | Cellular orgnism | Grimontia hollisae CIP 101886 |
| 1 | Cellular orgnism | Haemophilus ducreyi |
| 1 | Cellular orgnism | Haemophilus ducreyi 35000HP |
| 1 | Cellular orgnism | Haemophilus influenzae |
| 1 | Cellular orgnism | Haemophilus influenzae 22.1-21 |
| 1 | Cellular orgnism | Haemophilus influenzae 22.4-21 |
| 1 | Cellular orgnism | Haemophilus influenzae 3655 |
| 1 | Cellular orgnism | Haemophilus influenzae 6P18H1 |
| 1 | Cellular orgnism | Haemophilus influenzae 7P49H1 |
| 1 | Cellular orgnism | Haemophilus influenzae 86-028NP |
| 1 | Cellular orgnism | Haemophilus influenzae HK1212 |
| 1 | Cellular orgnism | Haemophilus influenzae NT127 |
| 1 | Cellular orgnism | Haemophilus influenzae PittAA |
| 1 | Cellular orgnism | Haemophilus influenzae PittEE |
| 1 | Cellular orgnism | Haemophilus influenzae PittGG |
| 1 | Cellular orgnism | Haemophilus influenzae PittHH |
| 1 | Cellular orgnism | Haemophilus influenzae PittII |
| 1 | Cellular orgnism | Haemophilus influenzae R3021 |
| 1 | Cellular orgnism | Haemophilus influenzae Rd KW20 |
| 1 | Cellular orgnism | Haemophilus influenzae RdAW |
| 1 | Cellular orgnism | Haemophilus influenzae biotype aegyptius |
| 1 | Cellular orgnism | Haemophilus parasuis |
| 1 | Cellular orgnism | Haemophilus parasuis 29755 |
| 1 | Cellular orgnism | Haemophilus parasuis SH0165 |
| 1 | Cellular orgnism | Haemophilus somnus 129PT |
| 1 | Cellular orgnism | Haemophilus somnus 2336 |
| 1 | Cellular orgnism | Hafnia alvei |
| 1 | Cellular orgnism | Hahella chejuensis KCTC 2396 |
| 1 | Cellular orgnism | Haliangium ochraceum DSM 14365 |
| 1 | Cellular orgnism | Halomonas elongata |
| 1 | Cellular orgnism | Halomonas elongata DSM 2581 |
| 1 | Cellular orgnism | Halorhodospira halophila |
| 1 | Cellular orgnism | Halorhodospira halophila SL1 |
| 1 | Cellular orgnism | Halothermothrix orenii |
| 1 | Cellular orgnism | Halothermothrix orenii H 168 |
| 1 | Cellular orgnism | Halothiobacillus neapolitanus |
| 1 | Cellular orgnism | Halothiobacillus neapolitanus c2 |
| 1 | Cellular orgnism | Helicobacter acinonychis |
| 1 | Cellular orgnism | Helicobacter acinonychis str. Sheeba |
| 1 | Cellular orgnism | Helicobacter bilis |
| 1 | Cellular orgnism | Helicobacter bilis ATCC 43879 |
| 1 | Cellular orgnism | Helicobacter canadensis |
| 1 | Cellular orgnism | Helicobacter canadensis MIT 98-5491 |
| 1 | Cellular orgnism | Helicobacter cinaedi |
| 1 | Cellular orgnism | Helicobacter cinaedi CCUG 18818 |
| 1 | Cellular orgnism | Helicobacter felis |
| 1 | Cellular orgnism | Helicobacter hepaticus |
| 1 | Cellular orgnism | Helicobacter hepaticus ATCC 51449 |
| 1 | Cellular orgnism | Helicobacter mustelae |
| 1 | Cellular orgnism | Helicobacter mustelae 12198 |
| 1 | Cellular orgnism | Helicobacter pullorum |
| 1 | Cellular orgnism | Helicobacter pullorum MIT 98-5489 |
| 1 | Cellular orgnism | Helicobacter pylori |
| 1 | Cellular orgnism | Helicobacter pylori 26695 |
| 1 | Cellular orgnism | Helicobacter pylori 98-10 |
| 1 | Cellular orgnism | Helicobacter pylori B128 |
| 1 | Cellular orgnism | Helicobacter pylori B38 |
| 1 | Cellular orgnism | Helicobacter pylori B8 |
| 1 | Cellular orgnism | Helicobacter pylori G27 |
| 1 | Cellular orgnism | Helicobacter pylori HPAG1 |
| 1 | Cellular orgnism | Helicobacter pylori HPKX_438_AG0C1 |
| 1 | Cellular orgnism | Helicobacter pylori HPKX_438_CA4C1 |
| 1 | Cellular orgnism | Helicobacter pylori J99 |
| 1 | Cellular orgnism | Helicobacter pylori P12 |
| 1 | Cellular orgnism | Helicobacter pylori PeCan4 |
| 1 | Cellular orgnism | Helicobacter pylori SJM180 |
| 1 | Cellular orgnism | Helicobacter pylori Shi470 |
| 1 | Cellular orgnism | Helicobacter suis |
| 1 | Cellular orgnism | Helicobacter suis HS1 |
| 1 | Cellular orgnism | Helicobacter winghamensis |
| 1 | Cellular orgnism | Helicobacter winghamensis ATCC BAA-430 |
| 1 | Cellular orgnism | Heliobacterium modesticaldum |
| 1 | Cellular orgnism | Heliobacterium modesticaldum Ice1 |
| 1 | Cellular orgnism | Herbaspirillum seropedicae |
| 1 | Cellular orgnism | Herbaspirillum seropedicae SmR1 |
| 1 | Cellular orgnism | Herminiimonas arsenicoxydans |
| 1 | Cellular orgnism | Herpetosiphon aurantiacus |
| 1 | Cellular orgnism | Herpetosiphon aurantiacus ATCC 23779 |
| 1 | Cellular orgnism | Hirschia baltica ATCC 49814 |
| 1 | Cellular orgnism | Histophilus somni |
| 1 | Cellular orgnism | Hoeflea phototrophica DFL-43 |
| 1 | Cellular orgnism | Holdemania filiformis DSM 12042 |
| 1 | Cellular orgnism | Hydrogenivirga sp. 128-5-R1-1 |
| 1 | Cellular orgnism | Hydrogenobacter thermophilus |
| 1 | Cellular orgnism | Hydrogenobacter thermophilus TK-6 |
| 1 | Cellular orgnism | Hydrogenobaculum sp. Y04AAS1 |
| 1 | Cellular orgnism | Hyphomicrobium denitrificans |
| 1 | Cellular orgnism | Hyphomicrobium denitrificans ATCC 51888 |
| 1 | Cellular orgnism | Hyphomonas neptunium |
| 1 | Cellular orgnism | Hyphomonas neptunium ATCC 15444 |
| 1 | Cellular orgnism | Idiomarina baltica |
| 1 | Cellular orgnism | Idiomarina baltica OS145 |
| 1 | Cellular orgnism | Idiomarina loihiensis L2TR |
| 1 | Cellular orgnism | Ilyobacter polytropus DSM 2926 |
| 1 | Cellular orgnism | Isosphaera pallida ATCC 43644 |
| 1 | Cellular orgnism | Janibacter sp. HTCC2649 |
| 1 | Cellular orgnism | Jannaschia sp. CCS1 |
| 1 | Cellular orgnism | Janthinobacterium sp. Marseille |
| 1 | Cellular orgnism | Jonesia denitrificans DSM 20603 |
| 1 | Cellular orgnism | Jonquetella anthropi E3_33 E1 |
| 1 | Cellular orgnism | Kangiella koreensis DSM 16069 |
| 1 | Cellular orgnism | Ketogulonicigenium vulgare |
| 1 | Cellular orgnism | Ketogulonicigenium vulgare Y25 |
| 1 | Cellular orgnism | Kineococcus radiotolerans SRS30216 |
| 1 | Cellular orgnism | Kingella oralis ATCC 51147 |
| 1 | Cellular orgnism | Klebsiella oxytoca |
| 1 | Cellular orgnism | Klebsiella oxytoca KOX105 |
| 1 | Cellular orgnism | Klebsiella pneumoniae |
| 1 | Cellular orgnism | Klebsiella pneumoniae 342 |
| 1 | Cellular orgnism | Klebsiella pneumoniae NTUH-K2044 |
| 1 | Cellular orgnism | Klebsiella pneumoniae subsp. pneumoniae |
| 1 | Cellular orgnism | Klebsiella pneumoniae subsp. pneumoniae MGH 78578 |
| 1 | Cellular orgnism | Klebsiella pneumoniae subsp. rhinoscleromatis |
| 1 | Cellular orgnism | Klebsiella pneumoniae subsp. rhinoscleromatis ATCC 13884 |
| 1 | Cellular orgnism | Klebsiella sp. 1_1_55 |
| 1 | Cellular orgnism | Klebsiella sp. KCL-2 |
| 1 | Cellular orgnism | Klebsiella variicola |
| 1 | Cellular orgnism | Klebsiella variicola At-22 |
| 1 | Cellular orgnism | Kocuria rhizophila |
| 1 | Cellular orgnism | Kocuria rhizophila DC2201 |
| 1 | Cellular orgnism | Kordia algicida OT-1 |
| 1 | Cellular orgnism | Kosmotoga olearia TBF 19.5.1 |
| 1 | Cellular orgnism | Kribbella flavida |
| 1 | Cellular orgnism | Kribbella flavida DSM 17836 |
| 1 | Cellular orgnism | Ktedonobacter racemifer DSM 44963 |
| 1 | Cellular orgnism | Kytococcus sedentarius |
| 1 | Cellular orgnism | Kytococcus sedentarius DSM 20547 |
| 1 | Cellular orgnism | Labrenzia aggregata |
| 1 | Cellular orgnism | Labrenzia alexandrii DFL-11 |
| 1 | Cellular orgnism | Lactobacillus acidipiscis |
| 1 | Cellular orgnism | Lactobacillus acidophilus |
| 1 | Cellular orgnism | Lactobacillus acidophilus ATCC 4796 |
| 1 | Cellular orgnism | Lactobacillus acidophilus NCFM |
| 1 | Cellular orgnism | Lactobacillus amylolyticus |
| 1 | Cellular orgnism | Lactobacillus amylolyticus DSM 11664 |
| 1 | Cellular orgnism | Lactobacillus amylovorus |
| 1 | Cellular orgnism | Lactobacillus antri |
| 1 | Cellular orgnism | Lactobacillus antri DSM 16041 |
| 1 | Cellular orgnism | Lactobacillus brevis |
| 1 | Cellular orgnism | Lactobacillus brevis ATCC 367 |
| 1 | Cellular orgnism | Lactobacillus brevis subsp. gravesensis ATCC 27305 |
| 1 | Cellular orgnism | Lactobacillus buchneri |
| 1 | Cellular orgnism | Lactobacillus buchneri ATCC 11577 |
| 1 | Cellular orgnism | Lactobacillus casei |
| 1 | Cellular orgnism | Lactobacillus casei ATCC 334 |
| 1 | Cellular orgnism | Lactobacillus casei BL23 |
| 1 | Cellular orgnism | Lactobacillus casei str. Zhang |
| 1 | Cellular orgnism | Lactobacillus coleohominis |
| 1 | Cellular orgnism | Lactobacillus coleohominis 101-4-CHN |
| 1 | Cellular orgnism | Lactobacillus crispatus |
| 1 | Cellular orgnism | Lactobacillus crispatus 125-2-CHN |
| 1 | Cellular orgnism | Lactobacillus crispatus 214-1 |
| 1 | Cellular orgnism | Lactobacillus crispatus JV-V01 |
| 1 | Cellular orgnism | Lactobacillus crispatus MV-1A-US |
| 1 | Cellular orgnism | Lactobacillus crispatus MV-3A-US |
| 1 | Cellular orgnism | Lactobacillus crispatus ST1 |
| 1 | Cellular orgnism | Lactobacillus curvatus |
| 1 | Cellular orgnism | Lactobacillus delbrueckii |
| 1 | Cellular orgnism | Lactobacillus delbrueckii subsp. bulgaricus |
| 1 | Cellular orgnism | Lactobacillus delbrueckii subsp. bulgaricus ATCC 11842 |
| 1 | Cellular orgnism | Lactobacillus delbrueckii subsp. bulgaricus ATCC BAA-365 |
| 1 | Cellular orgnism | Lactobacillus delbrueckii subsp. bulgaricus PB2003/044-T3-4 |
| 1 | Cellular orgnism | Lactobacillus delbrueckii subsp. lactis |
| 1 | Cellular orgnism | Lactobacillus farciminis |
| 1 | Cellular orgnism | Lactobacillus fermentum |
| 1 | Cellular orgnism | Lactobacillus fermentum 28-3-CHN |
| 1 | Cellular orgnism | Lactobacillus fermentum ATCC 14931 |
| 1 | Cellular orgnism | Lactobacillus fermentum IFO 3956 |
| 1 | Cellular orgnism | Lactobacillus gasseri |
| 1 | Cellular orgnism | Lactobacillus gasseri 202-4 |
| 1 | Cellular orgnism | Lactobacillus gasseri 224-1 |
| 1 | Cellular orgnism | Lactobacillus gasseri ATCC 33323 |
| 1 | Cellular orgnism | Lactobacillus gasseri JV-V03 |
| 1 | Cellular orgnism | Lactobacillus gasseri MV-22 |
| 1 | Cellular orgnism | Lactobacillus helveticus |
| 1 | Cellular orgnism | Lactobacillus helveticus DPC 4571 |
| 1 | Cellular orgnism | Lactobacillus helveticus DSM 20075 |
| 1 | Cellular orgnism | Lactobacillus helveticus subsp. jugurti |
| 1 | Cellular orgnism | Lactobacillus hilgardii |
| 1 | Cellular orgnism | Lactobacillus hilgardii ATCC 8290 |
| 1 | Cellular orgnism | Lactobacillus iners |
| 1 | Cellular orgnism | Lactobacillus iners AB-1 |
| 1 | Cellular orgnism | Lactobacillus iners DSM 13335 |
| 1 | Cellular orgnism | Lactobacillus iners LactinV 01V1-a |
| 1 | Cellular orgnism | Lactobacillus iners LactinV 03V1-b |
| 1 | Cellular orgnism | Lactobacillus iners LactinV 09V1-c |
| 1 | Cellular orgnism | Lactobacillus iners LactinV 11V1-d |
| 1 | Cellular orgnism | Lactobacillus iners SPIN 2503V10-D |
| 1 | Cellular orgnism | Lactobacillus jensenii |
| 1 | Cellular orgnism | Lactobacillus jensenii 115-3-CHN |
| 1 | Cellular orgnism | Lactobacillus jensenii 1153 |
| 1 | Cellular orgnism | Lactobacillus jensenii 208-1 |
| 1 | Cellular orgnism | Lactobacillus jensenii 269-3 |
| 1 | Cellular orgnism | Lactobacillus jensenii 27-2-CHN |
| 1 | Cellular orgnism | Lactobacillus jensenii JV-V16 |
| 1 | Cellular orgnism | Lactobacillus jensenii SJ-7A-US |
| 1 | Cellular orgnism | Lactobacillus johnsonii |
| 1 | Cellular orgnism | Lactobacillus johnsonii ATCC 33200 |
| 1 | Cellular orgnism | Lactobacillus johnsonii FI9785 |
| 1 | Cellular orgnism | Lactobacillus johnsonii NCC 533 |
| 1 | Cellular orgnism | Lactobacillus oris |
| 1 | Cellular orgnism | Lactobacillus paracasei |
| 1 | Cellular orgnism | Lactobacillus paracasei TXW |
| 1 | Cellular orgnism | Lactobacillus paracasei subsp. paracasei |
| 1 | Cellular orgnism | Lactobacillus paracasei subsp. paracasei 8700:2 |
| 1 | Cellular orgnism | Lactobacillus paracasei subsp. paracasei ATCC 25302 |
| 1 | Cellular orgnism | Lactobacillus pentosus |
| 1 | Cellular orgnism | Lactobacillus plantarum |
| 1 | Cellular orgnism | Lactobacillus plantarum JDM1 |
| 1 | Cellular orgnism | Lactobacillus plantarum WCFS1 |
| 1 | Cellular orgnism | Lactobacillus plantarum subsp. plantarum |
| 1 | Cellular orgnism | Lactobacillus plantarum subsp. plantarum ATCC 14917 |
| 1 | Cellular orgnism | Lactobacillus plantarum subsp. plantarum ST-III |
| 1 | Cellular orgnism | Lactobacillus reuteri |
| 1 | Cellular orgnism | Lactobacillus reuteri 100-23 |
| 1 | Cellular orgnism | Lactobacillus reuteri CF48-3A |
| 1 | Cellular orgnism | Lactobacillus reuteri DSM 20016 |
| 1 | Cellular orgnism | Lactobacillus reuteri JCM 1112 |
| 1 | Cellular orgnism | Lactobacillus reuteri MM2-3 |
| 1 | Cellular orgnism | Lactobacillus reuteri MM4-1A |
| 1 | Cellular orgnism | Lactobacillus reuteri SD2112 |
| 1 | Cellular orgnism | Lactobacillus rhamnosus |
| 1 | Cellular orgnism | Lactobacillus rhamnosus GG |
| 1 | Cellular orgnism | Lactobacillus rhamnosus HN001 |
| 1 | Cellular orgnism | Lactobacillus rhamnosus LMS2-1 |
| 1 | Cellular orgnism | Lactobacillus rhamnosus Lc 705 |
| 1 | Cellular orgnism | Lactobacillus ruminis |
| 1 | Cellular orgnism | Lactobacillus ruminis ATCC 25644 |
| 1 | Cellular orgnism | Lactobacillus sakei |
| 1 | Cellular orgnism | Lactobacillus sakei subsp. sakei |
| 1 | Cellular orgnism | Lactobacillus sakei subsp. sakei 23K |
| 1 | Cellular orgnism | Lactobacillus salivarius |
| 1 | Cellular orgnism | Lactobacillus salivarius ACS-116-V-Col5a |
| 1 | Cellular orgnism | Lactobacillus salivarius ATCC 11741 |
| 1 | Cellular orgnism | Lactobacillus salivarius UCC118 |
| 1 | Cellular orgnism | Lactobacillus ultunensis |
| 1 | Cellular orgnism | Lactobacillus ultunensis DSM 16047 |
| 1 | Cellular orgnism | Lactobacillus vaginalis |
| 1 | Cellular orgnism | Lactobacillus vaginalis ATCC 49540 |
| 1 | Cellular orgnism | Lactococcus garvieae |
| 1 | Cellular orgnism | Lactococcus lactis |
| 1 | Cellular orgnism | Lactococcus lactis subsp. cremoris |
| 1 | Cellular orgnism | Lactococcus lactis subsp. cremoris MG1363 |
| 1 | Cellular orgnism | Lactococcus lactis subsp. cremoris SK11 |
| 1 | Cellular orgnism | Lactococcus lactis subsp. lactis |
| 1 | Cellular orgnism | Lactococcus lactis subsp. lactis Il1403 |
| 1 | Cellular orgnism | Lactococcus lactis subsp. lactis K214 |
| 1 | Cellular orgnism | Lactococcus lactis subsp. lactis KF147 |
| 1 | Cellular orgnism | Lactococcus lactis subsp. lactis bv. diacetylactis |
| 1 | Cellular orgnism | Laribacter hongkongensis |
| 1 | Cellular orgnism | Laribacter hongkongensis HLHK9 |
| 1 | Cellular orgnism | Lawsonia intracellularis |
| 1 | Cellular orgnism | Lawsonia intracellularis PHE/MN1-00 |
| 1 | Cellular orgnism | Leeuwenhoekiella blandensis MED217 |
| 1 | Cellular orgnism | Legionella drancourtii |
| 1 | Cellular orgnism | Legionella drancourtii LLAP12 |
| 1 | Cellular orgnism | Legionella longbeachae |
| 1 | Cellular orgnism | Legionella longbeachae D-4968 |
| 1 | Cellular orgnism | Legionella longbeachae NSW150 |
| 1 | Cellular orgnism | Legionella pneumophila |
| 1 | Cellular orgnism | Legionella pneumophila 2300/99 Alcoy |
| 1 | Cellular orgnism | Legionella pneumophila serogroup 1 |
| 1 | Cellular orgnism | Legionella pneumophila str. Corby |
| 1 | Cellular orgnism | Legionella pneumophila str. Lens |
| 1 | Cellular orgnism | Legionella pneumophila str. Paris |
| 1 | Cellular orgnism | Legionella pneumophila subsp. pneumophila |
| 1 | Cellular orgnism | Legionella pneumophila subsp. pneumophila str. Philadelphia 1 |
| 1 | Cellular orgnism | Leifsonia xyli |
| 1 | Cellular orgnism | Leifsonia xyli subsp. xyli |
| 1 | Cellular orgnism | Leifsonia xyli subsp. xyli str. CTCB07 |
| 1 | Cellular orgnism | Lentisphaera araneosa HTCC2155 |
| 1 | Cellular orgnism | Leptolyngbya boryana |
| 1 | Cellular orgnism | Leptolyngbya foveolarum |
| 1 | Cellular orgnism | Leptolyngbya sp. PCC 6402 |
| 1 | Cellular orgnism | Leptolyngbya valderiana BDU 20041 |
| 1 | Cellular orgnism | Leptospira biflexa |
| 1 | Cellular orgnism | Leptospira biflexa serovar Patoc |
| 1 | Cellular orgnism | Leptospira biflexa serovar Patoc strain 'Patoc 1 (Ames)' |
| 1 | Cellular orgnism | Leptospira biflexa serovar Patoc strain 'Patoc 1 (Paris)' |
| 1 | Cellular orgnism | Leptospira borgpetersenii |
| 1 | Cellular orgnism | Leptospira borgpetersenii serovar Hardjo-bovis |
| 1 | Cellular orgnism | Leptospira borgpetersenii serovar Hardjo-bovis JB197 |
| 1 | Cellular orgnism | Leptospira borgpetersenii serovar Hardjo-bovis L550 |
| 1 | Cellular orgnism | Leptospira interrogans |
| 1 | Cellular orgnism | Leptospira interrogans serovar Copenhageni |
| 1 | Cellular orgnism | Leptospira interrogans serovar Copenhageni str. Fiocruz L1-130 |
| 1 | Cellular orgnism | Leptospira interrogans serovar Lai |
| 1 | Cellular orgnism | Leptospira interrogans serovar Lai str. 56601 |
| 1 | Cellular orgnism | Leptospirillum ferrooxidans |
| 1 | Cellular orgnism | Leptothrix cholodnii |
| 1 | Cellular orgnism | Leptothrix cholodnii SP-6 |
| 1 | Cellular orgnism | Leptotrichia buccalis |
| 1 | Cellular orgnism | Leptotrichia buccalis C-1013-b |
| 1 | Cellular orgnism | Leptotrichia goodfellowii F0264 |
| 1 | Cellular orgnism | Leptotrichia hofstadii F0254 |
| 1 | Cellular orgnism | Leuconostoc citreum |
| 1 | Cellular orgnism | Leuconostoc citreum KM20 |
| 1 | Cellular orgnism | Leuconostoc gasicomitatum |
| 1 | Cellular orgnism | Leuconostoc gasicomitatum LMG 18811 |
| 1 | Cellular orgnism | Leuconostoc kimchii |
| 1 | Cellular orgnism | Leuconostoc kimchii IMSNU 11154 |
| 1 | Cellular orgnism | Leuconostoc mesenteroides |
| 1 | Cellular orgnism | Leuconostoc mesenteroides subsp. cremoris |
| 1 | Cellular orgnism | Leuconostoc mesenteroides subsp. cremoris ATCC 19254 |
| 1 | Cellular orgnism | Leuconostoc mesenteroides subsp. mesenteroides |
| 1 | Cellular orgnism | Leuconostoc mesenteroides subsp. mesenteroides ATCC 8293 |
| 1 | Cellular orgnism | Leuconostoc mesenteroides subsp. mesenteroides Y110 |
| 1 | Cellular orgnism | Limnobacter sp. MED105 |
| 1 | Cellular orgnism | Listeria grayi |
| 1 | Cellular orgnism | Listeria grayi DSM 20601 |
| 1 | Cellular orgnism | Listeria innocua |
| 1 | Cellular orgnism | Listeria innocua Clip11262 |
| 1 | Cellular orgnism | Listeria ivanovii |
| 1 | Cellular orgnism | Listeria marthii |
| 1 | Cellular orgnism | Listeria monocytogenes |
| 1 | Cellular orgnism | Listeria monocytogenes 08-5578 |
| 1 | Cellular orgnism | Listeria monocytogenes 08-5923 |
| 1 | Cellular orgnism | Listeria monocytogenes 10403S |
| 1 | Cellular orgnism | Listeria monocytogenes EGD-e |
| 1 | Cellular orgnism | Listeria monocytogenes F6900 |
| 1 | Cellular orgnism | Listeria monocytogenes FSL F2-515 |
| 1 | Cellular orgnism | Listeria monocytogenes FSL J1-175 |
| 1 | Cellular orgnism | Listeria monocytogenes FSL J1-194 |
| 1 | Cellular orgnism | Listeria monocytogenes FSL J1-208 |
| 1 | Cellular orgnism | Listeria monocytogenes FSL J2-003 |
| 1 | Cellular orgnism | Listeria monocytogenes FSL J2-064 |
| 1 | Cellular orgnism | Listeria monocytogenes FSL J2-071 |
| 1 | Cellular orgnism | Listeria monocytogenes FSL N1-017 |
| 1 | Cellular orgnism | Listeria monocytogenes FSL N3-165 |
| 1 | Cellular orgnism | Listeria monocytogenes FSL R2-503 |
| 1 | Cellular orgnism | Listeria monocytogenes FSL R2-561 |
| 1 | Cellular orgnism | Listeria monocytogenes Finland 1988 |
| 1 | Cellular orgnism | Listeria monocytogenes HCC23 |
| 1 | Cellular orgnism | Listeria monocytogenes HPB2262 |
| 1 | Cellular orgnism | Listeria monocytogenes J0161 |
| 1 | Cellular orgnism | Listeria monocytogenes J2818 |
| 1 | Cellular orgnism | Listeria monocytogenes LO28 |
| 1 | Cellular orgnism | Listeria monocytogenes serotype 4b str. F2365 |
| 1 | Cellular orgnism | Listeria seeligeri |
| 1 | Cellular orgnism | Listeria seeligeri serovar 1/2b str. SLCC3954 |
| 1 | Cellular orgnism | Listeria welshimeri |
| 1 | Cellular orgnism | Listeria welshimeri serovar 6b str. SLCC5334 |
| 1 | Cellular orgnism | Listonella anguillarum |
| 1 | Cellular orgnism | Listonella anguillarum 775 |
| 1 | Cellular orgnism | Listonella anguillarum serovar O2 |
| 1 | Cellular orgnism | Loktanella vestfoldensis |
| 1 | Cellular orgnism | Loktanella vestfoldensis SKA53 |
| 1 | Cellular orgnism | Lutiella nitroferrum 2002 |
| 1 | Cellular orgnism | Lyngbya sp. PCC 8106 |
| 1 | Cellular orgnism | Lysinibacillus fusiformis |
| 1 | Cellular orgnism | Lysinibacillus fusiformis ZC1 |
| 1 | Cellular orgnism | Lysinibacillus sphaericus |
| 1 | Cellular orgnism | Lysinibacillus sphaericus C3-41 |
| 1 | Cellular orgnism | Macrococcus caseolyticus |
| 1 | Cellular orgnism | Macrococcus caseolyticus JCSC5402 |
| 1 | Cellular orgnism | Magnetococcus sp. MC-1 |
| 1 | Cellular orgnism | Magnetospirillum gryphiswaldense |
| 1 | Cellular orgnism | Magnetospirillum gryphiswaldense MSR-1 |
| 1 | Cellular orgnism | Magnetospirillum magneticum |
| 1 | Cellular orgnism | Magnetospirillum magneticum AMB-1 |
| 1 | Cellular orgnism | Magnetospirillum magnetotacticum |
| 1 | Cellular orgnism | Magnetospirillum magnetotacticum MS-1 |
| 1 | Cellular orgnism | Mannheimia haemolytica |
| 1 | Cellular orgnism | Mannheimia haemolytica PHL213 |
| 1 | Cellular orgnism | Mannheimia haemolytica serotype A2 str. BOVINE |
| 1 | Cellular orgnism | Mannheimia haemolytica serotype A2 str. OVINE |
| 1 | Cellular orgnism | Mannheimia succiniciproducens MBEL55E |
| 1 | Cellular orgnism | Mannheimia varigena |
| 1 | Cellular orgnism | Maricaulis maris |
| 1 | Cellular orgnism | Maricaulis maris MCS10 |
| 1 | Cellular orgnism | Marinobacter algicola DG893 |
| 1 | Cellular orgnism | Marinobacter aquaeolei VT8 |
| 1 | Cellular orgnism | Marinobacter hydrocarbonoclasticus |
| 1 | Cellular orgnism | Marinobacter sp. ELB17 |
| 1 | Cellular orgnism | Marinococcus halophilus |
| 1 | Cellular orgnism | Marinomonas sp. MED121 |
| 1 | Cellular orgnism | Marinomonas sp. MWYL1 |
| 1 | Cellular orgnism | Mariprofundus ferrooxydans PV-1 |
| 1 | Cellular orgnism | Maritimibacter alkaliphilus HTCC2654 |
| 1 | Cellular orgnism | Megasphaera genomosp. type_1 str. 28L |
| 1 | Cellular orgnism | Megasphaera micronuciformis |
| 1 | Cellular orgnism | Megasphaera micronuciformis F0359 |
| 1 | Cellular orgnism | Meiothermus ruber |
| 1 | Cellular orgnism | Meiothermus ruber DSM 1279 |
| 1 | Cellular orgnism | Meiothermus silvanus DSM 9946 |
| 1 | Cellular orgnism | Mesoplasma florum |
| 1 | Cellular orgnism | Mesoplasma florum L1 |
| 1 | Cellular orgnism | Mesorhizobium ciceri |
| 1 | Cellular orgnism | Mesorhizobium ciceri biovar biserrulae |
| 1 | Cellular orgnism | Mesorhizobium loti |
| 1 | Cellular orgnism | Mesorhizobium loti MAFF303099 |
| 1 | Cellular orgnism | Mesorhizobium opportunistum |
| 1 | Cellular orgnism | Mesorhizobium opportunistum WSM2075 |
| 1 | Cellular orgnism | Methylacidiphilum infernorum V4 |
| 1 | Cellular orgnism | Methylibium petroleiphilum PM1 |
| 1 | Cellular orgnism | Methylobacillus flagellatus |
| 1 | Cellular orgnism | Methylobacillus flagellatus KT |
| 1 | Cellular orgnism | Methylobacter tundripaludum |
| 1 | Cellular orgnism | Methylobacter tundripaludum SV96 |
| 1 | Cellular orgnism | Methylobacterium chloromethanicum |
| 1 | Cellular orgnism | Methylobacterium chloromethanicum CM4 |
| 1 | Cellular orgnism | Methylobacterium extorquens |
| 1 | Cellular orgnism | Methylobacterium extorquens AM1 |
| 1 | Cellular orgnism | Methylobacterium extorquens DM4 |
| 1 | Cellular orgnism | Methylobacterium extorquens PA1 |
| 1 | Cellular orgnism | Methylobacterium nodulans |
| 1 | Cellular orgnism | Methylobacterium nodulans ORS 2060 |
| 1 | Cellular orgnism | Methylobacterium populi BJ001 |
| 1 | Cellular orgnism | Methylobacterium radiotolerans |
| 1 | Cellular orgnism | Methylobacterium radiotolerans JCM 2831 |
| 1 | Cellular orgnism | Methylobacterium sp. 4-46 |
| 1 | Cellular orgnism | Methylocella silvestris |
| 1 | Cellular orgnism | Methylocella silvestris BL2 |
| 1 | Cellular orgnism | Methylococcus capsulatus |
| 1 | Cellular orgnism | Methylococcus capsulatus str. Bath |
| 1 | Cellular orgnism | Methylocystis sp. ATCC 49242 |
| 1 | Cellular orgnism | Methylophaga thalassica |
| 1 | Cellular orgnism | Methylophilales bacterium HTCC2181 |
| 1 | Cellular orgnism | Methylosinus trichosporium |
| 1 | Cellular orgnism | Methylosinus trichosporium OB3b |
| 1 | Cellular orgnism | Methylotenera mobilis JLW8 |
| 1 | Cellular orgnism | Methylotenera sp. 301 |
| 1 | Cellular orgnism | Methylovorus sp. SIP3-4 |
| 1 | Cellular orgnism | Microbacterium testaceum |
| 1 | Cellular orgnism | Micrococcus luteus |
| 1 | Cellular orgnism | Micrococcus luteus NCTC 2665 |
| 1 | Cellular orgnism | Micrococcus luteus SK58 |
| 1 | Cellular orgnism | Micrococcus sp. 28 |
| 1 | Cellular orgnism | Microcoleus chthonoplastes |
| 1 | Cellular orgnism | Microcoleus chthonoplastes PCC 7420 |
| 1 | Cellular orgnism | Microcystis |
| 1 | Cellular orgnism | Microcystis aeruginosa |
| 1 | Cellular orgnism | Microcystis aeruginosa NIES-843 |
| 1 | Cellular orgnism | Micromonospora aurantiaca |
| 1 | Cellular orgnism | Micromonospora aurantiaca ATCC 27029 |
| 1 | Cellular orgnism | Micromonospora rosaria |
| 1 | Cellular orgnism | Micromonospora sp. ATCC 39149 |
| 1 | Cellular orgnism | Micromonospora sp. L5 |
| 1 | Cellular orgnism | Microscilla marina |
| 1 | Cellular orgnism | Microscilla marina ATCC 23134 |
| 1 | Cellular orgnism | Microscilla sp. PRE1 |
| 1 | Cellular orgnism | Mitsuokella multacida |
| 1 | Cellular orgnism | Mitsuokella multacida DSM 20544 |
| 1 | Cellular orgnism | Mobiluncus curtisii |
| 1 | Cellular orgnism | Mobiluncus curtisii ATCC 43063 |
| 1 | Cellular orgnism | Mobiluncus curtisii subsp. curtisii |
| 1 | Cellular orgnism | Mobiluncus curtisii subsp. curtisii ATCC 35241 |
| 1 | Cellular orgnism | Mobiluncus curtisii subsp. holmesii |
| 1 | Cellular orgnism | Mobiluncus mulieris 28-1 |
| 1 | Cellular orgnism | Mobiluncus mulieris ATCC 35239 |
| 1 | Cellular orgnism | Mobiluncus mulieris ATCC 35243 |
| 1 | Cellular orgnism | Mobiluncus mulieris FB024-16 |
| 1 | Cellular orgnism | Moorella thermoacetica |
| 1 | Cellular orgnism | Moorella thermoacetica ATCC 39073 |
| 1 | Cellular orgnism | Moraxella bovis |
| 1 | Cellular orgnism | Moraxella bovis Epp63 |
| 1 | Cellular orgnism | Moraxella catarrhalis |
| 1 | Cellular orgnism | Moraxella catarrhalis RH4 |
| 1 | Cellular orgnism | Moraxella sp. TA144 |
| 1 | Cellular orgnism | Moritella sp. PE36 |
| 1 | Cellular orgnism | Mycobacterium abscessus |
| 1 | Cellular orgnism | Mycobacterium abscessus ATCC 19977 |
| 1 | Cellular orgnism | Mycobacterium avium |
| 1 | Cellular orgnism | Mycobacterium avium 104 |
| 1 | Cellular orgnism | Mycobacterium avium subsp. avium |
| 1 | Cellular orgnism | Mycobacterium avium subsp. avium ATCC 25291 |
| 1 | Cellular orgnism | Mycobacterium avium subsp. paratuberculosis |
| 1 | Cellular orgnism | Mycobacterium avium subsp. paratuberculosis K-10 |
| 1 | Cellular orgnism | Mycobacterium bovis |
| 1 | Cellular orgnism | Mycobacterium bovis AF2122/97 |
| 1 | Cellular orgnism | Mycobacterium bovis BCG |
| 1 | Cellular orgnism | Mycobacterium bovis BCG str. Pasteur 1173P2 |
| 1 | Cellular orgnism | Mycobacterium bovis BCG str. Tokyo 172 |
| 1 | Cellular orgnism | Mycobacterium celatum |
| 1 | Cellular orgnism | Mycobacterium gilvum |
| 1 | Cellular orgnism | Mycobacterium gilvum PYR-GCK |
| 1 | Cellular orgnism | Mycobacterium intracellulare |
| 1 | Cellular orgnism | Mycobacterium intracellulare ATCC 13950 |
| 1 | Cellular orgnism | Mycobacterium kansasii |
| 1 | Cellular orgnism | Mycobacterium kansasii ATCC 12478 |
| 1 | Cellular orgnism | Mycobacterium leprae |
| 1 | Cellular orgnism | Mycobacterium leprae Br4923 |
| 1 | Cellular orgnism | Mycobacterium leprae TN |
| 1 | Cellular orgnism | Mycobacterium liflandii |
| 1 | Cellular orgnism | Mycobacterium liflandii 128FXT |
| 1 | Cellular orgnism | Mycobacterium marinum |
| 1 | Cellular orgnism | Mycobacterium marinum M |
| 1 | Cellular orgnism | Mycobacterium parascrofulaceum |
| 1 | Cellular orgnism | Mycobacterium parascrofulaceum ATCC BAA-614 |
| 1 | Cellular orgnism | Mycobacterium smegmatis |
| 1 | Cellular orgnism | Mycobacterium smegmatis str. MC2 155 |
| 1 | Cellular orgnism | Mycobacterium sp. JLS |
| 1 | Cellular orgnism | Mycobacterium sp. KMS |
| 1 | Cellular orgnism | Mycobacterium sp. MCS |
| 1 | Cellular orgnism | Mycobacterium sp. Spyr1 |
| 1 | Cellular orgnism | Mycobacterium tuberculosis |
| 1 | Cellular orgnism | Mycobacterium tuberculosis '98-R604 INH-RIF-EM' |
| 1 | Cellular orgnism | Mycobacterium tuberculosis 02_1987 |
| 1 | Cellular orgnism | Mycobacterium tuberculosis 210 |
| 1 | Cellular orgnism | Mycobacterium tuberculosis 94_M4241A |
| 1 | Cellular orgnism | Mycobacterium tuberculosis C |
| 1 | Cellular orgnism | Mycobacterium tuberculosis CDC1551 |
| 1 | Cellular orgnism | Mycobacterium tuberculosis CPHL_A |
| 1 | Cellular orgnism | Mycobacterium tuberculosis EAS054 |
| 1 | Cellular orgnism | Mycobacterium tuberculosis F11 |
| 1 | Cellular orgnism | Mycobacterium tuberculosis GM 1503 |
| 1 | Cellular orgnism | Mycobacterium tuberculosis H37Ra |
| 1 | Cellular orgnism | Mycobacterium tuberculosis H37Rv |
| 1 | Cellular orgnism | Mycobacterium tuberculosis K85 |
| 1 | Cellular orgnism | Mycobacterium tuberculosis KZN 1435 |
| 1 | Cellular orgnism | Mycobacterium tuberculosis KZN 4207 |
| 1 | Cellular orgnism | Mycobacterium tuberculosis KZN 605 |
| 1 | Cellular orgnism | Mycobacterium tuberculosis KZN R506 |
| 1 | Cellular orgnism | Mycobacterium tuberculosis KZN V2475 |
| 1 | Cellular orgnism | Mycobacterium tuberculosis SUMu001 |
| 1 | Cellular orgnism | Mycobacterium tuberculosis SUMu002 |
| 1 | Cellular orgnism | Mycobacterium tuberculosis SUMu003 |
| 1 | Cellular orgnism | Mycobacterium tuberculosis SUMu004 |
| 1 | Cellular orgnism | Mycobacterium tuberculosis SUMu005 |
| 1 | Cellular orgnism | Mycobacterium tuberculosis SUMu006 |
| 1 | Cellular orgnism | Mycobacterium tuberculosis SUMu007 |
| 1 | Cellular orgnism | Mycobacterium tuberculosis SUMu008 |
| 1 | Cellular orgnism | Mycobacterium tuberculosis SUMu009 |
| 1 | Cellular orgnism | Mycobacterium tuberculosis SUMu010 |
| 1 | Cellular orgnism | Mycobacterium tuberculosis SUMu011 |
| 1 | Cellular orgnism | Mycobacterium tuberculosis SUMu012 |
| 1 | Cellular orgnism | Mycobacterium tuberculosis T17 |
| 1 | Cellular orgnism | Mycobacterium tuberculosis T46 |
| 1 | Cellular orgnism | Mycobacterium tuberculosis T85 |
| 1 | Cellular orgnism | Mycobacterium tuberculosis T92 |
| 1 | Cellular orgnism | Mycobacterium tuberculosis str. Haarlem |
| 1 | Cellular orgnism | Mycobacterium ulcerans |
| 1 | Cellular orgnism | Mycobacterium ulcerans Agy99 |
| 1 | Cellular orgnism | Mycobacterium vanbaalenii |
| 1 | Cellular orgnism | Mycobacterium vanbaalenii PYR-1 |
| 1 | Cellular orgnism | Mycoplasma agalactiae |
| 1 | Cellular orgnism | Mycoplasma agalactiae PG2 |
| 1 | Cellular orgnism | Mycoplasma alligatoris A21JP2 |
| 1 | Cellular orgnism | Mycoplasma arthritidis |
| 1 | Cellular orgnism | Mycoplasma arthritidis 158L3-1 |
| 1 | Cellular orgnism | Mycoplasma bovis |
| 1 | Cellular orgnism | Mycoplasma capricolum |
| 1 | Cellular orgnism | Mycoplasma capricolum subsp. capricolum |
| 1 | Cellular orgnism | Mycoplasma capricolum subsp. capricolum ATCC 27343 |
| 1 | Cellular orgnism | Mycoplasma conjunctivae |
| 1 | Cellular orgnism | Mycoplasma conjunctivae HRC/581 |
| 1 | Cellular orgnism | Mycoplasma crocodyli MP145 |
| 1 | Cellular orgnism | Mycoplasma fermentans |
| 1 | Cellular orgnism | Mycoplasma fermentans JER |
| 1 | Cellular orgnism | Mycoplasma gallisepticum |
| 1 | Cellular orgnism | Mycoplasma gallisepticum str. R |
| 1 | Cellular orgnism | Mycoplasma gallisepticum str. R(low) |
| 1 | Cellular orgnism | Mycoplasma genitalium |
| 1 | Cellular orgnism | Mycoplasma genitalium G37 |
| 1 | Cellular orgnism | Mycoplasma haemofelis |
| 1 | Cellular orgnism | Mycoplasma hominis |
| 1 | Cellular orgnism | Mycoplasma hyopneumoniae |
| 1 | Cellular orgnism | Mycoplasma hyopneumoniae 232 |
| 1 | Cellular orgnism | Mycoplasma hyopneumoniae 7448 |
| 1 | Cellular orgnism | Mycoplasma hyopneumoniae J |
| 1 | Cellular orgnism | Mycoplasma hyorhinis |
| 1 | Cellular orgnism | Mycoplasma hyorhinis HUB-1 |
| 1 | Cellular orgnism | Mycoplasma leachii |
| 1 | Cellular orgnism | Mycoplasma mobile |
| 1 | Cellular orgnism | Mycoplasma mobile 163K |
| 1 | Cellular orgnism | Mycoplasma mycoides |
| 1 | Cellular orgnism | Mycoplasma mycoides subsp. mycoides |
| 1 | Cellular orgnism | Mycoplasma mycoides subsp. mycoides SC |
| 1 | Cellular orgnism | Mycoplasma mycoides subsp. mycoides SC str. PG1 |
| 1 | Cellular orgnism | Mycoplasma penetrans |
| 1 | Cellular orgnism | Mycoplasma penetrans HF-2 |
| 1 | Cellular orgnism | Mycoplasma pneumoniae |
| 1 | Cellular orgnism | Mycoplasma pneumoniae M129 |
| 1 | Cellular orgnism | Mycoplasma pulmonis |
| 1 | Cellular orgnism | Mycoplasma pulmonis UAB CTIP |
| 1 | Cellular orgnism | Mycoplasma suis |
| 1 | Cellular orgnism | Mycoplasma synoviae |
| 1 | Cellular orgnism | Mycoplasma synoviae 53 |
| 1 | Cellular orgnism | Mycoplasma yeatsii |
| 1 | Cellular orgnism | Myxococcus fulvus |
| 1 | Cellular orgnism | Myxococcus xanthus |
| 1 | Cellular orgnism | Myxococcus xanthus DK 1622 |
| 1 | Cellular orgnism | Nakamurella multipartita DSM 44233 |
| 1 | Cellular orgnism | Natranaerobius thermophilus JW/NM-WN-LF |
| 1 | Cellular orgnism | Nautilia profundicola AmH |
| 1 | Cellular orgnism | Neisseria cinerea |
| 1 | Cellular orgnism | Neisseria cinerea ATCC 14685 |
| 1 | Cellular orgnism | Neisseria elongata |
| 1 | Cellular orgnism | Neisseria elongata subsp. glycolytica ATCC 29315 |
| 1 | Cellular orgnism | Neisseria flavescens |
| 1 | Cellular orgnism | Neisseria flavescens NRL30031/H210 |
| 1 | Cellular orgnism | Neisseria flavescens SK114 |
| 1 | Cellular orgnism | Neisseria gonorrhoeae |
| 1 | Cellular orgnism | Neisseria gonorrhoeae 1291 |
| 1 | Cellular orgnism | Neisseria gonorrhoeae 35/02 |
| 1 | Cellular orgnism | Neisseria gonorrhoeae DGI18 |
| 1 | Cellular orgnism | Neisseria gonorrhoeae DGI2 |
| 1 | Cellular orgnism | Neisseria gonorrhoeae F62 |
| 1 | Cellular orgnism | Neisseria gonorrhoeae FA 1090 |
| 1 | Cellular orgnism | Neisseria gonorrhoeae FA19 |
| 1 | Cellular orgnism | Neisseria gonorrhoeae FA6140 |
| 1 | Cellular orgnism | Neisseria gonorrhoeae MS11 |
| 1 | Cellular orgnism | Neisseria gonorrhoeae NCCP11945 |
| 1 | Cellular orgnism | Neisseria gonorrhoeae PID1 |
| 1 | Cellular orgnism | Neisseria gonorrhoeae PID18 |
| 1 | Cellular orgnism | Neisseria gonorrhoeae PID24-1 |
| 1 | Cellular orgnism | Neisseria gonorrhoeae PID332 |
| 1 | Cellular orgnism | Neisseria gonorrhoeae SK-92-679 |
| 1 | Cellular orgnism | Neisseria gonorrhoeae SK-93-1035 |
| 1 | Cellular orgnism | Neisseria lactamica |
| 1 | Cellular orgnism | Neisseria lactamica ATCC 23970 |
| 1 | Cellular orgnism | Neisseria meningitidis |
| 1 | Cellular orgnism | Neisseria meningitidis 053442 |
| 1 | Cellular orgnism | Neisseria meningitidis ATCC 13091 |
| 1 | Cellular orgnism | Neisseria meningitidis FAM18 |
| 1 | Cellular orgnism | Neisseria meningitidis MC58 |
| 1 | Cellular orgnism | Neisseria meningitidis Z2491 |
| 1 | Cellular orgnism | Neisseria meningitidis alpha14 |
| 1 | Cellular orgnism | Neisseria meningitidis serogroup A |
| 1 | Cellular orgnism | Neisseria meningitidis serogroup B |
| 1 | Cellular orgnism | Neisseria meningitidis serogroup C |
| 1 | Cellular orgnism | Neisseria mucosa |
| 1 | Cellular orgnism | Neisseria mucosa ATCC 25996 |
| 1 | Cellular orgnism | Neisseria polysaccharea |
| 1 | Cellular orgnism | Neisseria polysaccharea ATCC 43768 |
| 1 | Cellular orgnism | Neisseria sicca |
| 1 | Cellular orgnism | Neisseria sicca ATCC 29256 |
| 1 | Cellular orgnism | Neisseria sp. oral taxon 014 str. F0314 |
| 1 | Cellular orgnism | Neisseria subflava |
| 1 | Cellular orgnism | Neisseria subflava NJ9703 |
| 1 | Cellular orgnism | Neorickettsia risticii str. Illinois |
| 1 | Cellular orgnism | Neorickettsia sennetsu |
| 1 | Cellular orgnism | Neorickettsia sennetsu str. Miyayama |
| 1 | Cellular orgnism | Nitratifractor salsuginis |
| 1 | Cellular orgnism | Nitratifractor salsuginis DSM 16511 |
| 1 | Cellular orgnism | Nitratiruptor sp. SB155-2 |
| 1 | Cellular orgnism | Nitrobacter hamburgensis |
| 1 | Cellular orgnism | Nitrobacter hamburgensis X14 |
| 1 | Cellular orgnism | Nitrobacter sp. Nb-311A |
| 1 | Cellular orgnism | Nitrobacter winogradskyi |
| 1 | Cellular orgnism | Nitrobacter winogradskyi Nb-255 |
| 1 | Cellular orgnism | Nitrococcus mobilis |
| 1 | Cellular orgnism | Nitrococcus mobilis Nb-231 |
| 1 | Cellular orgnism | Nitrosococcus halophilus |
| 1 | Cellular orgnism | Nitrosococcus halophilus Nc4 |
| 1 | Cellular orgnism | Nitrosococcus oceani |
| 1 | Cellular orgnism | Nitrosococcus oceani AFC27 |
| 1 | Cellular orgnism | Nitrosococcus oceani ATCC 19707 |
| 1 | Cellular orgnism | Nitrosococcus watsoni C-113 |
| 1 | Cellular orgnism | Nitrosomonas europaea |
| 1 | Cellular orgnism | Nitrosomonas europaea ATCC 19718 |
| 1 | Cellular orgnism | Nitrosomonas eutropha |
| 1 | Cellular orgnism | Nitrosomonas eutropha C91 |
| 1 | Cellular orgnism | Nitrosomonas sp. |
| 1 | Cellular orgnism | Nitrosomonas sp. AL212 |
| 1 | Cellular orgnism | Nitrosospira multiformis |
| 1 | Cellular orgnism | Nitrosospira multiformis ATCC 25196 |
| 1 | Cellular orgnism | Nocardia aobensis |
| 1 | Cellular orgnism | Nocardia farcinica |
| 1 | Cellular orgnism | Nocardia farcinica IFM 10152 |
| 1 | Cellular orgnism | Nocardia sp. 107 |
| 1 | Cellular orgnism | Nocardia sp. C-14-1 |
| 1 | Cellular orgnism | Nocardioides sp. JS614 |
| 1 | Cellular orgnism | Nocardiopsis dassonvillei |
| 1 | Cellular orgnism | Nocardiopsis dassonvillei subsp. dassonvillei |
| 1 | Cellular orgnism | Nocardiopsis dassonvillei subsp. dassonvillei DSM 43111 |
| 1 | Cellular orgnism | Nocardiopsis sp. 90127 |
| 1 | Cellular orgnism | Nodularia spumigena |
| 1 | Cellular orgnism | Nodularia spumigena CCY9414 |
| 1 | Cellular orgnism | Nostoc punctiforme |
| 1 | Cellular orgnism | Nostoc punctiforme PCC 73102 |
| 1 | Cellular orgnism | Nostoc sp. PCC 7120 |
| 1 | Cellular orgnism | Nostoc sp. PCC 7524 |
| 1 | Cellular orgnism | Novosphingobium aromaticivorans |
| 1 | Cellular orgnism | Novosphingobium aromaticivorans DSM 12444 |
| 1 | Cellular orgnism | Oceanibulbus indolifex |
| 1 | Cellular orgnism | Oceanibulbus indolifex HEL-45 |
| 1 | Cellular orgnism | Oceanicola batsensis |
| 1 | Cellular orgnism | Oceanicola batsensis HTCC2597 |
| 1 | Cellular orgnism | Oceanicola granulosus |
| 1 | Cellular orgnism | Oceanicola granulosus HTCC2516 |
| 1 | Cellular orgnism | Oceanobacillus iheyensis |
| 1 | Cellular orgnism | Oceanobacillus iheyensis HTE831 |
| 1 | Cellular orgnism | Ochrobactrum anthropi |
| 1 | Cellular orgnism | Ochrobactrum anthropi ATCC 49188 |
| 1 | Cellular orgnism | Ochrobactrum intermedium |
| 1 | Cellular orgnism | Ochrobactrum intermedium LMG 3301 |
| 1 | Cellular orgnism | Octadecabacter antarcticus |
| 1 | Cellular orgnism | Octadecabacter antarcticus 238 |
| 1 | Cellular orgnism | Octadecabacter antarcticus 307 |
| 1 | Cellular orgnism | Oenococcus oeni |
| 1 | Cellular orgnism | Oenococcus oeni ATCC BAA-1163 |
| 1 | Cellular orgnism | Oenococcus oeni AWRIB429 |
| 1 | Cellular orgnism | Oenococcus oeni PSU-1 |
| 1 | Cellular orgnism | Oligotropha carboxidovorans |
| 1 | Cellular orgnism | Oligotropha carboxidovorans OM5 |
| 1 | Cellular orgnism | Olsenella uli DSM 7084 |
| 1 | Cellular orgnism | Onion yellows phytoplasma |
| 1 | Cellular orgnism | Onion yellows phytoplasma OY-M |
| 1 | Cellular orgnism | Opitutaceae bacterium TAV2 |
| 1 | Cellular orgnism | Opitutus terrae |
| 1 | Cellular orgnism | Opitutus terrae PB90-1 |
| 1 | Cellular orgnism | Oribacterium sinus F0268 |
| 1 | Cellular orgnism | Oribacterium sp. oral taxon 078 str. F0262 |
| 1 | Cellular orgnism | Orientia tsutsugamushi |
| 1 | Cellular orgnism | Orientia tsutsugamushi str. Boryong |
| 1 | Cellular orgnism | Orientia tsutsugamushi str. Ikeda |
| 1 | Cellular orgnism | Ornithobacterium rhinotracheale |
| 1 | Cellular orgnism | Oscillatoria sp. PCC 6506 |
| 1 | Cellular orgnism | Oscillochloris trichoides |
| 1 | Cellular orgnism | Oxalobacter formigenes |
| 1 | Cellular orgnism | Oxalobacter formigenes HOxBLS |
| 1 | Cellular orgnism | Oxalobacter formigenes OXCC13 |
| 1 | Cellular orgnism | Paenibacillus curdlanolyticus |
| 1 | Cellular orgnism | Paenibacillus curdlanolyticus YK9 |
| 1 | Cellular orgnism | Paenibacillus larvae |
| 1 | Cellular orgnism | Paenibacillus larvae subsp. larvae |
| 1 | Cellular orgnism | Paenibacillus larvae subsp. larvae BRL-230010 |
| 1 | Cellular orgnism | Paenibacillus polymyxa |
| 1 | Cellular orgnism | Paenibacillus polymyxa E681 |
| 1 | Cellular orgnism | Paenibacillus polymyxa SC2 |
| 1 | Cellular orgnism | Paenibacillus popilliae |
| 1 | Cellular orgnism | Paenibacillus sp. JDR-2 |
| 1 | Cellular orgnism | Paenibacillus sp. oral taxon 786 str. D14 |
| 1 | Cellular orgnism | Pantoea agglomerans |
| 1 | Cellular orgnism | Pantoea ananatis |
| 1 | Cellular orgnism | Pantoea ananatis LMG 20103 |
| 1 | Cellular orgnism | Pantoea citrea |
| 1 | Cellular orgnism | Pantoea sp. At-9b |
| 1 | Cellular orgnism | Pantoea sp. aB |
| 1 | Cellular orgnism | Pantoea vagans |
| 1 | Cellular orgnism | Pantoea vagans C9-1 |
| 1 | Cellular orgnism | Parabacteroides distasonis |
| 1 | Cellular orgnism | Parabacteroides distasonis ATCC 8503 |
| 1 | Cellular orgnism | Parabacteroides johnsonii DSM 18315 |
| 1 | Cellular orgnism | Parabacteroides merdae |
| 1 | Cellular orgnism | Parabacteroides merdae ATCC 43184 |
| 1 | Cellular orgnism | Parabacteroides sp. D13 |
| 1 | Cellular orgnism | Parachlamydia acanthamoebae |
| 1 | Cellular orgnism | Parachlamydia acanthamoebae str. Hall's coccus |
| 1 | Cellular orgnism | Paracoccus aminophilus |
| 1 | Cellular orgnism | Paracoccus denitrificans |
| 1 | Cellular orgnism | Paracoccus denitrificans PD1222 |
| 1 | Cellular orgnism | Paracoccus methylutens |
| 1 | Cellular orgnism | Paracoccus pantotrophus |
| 1 | Cellular orgnism | Parascardovia denticolens DSM 10105 |
| 1 | Cellular orgnism | Parascardovia denticolens F0305 |
| 1 | Cellular orgnism | Parvibaculum lavamentivorans DS-1 |
| 1 | Cellular orgnism | Parvimonas micra |
| 1 | Cellular orgnism | Parvimonas micra ATCC 33270 |
| 1 | Cellular orgnism | Parvularcula bermudensis HTCC2503 |
| 1 | Cellular orgnism | Pasteurella dagmatis |
| 1 | Cellular orgnism | Pasteurella dagmatis ATCC 43325 |
| 1 | Cellular orgnism | Pasteurella multocida |
| 1 | Cellular orgnism | Pasteurella multocida subsp. multocida |
| 1 | Cellular orgnism | Pasteurella multocida subsp. multocida str. Pm70 |
| 1 | Cellular orgnism | Pasteuria nishizawae str. North American |
| 1 | Cellular orgnism | Paulownia witches'-broom phytoplasma |
| 1 | Cellular orgnism | Peanut witches'-broom phytoplasma |
| 1 | Cellular orgnism | Pectobacterium atrosepticum |
| 1 | Cellular orgnism | Pectobacterium atrosepticum SCRI1043 |
| 1 | Cellular orgnism | Pectobacterium carotovorum |
| 1 | Cellular orgnism | Pectobacterium carotovorum subsp. brasiliensis |
| 1 | Cellular orgnism | Pectobacterium carotovorum subsp. brasiliensis PBR1692 |
| 1 | Cellular orgnism | Pectobacterium carotovorum subsp. carotovorum |
| 1 | Cellular orgnism | Pectobacterium carotovorum subsp. carotovorum PC1 |
| 1 | Cellular orgnism | Pectobacterium carotovorum subsp. carotovorum WPP14 |
| 1 | Cellular orgnism | Pectobacterium wasabiae |
| 1 | Cellular orgnism | Pectobacterium wasabiae WPP163 |
| 1 | Cellular orgnism | Pediococcus acidilactici |
| 1 | Cellular orgnism | Pediococcus acidilactici 7_4 |
| 1 | Cellular orgnism | Pediococcus acidilactici DSM 20284 |
| 1 | Cellular orgnism | Pediococcus damnosus |
| 1 | Cellular orgnism | Pediococcus pentosaceus |
| 1 | Cellular orgnism | Pediococcus pentosaceus ATCC 25745 |
| 1 | Cellular orgnism | Pedobacter heparinus |
| 1 | Cellular orgnism | Pedobacter heparinus DSM 2366 |
| 1 | Cellular orgnism | Pedobacter sp. BAL39 |
| 1 | Cellular orgnism | Pelagibaca bermudensis HTCC2601 |
| 1 | Cellular orgnism | Pelobacter carbinolicus |
| 1 | Cellular orgnism | Pelobacter carbinolicus DSM 2380 |
| 1 | Cellular orgnism | Pelobacter propionicus |
| 1 | Cellular orgnism | Pelobacter propionicus DSM 2379 |
| 1 | Cellular orgnism | Pelodictyon luteolum |
| 1 | Cellular orgnism | Pelodictyon phaeoclathratiforme |
| 1 | Cellular orgnism | Pelodictyon phaeoclathratiforme BU-1 |
| 1 | Cellular orgnism | Pelotomaculum thermopropionicum |
| 1 | Cellular orgnism | Pelotomaculum thermopropionicum SI |
| 1 | Cellular orgnism | Peptoniphilus duerdenii ATCC BAA-1640 |
| 1 | Cellular orgnism | Peptoniphilus harei |
| 1 | Cellular orgnism | Peptoniphilus lacrimalis |
| 1 | Cellular orgnism | Peptoniphilus lacrimalis 315-B |
| 1 | Cellular orgnism | Peptoniphilus sp. oral taxon 386 str. F0131 |
| 1 | Cellular orgnism | Peptoniphilus sp. oral taxon 836 str. F0141 |
| 1 | Cellular orgnism | Peptostreptococcus anaerobius |
| 1 | Cellular orgnism | Peptostreptococcus anaerobius 653-L |
| 1 | Cellular orgnism | Peptostreptococcus stomatis DSM 17678 |
| 1 | Cellular orgnism | Persephonella marina |
| 1 | Cellular orgnism | Persephonella marina EX-H1 |
| 1 | Cellular orgnism | Petrotoga mobilis SJ95 |
| 1 | Cellular orgnism | Phaeobacter gallaeciensis |
| 1 | Cellular orgnism | Phaeobacter gallaeciensis 2.10 |
| 1 | Cellular orgnism | Phaeobacter gallaeciensis BS107 |
| 1 | Cellular orgnism | Phenylobacterium zucineum |
| 1 | Cellular orgnism | Phenylobacterium zucineum HLK1 |
| 1 | Cellular orgnism | Photobacterium angustum |
| 1 | Cellular orgnism | Photobacterium angustum S14 |
| 1 | Cellular orgnism | Photobacterium damselae |
| 1 | Cellular orgnism | Photobacterium damselae subsp. damselae |
| 1 | Cellular orgnism | Photobacterium damselae subsp. damselae CIP 102761 |
| 1 | Cellular orgnism | Photobacterium profundum |
| 1 | Cellular orgnism | Photobacterium profundum 3TCK |
| 1 | Cellular orgnism | Photobacterium profundum SS9 |
| 1 | Cellular orgnism | Photobacterium sp. SKA34 |
| 1 | Cellular orgnism | Photorhabdus asymbiotica |
| 1 | Cellular orgnism | Photorhabdus asymbiotica subsp. asymbiotica |
| 1 | Cellular orgnism | Photorhabdus asymbiotica subsp. asymbiotica ATCC 43949 |
| 1 | Cellular orgnism | Photorhabdus luminescens |
| 1 | Cellular orgnism | Photorhabdus luminescens subsp. laumondii |
| 1 | Cellular orgnism | Photorhabdus luminescens subsp. laumondii TTO1 |
| 1 | Cellular orgnism | Phytoplasma sp. |
| 1 | Cellular orgnism | Pirellula staleyi |
| 1 | Cellular orgnism | Pirellula staleyi DSM 6068 |
| 1 | Cellular orgnism | Planctomyces brasiliensis DSM 5305 |
| 1 | Cellular orgnism | Planctomyces limnophilus |
| 1 | Cellular orgnism | Planctomyces limnophilus DSM 3776 |
| 1 | Cellular orgnism | Planctomyces maris |
| 1 | Cellular orgnism | Planctomyces maris DSM 8797 |
| 1 | Cellular orgnism | Planococcus sp. ZOYM |
| 1 | Cellular orgnism | Plesiocystis pacifica |
| 1 | Cellular orgnism | Plesiocystis pacifica SIR-1 |
| 1 | Cellular orgnism | Polaribacter irgensii 23-P |
| 1 | Cellular orgnism | Polaribacter sp. MED152 |
| 1 | Cellular orgnism | Polaromonas naphthalenivorans CJ2 |
| 1 | Cellular orgnism | Polaromonas sp. JS666 |
| 1 | Cellular orgnism | Polynucleobacter necessarius subsp. asymbioticus |
| 1 | Cellular orgnism | Polynucleobacter necessarius subsp. asymbioticus QLW-P1DMWA-1 |
| 1 | Cellular orgnism | Polynucleobacter necessarius subsp. necessarius |
| 1 | Cellular orgnism | Polynucleobacter necessarius subsp. necessarius STIR1 |
| 1 | Cellular orgnism | Porphyromonas asaccharolytica |
| 1 | Cellular orgnism | Porphyromonas endodontalis |
| 1 | Cellular orgnism | Porphyromonas endodontalis ATCC 35406 |
| 1 | Cellular orgnism | Porphyromonas gingivalis |
| 1 | Cellular orgnism | Porphyromonas gingivalis ATCC 33277 |
| 1 | Cellular orgnism | Porphyromonas gingivalis W83 |
| 1 | Cellular orgnism | Porphyromonas uenonis |
| 1 | Cellular orgnism | Porphyromonas uenonis 60-3 |
| 1 | Cellular orgnism | Prevotella amnii |
| 1 | Cellular orgnism | Prevotella amnii CRIS 21A-A |
| 1 | Cellular orgnism | Prevotella bergensis |
| 1 | Cellular orgnism | Prevotella bergensis DSM 17361 |
| 1 | Cellular orgnism | Prevotella bivia |
| 1 | Cellular orgnism | Prevotella bivia JCVIHMP010 |
| 1 | Cellular orgnism | Prevotella bryantii |
| 1 | Cellular orgnism | Prevotella bryantii B14 |
| 1 | Cellular orgnism | Prevotella buccae |
| 1 | Cellular orgnism | Prevotella buccae ATCC 33574 |
| 1 | Cellular orgnism | Prevotella buccae D17 |
| 1 | Cellular orgnism | Prevotella buccalis |
| 1 | Cellular orgnism | Prevotella buccalis ATCC 35310 |
| 1 | Cellular orgnism | Prevotella copri DSM 18205 |
| 1 | Cellular orgnism | Prevotella disiens |
| 1 | Cellular orgnism | Prevotella disiens FB035-09AN |
| 1 | Cellular orgnism | Prevotella marshii |
| 1 | Cellular orgnism | Prevotella marshii DSM 16973 |
| 1 | Cellular orgnism | Prevotella melaninogenica |
| 1 | Cellular orgnism | Prevotella melaninogenica ATCC 25845 |
| 1 | Cellular orgnism | Prevotella melaninogenica D18 |
| 1 | Cellular orgnism | Prevotella oralis |
| 1 | Cellular orgnism | Prevotella oralis ATCC 33269 |
| 1 | Cellular orgnism | Prevotella oris |
| 1 | Cellular orgnism | Prevotella oris C735 |
| 1 | Cellular orgnism | Prevotella oris F0302 |
| 1 | Cellular orgnism | Prevotella ruminicola |
| 1 | Cellular orgnism | Prevotella ruminicola 23 |
| 1 | Cellular orgnism | Prevotella salivae DSM 15606 |
| 1 | Cellular orgnism | Prevotella sp. oral taxon 299 str. F0039 |
| 1 | Cellular orgnism | Prevotella sp. oral taxon 317 str. F0108 |
| 1 | Cellular orgnism | Prevotella sp. oral taxon 472 str. F0295 |
| 1 | Cellular orgnism | Prevotella tannerae ATCC 51259 |
| 1 | Cellular orgnism | Prevotella timonensis |
| 1 | Cellular orgnism | Prevotella timonensis CRIS 5C-B1 |
| 1 | Cellular orgnism | Prevotella veroralis |
| 1 | Cellular orgnism | Prevotella veroralis F0319 |
| 1 | Cellular orgnism | Prochlorococcus marinus |
| 1 | Cellular orgnism | Prochlorococcus marinus str. AS9601 |
| 1 | Cellular orgnism | Prochlorococcus marinus str. MIT 9202 |
| 1 | Cellular orgnism | Prochlorococcus marinus str. MIT 9211 |
| 1 | Cellular orgnism | Prochlorococcus marinus str. MIT 9215 |
| 1 | Cellular orgnism | Prochlorococcus marinus str. MIT 9301 |
| 1 | Cellular orgnism | Prochlorococcus marinus str. MIT 9303 |
| 1 | Cellular orgnism | Prochlorococcus marinus str. MIT 9312 |
| 1 | Cellular orgnism | Prochlorococcus marinus str. MIT 9313 |
| 1 | Cellular orgnism | Prochlorococcus marinus str. MIT 9515 |
| 1 | Cellular orgnism | Prochlorococcus marinus str. NATL1A |
| 1 | Cellular orgnism | Prochlorococcus marinus str. NATL2A |
| 1 | Cellular orgnism | Prochlorococcus marinus subsp. marinus str. CCMP1375 |
| 1 | Cellular orgnism | Prochlorococcus marinus subsp. pastoris |
| 1 | Cellular orgnism | Prochlorococcus marinus subsp. pastoris str. CCMP1986 |
| 1 | Cellular orgnism | Propionibacterium acidipropionici |
| 1 | Cellular orgnism | Propionibacterium acnes |
| 1 | Cellular orgnism | Propionibacterium acnes J139 |
| 1 | Cellular orgnism | Propionibacterium acnes J165 |
| 1 | Cellular orgnism | Propionibacterium acnes KPA171202 |
| 1 | Cellular orgnism | Propionibacterium acnes SK137 |
| 1 | Cellular orgnism | Propionibacterium acnes SK187 |
| 1 | Cellular orgnism | Propionibacterium freudenreichii |
| 1 | Cellular orgnism | Propionibacterium freudenreichii subsp. shermanii |
| 1 | Cellular orgnism | Propionibacterium freudenreichii subsp. shermanii CIRM-BIA1 |
| 1 | Cellular orgnism | Propionibacterium granulosum |
| 1 | Cellular orgnism | Propionibacterium jensenii |
| 1 | Cellular orgnism | Prosthecochloris aestuarii |
| 1 | Cellular orgnism | Prosthecochloris aestuarii DSM 271 |
| 1 | Cellular orgnism | Proteus mirabilis |
| 1 | Cellular orgnism | Proteus mirabilis ATCC 29906 |
| 1 | Cellular orgnism | Proteus mirabilis HI4320 |
| 1 | Cellular orgnism | Proteus penneri |
| 1 | Cellular orgnism | Proteus penneri ATCC 35198 |
| 1 | Cellular orgnism | Proteus vulgaris |
| 1 | Cellular orgnism | Providencia alcalifaciens |
| 1 | Cellular orgnism | Providencia alcalifaciens DSM 30120 |
| 1 | Cellular orgnism | Providencia rettgeri |
| 1 | Cellular orgnism | Providencia rettgeri DSM 1131 |
| 1 | Cellular orgnism | Providencia rustigianii |
| 1 | Cellular orgnism | Providencia rustigianii DSM 4541 |
| 1 | Cellular orgnism | Providencia stuartii |
| 1 | Cellular orgnism | Providencia stuartii ATCC 25827 |
| 1 | Cellular orgnism | Pseudoalteromonas atlantica |
| 1 | Cellular orgnism | Pseudoalteromonas atlantica T6c |
| 1 | Cellular orgnism | Pseudoalteromonas haloplanktis |
| 1 | Cellular orgnism | Pseudoalteromonas haloplanktis TAC125 |
| 1 | Cellular orgnism | Pseudoalteromonas sp. 643A |
| 1 | Cellular orgnism | Pseudoalteromonas sp. BSi20327 |
| 1 | Cellular orgnism | Pseudoalteromonas sp. PS1M3 |
| 1 | Cellular orgnism | Pseudoalteromonas sp. SM9913 |
| 1 | Cellular orgnism | Pseudoalteromonas tunicata |
| 1 | Cellular orgnism | Pseudoalteromonas tunicata D2 |
| 1 | Cellular orgnism | Pseudomonas |
| 1 | Cellular orgnism | Pseudomonas aeruginosa |
| 1 | Cellular orgnism | Pseudomonas aeruginosa 2192 |
| 1 | Cellular orgnism | Pseudomonas aeruginosa C3719 |
| 1 | Cellular orgnism | Pseudomonas aeruginosa LESB58 |
| 1 | Cellular orgnism | Pseudomonas aeruginosa PA7 |
| 1 | Cellular orgnism | Pseudomonas aeruginosa PACS2 |
| 1 | Cellular orgnism | Pseudomonas aeruginosa PAO1 |
| 1 | Cellular orgnism | Pseudomonas aeruginosa PAb1 |
| 1 | Cellular orgnism | Pseudomonas aeruginosa UCBPP-PA14 |
| 1 | Cellular orgnism | Pseudomonas alcaligenes |
| 1 | Cellular orgnism | Pseudomonas amygdali |
| 1 | Cellular orgnism | Pseudomonas coronafaciens |
| 1 | Cellular orgnism | Pseudomonas entomophila |
| 1 | Cellular orgnism | Pseudomonas entomophila L48 |
| 1 | Cellular orgnism | Pseudomonas fluorescens |
| 1 | Cellular orgnism | Pseudomonas fluorescens Pf-5 |
| 1 | Cellular orgnism | Pseudomonas fluorescens Pf0-1 |
| 1 | Cellular orgnism | Pseudomonas fluorescens SBW25 |
| 1 | Cellular orgnism | Pseudomonas fulva |
| 1 | Cellular orgnism | Pseudomonas mendocina |
| 1 | Cellular orgnism | Pseudomonas mendocina ymp |
| 1 | Cellular orgnism | Pseudomonas putida |
| 1 | Cellular orgnism | Pseudomonas putida F1 |
| 1 | Cellular orgnism | Pseudomonas putida GB-1 |
| 1 | Cellular orgnism | Pseudomonas putida KT2440 |
| 1 | Cellular orgnism | Pseudomonas putida W619 |
| 1 | Cellular orgnism | Pseudomonas resinovorans |
| 1 | Cellular orgnism | Pseudomonas savastanoi |
| 1 | Cellular orgnism | Pseudomonas savastanoi pv. savastanoi |
| 1 | Cellular orgnism | Pseudomonas savastanoi pv. savastanoi NCPPB 3335 |
| 1 | Cellular orgnism | Pseudomonas sp. ADP |
| 1 | Cellular orgnism | Pseudomonas sp. CA10 |
| 1 | Cellular orgnism | Pseudomonas sp. CG21 |
| 1 | Cellular orgnism | Pseudomonas sp. CT14 |
| 1 | Cellular orgnism | Pseudomonas sp. ND6 |
| 1 | Cellular orgnism | Pseudomonas sp. S-47 |
| 1 | Cellular orgnism | Pseudomonas sp. SLT2001 |
| 1 | Cellular orgnism | Pseudomonas sp. UK4 |
| 1 | Cellular orgnism | Pseudomonas stutzeri |
| 1 | Cellular orgnism | Pseudomonas stutzeri A1501 |
| 1 | Cellular orgnism | Pseudomonas syringae |
| 1 | Cellular orgnism | Pseudomonas syringae pv. aesculi |
| 1 | Cellular orgnism | Pseudomonas syringae pv. aesculi str. 2250 |
| 1 | Cellular orgnism | Pseudomonas syringae pv. aesculi str. NCPPB3681 |
| 1 | Cellular orgnism | Pseudomonas syringae pv. maculicola |
| 1 | Cellular orgnism | Pseudomonas syringae pv. maculicola str. M6 |
| 1 | Cellular orgnism | Pseudomonas syringae pv. oryzae |
| 1 | Cellular orgnism | Pseudomonas syringae pv. oryzae str. 1_6 |
| 1 | Cellular orgnism | Pseudomonas syringae pv. phaseolicola |
| 1 | Cellular orgnism | Pseudomonas syringae pv. phaseolicola 1448A |
| 1 | Cellular orgnism | Pseudomonas syringae pv. syringae |
| 1 | Cellular orgnism | Pseudomonas syringae pv. syringae 642 |
| 1 | Cellular orgnism | Pseudomonas syringae pv. syringae B728a |
| 1 | Cellular orgnism | Pseudomonas syringae pv. syringae FF5 |
| 1 | Cellular orgnism | Pseudomonas syringae pv. tabaci |
| 1 | Cellular orgnism | Pseudomonas syringae pv. tabaci ATCC 11528 |
| 1 | Cellular orgnism | Pseudomonas syringae pv. tomato |
| 1 | Cellular orgnism | Pseudomonas syringae pv. tomato K40 |
| 1 | Cellular orgnism | Pseudomonas syringae pv. tomato Max13 |
| 1 | Cellular orgnism | Pseudomonas syringae pv. tomato NCPPB 1108 |
| 1 | Cellular orgnism | Pseudomonas syringae pv. tomato T1 |
| 1 | Cellular orgnism | Pseudomonas syringae pv. tomato str. DC3000 |
| 1 | Cellular orgnism | Pseudonocardia autotrophica |
| 1 | Cellular orgnism | Pseudovibrio sp. JE062 |
| 1 | Cellular orgnism | Psychrobacter arcticus |
| 1 | Cellular orgnism | Psychrobacter arcticus 273-4 |
| 1 | Cellular orgnism | Psychrobacter cryohalolentis |
| 1 | Cellular orgnism | Psychrobacter cryohalolentis K5 |
| 1 | Cellular orgnism | Psychrobacter sp. PRwf-1 |
| 1 | Cellular orgnism | Psychroflexus torquis ATCC 700755 |
| 1 | Cellular orgnism | Psychromonas ingrahamii 37 |
| 1 | Cellular orgnism | Psychromonas sp. CNPT3 |
| 1 | Cellular orgnism | Pyramidobacter piscolens W5455 |
| 1 | Cellular orgnism | Rahnella sp. 'WMR15' |
| 1 | Cellular orgnism | Ralstonia eutropha H16 |
| 1 | Cellular orgnism | Ralstonia eutropha JMP134 |
| 1 | Cellular orgnism | Ralstonia pickettii |
| 1 | Cellular orgnism | Ralstonia pickettii 12D |
| 1 | Cellular orgnism | Ralstonia pickettii 12J |
| 1 | Cellular orgnism | Ralstonia solanacearum |
| 1 | Cellular orgnism | Ralstonia solanacearum CFBP2957 |
| 1 | Cellular orgnism | Ralstonia solanacearum GMI1000 |
| 1 | Cellular orgnism | Ralstonia solanacearum MolK2 |
| 1 | Cellular orgnism | Ralstonia solanacearum PSI07 |
| 1 | Cellular orgnism | Ralstonia solanacearum UW551 |
| 1 | Cellular orgnism | Ralstonia sp. 5_7_47FAA |
| 1 | Cellular orgnism | Raphidiopsis brookii D9 |
| 1 | Cellular orgnism | Renibacterium salmoninarum |
| 1 | Cellular orgnism | Renibacterium salmoninarum ATCC 33209 |
| 1 | Cellular orgnism | Rhizobium etli |
| 1 | Cellular orgnism | Rhizobium etli 8C-3 |
| 1 | Cellular orgnism | Rhizobium etli Brasil 5 |
| 1 | Cellular orgnism | Rhizobium etli CFN 42 |
| 1 | Cellular orgnism | Rhizobium etli CIAT 652 |
| 1 | Cellular orgnism | Rhizobium etli CIAT 894 |
| 1 | Cellular orgnism | Rhizobium etli GR56 |
| 1 | Cellular orgnism | Rhizobium etli IE4771 |
| 1 | Cellular orgnism | Rhizobium etli Kim 5 |
| 1 | Cellular orgnism | Rhizobium leguminosarum |
| 1 | Cellular orgnism | Rhizobium leguminosarum bv. trifolii |
| 1 | Cellular orgnism | Rhizobium leguminosarum bv. trifolii WSM1325 |
| 1 | Cellular orgnism | Rhizobium leguminosarum bv. trifolii WSM2304 |
| 1 | Cellular orgnism | Rhizobium leguminosarum bv. viciae |
| 1 | Cellular orgnism | Rhizobium leguminosarum bv. viciae 3841 |
| 1 | Cellular orgnism | Rhodobacter blasticus |
| 1 | Cellular orgnism | Rhodobacter capsulatus |
| 1 | Cellular orgnism | Rhodobacter capsulatus SB 1003 |
| 1 | Cellular orgnism | Rhodobacter sp. SW2 |
| 1 | Cellular orgnism | Rhodobacter sphaeroides |
| 1 | Cellular orgnism | Rhodobacter sphaeroides 2.4.1 |
| 1 | Cellular orgnism | Rhodobacter sphaeroides ATCC 17025 |
| 1 | Cellular orgnism | Rhodobacter sphaeroides ATCC 17029 |
| 1 | Cellular orgnism | Rhodobacter sphaeroides KD131 |
| 1 | Cellular orgnism | Rhodobacteraceae bacterium KLH11 |
| 1 | Cellular orgnism | Rhodobacterales bacterium Y4I |
| 1 | Cellular orgnism | Rhodococcus aetherivorans |
| 1 | Cellular orgnism | Rhodococcus equi |
| 1 | Cellular orgnism | Rhodococcus equi ATCC 33707 |
| 1 | Cellular orgnism | Rhodococcus erythropolis |
| 1 | Cellular orgnism | Rhodococcus erythropolis PR4 |
| 1 | Cellular orgnism | Rhodococcus erythropolis SK121 |
| 1 | Cellular orgnism | Rhodococcus jostii |
| 1 | Cellular orgnism | Rhodococcus jostii RHA1 |
| 1 | Cellular orgnism | Rhodococcus opacus |
| 1 | Cellular orgnism | Rhodococcus opacus B4 |
| 1 | Cellular orgnism | Rhodococcus rhodochrous |
| 1 | Cellular orgnism | Rhodococcus sp. B264-1 |
| 1 | Cellular orgnism | Rhodococcus sp. NS1 |
| 1 | Cellular orgnism | Rhodoferax ferrireducens T118 |
| 1 | Cellular orgnism | Rhodomicrobium vannielii |
| 1 | Cellular orgnism | Rhodomicrobium vannielii ATCC 17100 |
| 1 | Cellular orgnism | Rhodopirellula baltica |
| 1 | Cellular orgnism | Rhodopirellula baltica SH 1 |
| 1 | Cellular orgnism | Rhodopseudomonas palustris |
| 1 | Cellular orgnism | Rhodopseudomonas palustris BisA53 |
| 1 | Cellular orgnism | Rhodopseudomonas palustris BisB18 |
| 1 | Cellular orgnism | Rhodopseudomonas palustris BisB5 |
| 1 | Cellular orgnism | Rhodopseudomonas palustris CGA009 |
| 1 | Cellular orgnism | Rhodopseudomonas palustris DX-1 |
| 1 | Cellular orgnism | Rhodopseudomonas palustris HaA2 |
| 1 | Cellular orgnism | Rhodopseudomonas palustris TIE-1 |
| 1 | Cellular orgnism | Rhodospirillum centenum |
| 1 | Cellular orgnism | Rhodospirillum centenum SW |
| 1 | Cellular orgnism | Rhodospirillum rubrum |
| 1 | Cellular orgnism | Rhodospirillum rubrum ATCC 11170 |
| 1 | Cellular orgnism | Rhodothermus marinus |
| 1 | Cellular orgnism | Rhodothermus marinus DSM 4252 |
| 1 | Cellular orgnism | Rickettsia africae |
| 1 | Cellular orgnism | Rickettsia africae ESF-5 |
| 1 | Cellular orgnism | Rickettsia akari str. Hartford |
| 1 | Cellular orgnism | Rickettsia bellii |
| 1 | Cellular orgnism | Rickettsia bellii OSU 85-389 |
| 1 | Cellular orgnism | Rickettsia bellii RML369-C |
| 1 | Cellular orgnism | Rickettsia canadensis |
| 1 | Cellular orgnism | Rickettsia canadensis str. McKiel |
| 1 | Cellular orgnism | Rickettsia conorii |
| 1 | Cellular orgnism | Rickettsia conorii str. Malish 7 |
| 1 | Cellular orgnism | Rickettsia endosymbiont of Ixodes scapularis |
| 1 | Cellular orgnism | Rickettsia felis |
| 1 | Cellular orgnism | Rickettsia felis URRWXCal2 |
| 1 | Cellular orgnism | Rickettsia massiliae |
| 1 | Cellular orgnism | Rickettsia massiliae MTU5 |
| 1 | Cellular orgnism | Rickettsia monacensis |
| 1 | Cellular orgnism | Rickettsia peacockii |
| 1 | Cellular orgnism | Rickettsia peacockii str. Rustic |
| 1 | Cellular orgnism | Rickettsia prowazekii |
| 1 | Cellular orgnism | Rickettsia prowazekii str. Madrid E |
| 1 | Cellular orgnism | Rickettsia rickettsii |
| 1 | Cellular orgnism | Rickettsia rickettsii str. 'Sheila Smith' |
| 1 | Cellular orgnism | Rickettsia rickettsii str. Iowa |
| 1 | Cellular orgnism | Rickettsia sibirica |
| 1 | Cellular orgnism | Rickettsia sibirica 246 |
| 1 | Cellular orgnism | Rickettsia typhi |
| 1 | Cellular orgnism | Rickettsia typhi str. Wilmington |
| 1 | Cellular orgnism | Rickettsiella grylli |
| 1 | Cellular orgnism | Riemerella anatipestifer |
| 1 | Cellular orgnism | Robiginitalea biformata HTCC2501 |
| 1 | Cellular orgnism | Roseburia intestinalis |
| 1 | Cellular orgnism | Roseburia intestinalis L1-82 |
| 1 | Cellular orgnism | Roseburia inulinivorans |
| 1 | Cellular orgnism | Roseburia inulinivorans DSM 16841 |
| 1 | Cellular orgnism | Roseibium sp. TrichSKD4 |
| 1 | Cellular orgnism | Roseiflexus castenholzii |
| 1 | Cellular orgnism | Roseiflexus castenholzii DSM 13941 |
| 1 | Cellular orgnism | Roseiflexus sp. RS-1 |
| 1 | Cellular orgnism | Roseobacter denitrificans |
| 1 | Cellular orgnism | Roseobacter denitrificans OCh 114 |
| 1 | Cellular orgnism | Roseobacter litoralis |
| 1 | Cellular orgnism | Roseobacter litoralis Och 149 |
| 1 | Cellular orgnism | Roseobacter sp. AzwK-3b |
| 1 | Cellular orgnism | Roseobacter sp. CCS2 |
| 1 | Cellular orgnism | Roseobacter sp. GAI101 |
| 1 | Cellular orgnism | Roseobacter sp. MED193 |
| 1 | Cellular orgnism | Roseobacter sp. SK209-2-6 |
| 1 | Cellular orgnism | Roseomonas cervicalis ATCC 49957 |
| 1 | Cellular orgnism | Roseovarius nubinhibens |
| 1 | Cellular orgnism | Roseovarius nubinhibens ISM |
| 1 | Cellular orgnism | Roseovarius sp. 217 |
| 1 | Cellular orgnism | Roseovarius sp. TM1035 |
| 1 | Cellular orgnism | Rothia dentocariosa |
| 1 | Cellular orgnism | Rothia dentocariosa ATCC 17931 |
| 1 | Cellular orgnism | Rothia dentocariosa M567 |
| 1 | Cellular orgnism | Rothia mucilaginosa ATCC 25296 |
| 1 | Cellular orgnism | Rothia mucilaginosa DY-18 |
| 1 | Cellular orgnism | Rubrobacter xylanophilus |
| 1 | Cellular orgnism | Rubrobacter xylanophilus DSM 9941 |
| 1 | Cellular orgnism | Ruegeria pomeroyi |
| 1 | Cellular orgnism | Ruegeria pomeroyi DSS-3 |
| 1 | Cellular orgnism | Ruegeria sp. PR1b |
| 1 | Cellular orgnism | Ruegeria sp. R11 |
| 1 | Cellular orgnism | Ruegeria sp. TM1040 |
| 1 | Cellular orgnism | Ruminococcaceae bacterium D16 |
| 1 | Cellular orgnism | Ruminococcus albus |
| 1 | Cellular orgnism | Ruminococcus albus 7 |
| 1 | Cellular orgnism | Ruminococcus albus 8 |
| 1 | Cellular orgnism | Ruminococcus flavefaciens |
| 1 | Cellular orgnism | Ruminococcus flavefaciens FD-1 |
| 1 | Cellular orgnism | Ruminococcus gnavus |
| 1 | Cellular orgnism | Ruminococcus gnavus ATCC 29149 |
| 1 | Cellular orgnism | Ruminococcus lactaris ATCC 29176 |
| 1 | Cellular orgnism | Ruminococcus obeum ATCC 29174 |
| 1 | Cellular orgnism | Ruminococcus torques ATCC 27756 |
| 1 | Cellular orgnism | Saccharomonospora viridis |
| 1 | Cellular orgnism | Saccharomonospora viridis DSM 43017 |
| 1 | Cellular orgnism | Saccharophagus degradans 2-40 |
| 1 | Cellular orgnism | Saccharopolyspora erythraea |
| 1 | Cellular orgnism | Saccharopolyspora erythraea NRRL 2338 |
| 1 | Cellular orgnism | Sagittula stellata |
| 1 | Cellular orgnism | Sagittula stellata E-37 |
| 1 | Cellular orgnism | Salinibacter ruber |
| 1 | Cellular orgnism | Salinibacter ruber DSM 13855 |
| 1 | Cellular orgnism | Salinibacter ruber M8 |
| 1 | Cellular orgnism | Salinispora arenicola |
| 1 | Cellular orgnism | Salinispora arenicola CNS-205 |
| 1 | Cellular orgnism | Salinispora tropica |
| 1 | Cellular orgnism | Salinispora tropica CNB-440 |
| 1 | Cellular orgnism | Salmonella enterica |
| 1 | Cellular orgnism | Salmonella enterica subsp. arizonae |
| 1 | Cellular orgnism | Salmonella enterica subsp. arizonae serovar 62:z4,z23:-- |
| 1 | Cellular orgnism | Salmonella enterica subsp. arizonae serovar 62:z4,z23:-- str. RSK2980 |
| 1 | Cellular orgnism | Salmonella enterica subsp. enterica |
| 1 | Cellular orgnism | Salmonella enterica subsp. enterica serovar Agona |
| 1 | Cellular orgnism | Salmonella enterica subsp. enterica serovar Agona str. SL483 |
| 1 | Cellular orgnism | Salmonella enterica subsp. enterica serovar Berta |
| 1 | Cellular orgnism | Salmonella enterica subsp. enterica serovar Bovismorbificans |
| 1 | Cellular orgnism | Salmonella enterica subsp. enterica serovar Brandenburg |
| 1 | Cellular orgnism | Salmonella enterica subsp. enterica serovar Choleraesuis |
| 1 | Cellular orgnism | Salmonella enterica subsp. enterica serovar Choleraesuis str. SC-B67 |
| 1 | Cellular orgnism | Salmonella enterica subsp. enterica serovar Dublin |
| 1 | Cellular orgnism | Salmonella enterica subsp. enterica serovar Dublin str. CT_02021853 |
| 1 | Cellular orgnism | Salmonella enterica subsp. enterica serovar Enteritidis |
| 1 | Cellular orgnism | Salmonella enterica subsp. enterica serovar Enteritidis str. P125109 |
| 1 | Cellular orgnism | Salmonella enterica subsp. enterica serovar Gallinarum |
| 1 | Cellular orgnism | Salmonella enterica subsp. enterica serovar Gallinarum str. 287/91 |
| 1 | Cellular orgnism | Salmonella enterica subsp. enterica serovar Hadar |
| 1 | Cellular orgnism | Salmonella enterica subsp. enterica serovar Hadar str. RI_05P066 |
| 1 | Cellular orgnism | Salmonella enterica subsp. enterica serovar Heidelberg |
| 1 | Cellular orgnism | Salmonella enterica subsp. enterica serovar Heidelberg str. SL476 |
| 1 | Cellular orgnism | Salmonella enterica subsp. enterica serovar Heidelberg str. SL486 |
| 1 | Cellular orgnism | Salmonella enterica subsp. enterica serovar Javiana |
| 1 | Cellular orgnism | Salmonella enterica subsp. enterica serovar Javiana str. GA_MM04042433 |
| 1 | Cellular orgnism | Salmonella enterica subsp. enterica serovar Kentucky |
| 1 | Cellular orgnism | Salmonella enterica subsp. enterica serovar Kentucky str. CDC 191 |
| 1 | Cellular orgnism | Salmonella enterica subsp. enterica serovar Kentucky str. CVM29188 |
| 1 | Cellular orgnism | Salmonella enterica subsp. enterica serovar Newport |
| 1 | Cellular orgnism | Salmonella enterica subsp. enterica serovar Newport str. SL254 |
| 1 | Cellular orgnism | Salmonella enterica subsp. enterica serovar Newport str. SL317 |
| 1 | Cellular orgnism | Salmonella enterica subsp. enterica serovar Paratyphi A |
| 1 | Cellular orgnism | Salmonella enterica subsp. enterica serovar Paratyphi A str. AKU_12601 |
| 1 | Cellular orgnism | Salmonella enterica subsp. enterica serovar Paratyphi A str. ATCC 9150 |
| 1 | Cellular orgnism | Salmonella enterica subsp. enterica serovar Paratyphi B |
| 1 | Cellular orgnism | Salmonella enterica subsp. enterica serovar Paratyphi B str. SPB7 |
| 1 | Cellular orgnism | Salmonella enterica subsp. enterica serovar Paratyphi C |
| 1 | Cellular orgnism | Salmonella enterica subsp. enterica serovar Paratyphi C strain RKS4594 |
| 1 | Cellular orgnism | Salmonella enterica subsp. enterica serovar Saintpaul |
| 1 | Cellular orgnism | Salmonella enterica subsp. enterica serovar Saintpaul str. SARA23 |
| 1 | Cellular orgnism | Salmonella enterica subsp. enterica serovar Saintpaul str. SARA29 |
| 1 | Cellular orgnism | Salmonella enterica subsp. enterica serovar Schwarzengrund |
| 1 | Cellular orgnism | Salmonella enterica subsp. enterica serovar Schwarzengrund str. CVM19633 |
| 1 | Cellular orgnism | Salmonella enterica subsp. enterica serovar Schwarzengrund str. SL480 |
| 1 | Cellular orgnism | Salmonella enterica subsp. enterica serovar Tennessee |
| 1 | Cellular orgnism | Salmonella enterica subsp. enterica serovar Tennessee str. CDC07-0191 |
| 1 | Cellular orgnism | Salmonella enterica subsp. enterica serovar Typhi |
| 1 | Cellular orgnism | Salmonella enterica subsp. enterica serovar Typhi str. 404ty |
| 1 | Cellular orgnism | Salmonella enterica subsp. enterica serovar Typhi str. AG3 |
| 1 | Cellular orgnism | Salmonella enterica subsp. enterica serovar Typhi str. CT18 |
| 1 | Cellular orgnism | Salmonella enterica subsp. enterica serovar Typhi str. E00-7866 |
| 1 | Cellular orgnism | Salmonella enterica subsp. enterica serovar Typhi str. E01-6750 |
| 1 | Cellular orgnism | Salmonella enterica subsp. enterica serovar Typhi str. E02-1180 |
| 1 | Cellular orgnism | Salmonella enterica subsp. enterica serovar Typhi str. E98-0664 |
| 1 | Cellular orgnism | Salmonella enterica subsp. enterica serovar Typhi str. E98-2068 |
| 1 | Cellular orgnism | Salmonella enterica subsp. enterica serovar Typhi str. E98-3139 |
| 1 | Cellular orgnism | Salmonella enterica subsp. enterica serovar Typhi str. J185 |
| 1 | Cellular orgnism | Salmonella enterica subsp. enterica serovar Typhi str. M223 |
| 1 | Cellular orgnism | Salmonella enterica subsp. enterica serovar Typhi str. Ty2 |
| 1 | Cellular orgnism | Salmonella enterica subsp. enterica serovar Typhimurium |
| 1 | Cellular orgnism | Salmonella enterica subsp. enterica serovar Typhimurium str. LT2 |
| 1 | Cellular orgnism | Salmonella enterica subsp. enterica serovar Virchow |
| 1 | Cellular orgnism | Salmonella enterica subsp. enterica serovar Virchow str. SL491 |
| 1 | Cellular orgnism | Salmonella enterica subsp. enterica serovar Weltevreden |
| 1 | Cellular orgnism | Salmonella enterica subsp. enterica serovar Weltevreden str. HI_N05-537 |
| 1 | Cellular orgnism | Salmonella enterica subsp. enterica serovar Westhampton |
| 1 | Cellular orgnism | Sanguibacter keddieii DSM 10542 |
| 1 | Cellular orgnism | Scardovia inopinata |
| 1 | Cellular orgnism | Scardovia inopinata F0304 |
| 1 | Cellular orgnism | Sebaldella termitidis ATCC 33386 |
| 1 | Cellular orgnism | Segniliparus rotundus DSM 44985 |
| 1 | Cellular orgnism | Segniliparus rugosus |
| 1 | Cellular orgnism | Selenomonas flueggei ATCC 43531 |
| 1 | Cellular orgnism | Selenomonas noxia ATCC 43541 |
| 1 | Cellular orgnism | Selenomonas ruminantium |
| 1 | Cellular orgnism | Selenomonas sp. oral taxon 149 str. 67H29BP |
| 1 | Cellular orgnism | Selenomonas sputigena ATCC 35185 |
| 1 | Cellular orgnism | Serratia entomophila |
| 1 | Cellular orgnism | Serratia marcescens |
| 1 | Cellular orgnism | Serratia odorifera |
| 1 | Cellular orgnism | Serratia odorifera 4Rx13 |
| 1 | Cellular orgnism | Serratia odorifera DSM 4582 |
| 1 | Cellular orgnism | Serratia proteamaculans |
| 1 | Cellular orgnism | Serratia proteamaculans 568 |
| 1 | Cellular orgnism | Shewanella amazonensis SB2B |
| 1 | Cellular orgnism | Shewanella baltica |
| 1 | Cellular orgnism | Shewanella baltica BA175 |
| 1 | Cellular orgnism | Shewanella baltica OS155 |
| 1 | Cellular orgnism | Shewanella baltica OS183 |
| 1 | Cellular orgnism | Shewanella baltica OS185 |
| 1 | Cellular orgnism | Shewanella baltica OS195 |
| 1 | Cellular orgnism | Shewanella baltica OS223 |
| 1 | Cellular orgnism | Shewanella benthica |
| 1 | Cellular orgnism | Shewanella benthica KT99 |
| 1 | Cellular orgnism | Shewanella denitrificans OS217 |
| 1 | Cellular orgnism | Shewanella frigidimarina |
| 1 | Cellular orgnism | Shewanella frigidimarina NCIMB 400 |
| 1 | Cellular orgnism | Shewanella halifaxensis HAW-EB4 |
| 1 | Cellular orgnism | Shewanella loihica PV-4 |
| 1 | Cellular orgnism | Shewanella oneidensis |
| 1 | Cellular orgnism | Shewanella oneidensis MR-1 |
| 1 | Cellular orgnism | Shewanella pealeana |
| 1 | Cellular orgnism | Shewanella pealeana ATCC 700345 |
| 1 | Cellular orgnism | Shewanella piezotolerans WP3 |
| 1 | Cellular orgnism | Shewanella putrefaciens |
| 1 | Cellular orgnism | Shewanella putrefaciens CN-32 |
| 1 | Cellular orgnism | Shewanella sediminis |
| 1 | Cellular orgnism | Shewanella sediminis HAW-EB3 |
| 1 | Cellular orgnism | Shewanella sp. 33B |
| 1 | Cellular orgnism | Shewanella sp. ANA-3 |
| 1 | Cellular orgnism | Shewanella sp. MR-4 |
| 1 | Cellular orgnism | Shewanella sp. MR-7 |
| 1 | Cellular orgnism | Shewanella sp. W3-18-1 |
| 1 | Cellular orgnism | Shewanella violacea |
| 1 | Cellular orgnism | Shewanella violacea DSS12 |
| 1 | Cellular orgnism | Shewanella woodyi |
| 1 | Cellular orgnism | Shewanella woodyi ATCC 51908 |
| 1 | Cellular orgnism | Shigella boydii |
| 1 | Cellular orgnism | Shigella boydii CDC 3083-94 |
| 1 | Cellular orgnism | Shigella boydii Sb227 |
| 1 | Cellular orgnism | Shigella dysenteriae |
| 1 | Cellular orgnism | Shigella dysenteriae 1012 |
| 1 | Cellular orgnism | Shigella dysenteriae 1617 |
| 1 | Cellular orgnism | Shigella dysenteriae Sd197 |
| 1 | Cellular orgnism | Shigella flexneri |
| 1 | Cellular orgnism | Shigella flexneri 2a |
| 1 | Cellular orgnism | Shigella flexneri 2a str. 2457T |
| 1 | Cellular orgnism | Shigella flexneri 2a str. 301 |
| 1 | Cellular orgnism | Shigella flexneri 5 |
| 1 | Cellular orgnism | Shigella flexneri 5 str. 8401 |
| 1 | Cellular orgnism | Shigella flexneri 5a |
| 1 | Cellular orgnism | Shigella sonnei |
| 1 | Cellular orgnism | Shigella sonnei Ss046 |
| 1 | Cellular orgnism | Shigella sp. D9 |
| 1 | Cellular orgnism | Shuttleworthia satelles DSM 14600 |
| 1 | Cellular orgnism | Sideroxydans lithotrophicus ES-1 |
| 1 | Cellular orgnism | Silicibacter lacuscaerulensis ITI-1157 |
| 1 | Cellular orgnism | Silicibacter sp. TrichCH4B |
| 1 | Cellular orgnism | Simonsiella muelleri ATCC 29453 |
| 1 | Cellular orgnism | Sinorhizobium fredii |
| 1 | Cellular orgnism | Sinorhizobium medicae |
| 1 | Cellular orgnism | Sinorhizobium medicae WSM419 |
| 1 | Cellular orgnism | Sinorhizobium meliloti |
| 1 | Cellular orgnism | Sinorhizobium meliloti 1021 |
| 1 | Cellular orgnism | Sinorhizobium meliloti AK83 |
| 1 | Cellular orgnism | Sinorhizobium meliloti BL225C |
| 1 | Cellular orgnism | Sinorhizobium meliloti SM11 |
| 1 | Cellular orgnism | Slackia exigua ATCC 700122 |
| 1 | Cellular orgnism | Slackia heliotrinireducens DSM 20476 |
| 1 | Cellular orgnism | Sodalis glossinidius |
| 1 | Cellular orgnism | Sodalis glossinidius str. 'morsitans' |
| 1 | Cellular orgnism | Sorangium cellulosum |
| 1 | Cellular orgnism | Sorangium cellulosum 'So ce 56' |
| 1 | Cellular orgnism | Sphaerobacter thermophilus DSM 20745 |
| 1 | Cellular orgnism | Sphingobacterium spiritivorum |
| 1 | Cellular orgnism | Sphingobacterium spiritivorum ATCC 33300 |
| 1 | Cellular orgnism | Sphingobacterium spiritivorum ATCC 33861 |
| 1 | Cellular orgnism | Sphingobium chlorophenolicum |
| 1 | Cellular orgnism | Sphingobium chlorophenolicum L-1 |
| 1 | Cellular orgnism | Sphingobium japonicum |
| 1 | Cellular orgnism | Sphingobium japonicum UT26S |
| 1 | Cellular orgnism | Sphingobium xenophagum |
| 1 | Cellular orgnism | Sphingobium yanoikuyae |
| 1 | Cellular orgnism | Sphingomonas sp. A1 |
| 1 | Cellular orgnism | Sphingomonas sp. KA1 |
| 1 | Cellular orgnism | Sphingomonas sp. MM-1 |
| 1 | Cellular orgnism | Sphingomonas sp. SKA58 |
| 1 | Cellular orgnism | Sphingomonas wittichii RW1 |
| 1 | Cellular orgnism | Sphingopyxis alaskensis RB2256 |
| 1 | Cellular orgnism | Sphingopyxis macrogoltabida |
| 1 | Cellular orgnism | Spirochaeta smaragdinae DSM 11293 |
| 1 | Cellular orgnism | Spirochaeta thermophila |
| 1 | Cellular orgnism | Spirochaeta thermophila DSM 6192 |
| 1 | Cellular orgnism | Spiroplasma citri |
| 1 | Cellular orgnism | Spiroplasma kunkelii |
| 1 | Cellular orgnism | Spiroplasma kunkelii CR2-3x |
| 1 | Cellular orgnism | Spirosoma linguale DSM 74 |
| 1 | Cellular orgnism | Sporosarcina ureae |
| 1 | Cellular orgnism | Stackebrandtia nassauensis DSM 44728 |
| 1 | Cellular orgnism | Staphylococcus aureus |
| 1 | Cellular orgnism | Staphylococcus aureus 930918-3 |
| 1 | Cellular orgnism | Staphylococcus aureus A10102 |
| 1 | Cellular orgnism | Staphylococcus aureus A5937 |
| 1 | Cellular orgnism | Staphylococcus aureus A5948 |
| 1 | Cellular orgnism | Staphylococcus aureus A6224 |
| 1 | Cellular orgnism | Staphylococcus aureus A6300 |
| 1 | Cellular orgnism | Staphylococcus aureus A8115 |
| 1 | Cellular orgnism | Staphylococcus aureus A8117 |
| 1 | Cellular orgnism | Staphylococcus aureus A8796 |
| 1 | Cellular orgnism | Staphylococcus aureus A8819 |
| 1 | Cellular orgnism | Staphylococcus aureus A9299 |
| 1 | Cellular orgnism | Staphylococcus aureus A9635 |
| 1 | Cellular orgnism | Staphylococcus aureus A9719 |
| 1 | Cellular orgnism | Staphylococcus aureus A9754 |
| 1 | Cellular orgnism | Staphylococcus aureus A9763 |
| 1 | Cellular orgnism | Staphylococcus aureus A9765 |
| 1 | Cellular orgnism | Staphylococcus aureus A9781 |
| 1 | Cellular orgnism | Staphylococcus aureus D30 |
| 1 | Cellular orgnism | Staphylococcus aureus RF122 |
| 1 | Cellular orgnism | Staphylococcus aureus subsp. aureus |
| 1 | Cellular orgnism | Staphylococcus aureus subsp. aureus 132 |
| 1 | Cellular orgnism | Staphylococcus aureus subsp. aureus 55/2053 |
| 1 | Cellular orgnism | Staphylococcus aureus subsp. aureus 58-424 |
| 1 | Cellular orgnism | Staphylococcus aureus subsp. aureus 65-1322 |
| 1 | Cellular orgnism | Staphylococcus aureus subsp. aureus 68-397 |
| 1 | Cellular orgnism | Staphylococcus aureus subsp. aureus A017934/97 |
| 1 | Cellular orgnism | Staphylococcus aureus subsp. aureus ATCC 51811 |
| 1 | Cellular orgnism | Staphylococcus aureus subsp. aureus ATCC BAA-39 |
| 1 | Cellular orgnism | Staphylococcus aureus subsp. aureus Btn1260 |
| 1 | Cellular orgnism | Staphylococcus aureus subsp. aureus C101 |
| 1 | Cellular orgnism | Staphylococcus aureus subsp. aureus C160 |
| 1 | Cellular orgnism | Staphylococcus aureus subsp. aureus C427 |
| 1 | Cellular orgnism | Staphylococcus aureus subsp. aureus COL |
| 1 | Cellular orgnism | Staphylococcus aureus subsp. aureus D139 |
| 1 | Cellular orgnism | Staphylococcus aureus subsp. aureus E1410 |
| 1 | Cellular orgnism | Staphylococcus aureus subsp. aureus ED98 |
| 1 | Cellular orgnism | Staphylococcus aureus subsp. aureus EMRSA16 |
| 1 | Cellular orgnism | Staphylococcus aureus subsp. aureus H19 |
| 1 | Cellular orgnism | Staphylococcus aureus subsp. aureus JH1 |
| 1 | Cellular orgnism | Staphylococcus aureus subsp. aureus JH9 |
| 1 | Cellular orgnism | Staphylococcus aureus subsp. aureus M1015 |
| 1 | Cellular orgnism | Staphylococcus aureus subsp. aureus M809 |
| 1 | Cellular orgnism | Staphylococcus aureus subsp. aureus M876 |
| 1 | Cellular orgnism | Staphylococcus aureus subsp. aureus M899 |
| 1 | Cellular orgnism | Staphylococcus aureus subsp. aureus MN8 |
| 1 | Cellular orgnism | Staphylococcus aureus subsp. aureus MR1 |
| 1 | Cellular orgnism | Staphylococcus aureus subsp. aureus MRSA252 |
| 1 | Cellular orgnism | Staphylococcus aureus subsp. aureus MSSA476 |
| 1 | Cellular orgnism | Staphylococcus aureus subsp. aureus MW2 |
| 1 | Cellular orgnism | Staphylococcus aureus subsp. aureus Mu3 |
| 1 | Cellular orgnism | Staphylococcus aureus subsp. aureus Mu50 |
| 1 | Cellular orgnism | Staphylococcus aureus subsp. aureus Mu50-omega |
| 1 | Cellular orgnism | Staphylococcus aureus subsp. aureus N315 |
| 1 | Cellular orgnism | Staphylococcus aureus subsp. aureus NCTC 8325 |
| 1 | Cellular orgnism | Staphylococcus aureus subsp. aureus ST398 |
| 1 | Cellular orgnism | Staphylococcus aureus subsp. aureus TCH130 |
| 1 | Cellular orgnism | Staphylococcus aureus subsp. aureus TCH70 |
| 1 | Cellular orgnism | Staphylococcus aureus subsp. aureus USA300_FPR3757 |
| 1 | Cellular orgnism | Staphylococcus aureus subsp. aureus USA300_TCH1516 |
| 1 | Cellular orgnism | Staphylococcus aureus subsp. aureus USA300_TCH959 |
| 1 | Cellular orgnism | Staphylococcus aureus subsp. aureus WBG10049 |
| 1 | Cellular orgnism | Staphylococcus aureus subsp. aureus WW2703/97 |
| 1 | Cellular orgnism | Staphylococcus aureus subsp. aureus str. CF-Marseille |
| 1 | Cellular orgnism | Staphylococcus aureus subsp. aureus str. JKD6009 |
| 1 | Cellular orgnism | Staphylococcus aureus subsp. aureus str. Newman |
| 1 | Cellular orgnism | Staphylococcus capitis |
| 1 | Cellular orgnism | Staphylococcus capitis SK14 |
| 1 | Cellular orgnism | Staphylococcus caprae |
| 1 | Cellular orgnism | Staphylococcus carnosus |
| 1 | Cellular orgnism | Staphylococcus carnosus subsp. carnosus |
| 1 | Cellular orgnism | Staphylococcus carnosus subsp. carnosus TM300 |
| 1 | Cellular orgnism | Staphylococcus chromogenes |
| 1 | Cellular orgnism | Staphylococcus epidermidis |
| 1 | Cellular orgnism | Staphylococcus epidermidis ATCC 12228 |
| 1 | Cellular orgnism | Staphylococcus epidermidis BCM-HMP0060 |
| 1 | Cellular orgnism | Staphylococcus epidermidis M23864:W1 |
| 1 | Cellular orgnism | Staphylococcus epidermidis M23864:W2(grey) |
| 1 | Cellular orgnism | Staphylococcus epidermidis RP62A |
| 1 | Cellular orgnism | Staphylococcus epidermidis SK135 |
| 1 | Cellular orgnism | Staphylococcus epidermidis W23144 |
| 1 | Cellular orgnism | Staphylococcus haemolyticus |
| 1 | Cellular orgnism | Staphylococcus haemolyticus JCSC1435 |
| 1 | Cellular orgnism | Staphylococcus hominis |
| 1 | Cellular orgnism | Staphylococcus hominis SK119 |
| 1 | Cellular orgnism | Staphylococcus hominis subsp. hominis |
| 1 | Cellular orgnism | Staphylococcus lentus |
| 1 | Cellular orgnism | Staphylococcus lugdunensis |
| 1 | Cellular orgnism | Staphylococcus lugdunensis HKU09-01 |
| 1 | Cellular orgnism | Staphylococcus pasteuri |
| 1 | Cellular orgnism | Staphylococcus pseudintermedius |
| 1 | Cellular orgnism | Staphylococcus saprophyticus |
| 1 | Cellular orgnism | Staphylococcus saprophyticus subsp. saprophyticus |
| 1 | Cellular orgnism | Staphylococcus saprophyticus subsp. saprophyticus ATCC 15305 |
| 1 | Cellular orgnism | Staphylococcus sciuri |
| 1 | Cellular orgnism | Staphylococcus sciuri subsp. sciuri |
| 1 | Cellular orgnism | Staphylococcus simulans |
| 1 | Cellular orgnism | Staphylococcus simulans bv. staphylolyticus |
| 1 | Cellular orgnism | Staphylococcus sp. 693-2 |
| 1 | Cellular orgnism | Staphylococcus warneri |
| 1 | Cellular orgnism | Staphylococcus warneri L37603 |
| 1 | Cellular orgnism | Starkeya novella DSM 506 |
| 1 | Cellular orgnism | Stenotrophomonas maltophilia |
| 1 | Cellular orgnism | Stenotrophomonas maltophilia K279a |
| 1 | Cellular orgnism | Stenotrophomonas maltophilia R551-3 |
| 1 | Cellular orgnism | Stenotrophomonas sp. SKA14 |
| 1 | Cellular orgnism | Stigmatella aurantiaca |
| 1 | Cellular orgnism | Stigmatella aurantiaca DW4/3-1 |
| 1 | Cellular orgnism | Streptobacillus moniliformis |
| 1 | Cellular orgnism | Streptobacillus moniliformis DSM 12112 |
| 1 | Cellular orgnism | Streptococcus |
| 1 | Cellular orgnism | Streptococcus agalactiae |
| 1 | Cellular orgnism | Streptococcus agalactiae 18RS21 |
| 1 | Cellular orgnism | Streptococcus agalactiae 2603V/R |
| 1 | Cellular orgnism | Streptococcus agalactiae 515 |
| 1 | Cellular orgnism | Streptococcus agalactiae A909 |
| 1 | Cellular orgnism | Streptococcus agalactiae CJB111 |
| 1 | Cellular orgnism | Streptococcus agalactiae COH1 |
| 1 | Cellular orgnism | Streptococcus agalactiae H36B |
| 1 | Cellular orgnism | Streptococcus agalactiae NEM316 |
| 1 | Cellular orgnism | Streptococcus agalactiae serogroup III |
| 1 | Cellular orgnism | Streptococcus anginosus |
| 1 | Cellular orgnism | Streptococcus australis |
| 1 | Cellular orgnism | Streptococcus bovis ATCC 700338 |
| 1 | Cellular orgnism | Streptococcus cristatus |
| 1 | Cellular orgnism | Streptococcus cristatus ATCC 51100 |
| 1 | Cellular orgnism | Streptococcus downei |
| 1 | Cellular orgnism | Streptococcus dysgalactiae |
| 1 | Cellular orgnism | Streptococcus dysgalactiae subsp. equisimilis |
| 1 | Cellular orgnism | Streptococcus dysgalactiae subsp. equisimilis GGS_124 |
| 1 | Cellular orgnism | Streptococcus equi |
| 1 | Cellular orgnism | Streptococcus equi subsp. equi |
| 1 | Cellular orgnism | Streptococcus equi subsp. equi 4047 |
| 1 | Cellular orgnism | Streptococcus equi subsp. zooepidemicus |
| 1 | Cellular orgnism | Streptococcus equi subsp. zooepidemicus MGCS10565 |
| 1 | Cellular orgnism | Streptococcus equinus |
| 1 | Cellular orgnism | Streptococcus gallolyticus |
| 1 | Cellular orgnism | Streptococcus gallolyticus UCN34 |
| 1 | Cellular orgnism | Streptococcus gallolyticus subsp. gallolyticus |
| 1 | Cellular orgnism | Streptococcus gallolyticus subsp. gallolyticus TX20005 |
| 1 | Cellular orgnism | Streptococcus gordonii |
| 1 | Cellular orgnism | Streptococcus gordonii str. Challis |
| 1 | Cellular orgnism | Streptococcus gordonii str. Challis substr. CH1 |
| 1 | Cellular orgnism | Streptococcus infantarius |
| 1 | Cellular orgnism | Streptococcus infantarius subsp. infantarius |
| 1 | Cellular orgnism | Streptococcus infantarius subsp. infantarius ATCC BAA-102 |
| 1 | Cellular orgnism | Streptococcus infantis |
| 1 | Cellular orgnism | Streptococcus infantis ATCC 700779 |
| 1 | Cellular orgnism | Streptococcus infantis SK1302 |
| 1 | Cellular orgnism | Streptococcus mitis |
| 1 | Cellular orgnism | Streptococcus mitis ATCC 6249 |
| 1 | Cellular orgnism | Streptococcus mitis B6 |
| 1 | Cellular orgnism | Streptococcus mitis NCTC 12261 |
| 1 | Cellular orgnism | Streptococcus mitis SK321 |
| 1 | Cellular orgnism | Streptococcus mitis SK564 |
| 1 | Cellular orgnism | Streptococcus mitis SK597 |
| 1 | Cellular orgnism | Streptococcus mutans |
| 1 | Cellular orgnism | Streptococcus mutans NN2025 |
| 1 | Cellular orgnism | Streptococcus mutans UA159 |
| 1 | Cellular orgnism | Streptococcus oralis |
| 1 | Cellular orgnism | Streptococcus oralis ATCC 35037 |
| 1 | Cellular orgnism | Streptococcus parasanguinis |
| 1 | Cellular orgnism | Streptococcus parasanguinis ATCC 15912 |
| 1 | Cellular orgnism | Streptococcus peroris |
| 1 | Cellular orgnism | Streptococcus peroris ATCC 700780 |
| 1 | Cellular orgnism | Streptococcus pneumoniae |
| 1 | Cellular orgnism | Streptococcus pneumoniae 670-6B |
| 1 | Cellular orgnism | Streptococcus pneumoniae 70585 |
| 1 | Cellular orgnism | Streptococcus pneumoniae AP200 |
| 1 | Cellular orgnism | Streptococcus pneumoniae ATCC 700669 |
| 1 | Cellular orgnism | Streptococcus pneumoniae BS397 |
| 1 | Cellular orgnism | Streptococcus pneumoniae BS455 |
| 1 | Cellular orgnism | Streptococcus pneumoniae BS457 |
| 1 | Cellular orgnism | Streptococcus pneumoniae BS458 |
| 1 | Cellular orgnism | Streptococcus pneumoniae CCRI 1974 |
| 1 | Cellular orgnism | Streptococcus pneumoniae CCRI 1974M2 |
| 1 | Cellular orgnism | Streptococcus pneumoniae CDC0288-04 |
| 1 | Cellular orgnism | Streptococcus pneumoniae CDC1087-00 |
| 1 | Cellular orgnism | Streptococcus pneumoniae CDC1873-00 |
| 1 | Cellular orgnism | Streptococcus pneumoniae CDC3059-06 |
| 1 | Cellular orgnism | Streptococcus pneumoniae CGSP14 |
| 1 | Cellular orgnism | Streptococcus pneumoniae D39 |
| 1 | Cellular orgnism | Streptococcus pneumoniae G54 |
| 1 | Cellular orgnism | Streptococcus pneumoniae Hungary19A-6 |
| 1 | Cellular orgnism | Streptococcus pneumoniae JJA |
| 1 | Cellular orgnism | Streptococcus pneumoniae MLV-016 |
| 1 | Cellular orgnism | Streptococcus pneumoniae P1031 |
| 1 | Cellular orgnism | Streptococcus pneumoniae R6 |
| 1 | Cellular orgnism | Streptococcus pneumoniae SP-BS293 |
| 1 | Cellular orgnism | Streptococcus pneumoniae SP11-BS70 |
| 1 | Cellular orgnism | Streptococcus pneumoniae SP14-BS292 |
| 1 | Cellular orgnism | Streptococcus pneumoniae SP14-BS69 |
| 1 | Cellular orgnism | Streptococcus pneumoniae SP18-BS74 |
| 1 | Cellular orgnism | Streptococcus pneumoniae SP19-BS75 |
| 1 | Cellular orgnism | Streptococcus pneumoniae SP195 |
| 1 | Cellular orgnism | Streptococcus pneumoniae SP23-BS72 |
| 1 | Cellular orgnism | Streptococcus pneumoniae SP3-BS71 |
| 1 | Cellular orgnism | Streptococcus pneumoniae SP6-BS73 |
| 1 | Cellular orgnism | Streptococcus pneumoniae SP9-BS68 |
| 1 | Cellular orgnism | Streptococcus pneumoniae TCH8431/19A |
| 1 | Cellular orgnism | Streptococcus pneumoniae TIGR4 |
| 1 | Cellular orgnism | Streptococcus pneumoniae Taiwan19F-14 |
| 1 | Cellular orgnism | Streptococcus pneumoniae str. Canada MDR_19A |
| 1 | Cellular orgnism | Streptococcus pneumoniae str. Canada MDR_19F |
| 1 | Cellular orgnism | Streptococcus pyogenes |
| 1 | Cellular orgnism | Streptococcus pyogenes ATCC 10782 |
| 1 | Cellular orgnism | Streptococcus pyogenes M1 GAS |
| 1 | Cellular orgnism | Streptococcus pyogenes M49 591 |
| 1 | Cellular orgnism | Streptococcus pyogenes MGAS10270 |
| 1 | Cellular orgnism | Streptococcus pyogenes MGAS10394 |
| 1 | Cellular orgnism | Streptococcus pyogenes MGAS10750 |
| 1 | Cellular orgnism | Streptococcus pyogenes MGAS2096 |
| 1 | Cellular orgnism | Streptococcus pyogenes MGAS315 |
| 1 | Cellular orgnism | Streptococcus pyogenes MGAS5005 |
| 1 | Cellular orgnism | Streptococcus pyogenes MGAS6180 |
| 1 | Cellular orgnism | Streptococcus pyogenes MGAS8232 |
| 1 | Cellular orgnism | Streptococcus pyogenes MGAS9429 |
| 1 | Cellular orgnism | Streptococcus pyogenes NZ131 |
| 1 | Cellular orgnism | Streptococcus pyogenes SSI-1 |
| 1 | Cellular orgnism | Streptococcus pyogenes serotype M12 |
| 1 | Cellular orgnism | Streptococcus pyogenes serotype M3 |
| 1 | Cellular orgnism | Streptococcus pyogenes serotype M5 |
| 1 | Cellular orgnism | Streptococcus pyogenes serotype M6 |
| 1 | Cellular orgnism | Streptococcus pyogenes str. Manfredo |
| 1 | Cellular orgnism | Streptococcus salivarius |
| 1 | Cellular orgnism | Streptococcus salivarius SK126 |
| 1 | Cellular orgnism | Streptococcus sanguinis |
| 1 | Cellular orgnism | Streptococcus sanguinis SK36 |
| 1 | Cellular orgnism | Streptococcus sp. 2_1_36FAA |
| 1 | Cellular orgnism | Streptococcus sp. C150 |
| 1 | Cellular orgnism | Streptococcus sp. C300 |
| 1 | Cellular orgnism | Streptococcus sp. M143 |
| 1 | Cellular orgnism | Streptococcus sp. oral taxon 071 str. 73H25AP |
| 1 | Cellular orgnism | Streptococcus suis |
| 1 | Cellular orgnism | Streptococcus suis 05HAS68 |
| 1 | Cellular orgnism | Streptococcus suis 05ZYH33 |
| 1 | Cellular orgnism | Streptococcus suis 89/1591 |
| 1 | Cellular orgnism | Streptococcus suis 98HAH33 |
| 1 | Cellular orgnism | Streptococcus suis BM407 |
| 1 | Cellular orgnism | Streptococcus suis P1/7 |
| 1 | Cellular orgnism | Streptococcus suis SC84 |
| 1 | Cellular orgnism | Streptococcus thermophilus |
| 1 | Cellular orgnism | Streptococcus thermophilus CNRZ1066 |
| 1 | Cellular orgnism | Streptococcus thermophilus LMD-9 |
| 1 | Cellular orgnism | Streptococcus thermophilus LMG 18311 |
| 1 | Cellular orgnism | Streptococcus uberis |
| 1 | Cellular orgnism | Streptococcus uberis 0140J |
| 1 | Cellular orgnism | Streptococcus vestibularis |
| 1 | Cellular orgnism | Streptococcus vestibularis ATCC 49124 |
| 1 | Cellular orgnism | Streptomyces albulus |
| 1 | Cellular orgnism | Streptomyces albus |
| 1 | Cellular orgnism | Streptomyces albus J1074 |
| 1 | Cellular orgnism | Streptomyces avermitilis |
| 1 | Cellular orgnism | Streptomyces avermitilis MA-4680 |
| 1 | Cellular orgnism | Streptomyces clavuligerus |
| 1 | Cellular orgnism | Streptomyces clavuligerus ATCC 27064 |
| 1 | Cellular orgnism | Streptomyces coelicolor |
| 1 | Cellular orgnism | Streptomyces coelicolor A3(2) |
| 1 | Cellular orgnism | Streptomyces cyaneus |
| 1 | Cellular orgnism | Streptomyces filamentosus |
| 1 | Cellular orgnism | Streptomyces flavovirens |
| 1 | Cellular orgnism | Streptomyces ghanaensis |
| 1 | Cellular orgnism | Streptomyces ghanaensis ATCC 14672 |
| 1 | Cellular orgnism | Streptomyces griseoflavus |
| 1 | Cellular orgnism | Streptomyces griseoflavus Tu4000 |
| 1 | Cellular orgnism | Streptomyces griseus |
| 1 | Cellular orgnism | Streptomyces griseus subsp. griseus |
| 1 | Cellular orgnism | Streptomyces griseus subsp. griseus NBRC 13350 |
| 1 | Cellular orgnism | Streptomyces hygroscopicus |
| 1 | Cellular orgnism | Streptomyces hygroscopicus ATCC 53653 |
| 1 | Cellular orgnism | Streptomyces laurentii |
| 1 | Cellular orgnism | Streptomyces lavendulae |
| 1 | Cellular orgnism | Streptomyces lividans |
| 1 | Cellular orgnism | Streptomyces lividans TK24 |
| 1 | Cellular orgnism | Streptomyces natalensis |
| 1 | Cellular orgnism | Streptomyces phaeochromogenes |
| 1 | Cellular orgnism | Streptomyces pristinaespiralis |
| 1 | Cellular orgnism | Streptomyces pristinaespiralis ATCC 25486 |
| 1 | Cellular orgnism | Streptomyces rochei |
| 1 | Cellular orgnism | Streptomyces roseosporus NRRL 11379 |
| 1 | Cellular orgnism | Streptomyces roseosporus NRRL 15998 |
| 1 | Cellular orgnism | Streptomyces scabiei |
| 1 | Cellular orgnism | Streptomyces scabiei 87.22 |
| 1 | Cellular orgnism | Streptomyces sp. 44030 |
| 1 | Cellular orgnism | Streptomyces sp. 44414 |
| 1 | Cellular orgnism | Streptomyces sp. AA4 |
| 1 | Cellular orgnism | Streptomyces sp. ACT-1 |
| 1 | Cellular orgnism | Streptomyces sp. ACTE |
| 1 | Cellular orgnism | Streptomyces sp. C |
| 1 | Cellular orgnism | Streptomyces sp. EN27 |
| 1 | Cellular orgnism | Streptomyces sp. F11 |
| 1 | Cellular orgnism | Streptomyces sp. FQ1 |
| 1 | Cellular orgnism | Streptomyces sp. FR1 |
| 1 | Cellular orgnism | Streptomyces sp. HK1 |
| 1 | Cellular orgnism | Streptomyces sp. Mg1 |
| 1 | Cellular orgnism | Streptomyces sp. SPB74 |
| 1 | Cellular orgnism | Streptomyces sp. SPB78 |
| 1 | Cellular orgnism | Streptomyces sp. W9 |
| 1 | Cellular orgnism | Streptomyces sp. Y27 |
| 1 | Cellular orgnism | Streptomyces sp. ZL12 |
| 1 | Cellular orgnism | Streptomyces sp. e14 |
| 1 | Cellular orgnism | Streptomyces sp. x3 |
| 1 | Cellular orgnism | Streptomyces sviceus ATCC 29083 |
| 1 | Cellular orgnism | Streptomyces venezuelae |
| 1 | Cellular orgnism | Streptomyces violaceoruber |
| 1 | Cellular orgnism | Streptomyces violaceusniger |
| 1 | Cellular orgnism | Streptomyces violaceusniger Tu 4113 |
| 1 | Cellular orgnism | Streptomyces viridochromogenes |
| 1 | Cellular orgnism | Streptomyces viridochromogenes DSM 40736 |
| 1 | Cellular orgnism | Streptosporangium roseum |
| 1 | Cellular orgnism | Streptosporangium roseum DSM 43021 |
| 1 | Cellular orgnism | Subdoligranulum variabile DSM 15176 |
| 1 | Cellular orgnism | Sulfitobacter sp. EE-36 |
| 1 | Cellular orgnism | Sulfitobacter sp. NAS-14.1 |
| 1 | Cellular orgnism | Sulfurihydrogenibium azorense |
| 1 | Cellular orgnism | Sulfurihydrogenibium azorense Az-Fu1 |
| 1 | Cellular orgnism | Sulfurihydrogenibium sp. YO3AOP1 |
| 1 | Cellular orgnism | Sulfurihydrogenibium yellowstonense |
| 1 | Cellular orgnism | Sulfurihydrogenibium yellowstonense SS-5 |
| 1 | Cellular orgnism | Sulfurimonas autotrophica |
| 1 | Cellular orgnism | Sulfurimonas autotrophica DSM 16294 |
| 1 | Cellular orgnism | Sulfurimonas denitrificans |
| 1 | Cellular orgnism | Sulfurimonas denitrificans DSM 1251 |
| 1 | Cellular orgnism | Sulfurospirillum deleyianum |
| 1 | Cellular orgnism | Sulfurospirillum deleyianum DSM 6946 |
| 1 | Cellular orgnism | Sulfurovum sp. NBC37-1 |
| 1 | Cellular orgnism | Symbiobacterium thermophilum |
| 1 | Cellular orgnism | Symbiobacterium thermophilum IAM 14863 |
| 1 | Cellular orgnism | Synechococcus |
| 1 | Cellular orgnism | Synechococcus elongatus |
| 1 | Cellular orgnism | Synechococcus elongatus PCC 6301 |
| 1 | Cellular orgnism | Synechococcus elongatus PCC 7942 |
| 1 | Cellular orgnism | Synechococcus sp. |
| 1 | Cellular orgnism | Synechococcus sp. BL107 |
| 1 | Cellular orgnism | Synechococcus sp. CB0101 |
| 1 | Cellular orgnism | Synechococcus sp. CB0205 |
| 1 | Cellular orgnism | Synechococcus sp. CC9311 |
| 1 | Cellular orgnism | Synechococcus sp. CC9605 |
| 1 | Cellular orgnism | Synechococcus sp. CC9902 |
| 1 | Cellular orgnism | Synechococcus sp. JA-2-3B'a(2-13) |
| 1 | Cellular orgnism | Synechococcus sp. JA-3-3Ab |
| 1 | Cellular orgnism | Synechococcus sp. PCC 7002 |
| 1 | Cellular orgnism | Synechococcus sp. PCC 7335 |
| 1 | Cellular orgnism | Synechococcus sp. RCC307 |
| 1 | Cellular orgnism | Synechococcus sp. RS9916 |
| 1 | Cellular orgnism | Synechococcus sp. RS9917 |
| 1 | Cellular orgnism | Synechococcus sp. WH 5701 |
| 1 | Cellular orgnism | Synechococcus sp. WH 7803 |
| 1 | Cellular orgnism | Synechococcus sp. WH 7805 |
| 1 | Cellular orgnism | Synechococcus sp. WH 8102 |
| 1 | Cellular orgnism | Synechococcus sp. WH 8109 |
| 1 | Cellular orgnism | Synechocystis |
| 1 | Cellular orgnism | Synechocystis sp. PCC 6803 |
| 1 | Cellular orgnism | Syntrophobacter fumaroxidans MPOB |
| 1 | Cellular orgnism | Syntrophomonas wolfei subsp. wolfei |
| 1 | Cellular orgnism | Syntrophomonas wolfei subsp. wolfei str. Goettingen |
| 1 | Cellular orgnism | Syntrophothermus lipocalidus DSM 12680 |
| 1 | Cellular orgnism | Syntrophus aciditrophicus SB |
| 1 | Cellular orgnism | Teredinibacter turnerae |
| 1 | Cellular orgnism | Teredinibacter turnerae T7901 |
| 1 | Cellular orgnism | Tetragenococcus halophilus |
| 1 | Cellular orgnism | Thalassiobium sp. R2A62 |
| 1 | Cellular orgnism | Thauera sp. MZ1T |
| 1 | Cellular orgnism | Thermaerobacter marianensis DSM 12885 |
| 1 | Cellular orgnism | Thermanaerovibrio acidaminovorans DSM 6589 |
| 1 | Cellular orgnism | Thermincola potens JR |
| 1 | Cellular orgnism | Thermoanaerobacter brockii |
| 1 | Cellular orgnism | Thermoanaerobacter brockii subsp. finnii Ako-1 |
| 1 | Cellular orgnism | Thermoanaerobacter ethanolicus |
| 1 | Cellular orgnism | Thermoanaerobacter ethanolicus CCSD1 |
| 1 | Cellular orgnism | Thermoanaerobacter italicus Ab9 |
| 1 | Cellular orgnism | Thermoanaerobacter mathranii |
| 1 | Cellular orgnism | Thermoanaerobacter mathranii subsp. mathranii str. A3 |
| 1 | Cellular orgnism | Thermoanaerobacter pseudethanolicus ATCC 33223 |
| 1 | Cellular orgnism | Thermoanaerobacter sp. X513 |
| 1 | Cellular orgnism | Thermoanaerobacter sp. X514 |
| 1 | Cellular orgnism | Thermoanaerobacter sp. X561 |
| 1 | Cellular orgnism | Thermoanaerobacter tengcongensis MB4 |
| 1 | Cellular orgnism | Thermoanaerobacter wiegelii Rt8.B1 |
| 1 | Cellular orgnism | Thermoanaerobacterium thermosaccharolyticum |
| 1 | Cellular orgnism | Thermoanaerobacterium thermosaccharolyticum DSM 571 |
| 1 | Cellular orgnism | Thermoanaerobacterium xylanolyticum LX-11 |
| 1 | Cellular orgnism | Thermobaculum terrenum ATCC BAA-798 |
| 1 | Cellular orgnism | Thermobifida fusca |
| 1 | Cellular orgnism | Thermobifida fusca YX |
| 1 | Cellular orgnism | Thermobispora bispora |
| 1 | Cellular orgnism | Thermobispora bispora DSM 43833 |
| 1 | Cellular orgnism | Thermocrinis albus DSM 14484 |
| 1 | Cellular orgnism | Thermodesulfovibrio yellowstonii |
| 1 | Cellular orgnism | Thermodesulfovibrio yellowstonii DSM 11347 |
| 1 | Cellular orgnism | Thermomicrobium roseum |
| 1 | Cellular orgnism | Thermomicrobium roseum DSM 5159 |
| 1 | Cellular orgnism | Thermomonospora curvata |
| 1 | Cellular orgnism | Thermomonospora curvata DSM 43183 |
| 1 | Cellular orgnism | Thermosediminibacter oceani DSM 16646 |
| 1 | Cellular orgnism | Thermosinus carboxydivorans Nor1 |
| 1 | Cellular orgnism | Thermosipho africanus TCF52B |
| 1 | Cellular orgnism | Thermosipho melanesiensis BI429 |
| 1 | Cellular orgnism | Thermosynechococcus elongatus |
| 1 | Cellular orgnism | Thermosynechococcus elongatus BP-1 |
| 1 | Cellular orgnism | Thermotoga lettingae TMO |
| 1 | Cellular orgnism | Thermotoga maritima |
| 1 | Cellular orgnism | Thermotoga maritima MSB8 |
| 1 | Cellular orgnism | Thermotoga naphthophila |
| 1 | Cellular orgnism | Thermotoga naphthophila RKU-10 |
| 1 | Cellular orgnism | Thermotoga neapolitana |
| 1 | Cellular orgnism | Thermotoga neapolitana DSM 4359 |
| 1 | Cellular orgnism | Thermotoga petrophila |
| 1 | Cellular orgnism | Thermotoga petrophila RKU-1 |
| 1 | Cellular orgnism | Thermotoga sp. RQ2 |
| 1 | Cellular orgnism | Thermotogales bacterium mesG1.Ag.4.2 |
| 1 | Cellular orgnism | Thermovibrio ammonificans |
| 1 | Cellular orgnism | Thermus aquaticus |
| 1 | Cellular orgnism | Thermus aquaticus Y51MC23 |
| 1 | Cellular orgnism | Thermus scotoductus |
| 1 | Cellular orgnism | Thermus scotoductus SA-01 |
| 1 | Cellular orgnism | Thermus sp. 4C |
| 1 | Cellular orgnism | Thermus thermophilus |
| 1 | Cellular orgnism | Thermus thermophilus HB27 |
| 1 | Cellular orgnism | Thermus thermophilus HB8 |
| 1 | Cellular orgnism | Thioalkalivibrio sp. HL-EbGR7 |
| 1 | Cellular orgnism | Thioalkalivibrio sp. K90mix |
| 1 | Cellular orgnism | Thiobacillus denitrificans |
| 1 | Cellular orgnism | Thiobacillus denitrificans ATCC 25259 |
| 1 | Cellular orgnism | Thiomicrospira crunogena |
| 1 | Cellular orgnism | Thiomicrospira crunogena XCL-2 |
| 1 | Cellular orgnism | Thiomonas intermedia K12 |
| 1 | Cellular orgnism | Thiomonas sp. 3As |
| 1 | Cellular orgnism | Tolumonas auensis DSM 9187 |
| 1 | Cellular orgnism | Tomato big bud phytoplasma |
| 1 | Cellular orgnism | Treponema denticola |
| 1 | Cellular orgnism | Treponema denticola ATCC 35405 |
| 1 | Cellular orgnism | Treponema pallidum |
| 1 | Cellular orgnism | Treponema pallidum subsp. pallidum SS14 |
| 1 | Cellular orgnism | Treponema pallidum subsp. pallidum str. Nichols |
| 1 | Cellular orgnism | Treponema phagedenis |
| 1 | Cellular orgnism | Treponema vincentii |
| 1 | Cellular orgnism | Treponema vincentii ATCC 35580 |
| 1 | Cellular orgnism | Trichodesmium erythraeum |
| 1 | Cellular orgnism | Trichodesmium erythraeum IMS101 |
| 1 | Cellular orgnism | Tropheryma whipplei |
| 1 | Cellular orgnism | Tropheryma whipplei TW08/27 |
| 1 | Cellular orgnism | Tropheryma whipplei str. Twist |
| 1 | Cellular orgnism | Truepera radiovictrix DSM 17093 |
| 1 | Cellular orgnism | Tsukamurella paurometabola |
| 1 | Cellular orgnism | Tsukamurella paurometabola DSM 20162 |
| 1 | Cellular orgnism | Ureaplasma parvum |
| 1 | Cellular orgnism | Ureaplasma parvum serovar 1 str. ATCC 27813 |
| 1 | Cellular orgnism | Ureaplasma parvum serovar 14 str. ATCC 33697 |
| 1 | Cellular orgnism | Ureaplasma parvum serovar 3 |
| 1 | Cellular orgnism | Ureaplasma parvum serovar 3 str. ATCC 27815 |
| 1 | Cellular orgnism | Ureaplasma parvum serovar 3 str. ATCC 700970 |
| 1 | Cellular orgnism | Ureaplasma parvum serovar 6 |
| 1 | Cellular orgnism | Ureaplasma parvum serovar 6 str. ATCC 27818 |
| 1 | Cellular orgnism | Ureaplasma urealyticum |
| 1 | Cellular orgnism | Ureaplasma urealyticum serovar 10 str. ATCC 33699 |
| 1 | Cellular orgnism | Ureaplasma urealyticum serovar 11 str. ATCC 33695 |
| 1 | Cellular orgnism | Ureaplasma urealyticum serovar 12 str. ATCC 33696 |
| 1 | Cellular orgnism | Ureaplasma urealyticum serovar 13 str. ATCC 33698 |
| 1 | Cellular orgnism | Ureaplasma urealyticum serovar 2 str. ATCC 27814 |
| 1 | Cellular orgnism | Ureaplasma urealyticum serovar 4 str. ATCC 27816 |
| 1 | Cellular orgnism | Ureaplasma urealyticum serovar 5 str. ATCC 27817 |
| 1 | Cellular orgnism | Ureaplasma urealyticum serovar 7 str. ATCC 27819 |
| 1 | Cellular orgnism | Ureaplasma urealyticum serovar 8 str. ATCC 27618 |
| 1 | Cellular orgnism | Ureaplasma urealyticum serovar 9 str. ATCC 33175 |
| 1 | Cellular orgnism | Variovorax paradoxus |
| 1 | Cellular orgnism | Variovorax paradoxus S110 |
| 1 | Cellular orgnism | Veillonella atypica |
| 1 | Cellular orgnism | Veillonella atypica ACS-049-V-Sch6 |
| 1 | Cellular orgnism | Veillonella atypica ACS-134-V-Col7a |
| 1 | Cellular orgnism | Veillonella dispar |
| 1 | Cellular orgnism | Veillonella dispar ATCC 17748 |
| 1 | Cellular orgnism | Veillonella parvula |
| 1 | Cellular orgnism | Veillonella parvula ATCC 17745 |
| 1 | Cellular orgnism | Veillonella parvula DSM 2008 |
| 1 | Cellular orgnism | Veillonella sp. 3_1_44 |
| 1 | Cellular orgnism | Veillonella sp. 6_1_27 |
| 1 | Cellular orgnism | Verminephrobacter eiseniae |
| 1 | Cellular orgnism | Verminephrobacter eiseniae EF01-2 |
| 1 | Cellular orgnism | Verrucomicrobiae bacterium DG1235 |
| 1 | Cellular orgnism | Verrucomicrobium spinosum |
| 1 | Cellular orgnism | Verrucomicrobium spinosum DSM 4136 |
| 1 | Cellular orgnism | Vibrio alginolyticus |
| 1 | Cellular orgnism | Vibrio alginolyticus 12G01 |
| 1 | Cellular orgnism | Vibrio alginolyticus 40B |
| 1 | Cellular orgnism | Vibrio brasiliensis |
| 1 | Cellular orgnism | Vibrio caribbenthicus ATCC BAA-2122 |
| 1 | Cellular orgnism | Vibrio cholerae |
| 1 | Cellular orgnism | Vibrio cholerae 12129(1) |
| 1 | Cellular orgnism | Vibrio cholerae 1587 |
| 1 | Cellular orgnism | Vibrio cholerae 2740-80 |
| 1 | Cellular orgnism | Vibrio cholerae 623-39 |
| 1 | Cellular orgnism | Vibrio cholerae AM-19226 |
| 1 | Cellular orgnism | Vibrio cholerae B33 |
| 1 | Cellular orgnism | Vibrio cholerae BX 330286 |
| 1 | Cellular orgnism | Vibrio cholerae CT 5369-93 |
| 1 | Cellular orgnism | Vibrio cholerae INDRE 91/1 |
| 1 | Cellular orgnism | Vibrio cholerae M66-2 |
| 1 | Cellular orgnism | Vibrio cholerae MAK 757 |
| 1 | Cellular orgnism | Vibrio cholerae MJ-1236 |
| 1 | Cellular orgnism | Vibrio cholerae MO10 |
| 1 | Cellular orgnism | Vibrio cholerae MZO-2 |
| 1 | Cellular orgnism | Vibrio cholerae MZO-3 |
| 1 | Cellular orgnism | Vibrio cholerae NCTC 8457 |
| 1 | Cellular orgnism | Vibrio cholerae O1 |
| 1 | Cellular orgnism | Vibrio cholerae O1 biovar El Tor |
| 1 | Cellular orgnism | Vibrio cholerae O1 biovar El Tor str. N16961 |
| 1 | Cellular orgnism | Vibrio cholerae O395 |
| 1 | Cellular orgnism | Vibrio cholerae RC27 |
| 1 | Cellular orgnism | Vibrio cholerae RC385 |
| 1 | Cellular orgnism | Vibrio cholerae RC9 |
| 1 | Cellular orgnism | Vibrio cholerae TM 11079-80 |
| 1 | Cellular orgnism | Vibrio cholerae TMA 21 |
| 1 | Cellular orgnism | Vibrio cholerae V51 |
| 1 | Cellular orgnism | Vibrio cholerae V52 |
| 1 | Cellular orgnism | Vibrio cholerae bv. albensis |
| 1 | Cellular orgnism | Vibrio cholerae bv. albensis VL426 |
| 1 | Cellular orgnism | Vibrio coralliilyticus |
| 1 | Cellular orgnism | Vibrio coralliilyticus ATCC BAA-450 |
| 1 | Cellular orgnism | Vibrio fischeri ES114 |
| 1 | Cellular orgnism | Vibrio fischeri MJ11 |
| 1 | Cellular orgnism | Vibrio fluvialis |
| 1 | Cellular orgnism | Vibrio furnissii |
| 1 | Cellular orgnism | Vibrio furnissii CIP 102972 |
| 1 | Cellular orgnism | Vibrio harveyi |
| 1 | Cellular orgnism | Vibrio harveyi 1DA3 |
| 1 | Cellular orgnism | Vibrio harveyi ATCC BAA-1116 |
| 1 | Cellular orgnism | Vibrio harveyi HY01 |
| 1 | Cellular orgnism | Vibrio metschnikovii |
| 1 | Cellular orgnism | Vibrio metschnikovii CIP 69.14 |
| 1 | Cellular orgnism | Vibrio mimicus |
| 1 | Cellular orgnism | Vibrio mimicus VM223 |
| 1 | Cellular orgnism | Vibrio mimicus VM573 |
| 1 | Cellular orgnism | Vibrio mimicus VM603 |
| 1 | Cellular orgnism | Vibrio nigripulchritudo |
| 1 | Cellular orgnism | Vibrio orientalis |
| 1 | Cellular orgnism | Vibrio orientalis CIP 102891 |
| 1 | Cellular orgnism | Vibrio parahaemolyticus |
| 1 | Cellular orgnism | Vibrio parahaemolyticus 16 |
| 1 | Cellular orgnism | Vibrio parahaemolyticus AN-5034 |
| 1 | Cellular orgnism | Vibrio parahaemolyticus AQ3810 |
| 1 | Cellular orgnism | Vibrio parahaemolyticus AQ4037 |
| 1 | Cellular orgnism | Vibrio parahaemolyticus K5030 |
| 1 | Cellular orgnism | Vibrio parahaemolyticus Peru-466 |
| 1 | Cellular orgnism | Vibrio parahaemolyticus RIMD 2210633 |
| 1 | Cellular orgnism | Vibrio shilonii |
| 1 | Cellular orgnism | Vibrio shilonii AK1 |
| 1 | Cellular orgnism | Vibrio sinaloensis |
| 1 | Cellular orgnism | Vibrio sp. 09022 |
| 1 | Cellular orgnism | Vibrio sp. 0908 |
| 1 | Cellular orgnism | Vibrio sp. 23023 |
| 1 | Cellular orgnism | Vibrio sp. 41 |
| 1 | Cellular orgnism | Vibrio sp. AND4 |
| 1 | Cellular orgnism | Vibrio sp. Ex25 |
| 1 | Cellular orgnism | Vibrio sp. MED222 |
| 1 | Cellular orgnism | Vibrio sp. RC341 |
| 1 | Cellular orgnism | Vibrio sp. RC586 |
| 1 | Cellular orgnism | Vibrio sp. TC68 |
| 1 | Cellular orgnism | Vibrio splendidus |
| 1 | Cellular orgnism | Vibrio splendidus 12B01 |
| 1 | Cellular orgnism | Vibrio splendidus LGP32 |
| 1 | Cellular orgnism | Vibrio tapetis |
| 1 | Cellular orgnism | Vibrio vulnificus |
| 1 | Cellular orgnism | Vibrio vulnificus CMCP6 |
| 1 | Cellular orgnism | Vibrio vulnificus YJ016 |
| 1 | Cellular orgnism | Vibrionales bacterium SWAT-3 |
| 1 | Cellular orgnism | Victivallis vadensis ATCC BAA-548 |
| 1 | Cellular orgnism | Waddlia chondrophila |
| 1 | Cellular orgnism | Waddlia chondrophila WSU 86-1044 |
| 1 | Cellular orgnism | Weissella cibaria |
| 1 | Cellular orgnism | Weissella paramesenteroides |
| 1 | Cellular orgnism | Weissella paramesenteroides ATCC 33313 |
| 1 | Cellular orgnism | Wigglesworthia glossinidia |
| 1 | Cellular orgnism | Wigglesworthia glossinidia endosymbiont of Glossina brevipalpis |
| 1 | Cellular orgnism | Wolbachia endosymbiont of Brugia malayi |
| 1 | Cellular orgnism | Wolbachia endosymbiont of Culex quinquefasciatus |
| 1 | Cellular orgnism | Wolbachia endosymbiont of Culex quinquefasciatus JHB |
| 1 | Cellular orgnism | Wolbachia endosymbiont of Culex quinquefasciatus Pel |
| 1 | Cellular orgnism | Wolbachia endosymbiont of Drosophila ananassae |
| 1 | Cellular orgnism | Wolbachia endosymbiont of Drosophila melanogaster |
| 1 | Cellular orgnism | Wolbachia endosymbiont of Drosophila simulans |
| 1 | Cellular orgnism | Wolbachia endosymbiont of Drosophila willistoni |
| 1 | Cellular orgnism | Wolbachia endosymbiont of Drosophila willistoni TSC#14030-0811.24 |
| 1 | Cellular orgnism | Wolbachia endosymbiont of Muscidifurax uniraptor |
| 1 | Cellular orgnism | Wolbachia endosymbiont strain TRS of Brugia malayi |
| 1 | Cellular orgnism | Wolbachia sp. wRi |
| 1 | Cellular orgnism | Wolinella succinogenes |
| 1 | Cellular orgnism | Wolinella succinogenes DSM 1740 |
| 1 | Cellular orgnism | Xanthobacter autotrophicus |
| 1 | Cellular orgnism | Xanthobacter autotrophicus Py2 |
| 1 | Cellular orgnism | Xanthomonas albilineans |
| 1 | Cellular orgnism | Xanthomonas axonopodis |
| 1 | Cellular orgnism | Xanthomonas axonopodis pv. citri |
| 1 | Cellular orgnism | Xanthomonas axonopodis pv. citri str. 306 |
| 1 | Cellular orgnism | Xanthomonas axonopodis pv. glycines |
| 1 | Cellular orgnism | Xanthomonas campestris |
| 1 | Cellular orgnism | Xanthomonas campestris pv. campestris |
| 1 | Cellular orgnism | Xanthomonas campestris pv. campestris str. 8004 |
| 1 | Cellular orgnism | Xanthomonas campestris pv. campestris str. ATCC 33913 |
| 1 | Cellular orgnism | Xanthomonas campestris pv. campestris str. B100 |
| 1 | Cellular orgnism | Xanthomonas campestris pv. musacearum |
| 1 | Cellular orgnism | Xanthomonas campestris pv. musacearum NCPPB4381 |
| 1 | Cellular orgnism | Xanthomonas campestris pv. vasculorum |
| 1 | Cellular orgnism | Xanthomonas campestris pv. vasculorum NCPPB702 |
| 1 | Cellular orgnism | Xanthomonas campestris pv. vesicatoria str. 85-10 |
| 1 | Cellular orgnism | Xanthomonas citri |
| 1 | Cellular orgnism | Xanthomonas euvesicatoria |
| 1 | Cellular orgnism | Xanthomonas fuscans |
| 1 | Cellular orgnism | Xanthomonas fuscans subsp. aurantifolii |
| 1 | Cellular orgnism | Xanthomonas fuscans subsp. aurantifolii str. ICPB 10535 |
| 1 | Cellular orgnism | Xanthomonas fuscans subsp. aurantifolii str. ICPB 11122 |
| 1 | Cellular orgnism | Xanthomonas oryzae |
| 1 | Cellular orgnism | Xanthomonas oryzae pv. oryzae |
| 1 | Cellular orgnism | Xanthomonas oryzae pv. oryzae KACC10331 |
| 1 | Cellular orgnism | Xanthomonas oryzae pv. oryzae MAFF 311018 |
| 1 | Cellular orgnism | Xanthomonas oryzae pv. oryzae PXO99A |
| 1 | Cellular orgnism | Xanthomonas oryzae pv. oryzicola |
| 1 | Cellular orgnism | Xanthomonas oryzae pv. oryzicola BLS256 |
| 1 | Cellular orgnism | Xenorhabdus bovienii |
| 1 | Cellular orgnism | Xenorhabdus bovienii SS-2004 |
| 1 | Cellular orgnism | Xenorhabdus nematophila |
| 1 | Cellular orgnism | Xenorhabdus nematophila ATCC 19061 |
| 1 | Cellular orgnism | Xylanimonas cellulosilytica DSM 15894 |
| 1 | Cellular orgnism | Xylella fastidiosa |
| 1 | Cellular orgnism | Xylella fastidiosa 9a5c |
| 1 | Cellular orgnism | Xylella fastidiosa Dixon |
| 1 | Cellular orgnism | Xylella fastidiosa M12 |
| 1 | Cellular orgnism | Xylella fastidiosa M23 |
| 1 | Cellular orgnism | Xylella fastidiosa Temecula1 |
| 1 | Cellular orgnism | Xylella fastidiosa subsp. sandyi |
| 1 | Cellular orgnism | Yersinia aldovae |
| 1 | Cellular orgnism | Yersinia aldovae ATCC 35236 |
| 1 | Cellular orgnism | Yersinia bercovieri |
| 1 | Cellular orgnism | Yersinia bercovieri ATCC 43970 |
| 1 | Cellular orgnism | Yersinia enterocolitica |
| 1 | Cellular orgnism | Yersinia enterocolitica (type O:9) |
| 1 | Cellular orgnism | Yersinia enterocolitica subsp. enterocolitica |
| 1 | Cellular orgnism | Yersinia enterocolitica subsp. enterocolitica 8081 |
| 1 | Cellular orgnism | Yersinia frederiksenii |
| 1 | Cellular orgnism | Yersinia frederiksenii ATCC 33641 |
| 1 | Cellular orgnism | Yersinia intermedia |
| 1 | Cellular orgnism | Yersinia intermedia ATCC 29909 |
| 1 | Cellular orgnism | Yersinia kristensenii |
| 1 | Cellular orgnism | Yersinia kristensenii ATCC 33638 |
| 1 | Cellular orgnism | Yersinia mollaretii |
| 1 | Cellular orgnism | Yersinia mollaretii ATCC 43969 |
| 1 | Cellular orgnism | Yersinia pestis |
| 1 | Cellular orgnism | Yersinia pestis Angola |
| 1 | Cellular orgnism | Yersinia pestis Antiqua |
| 1 | Cellular orgnism | Yersinia pestis CA88-4125 |
| 1 | Cellular orgnism | Yersinia pestis CO92 |
| 1 | Cellular orgnism | Yersinia pestis FV-1 |
| 1 | Cellular orgnism | Yersinia pestis KIM 10 |
| 1 | Cellular orgnism | Yersinia pestis KIM D27 |
| 1 | Cellular orgnism | Yersinia pestis Nepal516 |
| 1 | Cellular orgnism | Yersinia pestis Pestoides A |
| 1 | Cellular orgnism | Yersinia pestis Pestoides F |
| 1 | Cellular orgnism | Yersinia pestis Z176003 |
| 1 | Cellular orgnism | Yersinia pestis biovar Antiqua str. B42003004 |
| 1 | Cellular orgnism | Yersinia pestis biovar Antiqua str. E1979001 |
| 1 | Cellular orgnism | Yersinia pestis biovar Antiqua str. UG05-0454 |
| 1 | Cellular orgnism | Yersinia pestis biovar Mediaevalis str. K1973002 |
| 1 | Cellular orgnism | Yersinia pestis biovar Microtus str. 91001 |
| 1 | Cellular orgnism | Yersinia pestis biovar Orientalis str. F1991016 |
| 1 | Cellular orgnism | Yersinia pestis biovar Orientalis str. IP275 |
| 1 | Cellular orgnism | Yersinia pestis biovar Orientalis str. India 195 |
| 1 | Cellular orgnism | Yersinia pestis biovar Orientalis str. MG05-1020 |
| 1 | Cellular orgnism | Yersinia pestis biovar Orientalis str. PEXU2 |
| 1 | Cellular orgnism | Yersinia pseudotuberculosis |
| 1 | Cellular orgnism | Yersinia pseudotuberculosis IP 31758 |
| 1 | Cellular orgnism | Yersinia pseudotuberculosis IP 32953 |
| 1 | Cellular orgnism | Yersinia pseudotuberculosis PB1/+ |
| 1 | Cellular orgnism | Yersinia pseudotuberculosis YPIII |
| 1 | Cellular orgnism | Yersinia rohdei |
| 1 | Cellular orgnism | Yersinia rohdei ATCC 43380 |
| 1 | Cellular orgnism | Yersinia ruckeri |
| 1 | Cellular orgnism | Yersinia ruckeri ATCC 29473 |
| 1 | Cellular orgnism | Zunongwangia profunda SM-A87 |
| 1 | Cellular orgnism | Zymomonas mobilis |
| 1 | Cellular orgnism | Zymomonas mobilis subsp. mobilis |
| 1 | Cellular orgnism | Zymomonas mobilis subsp. mobilis ATCC 10988 |
| 1 | Cellular orgnism | Zymomonas mobilis subsp. mobilis CP4 |
| 1 | Cellular orgnism | Zymomonas mobilis subsp. mobilis NCIMB 11163 |
| 1 | Cellular orgnism | Zymomonas mobilis subsp. mobilis ZM4 |
| 1 | Cellular orgnism | alpha proteobacterium BAL199 |
| 1 | Cellular orgnism | alpha proteobacterium HIMB114 |
| 1 | Cellular orgnism | alpha proteobacterium endosymbiont of Amoeba proteus |
| 1 | Cellular orgnism | bacterium Ellin514 |
| 1 | Cellular orgnism | beta proteobacterium KB13 |
| 1 | Cellular orgnism | candidate division TM7 genomosp. GTL1 |
| 1 | Cellular orgnism | candidate division TM7 single-cell isolate TM7a |
| 1 | Cellular orgnism | candidate division TM7 single-cell isolate TM7b |
| 1 | Cellular orgnism | candidate division TM7 single-cell isolate TM7c |
| 1 | Cellular orgnism | cyanobacterium UCYN-A |
| 1 | Cellular orgnism | delta proteobacterium MLMS-1 |
| 1 | Cellular orgnism | delta proteobacterium NaphS2 |
| 1 | Cellular orgnism | endophytic bacterium LOB-07 |
| 1 | Cellular orgnism | gamma proteobacterium HTCC2207 |
| 1 | Cellular orgnism | gamma proteobacterium HTCC5015 |
| 1 | Cellular orgnism | gamma proteobacterium HdN1 |
| 1 | Cellular orgnism | gamma proteobacterium NOR5-3 |
| 1 | Cellular orgnism | gamma proteobacterium NOR51-B |
| 1 | Cellular orgnism | marine actinobacterium PHSC20C1 |
| 1 | Cellular orgnism | marine gamma proteobacterium HTCC2080 |
| 1 | Cellular orgnism | marine gamma proteobacterium HTCC2143 |
| 1 | Cellular orgnism | marine gamma proteobacterium HTCC2148 |
| 1 | Cellular orgnism | uncultured Termite group 1 bacterium |
| 1 | Cellular orgnism | uncultured Termite group 1 bacterium phylotype Rs-D17 |
| 1 | Cellular orgnism | uncultured bacterium |
| 1 | Cellular orgnism | uncultured eubacterium pIE1115 |
| 1 | Cellular orgnism | unidentified eubacterium SCB49 |
| 1 | Cellular orgnism | Acidianus ambivalens |
| 1 | Cellular orgnism | Acidianus hospitalis |
| 1 | Cellular orgnism | Acidilobus saccharovorans 345-15 |
| 1 | Cellular orgnism | Aciduliprofundum boonei T469 |
| 1 | Cellular orgnism | Aeropyrum pernix |
| 1 | Cellular orgnism | Aeropyrum pernix K1 |
| 1 | Cellular orgnism | Archaeoglobus fulgidus |
| 1 | Cellular orgnism | Archaeoglobus fulgidus DSM 4304 |
| 1 | Cellular orgnism | Archaeoglobus profundus |
| 1 | Cellular orgnism | Archaeoglobus profundus DSM 5631 |
| 1 | Cellular orgnism | Caldivirga maquilingensis |
| 1 | Cellular orgnism | Caldivirga maquilingensis IC-167 |
| 1 | Cellular orgnism | Candidatus Korarchaeum cryptofilum OPF8 |
| 1 | Cellular orgnism | Cenarchaeum symbiosum |
| 1 | Cellular orgnism | Cenarchaeum symbiosum A |
| 1 | Cellular orgnism | Desulfurococcus |
| 1 | Cellular orgnism | Desulfurococcus kamchatkensis 1221n |
| 1 | Cellular orgnism | Desulfurococcus mucosus |
| 1 | Cellular orgnism | Ferroglobus placidus |
| 1 | Cellular orgnism | Ferroglobus placidus DSM 10642 |
| 1 | Cellular orgnism | Ferroplasma acidarmanus fer1 |
| 1 | Cellular orgnism | Haladaptatus paucihalophilus DX253 |
| 1 | Cellular orgnism | Halalkalicoccus jeotgali B3 |
| 1 | Cellular orgnism | Haloarcula |
| 1 | Cellular orgnism | Haloarcula marismortui |
| 1 | Cellular orgnism | Haloarcula marismortui ATCC 43049 |
| 1 | Cellular orgnism | Haloarcula sp. AS7094 |
| 1 | Cellular orgnism | Halobacterium salinarum |
| 1 | Cellular orgnism | Halobacterium salinarum R1 |
| 1 | Cellular orgnism | Halobacterium sp. GN101 |
| 1 | Cellular orgnism | Halobacterium sp. NRC-1 |
| 1 | Cellular orgnism | Haloferax volcanii |
| 1 | Cellular orgnism | Haloferax volcanii DS2 |
| 1 | Cellular orgnism | Halogeometricum borinquense |
| 1 | Cellular orgnism | Halogeometricum borinquense DSM 11551 |
| 1 | Cellular orgnism | Halomicrobium mukohataei |
| 1 | Cellular orgnism | Halomicrobium mukohataei DSM 12286 |
| 1 | Cellular orgnism | Haloquadratum walsbyi |
| 1 | Cellular orgnism | Haloquadratum walsbyi DSM 16790 |
| 1 | Cellular orgnism | Halorhabdus utahensis |
| 1 | Cellular orgnism | Halorhabdus utahensis DSM 12940 |
| 1 | Cellular orgnism | Halorubrum lacusprofundi |
| 1 | Cellular orgnism | Halorubrum lacusprofundi ATCC 49239 |
| 1 | Cellular orgnism | Halorubrum saccharovorum |
| 1 | Cellular orgnism | Haloterrigena thermotolerans |
| 1 | Cellular orgnism | Haloterrigena turkmenica |
| 1 | Cellular orgnism | Haloterrigena turkmenica DSM 5511 |
| 1 | Cellular orgnism | Hyperthermus butylicus |
| 1 | Cellular orgnism | Hyperthermus butylicus DSM 5456 |
| 1 | Cellular orgnism | Ignicoccus hospitalis KIN4/I |
| 1 | Cellular orgnism | Ignisphaera aggregans |
| 1 | Cellular orgnism | Ignisphaera aggregans DSM 17230 |
| 1 | Cellular orgnism | Metallosphaera sedula |
| 1 | Cellular orgnism | Metallosphaera sedula DSM 5348 |
| 1 | Cellular orgnism | Methanobrevibacter ruminantium |
| 1 | Cellular orgnism | Methanobrevibacter ruminantium M1 |
| 1 | Cellular orgnism | Methanobrevibacter smithii |
| 1 | Cellular orgnism | Methanobrevibacter smithii ATCC 35061 |
| 1 | Cellular orgnism | Methanobrevibacter smithii DSM 2374 |
| 1 | Cellular orgnism | Methanobrevibacter smithii DSM 2375 |
| 1 | Cellular orgnism | Methanocaldococcus fervens AG86 |
| 1 | Cellular orgnism | Methanocaldococcus infernus |
| 1 | Cellular orgnism | Methanocaldococcus infernus ME |
| 1 | Cellular orgnism | Methanocaldococcus jannaschii |
| 1 | Cellular orgnism | Methanocaldococcus jannaschii DSM 2661 |
| 1 | Cellular orgnism | Methanocaldococcus sp. FS406-22 |
| 1 | Cellular orgnism | Methanocaldococcus vulcanius M7 |
| 1 | Cellular orgnism | Methanocella paludicola SANAE |
| 1 | Cellular orgnism | Methanococcoides burtonii |
| 1 | Cellular orgnism | Methanococcoides burtonii DSM 6242 |
| 1 | Cellular orgnism | Methanococcus aeolicus |
| 1 | Cellular orgnism | Methanococcus aeolicus Nankai-3 |
| 1 | Cellular orgnism | Methanococcus maripaludis |
| 1 | Cellular orgnism | Methanococcus maripaludis C5 |
| 1 | Cellular orgnism | Methanococcus maripaludis C6 |
| 1 | Cellular orgnism | Methanococcus maripaludis C7 |
| 1 | Cellular orgnism | Methanococcus maripaludis S2 |
| 1 | Cellular orgnism | Methanococcus vannielii |
| 1 | Cellular orgnism | Methanococcus vannielii SB |
| 1 | Cellular orgnism | Methanococcus voltae |
| 1 | Cellular orgnism | Methanococcus voltae A3 |
| 1 | Cellular orgnism | Methanocorpusculum labreanum |
| 1 | Cellular orgnism | Methanocorpusculum labreanum Z |
| 1 | Cellular orgnism | Methanoculleus marisnigri JR1 |
| 1 | Cellular orgnism | Methanohalobium evestigatum |
| 1 | Cellular orgnism | Methanohalobium evestigatum Z-7303 |
| 1 | Cellular orgnism | Methanohalophilus mahii |
| 1 | Cellular orgnism | Methanohalophilus mahii DSM 5219 |
| 1 | Cellular orgnism | Methanoplanus petrolearius DSM 11571 |
| 1 | Cellular orgnism | Methanopyrus kandleri |
| 1 | Cellular orgnism | Methanopyrus kandleri AV19 |
| 1 | Cellular orgnism | Methanosaeta thermophila PT |
| 1 | Cellular orgnism | Methanosarcina acetivorans |
| 1 | Cellular orgnism | Methanosarcina acetivorans C2A |
| 1 | Cellular orgnism | Methanosarcina barkeri |
| 1 | Cellular orgnism | Methanosarcina barkeri str. Fusaro |
| 1 | Cellular orgnism | Methanosarcina mazei |
| 1 | Cellular orgnism | Methanosarcina mazei Go1 |
| 1 | Cellular orgnism | Methanosphaera stadtmanae |
| 1 | Cellular orgnism | Methanosphaera stadtmanae DSM 3091 |
| 1 | Cellular orgnism | Methanosphaerula palustris |
| 1 | Cellular orgnism | Methanosphaerula palustris E1-9c |
| 1 | Cellular orgnism | Methanospirillum hungatei JF-1 |
| 1 | Cellular orgnism | Methanothermobacter marburgensis |
| 1 | Cellular orgnism | Methanothermobacter marburgensis str. Marburg |
| 1 | Cellular orgnism | Methanothermobacter thermautotrophicus |
| 1 | Cellular orgnism | Methanothermobacter thermautotrophicus str. Delta H |
| 1 | Cellular orgnism | Methanothermococcus okinawensis |
| 1 | Cellular orgnism | Methanothermococcus okinawensis IH1 |
| 1 | Cellular orgnism | Methanothermus fervidus |
| 1 | Cellular orgnism | Methanothermus fervidus DSM 2088 |
| 1 | Cellular orgnism | Nanoarchaeum equitans Kin4-M |
| 1 | Cellular orgnism | Natrialba magadii |
| 1 | Cellular orgnism | Natrialba magadii ATCC 43099 |
| 1 | Cellular orgnism | Natrinema sp. CX2021 |
| 1 | Cellular orgnism | Natronobacterium sp. AS-7091 |
| 1 | Cellular orgnism | Natronomonas pharaonis |
| 1 | Cellular orgnism | Natronomonas pharaonis DSM 2160 |
| 1 | Cellular orgnism | Nitrosopumilus maritimus |
| 1 | Cellular orgnism | Nitrosopumilus maritimus SCM1 |
| 1 | Cellular orgnism | Picrophilus torridus |
| 1 | Cellular orgnism | Picrophilus torridus DSM 9790 |
| 1 | Cellular orgnism | Pyrobaculum aerophilum |
| 1 | Cellular orgnism | Pyrobaculum aerophilum str. IM2 |
| 1 | Cellular orgnism | Pyrobaculum arsenaticum |
| 1 | Cellular orgnism | Pyrobaculum arsenaticum DSM 13514 |
| 1 | Cellular orgnism | Pyrobaculum calidifontis |
| 1 | Cellular orgnism | Pyrobaculum calidifontis JCM 11548 |
| 1 | Cellular orgnism | Pyrobaculum islandicum |
| 1 | Cellular orgnism | Pyrobaculum islandicum DSM 4184 |
| 1 | Cellular orgnism | Pyrococcus abyssi |
| 1 | Cellular orgnism | Pyrococcus abyssi GE5 |
| 1 | Cellular orgnism | Pyrococcus furiosus |
| 1 | Cellular orgnism | Pyrococcus furiosus DSM 3638 |
| 1 | Cellular orgnism | Pyrococcus horikoshii |
| 1 | Cellular orgnism | Pyrococcus horikoshii OT3 |
| 1 | Cellular orgnism | Pyrococcus sp. 12/1 |
| 1 | Cellular orgnism | Pyrococcus sp. JT1 |
| 1 | Cellular orgnism | Staphylothermus hellenicus |
| 1 | Cellular orgnism | Staphylothermus hellenicus DSM 12710 |
| 1 | Cellular orgnism | Staphylothermus marinus |
| 1 | Cellular orgnism | Staphylothermus marinus F1 |
| 1 | Cellular orgnism | Sulfolobus acidocaldarius |
| 1 | Cellular orgnism | Sulfolobus acidocaldarius DSM 639 |
| 1 | Cellular orgnism | Sulfolobus islandicus |
| 1 | Cellular orgnism | Sulfolobus islandicus L.D.8.5 |
| 1 | Cellular orgnism | Sulfolobus islandicus L.S.2.15 |
| 1 | Cellular orgnism | Sulfolobus islandicus M.14.25 |
| 1 | Cellular orgnism | Sulfolobus islandicus M.16.27 |
| 1 | Cellular orgnism | Sulfolobus islandicus M.16.4 |
| 1 | Cellular orgnism | Sulfolobus islandicus Y.G.57.14 |
| 1 | Cellular orgnism | Sulfolobus islandicus Y.N.15.51 |
| 1 | Cellular orgnism | Sulfolobus neozealandicus |
| 1 | Cellular orgnism | Sulfolobus solfataricus |
| 1 | Cellular orgnism | Sulfolobus solfataricus 98/2 |
| 1 | Cellular orgnism | Sulfolobus solfataricus P2 |
| 1 | Cellular orgnism | Sulfolobus sp. NOB8H2 |
| 1 | Cellular orgnism | Sulfolobus tengchongensis |
| 1 | Cellular orgnism | Sulfolobus tokodaii |
| 1 | Cellular orgnism | Sulfolobus tokodaii str. 7 |
| 1 | Cellular orgnism | Thermococcus barophilus MP |
| 1 | Cellular orgnism | Thermococcus gammatolerans EJ3 |
| 1 | Cellular orgnism | Thermococcus kodakarensis KOD1 |
| 1 | Cellular orgnism | Thermococcus nautilus |
| 1 | Cellular orgnism | Thermococcus onnurineus |
| 1 | Cellular orgnism | Thermococcus onnurineus NA1 |
| 1 | Cellular orgnism | Thermococcus sibiricus MM 739 |
| 1 | Cellular orgnism | Thermococcus sp. 26/2 |
| 1 | Cellular orgnism | Thermococcus sp. AM4 |
| 1 | Cellular orgnism | Thermococcus sp. AMT11 |
| 1 | Cellular orgnism | Thermofilum pendens |
| 1 | Cellular orgnism | Thermofilum pendens Hrk 5 |
| 1 | Cellular orgnism | Thermoplasma acidophilum |
| 1 | Cellular orgnism | Thermoplasma acidophilum DSM 1728 |
| 1 | Cellular orgnism | Thermoplasma volcanium |
| 1 | Cellular orgnism | Thermoplasma volcanium GSS1 |
| 1 | Cellular orgnism | Thermoproteus neutrophilus |
| 1 | Cellular orgnism | Thermoproteus neutrophilus V24Sta |
| 1 | Cellular orgnism | Thermosphaera aggregans DSM 11486 |
| 1 | Cellular orgnism | Vulcanisaeta distributa |
| 1 | Cellular orgnism | Vulcanisaeta distributa DSM 14429 |
| 1 | Cellular orgnism | uncultured methanogenic archaeon RC-I |
| 2 | Eukaryota | Arabidopsis thaliana |
| 2 | Eukaryota | Chlamydomonas reinhardtii |
| 2 | Eukaryota | Cucumis sativus |
| 2 | Eukaryota | Cyanidioschyzon merolae strain 10D |
| 2 | Eukaryota | Cyanophora paradoxa |
| 2 | Eukaryota | Dictyostelium discoideum AX4 |
| 2 | Eukaryota | Entamoeba histolytica HM-1:IMSS |
| 2 | Eukaryota | Giardia lamblia ATCC 50803 |
| 2 | Eukaryota | Leishmania braziliensis MHOM/BR/75/M2904 |
| 2 | Eukaryota | Paramecium tetraurelia strain d4-2 |
| 2 | Eukaryota | Physcomitrella patens subsp. patens |
| 2 | Eukaryota | Phytophthora infestans T30-4 |
| 2 | Eukaryota | Picea sitchensis |
| 2 | Eukaryota | Plasmodium falciparum 3D7 |
| 2 | Eukaryota | Tetrahymena thermophila |
| 2 | Eukaryota | Thalassiosira pseudonana CCMP1335 |
| 2 | Eukaryota | Toxoplasma gondii ME49 |
| 2 | Eukaryota | Trichomonas vaginalis G3 |
| 2 | Eukaryota | Trypanosoma brucei TREU927 |
| 3 | Opisthokonts | Ashbya gossypii ATCC 10895 |
| 3 | Opisthokonts | Aspergillus fumigatus Af293 |
| 3 | Opisthokonts | Aspergillus nidulans FGSC A4 |
| 3 | Opisthokonts | Candida dubliniensis CD36 |
| 3 | Opisthokonts | Candida glabrata CBS 138 |
| 3 | Opisthokonts | Cryptococcus neoformans var. neoformans JEC21 |
| 3 | Opisthokonts | Debaryomyces hansenii CBS767 |
| 3 | Opisthokonts | Encephalitozoon cuniculi GB-M1 |
| 3 | Opisthokonts | Gibberella zeae PH-1 |
| 3 | Opisthokonts | Kluyveromyces lactis NRRL Y-1140 |
| 3 | Opisthokonts | Magnaporthe oryzae 70-15 |
| 3 | Opisthokonts | Monosiga brevicollis MX1 |
| 3 | Opisthokonts | Neurospora crassa OR74A |
| 3 | Opisthokonts | Pichia pastoris GS115 |
| 3 | Opisthokonts | Saccharomyces cerevisiae S288c |
| 3 | Opisthokonts | Scheffersomyces stipitis CBS 6054 |
| 3 | Opisthokonts | Schizosaccharomyces pombe |
| 3 | Opisthokonts | Ustilago maydis 521 |
| 3 | Opisthokonts | Yarrowia lipolytica CLIB122 |
| 4 | Metazoa | Amphimedon queenslandica |
| 5 | Eumetazoa | Hydra magnipapillata |
| 5 | Eumetazoa | Nematostella vectensis |
| 5 | Eumetazoa | Trichoplax adhaerens |
| 6 | Bilateria | Bos taurus |
| 6 | Bilateria | Canis lupus familiaris |
| 6 | Bilateria | Danio rerio |
| 6 | Bilateria | Gallus gallus |
| 6 | Bilateria | Homo sapiens |
| 6 | Bilateria | Mus musculus |
| 6 | Bilateria | Pan troglodytes |
| 6 | Bilateria | Rattus norvegicus |
| 6 | Bilateria | Saccoglossus kowalevskii |
| 6 | Bilateria | Strongylocentrotus purpuratus |
| 6 | Bilateria | Tetraodon nigroviridis |
| 6 | Bilateria | Xenopus laevis |
| 7 | Protostomia | Acyrthosiphon pisum |
| 7 | Protostomia | Apis mellifera |
| 7 | Protostomia | Caenorhabditis elegans |
| 7 | Protostomia | Daphnia pulex |
| 7 | Protostomia | Drosophila melanogaster |
| 8 | Lophotrochozoa | Capitella teleta |
| 8 | Lophotrochozoa | *Helobdella robusta* |
| 8 | Lophotrochozoa | *Schistosoma japonicum* |
| 9 | Mollusca | *Aplysia californica* |
| 9 | Mollusca | *Lottia gigantea* |
| 10 | Bivalvia | *Crassostrea gigas* |

**Supplementary Table 2 Samples and data used in this study**

1. **Biotic stress RNA-seq samples and alignment statistics.**

| **Challenge** | **Samples Description** | **Biological Sample ID** | **Tissue** | **Gram stain/virus** | **Total reads(M)** | **Mappable reads(M)** | **Mappable**  **(%)** | **mRNA** | | |
| --- | --- | --- | --- | --- | --- | --- | --- | --- | --- | --- |
|  |  |  |  |  |  |  |  | **rpkm>0** | **rpkm>=1** | **rpkm>=5** |
| *Vibrio* challenge time series* | 0h after *Vibrio* challenge | V0h** | Gill | G^-^ | 16.66 | 12.19 | 73.19 | 23960 | 20363 | 15132 |
|  | 6h after *Vibrio* challenge | V6h | Gill | G^-^ | 17.09 | 12.34 | 72.19 | 23965 | 20081 | 14590 |
|  | 12h after *Vibrio* challenge | V12h | Gill | G^-^ | 16.1 | 11.73 | 72.89 | 23838 | 20252 | 14815 |
|  | 24h after *Vibrio* challenge | V24h | Gill | G^-^ | 17.19 | 12.57 | 73.12 | 23927 | 20448 | 15289 |
|  | 48h after *Vibrio* challenge | V48h | Gill | G^-^ | 17.45 | 12.83 | 73.54 | 24029 | 20479 | 15364 |
| Control, no injection | Control-1, no injection | Control | Gill | - | 14.98 | 10.88 | 72.63 | 23648 | 20135 | 15005 |
| Challenge 12h  with different agents | Control-2, challenge 12h with PBS | PBS** | Gill | - | 14.49 | 10.56 | 72.91 | 23602 | 19835 | 14548 |
|  | Challenge 12h with LPS | LPS | Gill | - | 13.61 | 9.96 | 73.21 | 23639 | 19973 | 14445 |
|  | Challenge 12h with *V. aestuarianus* | V. aes | Gill | G^-^ | 15.59 | 11.34 | 72.75 | 23676 | 19856 | 14311 |
|  | Challenge 12h with *V. anguillarum* | V. ang | Gill | G^-^ | 15.07 | 11.05 | 73.29 | 23759 | 20083 | 14814 |
|  | Challenge 12h with *V. alginolyticus*-1 | V. alg 1 | Gill | G^-^ | 14.8 | 10.65 | 71.93 | 23953 | 20163 | 14525 |
|  | Challenge 12h with *V. alginolyticus*-2 | V. alg 2 | Gill | G^-^ | 15.17 | 11.09 | 73.07 | 23730 | 19695 | 13860 |
|  | Challenge 12h with *V. tubiashii* | V. tub | Gill | G^-^ | 13.47 | 9.84 | 73.06 | 23402 | 19912 | 14603 |
|  | Challenge 12h with *Micrococcus luteus* | M. lut | Gill | G^+^ | 15.68 | 11.46 | 73.11 | 23778 | 19981 | 14772 |
| Virus infection time series | Baseline of virus infection experiment | VirBase** | larvae | OsHV-1 | 24.25 | 19.71 | 81.29 | 24209 | 19201 | 13478 |
|  | 1 day after Virus infection | Vir1d | larvae | OsHV-1 | 26.65 | 21.48 | 80.61 | 24564 | 19541 | 13903 |
|  | 2 days after Virus infection | Vir2d | larvae | OsHV-1 | 11.69 | 9.29 | 79.49 | 23333 | 19277 | 13623 |

*: An equal mixture of four pathogenic *Vibrio* species/strains (*V. anguillarum, V. tubiashii, V. aestuarianus, V. alginolyticus* grown separately)

**: Samples used as control for differential expressed gene analyses.

1. **Transcriptome data from different organs, developmental stages and samples subjected to abiotic challenges of Zhang et al. (Nature, 2012)**

| **Condition** | | **Time** | **Control** | **Organ** | **RNA-seq** |
| --- | --- | --- | --- | --- | --- |
| Tissue | mantle, gill, adductor muscle, digestive gland, hemocyte,  labial palp, female gonad, male gonad | - | - | - | 8 |
| Development | eggs, cleavage, embryogenesis, trochophore, D-shape larvae | | - | Larvae, all | 24 |
| Exposure to air |  | 0, 1, 3, 5, 7, 9, 10, 11 d | 0 d | Gill, adductor muscle | 15 |
| Salinity | 5, 10, 15, 20, 25, 30,40 ppt | 7 d | 30 ppt | Gill | 7 |
| Temperature | 30, 35 °C | 12 h | 20 °C | Gill | 2 |
|  | 5, 10, 15, 20, 25 °C | 7 d |  | Gill | 5 |

**Supplementary Table 3** **GO enrichment in genes differentially expressed under *Vibrio* challenge at different time points, after seven types of biotic challenges and under virus challenge**

1. GO enrichment in genes differentially expressed under Vibrio challenge at different time points

| **ID** | **Description** | **Class** | **P value** |
| --- | --- | --- | --- |
| GO:0009408 | response to heat | BP | 0.000641 |
| GO:0005576 | extracellular region | CC | 0.000641 |
| GO:0004478 | methionine adenosyltransferase activity | MF | 0.010717 |
| GO:0015321 | sodium-dependent phosphate transmembrane transporter activity | MF | 0.017241 |
| GO:0004725 | protein tyrosine phosphatase activity | MF | 0.026268 |
| GO:0006817 | phosphate transport | BP | 0.034523 |
| GO:0008237 | metallopeptidase activity | MF | 0.039687 |
| GO:0008509 | anion transmembrane transporter activity | MF | 0.041012 |
| GO:0006820 | anion transport | BP | 0.042267 |
| GO:0005125 | cytokine activity | MF | 0.043152 |
| GO:0006954 | inflammatory response | BP | 0.043152 |
| GO:0006259 | DNA metabolic process | BP | 3.23E-06 |
| GO:0006260 | DNA replication | BP | 0.000201 |
| GO:0009262 | deoxyribonucleotide metabolic process | BP | 0.002078 |
| GO:0009219 | pyrimidine deoxyribonucleotide metabolic process | BP | 0.02111 |
| GO:0004478 | methionine adenosyltransferase activity | MF | 0.005114 |
| GO:0070011 | peptidase activity, acting on L-amino acid peptides | MF | 0.005114 |
| GO:0008237 | metallopeptidase activity | MF | 0.02119 |
| GO:0007160 | cell-matrix adhesion | BP | 0.02119 |
| GO:0006261 | DNA-dependent DNA replication | BP | 2.42E-05 |
| GO:0006259 | DNA metabolic process | BP | 0.003103 |
| GO:0016888 | endodeoxyribonuclease activity, producing 5'-phosphomonoesters | MF | 0.026202 |
| GO:0003715 | transcription termination factor activity | MF | 0.026202 |
| GO:0006353 | transcription termination, DNA-dependent | BP | 0.026202 |
| GO:0004185 | serine-type carboxypeptidase activity | MF | 0.026202 |
| GO:0008833 | deoxyribonuclease IV (phage-T4-induced) activity | MF | 0.026202 |
| GO:0040007 | growth | BP | 0.04497 |
| GO:0005102 | receptor binding | MF | 0.018946 |
| None |  |  |  |
| GO:0005102 | receptor binding | MF | 0.011424 |
| GO:0000785 | chromatin | CC | 0.047965 |
| GO:0006333 | chromatin assembly or disassembly | BP | 0.047965 |
| GO:0016702 | oxidoreductase activity, acting on single donors with incorporation of molecular oxygen, incorporation of two atoms of oxygen | MF | 0.035247 |
| GO:0003715 | transcription termination factor activity | MF | 0.035247 |
| GO:0008484 | sulfuric ester hydrolase activity | MF | 0.049713 |

1. GO enrichment in genes differentially expressed at 12h after seven types of biotic challenges

| **ID** | | **Description** | **Class** | **P-value** |
| --- | --- | --- | --- | --- |
|  |  | | | |
| GO:0005102 | | receptor binding | MF | 0.015976 |
| GO:0035556 | | intracellular signal transduction | BP | 0.015976 |
| GO:0007264 | | small GTPase mediated signal transduction | BP | 0.024012 |
|  |  | | | |
| GO:0005319 | | lipid transporter activity | MF | 1.42E-06 |
| GO:0006801 | | superoxide metabolic process | BP | 0.000499 |
| GO:0044085 | | cellular component biogenesis | BP | 0.023016 |
| GO:0071841 | | cellular component organization or biogenesis at cellular level | BP | 0.023016 |
| GO:0055114 | | oxidation-reduction process | BP | 0.030171 |
| GO:0000785 | | chromatin | CC | 0.034232 |
| GO:0034622 | | cellular macromolecular complex assembly | BP | 0.034232 |
| GO:0006333 | | chromatin assembly or disassembly | BP | 0.034232 |
| GO:0000786 | | nucleosome | CC | 0.034232 |
| GO:0006270 | | DNA-dependent DNA replication initiation | BP | 0.034232 |
| GO:0006334 | | nucleosome assembly | BP | 0.034232 |
| GO:0004579 | | dolichyl-diphosphooligosaccharide-protein glycotransferase activity | MF | 0.048137 |
|  |  | | | |
| GO:0003715 | | transcription termination factor activity | MF | 0.023467 |
| GO:0004800 | | thyroxine 5'-deiodinase activity | MF | 0.040363 |
| GO:0007160 | | cell-matrix adhesion | BP | 0.049926 |
|  |  | | | |
| GO:0006801 | | superoxide metabolic process | BP | 0.000119 |
| GO:0072593 | | reactive oxygen species metabolic process | BP | 0.000119 |
| GO:0005319 | | lipid transporter activity | MF | 0.000799 |
| GO:0006869 | | lipid transport | BP | 0.003813 |
| GO:0010876 | | lipid localization | BP | 0.003813 |
|  |  | | | |
| GO:0005102 | | receptor binding | MF | 0.005468 |
| GO:0005525 | | GTP binding | MF | 0.005468 |
| GO:0005164 | | tumor necrosis factor receptor binding | MF | 0.005468 |
| GO:0006955 | | immune response | BP | 0.027203 |
| GO:0004817 | | cysteine-tRNA ligase activity | MF | 0.048357 |
| GO:0006423 | | cysteinyl-tRNA aminoacylation | BP | 0.048357 |
|  |  | | | |
| GO:0005319 | | lipid transporter activity | MF | 8.16E-05 |
| GO:0006950 | | response to stress | BP | 0.000425 |
| GO:0006801 | | superoxide metabolic process | BP | 0.001694 |
| GO:0008833 | | deoxyribonuclease IV (phage-T4-induced) activity | MF | 0.012248 |
|  |  | | | |
| GO:0005102 | | receptor binding | MF | 0.011601 |
| GO:0006955 | | immune response | BP | 0.030396 |
|  |  | | | |
| GO:0004576 | | oligosaccharyl transferase activity | MF | 1.18E-07 |
| GO:0005319 | | lipid transporter activity | MF | 1.16E-06 |
| GO:0006270 | | DNA-dependent DNA replication initiation | BP | 7.58E-06 |
| GO:0004579 | | dolichyl-diphosphooligosaccharide-protein glycotransferase activity | MF | 3.62E-05 |
| GO:0006869 | | lipid transport | BP | 3.62E-05 |
| GO:0033036 | | macromolecule localization | BP | 3.62E-05 |
| GO:0006801 | | superoxide metabolic process | BP | 0.000342 |
| GO:0006396 | | RNA processing | BP | 0.000557 |
| GO:0070972 | | protein localization in endoplasmic reticulum | BP | 0.001131 |
| GO:0015035 | | protein disulfide oxidoreductase activity | MF | 0.001228 |
| GO:0016667 | | oxidoreductase activity, acting on a sulfur group of donors | MF | 0.001228 |
| GO:0006662 | | glycerol ether metabolic process | BP | 0.003357 |
| GO:0008312 | | 7S RNA binding | MF | 0.004243 |
| GO:0048500 | | signal recognition particle | CC | 0.004243 |
| GO:0033365 | | protein localization to organelle | BP | 0.004446 |
| GO:0006612 | | protein targeting to membrane | BP | 0.007869 |
| GO:0045454 | | cell redox homeostasis | BP | 0.008088 |
| GO:0072594 | | establishment of protein localization to organelle | BP | 0.009452 |
| GO:0003723 | | RNA binding | MF | 0.009452 |
| GO:0006614 | | SRP-dependent cotranslational protein targeting to membrane | BP | 0.009452 |
| GO:0009055 | | electron carrier activity | MF | 0.009452 |
| GO:0006457 | | protein folding | BP | 0.009452 |
| GO:0008250 | | oligosaccharyltransferase complex | CC | 0.009452 |
| GO:0018279 | | protein N-linked glycosylation via asparagine | BP | 0.009452 |
| GO:0045900 | | negative regulation of translational elongation | BP | 0.009452 |
| GO:0005048 | | signal sequence binding | MF | 0.009452 |
| GO:0005741 | | mitochondrial outer membrane | CC | 0.009511 |
| GO:0005783 | | endoplasmic reticulum | CC | 0.009511 |
| GO:0055114 | | oxidation-reduction process | BP | 0.009519 |
| GO:0008152 | | metabolic process | BP | 0.009542 |
| GO:0003676 | | nucleic acid binding | MF | 0.011243 |
| GO:0003840 | | gamma-glutamyltransferase activity | MF | 0.011847 |
| GO:0008745 | | N-acetylmuramoyl-L-alanine amidase activity | MF | 0.011847 |
| GO:0009253 | | peptidoglycan catabolic process | BP | 0.011847 |
| GO:0065008 | | regulation of biological quality | BP | 0.014648 |
| GO:0006260 | | DNA replication | BP | 0.015262 |
| GO:0006364 | | rRNA processing | BP | 0.023869 |
| GO:0034470 | | ncRNA processing | BP | 0.026143 |
| GO:0005737 | | cytoplasm | CC | 0.029405 |
| GO:0044444 | | cytoplasmic part | CC | 0.042489 |
| GO:0030684 | | preribosome | CC | 0.043377 |
| GO:0006486 | | protein glycosylation | BP | 0.045562 |
|  |  | | | |
| GO:0016717 | | oxidoreductase activity, acting on paired donors, with oxidation of a pair of donors resulting in the reduction of molecular oxygen to two molecules of water | MF | 0.003975 |
| GO:0004725 | | protein tyrosine phosphatase activity | MF | 0.016908 |
| GO:0005525 | | GTP binding | MF | 0.016908 |
| GO:0035556 | | intracellular signal transduction | BP | 0.024644 |
| GO:0007264 | | small GTPase mediated signal transduction | BP | 0.032557 |
| GO:0004478 | | methionine adenosyltransferase activity | MF | 0.032557 |
| GO:0008374 | | O-acyltransferase activity | MF | 0.032557 |
| GO:0046914 | | transition metal ion binding | MF | 0.036015 |
|  |  | | | |
| GO:0005319 | | lipid transporter activity | MF | 4.02E-05 |
| GO:0003723 | | RNA binding | MF | 0.000503 |
| GO:0006869 | | lipid transport | BP | 0.00175 |
| GO:0003676 | | nucleic acid binding | MF | 0.002452 |
| GO:0006270 | | DNA-dependent DNA replication initiation | BP | 0.00797 |
| GO:0006457 | | protein folding | BP | 0.025289 |
| GO:0051082 | | unfolded protein binding | MF | 0.025289 |
| GO:0071702 | | organic substance transport | BP | 0.032036 |
|  |  | | | |
| GO:0016986 | | transcription initiation factor activity | MF | 7.89E-08 |
| GO:0006352 | | transcription initiation, DNA-dependent | BP | 6.24E-05 |
| GO:0005319 | | lipid transporter activity | MF | 7.27E-05 |
| GO:0006869 | | lipid transport | BP | 7.27E-05 |
| GO:0006801 | | superoxide metabolic process | BP | 8.40E-05 |
| GO:0000786 | | nucleosome | CC | 0.001854 |
| GO:0006334 | | nucleosome assembly | BP | 0.001854 |
| GO:0042719 | | mitochondrial intermembrane space protein transporter complex | CC | 0.038404 |
| GO:0045039 | | protein import into mitochondrial inner membrane | BP | 0.038404 |
|  |  | | | |
| GO:0005102 | | receptor binding | MF | 0.000251 |
|  |  | | | |
| GO:0005319 | | lipid transporter activity | MF | 0.001063 |
| GO:0006869 | | lipid transport | BP | 0.005174 |
| GO:0006952 | | defense response | BP | 0.0267 |
| GO:0006955 | | immune response | BP | 0.031121 |
| GO:0004518 | | nuclease activity | MF | 0.032238 |
| GO:0045087 | | innate immune response | BP | 0.033386 |
| GO:0006950 | | response to stress | BP | 0.049277 |
